# Supplementary material for: Anti-Markovnikov hydro(amino)alkylation of vinylarenes via photoredox catalysis
Source: Nat Commun. 2021 Oct 12;12:5956. doi: 10.1038/s41467-021-26170-6 (PMC8511241; doi:10.1038/s41467-021-26170-6)
Supplement: Supplementary file 1 — Supplementary Information [file 41467_2021_26170_MOESM1_ESM.pdf]

Anti-Markovnikov Hydro(amino)alkylation Vinaylarenes *via* Photoredox Catalysis

Zhao Wu,[†,‡] Samuel N. Gockel,[†,§,‡] and Kami L. Hull*[,§]

[†] Department of Chemistry, University of Illinois at Urbana–Champaign, Urbana, IL 61801, United States

[§] Department of Chemistry, University of Texas at Austin, Austin, TX 78712, United States

[‡] These authors contribute equally.

Supplementary Information

Table of Contents

I. Supplementary Methods

A. General Information **2**

B. Substrate Synthesis **3**

C. Reaction Set-up for Photoredox Catalysis **16**

D. Selected Optimization Results **17**

E. Mechanistic Studies **36**

F. Stern-Volmer Experiments **42**

G. Experimental Procedure, Isolation, and Characterization **45**

H. Spectra **75**

II. Supplementary References **160**

I. Supplementary Methods

A. General Information

General Experimental Procedures: All reactions were carried out in flame-dried (or oven-dried at 140 °C for at least 2 h) glassware under an atmosphere of nitrogen unless otherwise indicated. Nitrogen was dried using a drying tube equipped with Drierite™ unless otherwise noted. Air- and moisture-sensitive reagents were handled in a nitrogen-filled glovebox (working oxygen level ~ 0.1 ppm). Column chromatography was performed with 1) basic aluminium oxide from ACROS Organics (50-200 μm, 60 A), Brockmann I grade, activated upon addition of certain amount of water according to the substrates, dry loading of activated aluminium oxide was applied followed by flush with eluent to get rid of air bubbles; 2) silica gel from Grace Davison Discovery Sciences (35-75 μm) with a column mixed as a slurry with the eluent and was packed, rinsed, and run under air pressure. Analytical thin-layer chromatography (TLC) was performed on precoated glass silica gel plates (by EMD Chemicals Inc.) with F-254 indicator. Visualization was either by short wave (254 nm) ultraviolet light, or by staining with potassium permanganate followed by brief heating on a hot plate or by a heat gun. Distillations were performed using a 3 cm short-path column under reduced pressure or by using a Hickman still at ambient pressure.

Instrumentation: ^{1}H NMR and ^{13}C NMR were recorded on a Varian Unity 400/500 MHz (100/125 MHz respectively for ^{13}C) or a VXR-500 MHz spectrometer. Spectra were referenced using either $CDCl_3$ or C_6D_6 as solvents (unless otherwise noted) with the residual solvent peak as the internal standard (^{1}H NMR: δ 7.26 ppm, ^{13}C NMR: δ 77.00 ppm for $CDCl_3$ and ^{1}H NMR: δ 7.15 ppm, ^{13}C NMR: δ 128.60 ppm for C_6D_6). Chemical shifts were reported in parts per million and multiplicities are as indicated: s (singlet,) d (doublet,) t (triplet,) q (quartet,) p (pentet,) m (multiplet,) and br (broad). Coupling constants, *J*, are reported in Hertz and integration is provided. Analysis by Gas Chromatography-Mass Spectrometry (GC-MS) was performed using a Shimadzu GC-2010 Plus Gas chromatograph fitted with a Shimadzu GCMS-QP2010 SE mass spectrometer using electron impact (EI) ionization after analytes traveled through a SHRXI–5MS- 30m x 0.25 mm x 0.25 μm column using a helium carrier gas. Data are reported in the form of m/z (intensity relative to base peak = 100). Gas Chromatography (GC) was performed on a Shimadzu GC-2010 Plus gas chromatograph with SHRXI–MS- 15m x 0.25 mm x 0.25 μm column with nitrogen carrier gas and a flame ionization detector (FID). Low-resolution Mass Spectrometry and High Resolution Mass Spectrometry were performed in the Department of Chemistry at University of Illinois at Urbana-Champaign. The glove box, MBraun LABmaster sp, was maintained under nitrogen atmosphere. Melting points were recorded on a Thomas Hoover capillary melting point apparatus.

Materials: Solvents used for extraction and column chromatography were reagent grade and used as received. Reaction solvents tetrahydrofuran (Fisher, unstabilized HPLC ACS grade), diethyl ether (Fisher, BHT stabilized ACS grade), methylene chloride (Fisher, unstabilized HPLC grade), dimethoxyethane (Fisher, certified ACS), toluene (Fisher, optima ACS grade), 1,4-dioxane (Fisher, certified ACS), acetonitrile (Fisher, HPLC grade), and hexanes (Fisher, ACS HPLC grade) were dried on a Pure Process Technology Glass Contour Solvent Purification System using activated Stainless Steel columns while following manufacture's recommendations for solvent preparation and dispensation unless otherwise noted.

B. Substrate synthesis

Alkene Synthesis

Unless prepared according to the following procedure, alkene substrates were distilled after received from commercial sources.

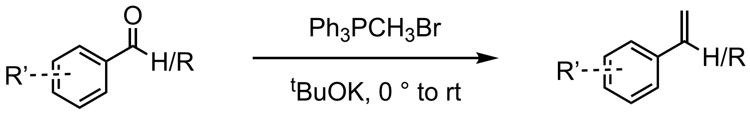

Procedure: To a dry 100 mL three-neck flask was charged with a stir bar and 5.4 g Ph_3PCH_3Br (15 mmol, 2.5 equiv) purged with nitrogen followed by the addition of 30 mL dry THF. Cooled to 0 °C, 1.68 g ^tBuOK (15 mmol, 2.5 equiv) was added under nitrogen flow in one portion. The reaction was allowed to stir at 0 °C for 30 min followed by the slow addition of the aldehyde or ketone (6.0 mmol, 1.0 equiv). The reaction flask was then warmed up to rt and stir overnight. The reaction was quenched by water and extracted with EtOAc three time, then the combined organic layers were washed with brine, dried by $MgSO_4$, concentrated *in vacuo*, and purified by flash column chromatography on silica gel to afford the desire alkenes.

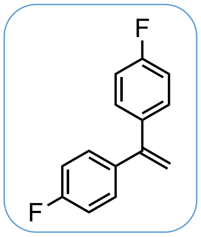

4,4'-(ethene-1,1-diyl)bis(fluorobenzene): prepared according to previously described procedure in 79% yield.

Column Chromatography Condition: 30 : 1 hexane/EtOAc

^{1}**H NMR** (500 MHz, $CDCl_3$) δ: 7.37 – 7.28 (m, 4H), 7.05 (t, J = 8.7 Hz, 4H), 5.41 (s, 2H).

^{13}**C NMR** (125 MHz, $CDCl_3$) δ: 162.71 (d, J = 247.0 Hz), 148.20, 137.51 (d, J = 3.3 Hz), 129.95 (d, J = 8.0 Hz), 115.27 (d, J = 21.4 Hz), 114.28.

^{19}**F NMR** (471 MHz, $CDCl_3$) δ: -114.40 – -114.53 (m).

HRMS (ESI-TOF) *m/z*: [M+H$^+$] calculated for $C_{14}H_{10}F_2$, 216.0751; found, 216.0747.

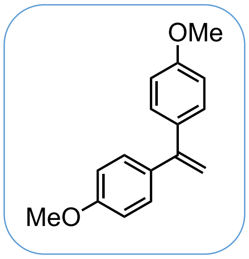

4,4'-(ethene-1,1-diyl)bis(methoxybenzene): prepared according to previously described procedure in 92% yield.

Column Chromatography Condition: 4 : 1 hexane/EtOAc

^{1}**H NMR** (500 MHz, $CDCl_3$) δ: 7.40 – 7.16 (m, 4H), 6.96 – 6.66 (m, 4H), 5.30 (s, 2H), 3.83 (s, 6H).

^{13}**C NMR** (125 MHz, $CDCl_3$) δ: 159.44, 149.12, 134.46, 129.57, 113.63, 111.81, 55.45.

HRMS (ESI-TOF) *m/z*: [M+H$^+$] calculated for $C_{16}H_{17}O_2$, 241.1229; found, 241.1222.

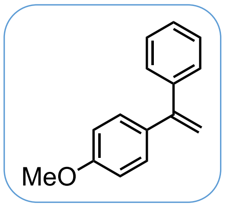

1-methoxy-4-(1-phenylvinyl)benzene: prepared according to previously described procedure in 76% yield.

Column Chromatography Condition: 10 : 1 hexane/EtOAc

^{1}H NMR (500 MHz, $CDCl_3$) δ: 7.37 – 7.31 (m, 5H), 7.30 – 7.26 (m, 2H), 6.91 – 6.84 (m, 2H), 5.40 (d, J = 1.3 Hz, 1H), 5.36 (d, J = 1.3 Hz, 1H), 3.83 (s, 3H).

^{13}C NMR (125 MHz, $CDCl_3$) δ: 159.40, 149.58, 141.91, 134.07, 129.52, 128.44, 128.25, 127.78, 113.61, 113.13, 55.44.

HRMS (ESI-TOF) *m/z*: [M+H$^+$] calculated for $C_{15}H_{15}O$, 211.1123; found, 211.1123.

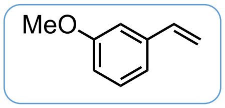

1-methoxy-3-vinylbenzene: prepared according to previously described procedure in 61% yield after distillation.

Column Chromatography Condition: 8 : 1 hexane/EtOAc

^{1}H NMR (500 MHz, $CDCl_3$) δ: 7.25 (t, J = 7.9 Hz, 1H), 7.02 (dt, J = 7.6, 1.2 Hz, 1H), 6.96 (dd, J = 2.6, 1.5 Hz, 1H), 6.82 (ddd, J = 8.3, 2.6, 1.0 Hz, 1H), 6.70 (dd, J = 17.6, 10.8 Hz, 1H), 5.75 (dd, J = 17.6, 0.9 Hz, 1H), 5.26 (dd, J = 10.9, 0.9 Hz, 1H), 3.83 (s, 3H).

^{13}C NMR (125 MHz, $CDCl_3$) δ: 159.95, 139.18, 136.92, 129.63, 119.05, 114.26, 113.58, 111.67, 55.37.

HRMS (ESI-TOF) *m/z*: [M+H$^+$] calculated for $C_9H_{11}O$, 135.0810; found, 135.0807.

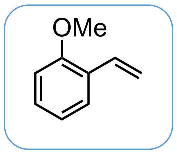

1-methoxy-2-vinylbenzene: prepared according to previously described procedure in 84% yield.

Column Chromatography Condition: 8 : 1 hexane/EtOAc

^{1}H NMR (500 MHz, $CDCl_3$) δ: 7.48 (dd, J = 7.6, 1.7 Hz, 1H), 7.25 (ddd, J = 8.3, 7.3, 1.7 Hz, 1H), 7.07 (dd, J = 17.8, 11.1 Hz, 1H), 6.99 – 6.92 (m, 1H), 6.88 (dd, J = 8.3, 1.1 Hz, 1H), 5.75 (dd, J = 17.8, 1.5 Hz, 1H), 5.28 (dd, J = 11.2, 1.5 Hz, 1H), 3.86 (s, 3H).

^{13}C NMR (125 MHz, $CDCl_3$) δ: 156.87, 131.82, 128.97, 126.92, 126.67, 120.75, 114.58, 110.98, 55.61.

HRMS (ESI-TOF) *m/z*: [M+H$^+$] calculated for $C_9H_{11}O$, 135.0810; found, 135.0814.

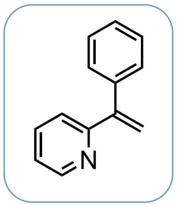

2-(1-phenylvinyl)pyridine: prepared according to previously described procedure in 64% yield.

Column Chromatography Condition: 4 : 1 hexane/EtOAc

^{1}H NMR (500 MHz, $CDCl_3$) δ: 8.65 (ddd, J = 4.9, 2.0, 1.0 Hz, 1H), 7.63 (td, J = 7.7, 1.8 Hz, 1H), 7.40 – 7.31 (m, 5H), 7.30 – 7.24 (m, 1H), 7.21 (ddd, J = 7.4, 4.8, 1.2 Hz, 1H), 6.09 – 5.94 (m, 1H), 5.72 – 5.54 (m, 1H).

^{13}C NMR (125 MHz, $CDCl_3$) δ: 158.69, 149.54, 149.34, 140.53, 136.40, 128.57, 128.43, 127.96, 122.97, 122.56, 117.84.

HRMS (ESI-TOF) *m/z*: [M+H$^+$] calculated for $C_{13}H_{12}N$, 182.0970; found, 182.0973.

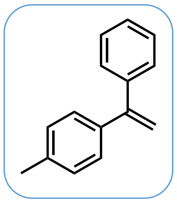

1-methyl-4-(1-phenylvinyl)benzene: prepared according to previously described procedure in 77% yield.

Column Chromatography Condition: 99 : 1 hexane/EtOAc

^{1}H NMR (500 MHz, $CDCl_3$) δ: 7.38 – 7.28 (m, 5H), 7.24 (d, J = 7.9 Hz, 2H), 7.15 (d, J = 7.9 Hz, 2H), 5.44 (d, J = 1.1 Hz, 1H), 5.41 (d, J = 1.3 Hz, 1H), 2.37 (s, 3H).

^{13}C NMR (125 MHz, $CDCl_3$) δ: 150.06, 141.84, 138.76, 137.65, 129.00, 128.43, 128.29, 128.25, 127.77, 113.76, 21.32.

HRMS (ESI-TOF) *m/z*: [M+H$^+$] calculated for $C_{15}H_{14}$, 194.1096; found, 194.1093.

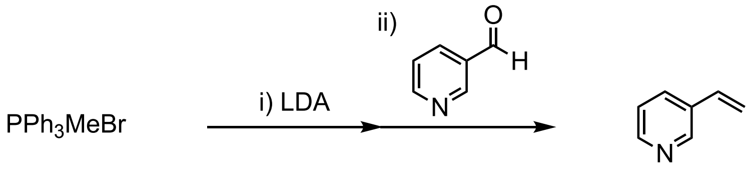

Procedure: A dry 100 mL schlenk flask was charged with a stir bar, then purged with nitrogen followed by the addition of 50 mL dry THF and 1.4 mL $HN(^iPr)_2$ (10 mmol, 1.0 equiv). Cooled to -78 °C, *n*-BuLi solution (10 mmol, 1.0 equiv) was added slowly into the flask. The reaction was then warmed up to room temperature and allowed to stir for 10 min. 3.57 g PPh_3MeBr (10 mmol, 1.0 equiv) was then added into the reaction in one portion under N_2 flow at 0 °C and the resulting crude was stirred at 0 °C for another 1 hour. After 1 hour, the reaction was cooled to -78 °C followed by the slow addition of 0.94 mL nicotinaldehyde (10 mmol, 1.0 equiv), and warmed up to room temperature and allowed to stir overnight. The reaction was quenched by the addition of 50 mL water and extracted with Et_2O three time, then the combined organic layers were dried by $MgSO_4$, concentrated *in vacuo*, and purified by flash column chromatography on silica gel (1:1 hexanes/Et_2O as eluent) to afford the desire alkene. (Caution: product is volatile, avoid the high-vac).

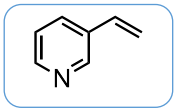

3-vinylpyridine: prepared according to previously described procedure in 75% yield (with some Et_2O and hexane residues)

Column Chromatography Condition: 1:1 hexane / Et_2O

^{1}H NMR (500 MHz, $CDCl_3$) δ: 8.61 (d, J = 2.3 Hz, 1H), 8.48 (dd, J = 4.8, 1.6 Hz, 1H), 7.72 (dt, J = 7.9, 2.0 Hz, 1H), 7.27 – 7.20 (m, 1H), 6.70 (dd, J = 17.7, 11.0 Hz, 1H), 5.82 (dd, J = 17.6, 0.7 Hz, 1H), 5.37 (dd, J = 11.0, 0.7 Hz, 1H).

^{13}C NMR (125 MHz, $CDCl_3$) δ: 149.03, 148.43, 133.61, 133.15, 132.74, 123.52, 116.32.

HRMS (ESI-TOF) *m/z*: [M+H$^+$] calculated for C_7H_8N, 106.0657; found, 106.0658.

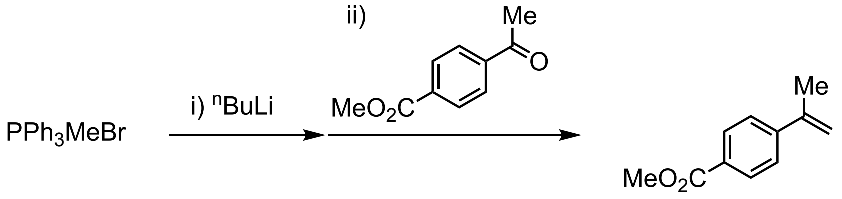

Procedure: A dry 100 mL schlenk flask was charged with a stir bar and 7.14 g PPh_3MeBr (20 mmol, 2.0 equiv), then purged with nitrogen followed by the addition of 50 mL dry THF. Cooled to 0 °C, *n*-BuLi solution (19 mmol, 1.9 equiv) was added slowly into the flask. The reaction was allowed to stir at 0 °C for 1 hour. 1.78 g starting material ketone (10 mmol, 1.0 equiv) was added into the reaction in one portion under N_2 flow at 0 °C then the resulting reaction crude was warmed up to room temperature and stirred overnight. The reaction was quenched by the addition of 50 mL water and extracted with Et_2O three time, then the combined organic layers were washed with brine and dried by $MgSO_4$, concentrated *in vacuo*, and purified by flash column chromatography on silica gel (10:1 to 5:1 hexanes/EtOAc as gradient eluent) to afford the product in 45% yield as a white solid.

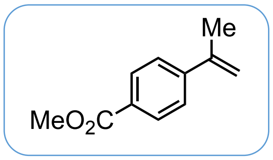

Ethyl 4-(prop-1-en-2-yl)benzoate. ^{1}**H NMR** (500 MHz, $CDCl_3$) δ: 7.99 (d, J = 8.2 Hz, 2H), 7.52 (d, J = 8.4 Hz, 2H), 5.47 (q, J = 1.0 Hz, 1H), 5.19 (p, J = 1.5 Hz, 1H), 3.92 (s, 3H), 2.23 – 2.08 (m, 3H).

^{13}**C NMR** (125 MHz, $CDCl_3$) δ: 167.07, 145.81, 142.62, 129.72, 129.09, 125.57, 114.69, 52.19, 21.78.

HRMS (ESI-TOF) *m/z*: [M+H$^+$] calculated for $C_{11}H_{13}O_2$, 177.0916; found, 177.0918.

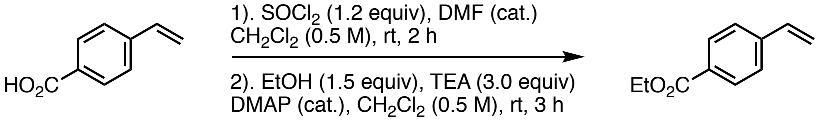

Procedure:[1] An oven-dried 250 mL round-bottomed flask equipped with a stir bar was charged with 4-vinylbenzoic acid (2.96 g, 20 mmol), anhydrous methylene chloride (40 mL, 0.50 M), and anhydrous DMF (100 µL). To the stirring solution under nitrogen is added thionyl chloride (1.75 mL, 24 mmol) in a dropwise manner. After stirring at rt for 2 h, the mixture is evaporated to dryness with the use of a rotary evaporator. Methylene chloride (40 mL, 0.50 M) is added, followed by ethanol (1.76 mL, 30 mmol) and 4-dimethylamino pyridine (20 mg). Then, in a dropwise manner, freshly distilled triethylamine (8.5 mL, 60 mmol) is added over 3 min. The mixture is allowed to stir at rt for 3 h after which point it is quenched by the addition of aqueous HCl (1 M). The mixture is transferred to a separatory funnel and extracted with ethyl acetate (3x25 mL). The combined organic layers are then washed with brine (1x10 mL), then dried with anhydrous MgSO4, filtered, and the solvent is removed under reduced pressure with the aid of a rotary evaporator. The product is further purified *via* flash chromatography on silica gel, eluting with 5% ethyl acetate in hexanes. The product is obtained as a clear oil (3.22 g, 91% yield). The spectral data were in accordance with those previously reported.[1]

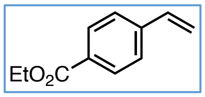

^{1}**H NMR** (500 MHz, Chloroform-*d*) δ 8.00 (d, *J* = 7.9 Hz, 2H), 7.46 (d, *J* = 8.3 Hz, 2H), 6.75 (dd, *J* = 17.6, 10.9 Hz, 1H), 5.86 (dd, *J* = 17.6, 0.7 Hz, 1H), 5.38 (dd, *J* = 10.9, 0.7 Hz, 1H), 4.38 (q, *J* = 7.1 Hz, 2H), 1.40 (t, *J* = 7.1 Hz, 3H).

^{13}C NMR (126 MHz, $CDCl_3$) δ 166.69, 142.10, 136.35, 130.13, 129.93, 126.34, 116.65, 61.20, 14.62.

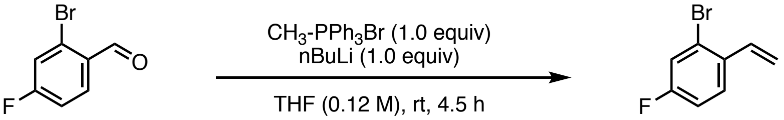

Procedure: An oven-dried 250 mL round-bottomed flask equipped with a stir bar was charged with methyltriphenylphosphonium bromide (3.57 g, 10 mmol) and anhydrous THF (83 mL, 0.12 M). The mixture was cooled to 0 ^{o}C with an ice bath, and ^{n}BuLi (1.6 M in hexanes, 6.25 mL, 10 mmol) was added dropwise over 1 min. The ice bath was removed and the yellow mixture was allowed to stir at rt for 25 min. A solution of the aldehyde (2.03 g, 10 mmol) in anhydrous THF (5 mL) was added *via* syringe and the mixture was allowed to stir at rt for 4.5 h at which point the reaction was deemed complete by TLC analysis. The reaction was then quenched with saturated aqueous NH4Cl. The mixture was transferred to a separatory funnel and the aqueous layer was washed with methylene chloride (3x50 mL). The combined organic layers were dried with anhydrous MgSO4 and then filtered. The solvent was removed under reduced pressure with the aid of a rotary evaporator. The product was further purified *via* flash column chromatography on silica gel, eluting with 5% ethyl acetate in hexanes. The product was obtained as a clear oil (1.41 g, 70% yield). The spectra matched those previously reported.[2]

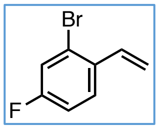

^{1}H NMR (499 MHz, Chloroform-*d*) δ 7.52 (dd, *J* = 8.7, 6.0 Hz, 1H), 7.30 (dd, *J* = 8.3, 2.6 Hz, 1H), 7.02 (td, *J* = 8.5, 2.7 Hz, 1H), 6.98 (dd, *J* = 17.5, 11.0 Hz, 1H), 5.64 (d, *J* = 17.4 Hz, 1H), 5.35 (d, *J* = 10.6 Hz, 1H).

^{13}C NMR (126 MHz, Chloroform-*d*) δ 161.83 (d, *J* = 251.3 Hz), 134.74, 133.85 (d, *J* = 3.7 Hz), 127.72 (d, *J* = 8.4 Hz), 123.44 (d, *J* = 9.3 Hz), 119.88 (d, *J* = 24.5 Hz), 116.51 (d, *J* = 2.0 Hz), 114.87 (d, *J* = 21.2 Hz).

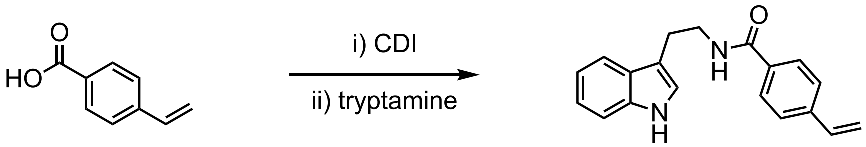

Procedure: A 100 mL round-bottom-flask was charged with a stir bar, 1.48 g 4-vinylbenzoic acid (10 mmol, 1.0 equiv), 1.78 g carbonyldiimidazole (11 mmol, 1.1 equiv) and 10 mL DCM. The mixture was then stirred at room temperature for 15 min until no gas formation. 1.68 g tryptamine (10.5 mmol, 1.05 equiv) was added into reaction in one portion. After 12 hours, the reaction crude was diluted in DCM and washed with 1 M HCl solution three times and 1M NaOH solution once. The organic layer was dried by $MgSO_4$, concentrated *in vacuo*, and re-crystalized in DCM to afford the white solid as pure product.

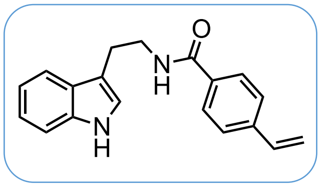

***N*-(2-(1H-indol-3-yl)ethyl)-4-vinylbenzamide:** prepared according to previously described procedure in 63% yield (first crop of recrystallization) as a white solid. **m.p.** = 134 °C.

^{1}H NMR (500 MHz, $CDCl_3$) δ: 8.12 (brs, 1H), 7.76 – 7.58 (m, 3H), 7.48 – 7.34 (m, 3H), 7.23 (ddd, J = 8.2, 7.0, 1.1 Hz, 1H), 7.14 (ddd, J = 8.0, 7.1, 1.0 Hz, 1H), 7.07 (d, J = 2.3 Hz, 1H), 6.72 (dd, J =

17.6, 10.9 Hz, 1H), 6.21 (brs, 1H), 5.81 (dd, J = 17.6, 0.8 Hz, 1H), 5.41 – 5.22 (m, 1H), 3.81 (q, J = 6.4 Hz, 2H), 3.11 (t, J = 6.6 Hz, 2H).

^{13}C NMR (125 MHz, $CDCl_3$) δ: 167.21, 140.62, 136.60, 136.07, 133.84, 127.46, 127.28, 126.38, 122.39, 122.27, 119.67, 118.87, 115.96, 113.12, 111.46, 40.45, 25.44.

HRMS (ESI-TOF) *m/z*: [M+H$^+$] calculated for $C_{19}H_{19}N_2O$, 291.1497; found, 291.1491.

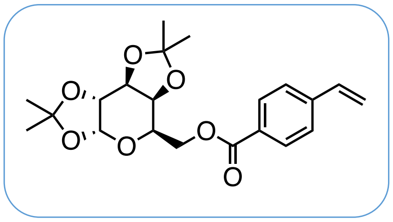

Prepared according to previously described procedure using 1,2:3,4-Di-O-isopropylide-ne-α-D-galactopyranose as nucleophile followed by silica column chromatography (4:1 to 2:1 hexanes/EtOAc as gradient eluent) in 82% yield as a white solid. **m.p.** = 68–69 °C.

^{1}H NMR (500 MHz, $CDCl_3$) δ: 8.01 (d, J = 8.3 Hz, 2H), 7.45 (d, J = 8.4 Hz, 2H), 6.75 (dd, J = 17.6, 10.9 Hz, 1H), 5.86 (d, J = 17.6 Hz, 1H), 5.57 (d, J = 4.9 Hz, 1H), 5.38 (d, J = 10.9 Hz, 1H), 4.65 (dd, J = 7.9, 2.2 Hz, 1H), 4.53 (dd, J = 11.5, 4.8 Hz, 1H), 4.42 (dd, J = 11.4, 7.5 Hz, 1H), 4.37 – 4.29 (m, 2H), 4.22 – 4.14 (m, 1H), 1.51 (s, 3H), 1.48 (s, 3H), 1.36 (s, 3H), 1.33 (s, 3H).

^{13}C NMR (125 MHz, $CDCl_3$) δ: 166.32, 142.14, 136.19, 130.16, 129.34, 126.23, 116.61, 109.84, 108.95, 96.48, 71.32, 70.90, 70.70, 66.34, 64.01, 26.18, 26.14, 25.14, 24.66.

HRMS (ESI-TOF) *m/z*: [M+H$^+$] calculated for $C_{21}H_{27}O_7$, 391.1757; found, 391.1748.

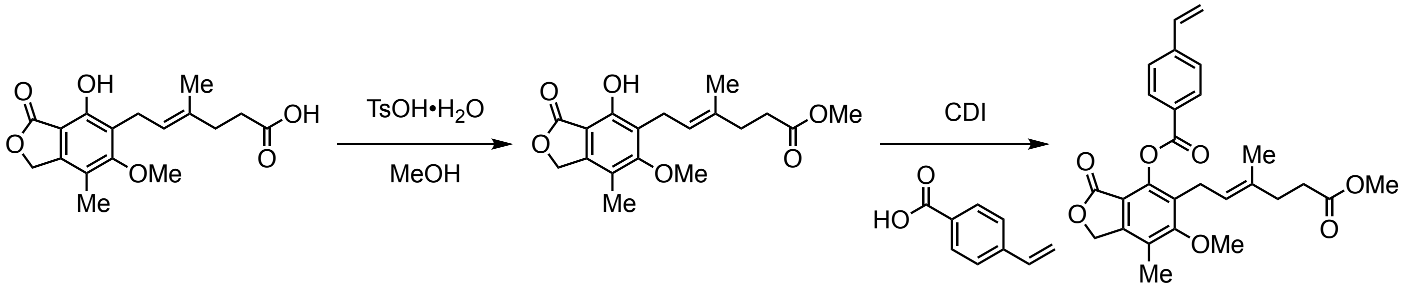

Esterification: A 100 mL round-bottom-flask was charged with a stir bar, 6.41 g mycophenolic acid (20 mmol, 1.0 equiv), 0.76 g $TsOH \cdot H_2O$ (4 mmol, 20 mol %) and 40 mL MeOH. The mixture was then stirred at room temperature overnight. The reaction crude was concentrated *in vacuo* to remove MeOH, then diluted in EtOAc and washed with sat. $NaHCO_3$ solution three times. The organic layer was dried by $MgSO_4$, concentrated *in vacuo* to afford a white solid as pure product in 94% yield as a white solid. **m.p.** = 93–95 °C.

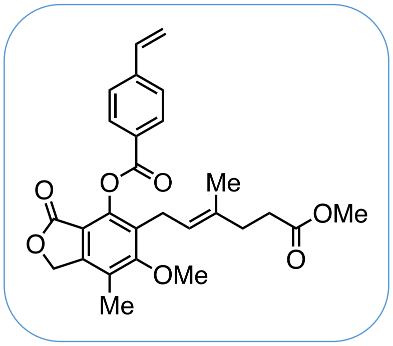

CDI Coupling: Same procedure as previously described.

^{1}H NMR (500 MHz, $CDCl_3$) δ: 8.16 (d, J = 8.4 Hz, 2H), 7.53 (d, J = 8.4 Hz, 2H), 6.79 (dd, J = 17.6, 10.9 Hz, 1H), 5.91 (d, J = 17.6 Hz, 1H), 5.42 (d, J = 10.9 Hz, 1H), 5.17 (s, 2H), 5.16 – 5.12 (m, 1H), 3.81 (s, 3H), 3.60 (s, 3H), 2.31 (dd, J = 9.4, 6.6 Hz, 2H), 2.25 (s, 3H), 2.21 (dd, J = 9.3, 6.5 Hz, 2H), 1.60 (s, 3H).

^{13}C NMR (125 MHz, $CDCl_3$) δ: 173.82, 168.14, 164.37, 162.74, 146.34, 146.31, 142.90, 136.15, 134.74, 130.88, 129.58, 128.04, 126.44, 123.17, 122.33, 117.06, 114.01, 68.42, 61.39, 51.62, 34.52, 32.76, 23.87, 16.35, 11.95.

HRMS (ESI-TOF) *m/z*: [M+H$^+$] calculated for $C_{27}H_{29}O_7$, 465.1913; found, 465.1905.

α-Silyl Amine Synthesis

Unless prepared according to the following procedure, α-silyl amine substrates were used directly from commercial sources.

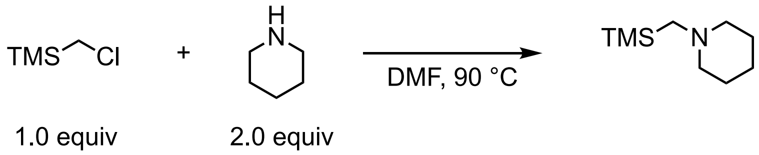

Procedure: A dry 100 mL round-bottom-flask was charged with a stir bar, (chloromethyl)trimethylsilane (20 mmol, 1.0 equiv), piperidine (40 mmol, 2.0 equiv), and 30 mL DMF. The flask was then heated up to 90 °C under N_2 atmosphere overnight. The reaction was quenched by the addition of 30 mL H_2O, and extracted with Et_2O five times. The combined organic layers were washed with H_2O twice and brine once, then dried by $MgSO_4$, concentrated *in vacuo*, and purified by fractional distillation to afford the colorless liquid in 70 % yield.

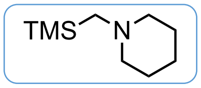

1-((trimethylsilyl)methyl)piperidine: prepared according to previously described procedure.

^{1}H NMR (500 MHz, $CDCl_3$) δ: 2.31 (brs, 4H), 1.88 (s, 2H), 1.55 (p, J = 5.6 Hz, 4H), 1.43 – 1.29 (m, 2H), 0.05 (s, 9H).

^{13}C NMR (125 MHz, $CDCl_3$) δ: 58.61, 51.88, 26.38, 23.95, -0.85.

HRMS (ESI-TOF) *m/z*: [M+H$^+$] calculated for $C_9H_{22}NSi$, 172.1522; found, 172.1519.

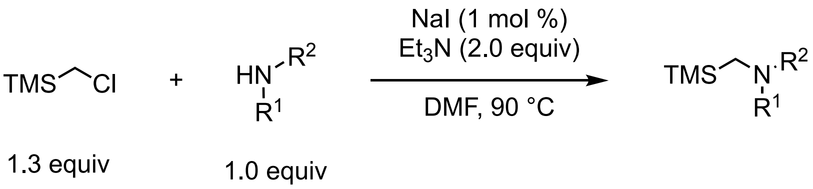

Procedure: A dry 50 mL round-bottom-flask was charged with a stir bar, (chloromethyl)trimethylsilane (13 mmol, 1.3 equiv), amine (10 mmol, 1.0 equiv), NaI(0.1 mmol, 1 mol %), Et_3N (20 mmol, 2.0 equiv) and 10 mL DMF. The flask was then heated up to 90 °C

under N_2 atmosphere overnight. The reaction was quenched by the addition of 30 mL H_2O, and extracted with EtOAc three times. The combined organic layers were washed with H_2O twice and brine once, then dried by $MgSO_4$, concentrated *in vacuo*, and purified by basic alumina chromatography to afford the desired product.

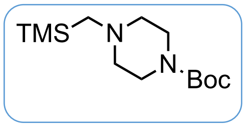

***tert*-butyl 4-((trimethylsilyl)methyl)piperazine-1-carboxylate:** Prepared according to previously described procedure in 84% isolated yield.

Column Chromatography Condition: 300 g Al_2O_3 + 14 g H_2O, 30 : 1 hexanes/EtOAc with 0.5% MeOH to 15 : 1 hexanes/EtOAc with 1.0% MeOH as gradient eluent.

^{1}H NMR (500 MHz, $CDCl_3$) δ: 3.48 – 3.32 (m, 4H), 2.32 (t, J = 5.4 Hz, 4H), 1.90 (s, 2H), 1.45 (s, 9H), 0.05 (s, 9H).

^{13}C NMR (125 MHz, $CDCl_3$) δ: 154.89, 79.51, 56.77, 50.97, 43.90, 28.56, -1.10.

HRMS (ESI-TOF) *m/z*: [M+H$^+$] calculated for $C_{13}H_{29}N_2O_2Si$, 273.1998; found, 273.2006.

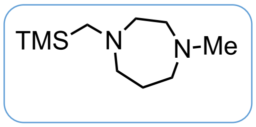

1-methyl-4-((trimethylsilyl)methyl)-1,4-diazepane: Prepared according to previously described procedure, purified by fractional distillation to afford product in 74% yield.

^{1}H NMR (500 MHz, $CDCl_3$) δ: δ 2.71 – 2.65 (m, 4H), 2.64 – 2.54 (m, 4H), 2.34 (s, 3H), 2.06 (s, 2H), 1.77 (dq, J = 7.0, 5.8 Hz, 2H), 0.04 (s, 9H).

^{13}C NMR (125 MHz, $CDCl_3$) δ: 58.84, 58.36, 57.83, 57.12, 51.26, 47.18, 27.40, -1.10.

HRMS (ESI-TOF) *m/z*: [M+H$^+$] calculated for $C_{10}H_{25}N_2Si$, 201.1787; found, 201.1787.

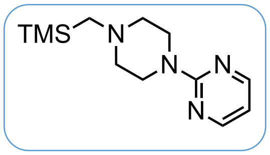

2-(4-((trimethylsilyl)methyl)piperazin-1-yl)pyrimidine: Prepared according to previously described procedure in 71% isolated yield.

Column Chromatography Condition: 200 g Al_2O_3 + 12 g H_2O, 30 : 1 hexanes/EtOAc with 0.5% MeOH as eluent.

^{1}H NMR (500 MHz, $CDCl_3$) δ: 8.29 (d, J = 4.7 Hz, 2H), 6.45 (t, J = 4.7 Hz, 1H), 4.12 – 3.54 (m, 4H), 2.77 – 2.28 (m, 4H), 1.94 (s, 2H), 0.08 (s, 9H).

^{13}C NMR (125 MHz, $CDCl_3$) δ: 161.73, 157.83, 109.76, 56.88, 51.07, 43.97, -1.00.

HRMS (ESI-TOF) *m/z*: [M+H$^+$] calculated for $C_{12}H_{23}N_4Si$, 251.1692; found, 251.1688.

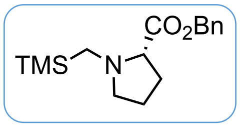

Benzyl ((trimethylsilyl)methyl)-L-prolinate: Prepared according to previously described procedure in 46% isolated yield.

Column Chromatography Condition: silica, 10 : 1 to 6 : 1 hexanes/EtOAc as gradient eluent.

^{1}H NMR (500 MHz, $CDCl_3$) δ: 7.38 – 7.29 (m, 5H), 5.20 (d, J = 12.3 Hz, 1H), 5.11 (d, J = 12.3 Hz, 1H), 3.10 (ddd, J = 7.8, 6.3, 3.7 Hz, 2H), 2.33 (q, J = 8.0 Hz, 1H), 2.31 (d, J = 13.9 Hz, 1H), 2.15 – 2.03 (m, 1H), 1.98 – 1.86 (m, 2H), 1.82 (d, J = 14.0 Hz, 1H) 1.81 – 1.74 (m, 1H), 0.03 (s, 9H).

^{13}C NMR (125 MHz, $CDCl_3$) δ: 174.08, 136.22, 128.63, 128.36, 128.29, 70.16, 66.18, 56.10, 46.00, 29.01, 23.65, -1.29.

HRMS (ESI-TOF) *m/z*: [M+H$^+$] calculated for $C_{16}H_{26}NO_2Si$, 292.1733; found, 292.1726.

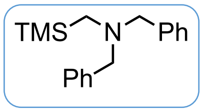

***N,N*-dibenzyl-1-(trimethylsilyl)methanamine:** Prepared according to previously described procedure with 2.0 equiv of (chloromethyl)trimethylsilane in 57% isolated yield.

Column Chromatography Condition: silica, 99 : 1 hexanes/EtOAc as eluent.

^{1}H NMR (500 MHz, $CDCl_3$) δ: 7.42 – 7.36 (m, 4H), 7.31 (dd, J = 8.3, 6.8 Hz, 4H), 7.25 – 7.20 (m, 2H), 3.47 (s, 4H), 1.94 (s, 2H), 0.04 (s, 9H).

^{13}C NMR (125 MHz, $CDCl_3$) δ: 140.45, 128.82, 128.26, 126.83, 62.06, 46.00, -1.10.

HRMS (ESI-TOF) *m/z*: [M+H$^+$] calculated for $C_{18}H_{26}NSi$, 284.1835; found, 284.1824.

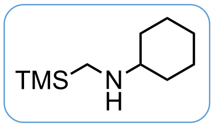

***N*-((trimethylsilyl)methyl)cyclohexanamine:** Prepared according to previously described procedure, purified by fractional distillation to afford product in 60% yield.

^{1}H NMR (500 MHz, $CDCl_3$) δ: 2.26 (tt, J = 10.5, 3.7 Hz, 1H), 2.06 (s, 2H), 1.94 – 1.82 (m, 2H), 1.72 (dt, J = 12.8, 3.6 Hz, 2H), 1.60 (ddt, J = 12.2, 5.0, 2.7 Hz, 1H), 1.33 – 1.08 (m, 3H), 1.08 – 0.96 (m, 2H), 0.57 (brs, 1H), 0.03 (s, 9H).

^{13}C NMR (125 MHz, $CDCl_3$) δ: 60.87, 37.16, 33.29, 26.47, 25.32, -2.36.

HRMS (ESI-TOF) *m/z*: [M+H$^+$] calculated for $C_{10}H_{24}NSi$, 186.1678; found, 186.1678.

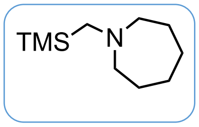

1-((trimethylsilyl)methyl)azepane: Prepared according to previously described procedure, purified by fractional distillation to afford product in 68% yield.

^{1}H NMR (500 MHz, $CDCl_3$) δ: 2.72 – 2.51 (m, 4H), 2.07 (s, 2H), 1.73 – 1.49 (m, 8H), 0.05 (s, 9H).

^{13}C NMR (125 MHz, $CDCl_3$) δ: 59.56, 50.72, 27.80, 27.23, -1.02.

HRMS (ESI-TOF) *m/z*: [M+H$^+$] calculated for $C_{10}H_{24}NSi$, 186.1678; found, 186.1679.

Potassium Alkyl Trifluoroborate Syntheses

Alkyltrifluoroborate salts were prepared from the corresponding alkylboronic acids or alkyl pinacolboronate esters following known literature protocols. Alkyl pinacolboronate esters were prepared from the corresponding alkyl bromides or alkenes following known literature protocols.

From alkylboronic acids:[3]

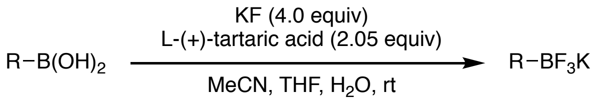

A round-bottomed flask was charged with the alkylboronic acid (1.0 equiv) and acetonitrile (0.25 M). A solution of KF (4.0 equiv) in water (10 M) is added with rapid stirring to produce a clear, homogeneous solution. Next, a solution of L-(+)-tartaric acid (2.05 equiv) in THF (1.37 M) is added dropwise over 2 min, resulting in the formation of a solid precipitate, which settles upon cessation of stirring. After stirring for 5 min at rt, the mixture is filtered over diatomaceous earth to afford a clear solution. The solvent is removed under reduced pressure with a rotary evaporator to afford the product as a white solid. This is further dried under high vacuum to remove trace water. The final product is obtained as a white solid.

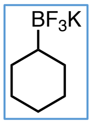

Scale: 10 mmol **Yield:** 1.05 g, 53%

^{1}H NMR (500 MHz, DMSO-d_6) δ: 1.56 (d, J = 9.7 Hz, 3H), 1.47 (d, J = 13.0 Hz, 2H), 1.14 – 0.95 (m, 3H), 0.88 (q, J = 12.1 Hz, 2H), -0.06 (br s, 1H).

^{13}C NMR (126 MHz, Acetone-d_6) δ: 29.39, 28.65, 28.21, 27.64.

^{11}B NMR (128 MHz, DMSO-d_6) δ: 4.80 (m).

^{19}F NMR (470 MHz, DMSO-d_6) δ: -144.63 (m).

The spectroscopic data matched that which was previously reported in the literature.[4]

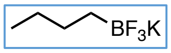

Scale: 20 mmol **Yield:** 2.50 g, 76%

^{1}H NMR (500 MHz, DMSO-d_6) δ: 1.21 – 1.14 (m, 2H), 1.11 – 1.05 (m, 2H), 0.78 (t, J = 7.2 Hz, 3H), -0.08 (q, J = 7.3 Hz, 2H).

^{13}C NMR (126 MHz, DMSO-d_6) δ: 28.27, 26.18, 14.58. (C–B *ipso* carbon not visible)

^{11}B NMR (161 MHz, DMSO-d_6) δ: 9.60 (q, J = 64.4 Hz).

^{19}F NMR (471 MHz, DMSO-d_6) δ: -132.02 (m).

The spectroscopic data matched that which was previously reported in the literature.[5]

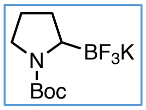

Scale: 10 mmol **Yield:** 1.96 g, 71%

^{1}H NMR (400 MHz, Acetone-d_6) δ: 3.25 (m, 1H), 3.14 (m, 1H), 2.87 (m, 1H), 1.83 (m, 2H), 1.61 (m, 2H), 1.39 (s, 9H). (mixture of rotamers)

^{13}C NMR (126 MHz, Acetone-d_6) δ: 155.54, 77.90, 51.15, 47.72, 29.07, 28.54, 25.90. (mixture of rotamers)

^{11}B NMR (128 MHz, Acetone-d_6) δ: 3.44 (m).

^{19}F NMR (376 MHz, Acetone-d_6) δ: -146.39 (m).

From alkyl bromides:[6]

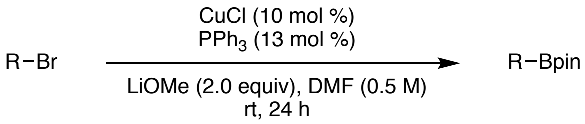

In the glovebox, an oven-dried flask equipped with a stir bar is charged with the alkyl bromide (1.0 equiv), CuI (10 mol %), triphenylphosphine (13 mol %), lithium methoxide (2.0 equiv), B_2pin_2 (1.5 equiv), and DMF (0.5 M). The flask was sealed, removed from the glovebox, and rapidly stirred at 37 °C for 24 h. Next, the thick black mixture is filtered over a pad of diatomaceous earth to afford a clear colored filtrate. This is added to a separatory funnel, rinsing with ethyl acetate, and the mixture is washed with water (3x20 mL) and brine (1x10 mL) to remove DMF and salts. The organic layer is dried with anhydrous sodium sulfate, filtered, and the solvent is removed under reduced pressure with the aid of a rotary evaporator. The crude residue is further purified *via* flash chromatography on silica gel, eluting with mixtures of ethyl acetate and hexane, to afford pure products.

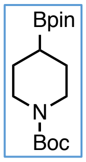

Scale: 16.5 mmol **Yield:** 3.99 g, 83%

^{1}H NMR (500 MHz, $CDCl_3$) δ: 3.78 (m, 2H), 2.92 (m, 2H), 1.63 (m, 2H), 1.49 (m, 2H), 1.44 (s, 9H), 1.23 (s, 12H), 1.10 (m, 1H).

^{13}C NMR (126 MHz, $CDCl_3$) δ: 154.89, 83.14, 79.02, 44.87, 28.50, 26.97, 24.76.

^{11}B NMR (161 MHz, $CDCl_3$) δ: 33.74 (m).

The spectroscopic data matched that which was previously reported in the literature.[6]

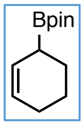

Scale: 25 mmol **Yield:** 3.17 g, 61%

^{1}H NMR (500 MHz, $CDCl_3$) δ: 5.72 (m, 1H), 5.67 (m, 1H), 1.99 (m, 2H), 1.76 (m, 2H), 1.66 (m, 2H), 1.59 (m, 1H), 1.24 (s, 12H).

^{13}C NMR (126 MHz, $CDCl_3$) δ: 127.86, 126.33, 83.40, 25.26, 25.07, 24.94, 24.38, 22.80.

^{11}B NMR (128 MHz, $CDCl_3$) δ: 33.36 (m).

The spectroscopic data matched that which was previously reported in the literature.[6]

From styrene:[7]

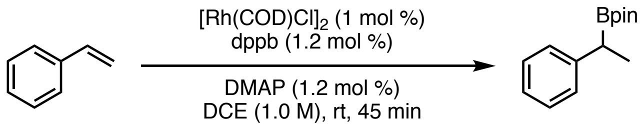

In the glovebox, an oven-dried round-bottomed flask equipped with a stir bar is charged with $[Rh(COD)Cl]_2$ (49.3 mg, 1 mol % Rh), 1,4-bis(diphenylphosphino)butane (102 mg, 1.2 mol %), 4-dimethylamino pyridine (29.3 mg, 1.2 mol %), and 1,2-dichloroethane (20 mL, 1 M). Styrene (2.3 mL, 1.0 equiv, 20 mmol) and pinacolborane (3.5 mL, 1.2 equiv, 24 mmol) are next added and the flask is sealed,

removed from the glovebox, and allowed to stir at rt from 45 min after which point is was deemed to be complete by GCMS analysis. The solvents are removed under reduced pressure with the aid of a rotary evaporator, and the crude product is directly loaded onto a silica gel column and purified, eluting with 5% ethyl acetate in hexanes. The pure product is obtained as a clear oil.

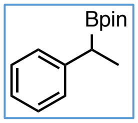

Scale: 20 mmol **Yield:** 4.51 g, 97%

^{1}H NMR (500 MHz, $CDCl_3$) δ: 7.26 (m, 2H), 7.22 (m, 2H), 7.14 (m, 1H), 2.43 (q, *J* = 7.5 Hz, 1H), 1.33 (d, *J* = 7.5 Hz, 3H), 1.21 (s, 12H).

^{13}C NMR (126 MHz, $CDCl_3$) δ: 145.25, 128.56, 128.05, 125.34, 83.55, 24.90, 24.86, 17.32.

^{11}B NMR (161 MHz, $CDCl_3$) δ: 33.38 (m).

The spectroscopic data matched that which was previously reported in the literature.[8]

Conversion of pinacolboronate esters into the corresponding trifluoroborate salts:

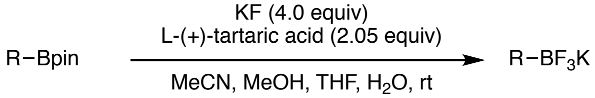

Procedure:[3] To convert the alkyl pinacolboronate ester into the corresponding trifluoroborate salt, it is taken up in a 1:1 mixture of MeCN:MeOH (0.25 M). A solution of KF (4.0 equiv) in water (10 M) is added and the mixture is stirred until homogeneous. Next, a solution of L-(+)-tartaric acid (2.05 equiv) in THF (1.37 M) is added dropwise over 5 min. The mixture is allowed to stir at rt for 2 min after which point it is diluted with additional MeCN (3 mL/mmol substrate). Following stirring at rt for a further 2 min, the mixture is filtered over a pad of diatomaceous earth, rinsing with MeCN. The solvents are removed under reduced pressure with the aid of a rotary evaporator to afford a crude mixture of the product and pinacol as a white solid. The pinacol is removed under high vacuum at 80 °C to provide the pure product. When this reaction is conducted on a large scale (≥10 mmol), an initial recrystallization of the product from acetone and ether aids to remove most of the pinacol, the rest of which could be removed under high vacuum at 80 °C.

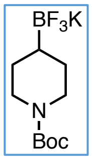

Scale: 12.8 mmol **Yield:** 1.79 g, 48%

^{1}H NMR (400 MHz, Acetone-d_6) δ: 4.06 – 3.88 (m, 2H), 2.64 – 2.38 (m, 2H), 1.55 – 1.42 (m, 2H), 1.39 (s, 9H), 1.24 – 1.08 (m, 2H), 0.33 – 0.19 (m, 1H).

^{13}C NMR (126 MHz, Acetone-d_6) δ: 155.17, 78.28, 46.78, 28.89, 28.68.

^{11}B NMR (128 MHz, Acetone-d_6) δ: 4.90 (m).

^{19}F NMR (376 MHz, Acetone-d_6) δ: -149.02 (m).

The spectroscopic data matched that which was previously reported in the literature.[9]

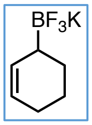

Scale: 15.2 mmol **Yield:** 2.19 g, 77%

^{1}H NMR (400 MHz, Acetone-d_6) δ: 5.80 (dd, *J* = 10.1, 2.6 Hz, 1H), 5.29 (dq, *J* = 10.2, 3.5 Hz, 1H), 1.88 – 1.79 (m, 2H), 1.76 – 1.66 (m, 1H), 1.59 (m, 1H), 1.51 – 1.40 (m, 1H), 1.40 – 1.28 (m, 1H), 1.05 (br, 1H).

^{13}C NMR (126 MHz, Acetone-d_6) δ: 136.05, 121.84, 26.44, 25.74, 25.73, 24.13.

^{11}B NMR (128 MHz, Acetone-d_6) δ: 4.67 (q, *J* = 63.0 Hz).

^{19}F NMR (376 MHz, Acetone-d_6) δ: -146.62 (dd, J = 118.4, 52.9 Hz).

The spectroscopic data matched that which was previously reported in the literature.[10]

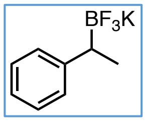

Scale: 6.9 mmol

Yield: 1.79 g, 84%

^{1}H NMR (500 MHz, Acetone-d_6) δ 7.17 (d, J = 7.6 Hz, 2H), 7.08 (t, J = 7.7 Hz, 2H), 6.90 (t, J = 7.2 Hz, 1H), 1.84 – 1.73 (m, 1H), 1.16 (d, J = 7.4 Hz, 3H).

^{13}C NMR (126 MHz, Acetone-d_6) δ: 153.22, 128.57, 127.71, 123.23, 25.52, 17.38.

^{11}B NMR (161 MHz, Acetone-d_6) δ: 4.65 (q, J = 62.4 Hz).

^{19}F NMR (471 MHz, Acetone-d_6) δ: -146.45.

The spectroscopic data matched that which was previously reported in the literature.[11]

All other carbon components were commercially available.

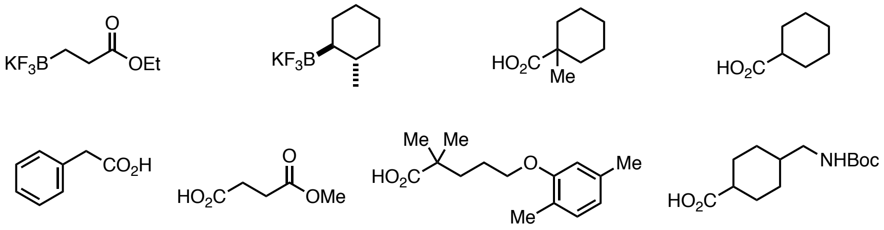

C. Reaction Set-up for Photoredox Catalysis

The photo-reactor consists of an ABI 24W blue LED grow light (purchased from Amazon), a clip fan, a Fisher stir plate, contained in a housing made out of a plastic sharp container (8 gallons, 30 × 25 × 35 cm). Three 20 × 20 cm windows were cut out at front, left, and right sides of the sharp container using a razor blade or scissors. As shown in the pictures, the modified housing is put upside down on the Fisher stir plate with a window facing front for loading samples. The blue LED light and clip fan are stuck into the container through the side windows, and supported by the clamps. A 10 × 10 cm platform is made out of cardboard, wrapped with aluminum foil, and covered with transparent tape to help holding the reaction vials. The front window is cover with a foldable cardboard.

The photoreactions are set-up in 4 mL vials (Chemglass, CG-4904-05) and placed on the platform, about 5-8 cm away from the LED light and 15-20 cm from the clip fan. The temperature is measured by a thermometer placed inside the housing.

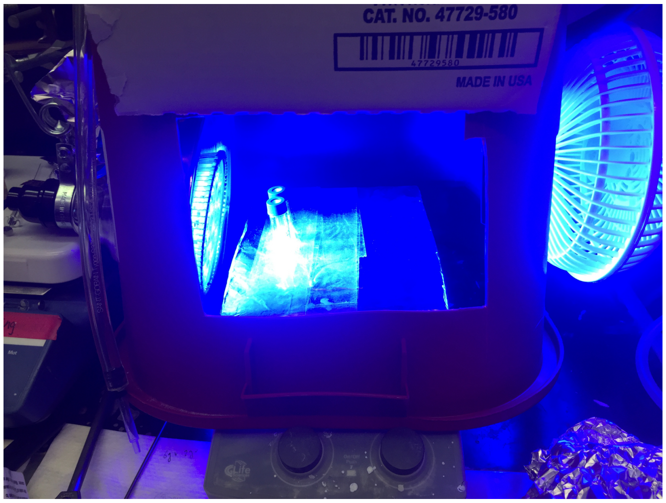

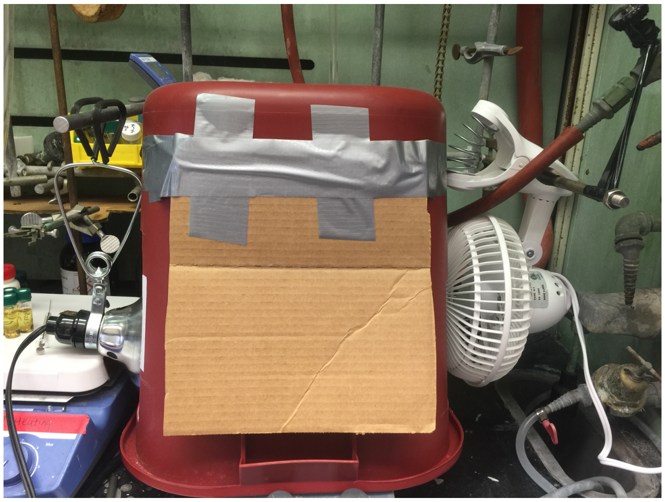

Supplementary Figure 1. Reaction Set-up

D. Selected Optimization Results

Supplementary Table 1. Survey of hydrogen-sources with Bn–BF_3K[a]

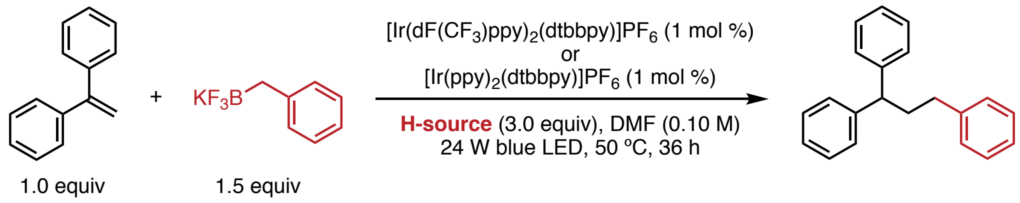

Entry	Photocatalyst	hydrogen-source	GC Yield (%)
1	$[Ir(dF(CF_3)ppy)_2(dtbbpy)]PF_6$	^{i}PrOH	15
2	$[Ir(dF(CF_3)ppy)_2(dtbbpy)]PF_6$	MeOH	12
3	$[Ir(dF(CF_3)ppy)_2(dtbbpy)]PF_6$	HFIP	15
4	$[Ir(dF(CF_3)ppy)_2(dtbbpy)]PF_6$	AcOH	15
5	$[Ir(dF(CF_3)ppy)_2(dtbbpy)]PF_6$	trifluoroethanol	11
6	$[Ir(ppy)_2(dtbbpy)]PF_6$	^{i}PrOH	3
7	$[Ir(ppy)_2(dtbbpy)]PF_6$	MeOH	10
8	$[Ir(ppy)_2(dtbbpy)]PF_6$	HFIP	2
9	$[Ir(ppy)_2(dtbbpy)]PF_6$	AcOH	2
10	$[Ir(ppy)_2(dtbbpy)]PF_6$	trifluoroethanol	5

[a] **Conditions:** In the glovebox, an oven-dried 4-mL reaction vial equipped with a stir bar was charged with the photocatalyst (1 mol %), $BnBF_3K$ (29.7 mg, 0.15 mmol, 1.5 equiv), DMF (1.0 mL, 0.10 M), and 1,1-diphenylethylene (17.8 μL, 0.10 mmol, 1.0 equiv). The vial was then sealed, removed from the glovebox, and through the septum was added the hydrogen-source (3.0 equiv) *via* syringe. The reaction was then stirred for 36 h under irradiation by a 24 W blue LED lamp. The apparatus was surrounded by foil to minimize escape of incident light, which allowed the temperature to rise to 50 °C. The reaction was analyzed by GC with 1-methylnaphthalene (10 μL) as internal standard.

Supplementary Table 2. Survey of 1,1-diphenylethylene and Bn–BF_3K equivalencies[a]

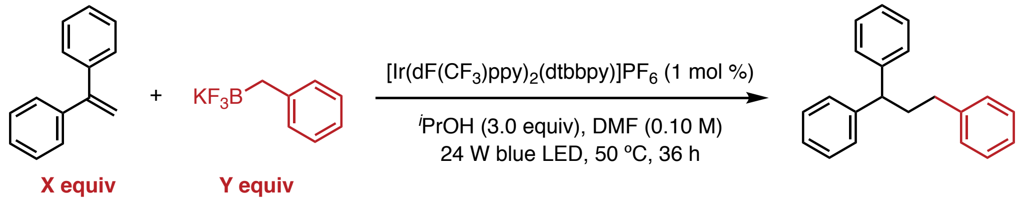

Entry	1,1-diphenylethylene equivalents	$BnBF_3K$ equivalents	GC Yield (%)
1	1.0	1.0	37
2	1.0	1.5	15
3	1.0	2.0	21
4	1.0	3.0	22
5	1.0	5.0	20
6	1.5	1.0	68
7	2.0	1.0	83
8	3.0	1.0	97
9	5.0	1.0	94

[a.] **Conditions:** In the glovebox, an oven-dried 4-mL reaction vial equipped with a stir bar was charged with $[Ir(dF(CF_3)ppy)_2(dtbbpy)]PF_6$ (1.2 mg, 1 mol %), $BnBF_3K$ (1.0-5.0 equiv), DMF (1.0 mL, 0.10 M), 1,1-diphenylethylene (1.0-5.0 equiv), and ^{i}PrOH (23 μL, 0.30 mmol, 3.0 equiv). The vial was then sealed, removed from the glovebox, and stirred for 36 h under irradiation by a 24 W blue LED lamp. The apparatus was surrounded by foil to minimize escape of incident light, which allowed the temperature to rise to 50 °C. The reaction was analyzed by GC with 1-methylnaphthalene (10 μL) as internal standard.

Supplementary Table 3. Survey of solvents[a]

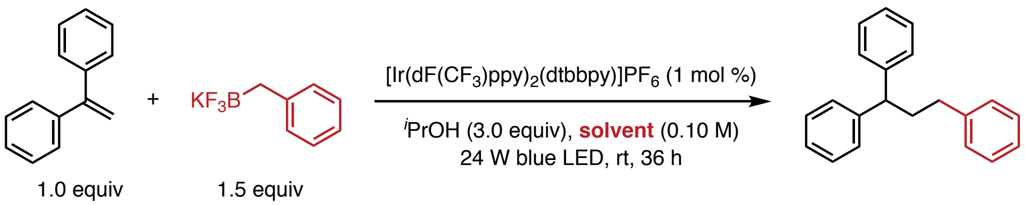

Entry	Solvent	GC Yield (%)
1	DMF	15
2	DMSO	15
3	DMPU	6
4	THF	10
5	1,2-dichloroethane	11
6	PhMe	7
7	MeCN	15
8[b]	DMF	11

[a.] **Conditions:** In the glovebox, an oven-dried 4-mL reaction vial equipped with a stir bar was charged with $[Ir(dF(CF_3)ppy)_2(dtbbpy)]PF_6$ (1.2 mg, 1 mol %), $BnBF_3K$ (29.7 mg, 0.15 mmol, 1.5 equiv), solvent (1.0 mL, 0.10 M), 1,1-diphenylethylene (17.8 µL, 0.10 mmol, 1.0 equiv), and ^{i}PrOH (23 µL, 0.30 mmol, 3.0 equiv). The vial was then sealed, removed from the glovebox, and stirred for 36 h under irradiation by a 24 W blue LED lamp. The apparatus was surrounded by foil to minimize escape of incident light, which allowed the temperature to rise to 50 °C. The reaction was analyzed by GC with 1-methylnaphthalene (10 µL) as internal standard. [b.] reaction was conducted at 0.50 M in DMF.

Supplementary Table 4. Survey of ^{i}PrOH equivalencies[a]

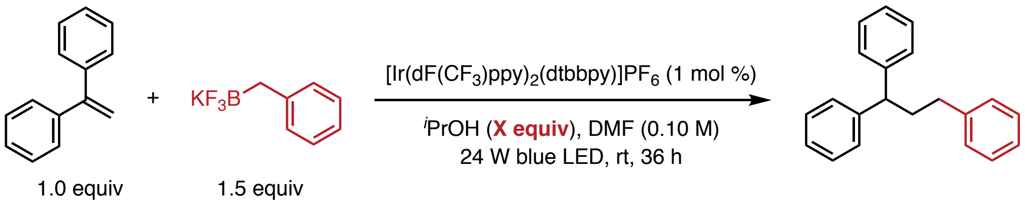

**Entry**	**^{i}PrOH equivalencies**	**GC Yield (%)**
1	1.0	14
2	3.0	15
3	5.0	12
4	10	12

[a.] **Conditions:** In the glovebox, an oven-dried 4-mL reaction vial equipped with a stir bar was charged with [Ir(dF(CF$_3$)ppy)$_2$(dtbbpy)]PF$_6$ (1.2 mg, 1 mol %), BnBF$_3$K (29.7 mg, 0.15 mmol, 1.5 equiv), solvent (1.0 mL, 0.10 M), 1,1-diphenylethylene (17.8 µL, 0.10 mmol, 1.0 equiv), and ^{i}PrOH (23 µL, 0.30 mmol, 3.0 equiv). The vial was then sealed, removed from the glovebox, and stirred for 36 h under irradiation by a 24 W blue LED lamp. The apparatus was surrounded by foil to minimize escape of incident light, which allowed the temperature to rise to 50 °C. The reaction was analyzed by GC with 1-methylnaphthalene (10 µL) as internal standard. [b.] reaction was conducted at 0.50 M in DMF.

Supplementary Table 5. Survey of additional hydrogen-sources[a]

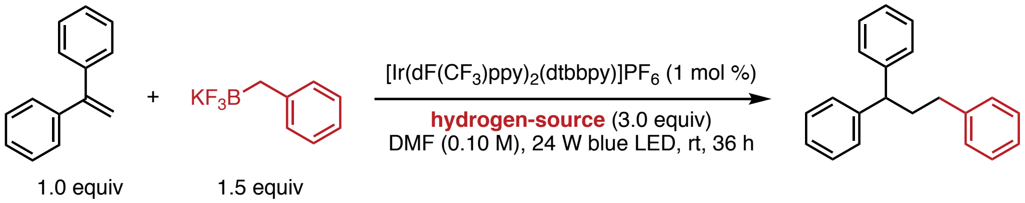

Entry	Hydrogen-source	GC Yield (%)
1	H–Si(Me)(OEt)$_2$	0
2	PhNMe$_2$	0
3	1,4-cyclohexadiene	14
4	PhSH	0
5	PhOH	83
6	4-MeO-PhOH	>99
7	guaiacol	85
8	4-Me-PhOH	>99
9	8-hydroxyquinoline	40
10	4-hydroxypyridine	65
11	4-Cl-PhOH	71
12	4-F-PhOH	60
13	3,5-(MeO)$_2$-PhOH	62
14	4-CHO-PhOH	7
15	pentafluorophenol	34
16	4-CF$_3$-PhOH	52
17	ethylparaben	65
18	4-NO$_2$-PhOH	11
19	BHT	8
20	4-^{t}Butyl-catechol	60

[a]. **Conditions:** In the glovebox, an oven-dried 4-mL reaction vial equipped with a stir bar was charged with [Ir(dF(CF$_3$)ppy)$_2$(dtbbpy)]PF$_6$ (1.2 mg, 1 mol %), BnBF$_3$K (29.7 mg, 0.15 mmol, 1.5 equiv), DMF (1.0 mL, 0.10 M), 1,1-diphenylethylene (17.8 μL, 0.10 mmol, 1.0 equiv), and the H-source (0.30 mmol, 3.0 equiv). The vial was then sealed, removed from

the glovebox, and stirred for 36 h at rt under irradiation by a 24 W blue LED lamp. The reaction was analyzed by GC with 1-methylnaphthalene (10 μL) as internal standard.

Supplementary Table 6. Survey of guaiacol equivalencies[a]

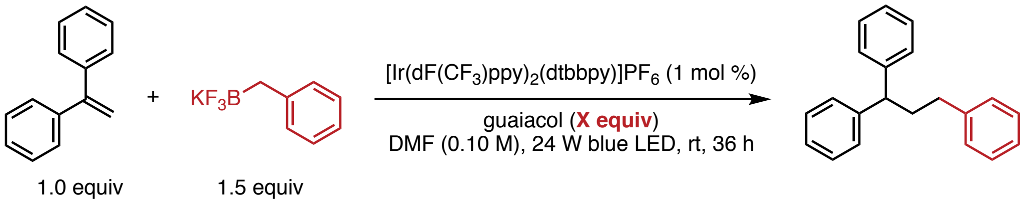

Entry	guaiacol equivalencies	GC Yield (%)
1	1.0	45
2	1.5	78
3	3.0	85
4	5.0	54

[a] **Conditions:** In the glovebox, an oven-dried 4-mL reaction vial equipped with a stir bar was charged with $[Ir(dF(CF_3)ppy)_2(dtbbpy)]PF_6$ (1.2 mg, 1 mol %), $BnBF_3K$ (29.7 mg, 0.15 mmol, 1.5 equiv), DMF (1.0 mL, 0.10 M), 1,1-diphenylethylene (17.8 μL, 0.10 mmol, 1.0 equiv), and guaiacol (1.0-5.0 equiv). The vial was then sealed, removed from the glovebox, and stirred for 36 h at rt under irradiation by a 24 W blue LED lamp. The reaction was analyzed by GC with 1-methylnaphthalene (10 μL) as internal standard.

Supplementary Table 7. Time studies[a]

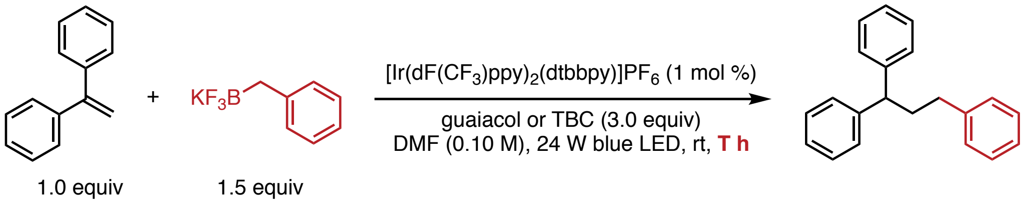

Entry	H-source	Time (h)	GC Yield (%)
1	4-*t*Butyl-catechol	0.5	3
2	4-*t*Butyl-catechol	1	4
3	4-*t*Butyl-catechol	3.6	8
4	4-*t*Butyl-catechol	4	10
5	4-*t*Butyl-catechol	15	20
6	4-*t*Butyl-catechol	36	41
7	4-*t*Butyl-catechol	48	60
8	guaiacol	0.5	3
9	guaiacol	1	6
10	guaiacol	3.6	17
11	guaiacol	4	22
12	guaiacol	15	43
13	guaiacol	36	59
14	guaiacol	48	85

[a.] **Conditions:** In the glovebox, an oven-dried 4-mL reaction vial equipped with a stir bar was charged with $[Ir(dF(CF_3)ppy)_2(dtbbpy)]PF_6$ (1.2 mg, 1 mol %), $BnBF_3K$ (29.7 mg, 0.15 mmol, 1.5 equiv), DMF (1.0 mL, 0.10 M), 1,1-diphenylethylene (17.8 µL, 0.10 mmol, 1.0 equiv), and either guaiacol (33.6 µL, 0.30 mmol, 3.0 equiv) or 4-*t*Butyl-catechol (49.9 mg, 0.30 mmol, 3.0 equiv). The vial was then sealed, removed from the glovebox, and stirred at rt under irradiation by a 24 W blue LED lamp. The reaction was analyzed by GC with 1-methylnaphthalene (10 µL) as internal standard.

Supplementary Table 8. Survey of hydrogen-sources with 2° organoboron derivatives[a]

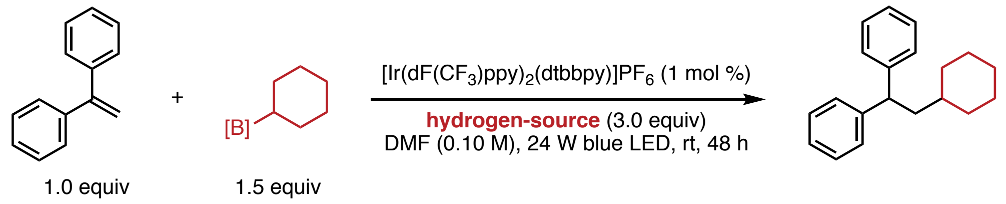

Entry	[B]	Hydrogen-source	GC Yield (%)
1	BF_3K	PhOH	93
2	BF_3K	4-MeO-PhOH	90
3	BF_3K	guaiacol	80
4	BF_3K	4-Me-PhOH	89
5	BF_3K	4-hydroxypyridine	32
6	BF_3K	4-Br-PhOH	67
7	BF_3K	4-CF_3-PhOH	4
8	BF_3K	ethylparaben	28
9	BF_3K	BHT	4
10	$B[(OCH_2)_3CMe]K$	PhOH	60
11	$B[(OCH_2)_3CMe]K$	4-MeO-PhOH	49
12	$B[(OCH_2)_3CMe]K$	guaiacol	54
13	$B[(OCH_2)_3CMe]K$	4-Me-PhOH	44
14	$B[(OCH_2)_3CMe]K$	4-hydroxypyridine	97
15	$B[(OCH_2)_3CMe]K$	4-Br-PhOH	19
16	$B[(OCH_2)_3CMe]K$	4-CF_3-PhOH	39
17	$B[(OCH_2)_3CMe]K$	ethylparaben	6
18	$B[(OCH_2)_3CMe]K$	BHT	30

[a] **Conditions:** In the glovebox, an oven-dried 4-mL reaction vial equipped with a stir bar was charged with $[Ir(dF(CF_3)ppy)_2(dtbbpy)]PF_6$ (1.2 mg, 1 mol %), Cy-[B] (0.15 mmol, 1.5 equiv), DMF (1.0 mL, 0.10 M), 1,1-diphenylethylene

(17.8 μL, 0.10 mmol, 1.0 equiv), and the H-source (0.30 mmol, 3.0 equiv). The vial was then sealed, removed from the glovebox, and stirred for 48 h at rt under irradiation by a 24 W blue LED lamp. The reaction was analyzed by GC with 1-methylnaphthalene (10 μL) as internal standard.

Supplementary Table 9. Survey of hydrogen-sources with a 1° organoboron derivative[a]

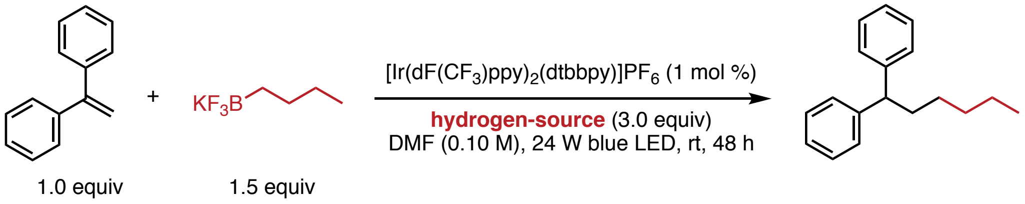

Entry	Hydrogen-source	GC Yield (%)
1	PhOH	31
2	4-MeO-PhOH	1
3	guaiacol	1
4	4-Me-PhOH	4
5	4-hydroxypyridine	6
6	4-Br-PhOH	17
7	ethylparaben	5
8	2-Ph-PhOH	2
9	2-CN-PhOH	2
10	2,4,6-Me_3-PhOH	0
11	^{i}PrOH	3

[a.] **Conditions:** In the glovebox, an oven-dried 4-mL reaction vial equipped with a stir bar was charged with [Ir(dF(CF_3)ppy)$_2$(dtbbpy)]PF_6 (1.2 mg, 1 mol %), $BuBF_3K$ (24.6 mg, 0.15 mmol, 1.5 equiv), DMF (1.0 mL, 0.10 M), 1,1-diphenylethylene (17.8 μL, 0.10 mmol, 1.0 equiv), and the H-source (0.30 mmol, 3.0 equiv). The vial was then sealed, removed from the glovebox, and stirred for 48 h at rt under irradiation by a 24 W blue LED lamp. The reaction was analyzed by GC with 1-methylnaphthalene (10 μL) as internal standard.

Supplementary Table 10. Survey of hydrogen-sources with a 2º benzylic organoboron derivative[a]

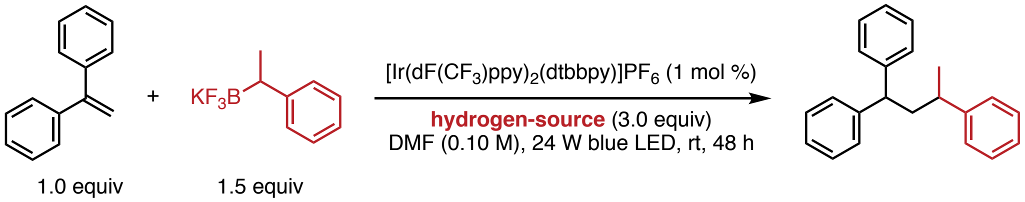

Entry	Hydrogen-source	Equivalents	A(pdt)/A(IS)
1	PhOH	1.5	1.72
2	4-MeO-PhOH	1.5	1.22
3	4-Me-PhOH	1.5	1.52
4	2-Ph-PhOH	1.5	1.81
5	guaiacol	1.5	1.95
6	PhOH	3.0	2.00
7	4-MeO-PhOH	3.0	1.75
8	4-Me-PhOH	3.0	1.95
9	2-Ph-PhOH	3.0	2.01
10	guaiacol	3.0	1.92

[a.] **Conditions:** In the glovebox, an oven-dried 4-mL reaction vial equipped with a stir bar was charged with $[Ir(dF(CF_3)ppy)_2(dtbbpy)]PF_6$ (1.2 mg, 1 mol %), α-Me-Bn-BF_3K (31.8 mg, 0.15 mmol, 1.5 equiv), DMF (1.0 mL, 0.10 M), 1,1-diphenylethylene (17.8 μL, 0.10 mmol, 1.0 equiv), and the H-source (0.30 mmol, 3.0 equiv). The vial was then sealed, removed from the glovebox, and stirred for 48 h at rt under irradiation by a 24 W blue LED lamp. The reaction was analyzed by GC with 1-methylnaphthalene (10 μL) as internal standard.

Supplementary Table 11. Survey of alkene and organoboron equivalencies with a 1° organoboron derivative[a]

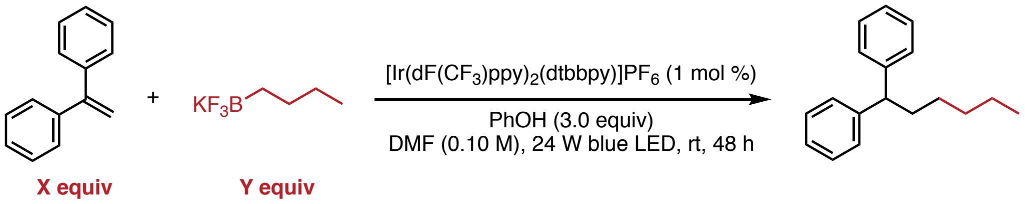

Entry	1,1-diphenylethylene equivalents	$BuBF_3K$ equivalents	GC Yield (%)
1	1.0	1.5	39
2	1.0	2.0	8
3	1.0	3.0	26
4	1.0	4.0	15
5	1.0	5.0	4
6	1.5	1.0	31
7	2.0	1.0	5
8	3.0	1.0	3
9	4.0	1.0	3
10	5.0	1.0	3

[a.] **Conditions:** In the glovebox, an oven-dried 4-mL reaction vial equipped with a stir bar was charged with $[Ir(dF(CF_3)ppy)_2(dtbbpy)]PF_6$ (1.2 mg, 1 mol %), $BuBF_3K$ (1.0-5.0 equiv), DMF (1.0 mL, 0.10 M), 1,1-diphenylethylene (1.0-5.0 equiv), and PhOH (28.2 mg, 0.30 mmol, 3.0 equiv). The vial was then sealed, removed from the glovebox, and stirred for 48 h at rt under irradiation by a 24 W blue LED lamp. The reaction was analyzed by GC with 1-methylnaphthalene (10 μL) as internal standard.

Supplementary Table 12. Survey of hydrogen-source equivalencies with a 1° organoboron derivative[a]

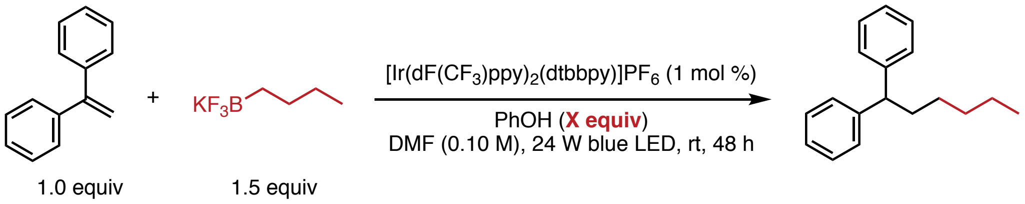

Entry	PhOH equivalents	GC Yield (%)
1	1.0	15
2	1.5	13
3	2.0	6
4	3.0	40
5	4.0	11
6	5.0	7

[a.] **Conditions:** In the glovebox, an oven-dried 4-mL reaction vial equipped with a stir bar was charged with [Ir(dF(CF_3)ppy)$_2$(dtbbpy)]PF_6 (1.2 mg, 1 mol %), $BuBF_3K$ (24.6 mg, 0.15 mmol, 1.5 equiv), DMF (1.0 mL, 0.10 M), 1,1-diphenylethylene (17.8 μL, 0.10 mmol, 1.0 equiv), and PhOH (1.0-5.0 equiv). The vial was then sealed, removed from the glovebox, and stirred for 48 h at rt under irradiation by a 24 W blue LED lamp. The reaction was analyzed by GC with 1-methylnaphthalene (10 μL) as internal standard.

Supplementary Table 13. Survey of hydrogen-sources with *trans*-2-methylcyclohexyl potassium trifluoroborate

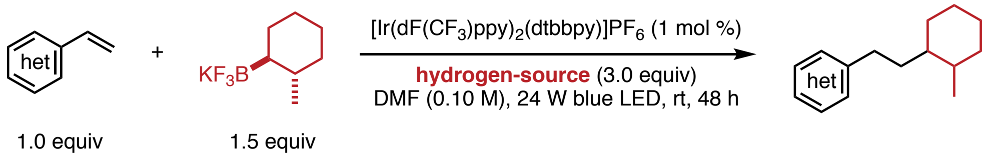

Entry	Alkene	Hydrogen-source	A(pdt)/A(IS)	d.r.
1	4-vinylpyridine	PhOH	1.36	4.3
2	4-vinylpyridine	4-MeO-PhOH	0.45	4.2
3	4-vinylpyridine	guaiacol	0.61	4.2
4	4-vinylpyridine	4-Me-PhOH	1.41	4.2
5	4-vinylpyridine	4-hydroxpyridine	0.47	4.3
6	4-vinylpyridine	4-Br-PhOH	0.90	4.3
7	4-vinylpyridine	ethylparaben	1.11	4.3
8	4-vinylpyridine	2-Ph-PhOH	1.63	4.2
9	4-vinylpyridine	2-CN-PhOH	0.36	4.0
10	4-vinylpyridine	2,4,6-Me_3-PhOH	0.05	4.0
11	4-vinylpyridine	^{i}PrOH	0.33	4.1
12	2-vinylpyridine	PhOH	0.64	4.1
13	2-vinylpyridine	4-MeO-PhOH	1.03	4.1
14	2-vinylpyridine	guaiacol	1.28	4.1
15	2-vinylpyridine	4-Me-PhOH	0.39	4.1
16	2-vinylpyridine	4-hydroxpyridine	0.99	4.4

17	2-vinylpyridine	4-Br-PhOH	1.25	4.1

[a] **Conditions:** In the glovebox, an oven-dried 4-mL reaction vial equipped with a stir bar was charged with $[Ir(dF(CF_3)ppy)_2(dtbbpy)]PF_6$ (1.2 mg, 1 mol %), *trans*-2-methylcyclohexyl potassium trifluoroborate (30.6 mg, 0.15 mmol, 1.5 equiv), DMF (1.0 mL, 0.10 M), the alkene (0.10 mmol, 1.0 equiv), and hydrogen-source (0.30 mmol, 3.0 equiv). The vial was then sealed, removed from the glovebox, and stirred for 48 h at rt under irradiation by a 24 W blue LED lamp. The reaction was analyzed by GC with 1-methylnaphthalene (10 μL) as internal standard.

Supplementary Table 14. Survey of bases with alkyl carboxylic acid substrates[a]

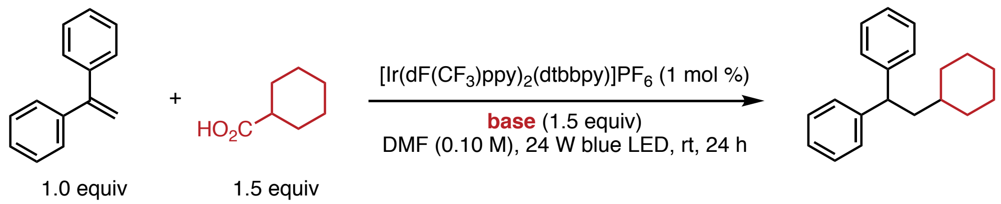

Entry	Base	GC Yield (%)
1	Cs_2CO_3	56
2	K_2CO_3	45
3	Na_2CO_3	42
4	K_2HPO_4	66
5	KH_2PO_4	72
6	K_3PO_4	93

[a] **Conditions:** In the glovebox, an oven-dried 4-mL reaction vial equipped with a stir bar was charged with $[Ir(dF(CF_3)ppy)_2(dtbbpy)]PF_6$ (1.2 mg, 1 mol %), cyclohexane carboxylic acid (19.2 mg, 0.15 mmol, 1.5 equiv), DMF (1.0 mL, 0.10 M), 1,1-diphenylethylene (17.8 µL, 0.10 mmol, 1.0 equiv), and base (0.15 mmol, 1.5 equiv). The vial was then sealed, removed from the glovebox, and stirred for 24 h at rt under irradiation by a 24 W blue LED lamp. The reaction was analyzed by GC with 1-methylnaphthalene (10 µL) as internal standard.

Supplementary Table 15. Survey of conditions with a 3° acid and styrene[a]

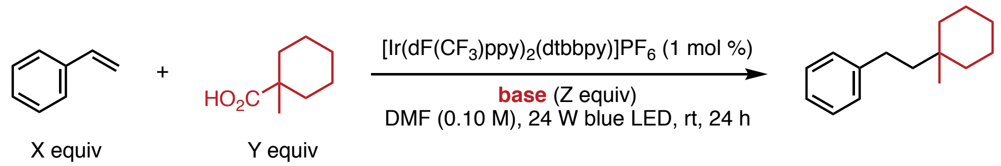

Entry	Base	Styrene equivalents	Acid equivalents	GC Yield (%)
1	Cs_2CO_3	1.0	1.5	13
2	K_2CO_3	1.0	1.5	21
3	Na_2CO_3	1.0	1.5	8
4	K_3PO_4	1.0	1.5	19
5	K_3PO_4	1.0	2.0	12
6	K_3PO_4	1.0	3.0	14
7	K_3PO_4	1.0	5.0	41
8	K_3PO_4	1.5	1.0	37
9	K_3PO_4	3.0	1.0	22
10	K_3PO_4	5.0	1.0	10
11[b]	K_3PO_4	3.0	1.0	62
12[b]	K_3PO_4	1.0	3.0	88
13[c]	K_3PO_4	1.0	1.5	25
14[d]	K_3PO_4	1.0	1.5	23
15[e]	K_3PO_4	1.0	1.5	7

[a.] **Conditions:** In the glovebox, an oven-dried 4-mL reaction vial equipped with a stir bar was charged with $[Ir(dF(CF_3)ppy)_2(dtbbpy)]PF_6$ (1.2 mg, 1 mol %), 1-methyl-cyclohexane-1-carboxylic acid (1.0-5.0 equiv), DMF (0.5 mL, 0.20 M), styrene (1.0-5.0 equiv), and base (0.15 mmol, 1.5 equiv). The vial was then sealed, removed from the glovebox, and stirred for 24 h at rt under irradiation by a 24 W blue LED lamp. The reaction was analyzed by GC with 1-methylnaphthalene (10 μL) as internal standard. [b.] reaction conducted for 48 h. [c.] with 2.0 equiv K_3PO_4 [d.] with 3.0 equiv K_3PO_4 [e.] with 5.0 equiv K_3PO_4.

Supplementary Table 16. Survey of conditions with a 1° acid[a]

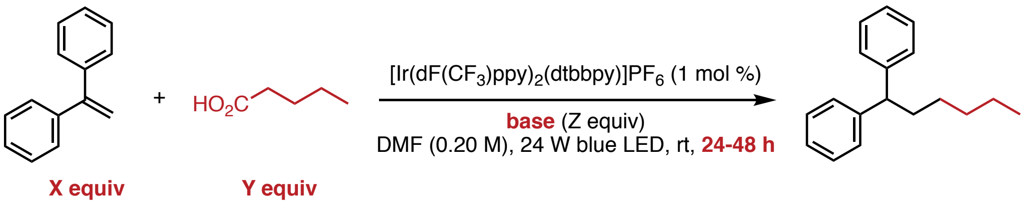

Entry	Base(s)	1,1-diphenylethylene equivalents	Acid equivalents	T (h)	GC Yield (%)
1	Cs_2CO_3	1.0	1.5	48	12
2	K_2CO_3	1.0	1.5	48	11
3	Na_2CO_3	1.0	1.5	48	8
4	$(Bu_4N)_2CO_3$	1.0	1.5	48	20
5	K_3PO_4	1.0	1.5	48	28
6[b]	K_3PO_4	1.0	2.0	48	11
7[c]	K_3PO_4	1.0	3.0	48	18
8[d]	K_3PO_4	1.0	5.0	48	10
9	K_3PO_4	1.5	1.0	48	15
10	K_3PO_4	3.0	1.0	48	12
11	K_3PO_4	5.0	1.0	48	7
12	K_3PO_4	1.5	1.0	24	7
13	K_3PO_4	3.0	1.0	24	6
14[c]	K_3PO_4	1.0	3.0	24	7
15	$(Bu_4N)_2CO_3$ (0.2 equiv) + K_2CO_3 (1.0 equiv)	1.0	1.5	48	30
16	$(Bu_4N)_2CO_3$ (0.2 equiv) + K_3PO_4 (1.0 equiv)	1.0	1.5	48	23
17	$(Bu_4N)_2CO_3$ (1.0 equiv) + K_3PO_4 (2.0 equiv)	1.0	3.0	48	27
18	$(Bu_4N)_2CO_3$ (1.0 equiv) + K_2CO_3 (2.0 equiv)	1.0	3.0	48	34

[a.] **Conditions:** In the glovebox, an oven-dried 4-mL reaction vial equipped with a stir bar was charged with $[Ir(dF(CF_3)ppy)_2(dtbbpy)]PF_6$ (1.2 mg, 1 mol %), 1-methyl-cyclohexane-1-carboxylic acid (1.0-5.0 equiv), DMF (0.5 mL, 0.20 M), styrene (1.0-5.0 equiv), and base (0.15 mmol, 1.5 equiv). The vial was then sealed, removed from the glovebox, and stirred for 24 h at rt under irradiation by a 24 W blue LED lamp. The reaction was analyzed by GC with 1-methylnaphthalene (10 μL) as internal standard. [b.] with 2.0 equiv K_3PO_4 [c.] with 3.0 equiv K_3PO_4 [d.] with 5.0 equiv K_3PO_4.

Supplementary Table 17. Survey of photocatalysts and proton sources for hydroaminomethylation.[a]

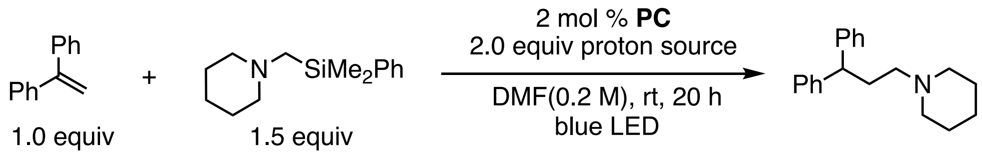

Entry	Photocatalyst	Proton Source H^+	Yield [b]
1	**PC 4**	H_2O	22
2	**PC 4**	MeOH	46
3	**PC 4**	IPA	40
4	**PC 4**	HOAc	45
5	**PC 1**	MeOH	32
6	**PC 1**	HOAc	12
7	**PC 2**	MeOH	8
8	**PC 2**	HOAc	11
9	**PC 3**	MeOH	**75**
10	**PC 3**	HOAc	61

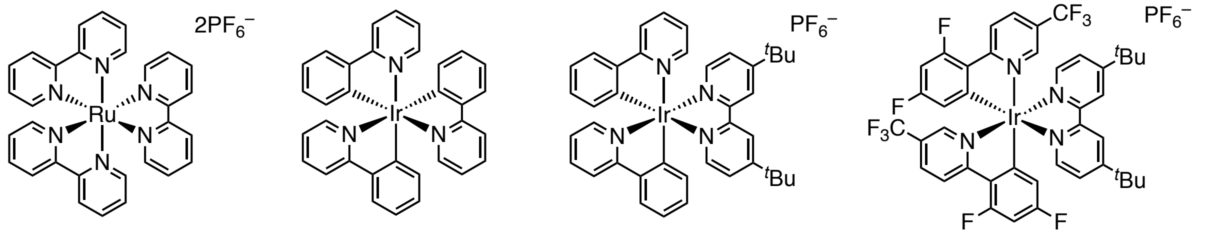

PC 1: $[Ru(bpy)_3](PF_6)_2$ [c]
Ru^{II*}/Ru^{I} = + 0.77 V
Ru^{I}/Ru^{II} = - 1.33 V

PC 2: *fac*-$Ir(ppy)_3$ [c]
Ir^{III*}/Ir^{II} = + 0.31 V
Ir^{II}/Ir^{III} = - 2.19 V

PC 3: $[Ir(ppy)_2(dtbbpy)]PF_6$ [c]
Ir^{III*}/Ir^{II} = + 0.66 V
Ir^{II}/Ir^{III} = - 1.51 V

PC 4: $[Ir(dF(CF_3)ppy)_2(dtbbpy)]PF_6$ [c]
Ir^{III*}/Ir^{II} = + 1.21 V
Ir^{II}/Ir^{III} = - 1.37 V

[a] Standard conditions are alkene (0.1 mmol, 1.0 equiv), amine (1.5 equiv), photocatalyst (2.0 mol %), proton source (2.0 equiv), DMF (0.5 mL, 0.2 M), rt, blue LED light. [b] In situ yield determined by GC analysis. [c] All potentials are given versus the saturated calomel electrode (SCE),

Supplementary Table 18. Survey of photocatalysts under optimized conditions for hydroaminomethylation.[a]

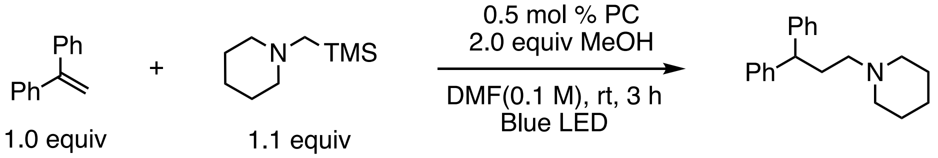

Entry	Photocatalyst	Yield [b]
1	**PC 1**	6
2	**PC 2**	39
3	**PC 3**	90
4	**PC 4**	92
5	none	0
6[c]	**PC 3**	0

[a] Standard conditions are alkene (0.1 mmol, 1.0 equiv), amine (1.1 equiv), photocatalyst (0.5 mol %), proton source (2.0 equiv), DMF (1.0 mL, 0.1 M), rt, blue LED light. [b] In situ yield determined by GC analysis. [c] Reaction run in dark.

Supplementary Table 19. Miscellaneous substrate combinations.

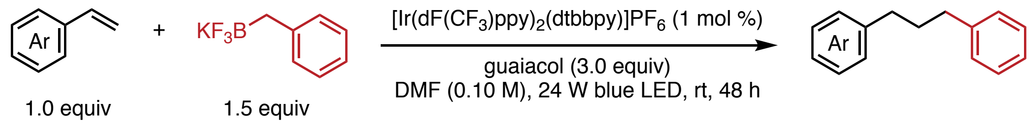

[a] **Conditions:** In the glovebox, an oven-dried 4-mL reaction vial equipped with a stir bar was charged with [Ir(dF(CF_3)ppy)$_2$(dtbbpy)]PF_6 (1.2 mg, 1 mol %), $BnBF_3K$ (29.7 mg, 0.15 mmol, 1.5 equiv), DMF (1.0 mL, 0.10 M), the alkene (0.10 mmol, 1.0 equiv), and guaiacol (33.6 µL, 0.30 mmol, 3.0 equiv). The vial was then sealed, removed from the glovebox, and stirred at rt under irradiation by a 24 W blue LED lamp. The reaction was analyzed by GC with 1-methylnaphthalene (10 µL) as internal standard.

A(pdt)/A(IS) values are provided.

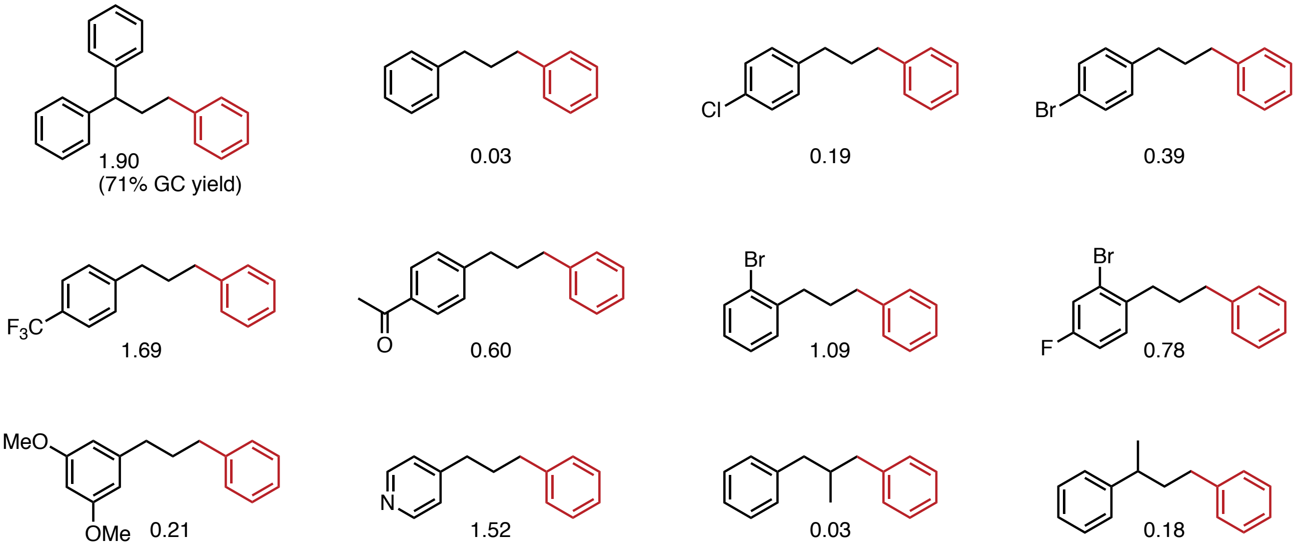

For hydroaminoalkylation:

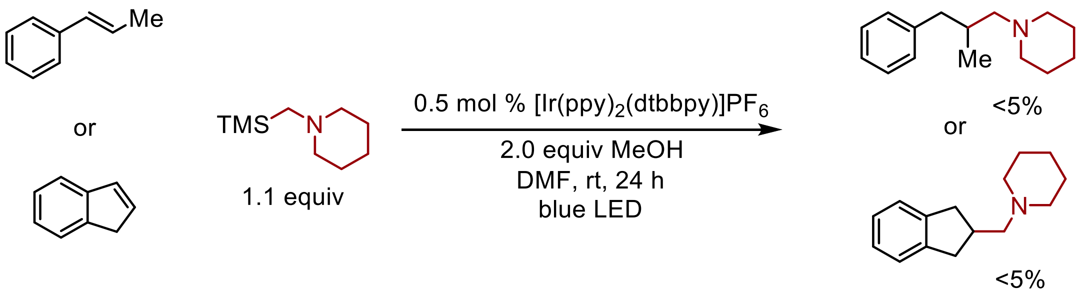

E. Mechanistic Studies

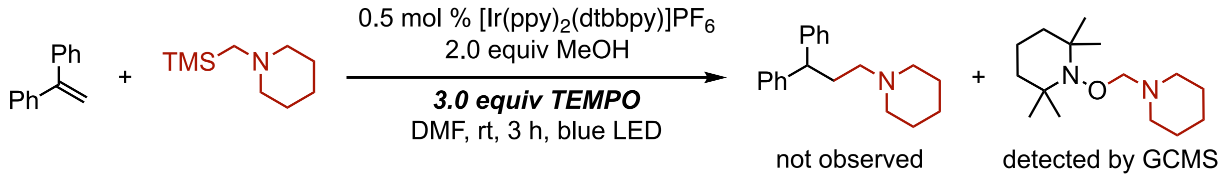

Procedure: [Ir(ppy)$_2$(dtbbpy)]PF$_6$ (**PC 3**, 0.45 mg, 0.0005 mmol, 0.5 mol %), alkene (0.1 mmol, 1.0 equiv), α-TMS amine (0.11 mmol, 1.1 equiv), TEMPO (0.3 mmol, 3.0 equiv) and DMF (1.0 mL) were added to a 4 mL vial equipped with a stir bar in the glove box under nitrogen atmosphere. The vial was then taken out of glove box and injected 8 uL MeOH (0.2 mmol, 2.0 equiv). The resulting solution was allowed to stir inside the photo-reactor (described above) with the lamp and fan on for 3 hours. The reaction crude was quenched by the addition of DCM, and then analyzed by GC and GC-MS upon the addition of the internal standard.

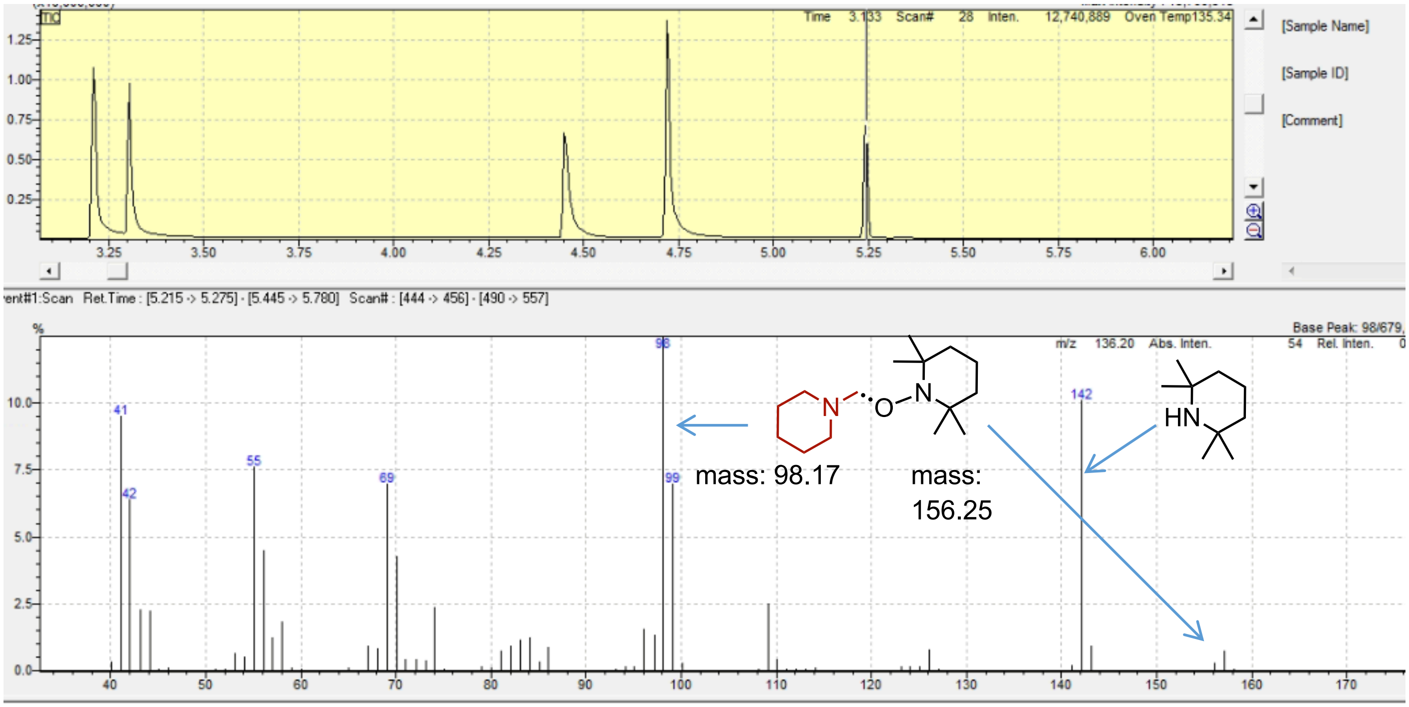

Supplementary Figure 2. GC-MS trace of the TEMPO quenching experiment

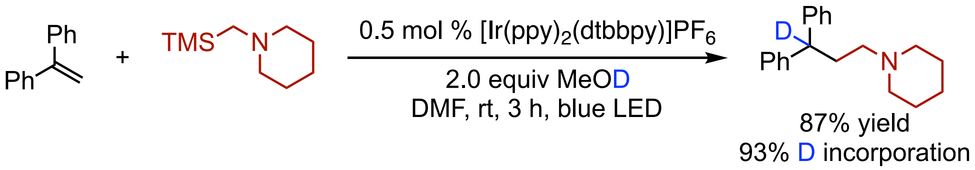

Procedure: [Ir(ppy)$_2$(dtbbpy)]PF_6 (**PC 3**, 0.9 mg, 0.001 mmol, 0.5 mol %), alkene (0.2 mmol, 1.0 equiv), α-TMS amine (0.22 mmol, 1.1 equiv), and DMF (2.0 mL) were added to a 4 mL vial equipped with a stir bar in the glove box under nitrogen atmosphere. The vial was then taken out of glove box and injected 16 uL MeOD (0.4 mmol, 2.0 equiv). The resulting solution was allowed to stir inside the photo-reactor (described above) with the lamp and fan on for 3 hours. The reaction crude was quenched by the addition of DCM, concentrated *in vacuo* and then purified by basic alumina chromatography to afford the desired product in 87% isolated yield, and 93% deuterium incorporation.

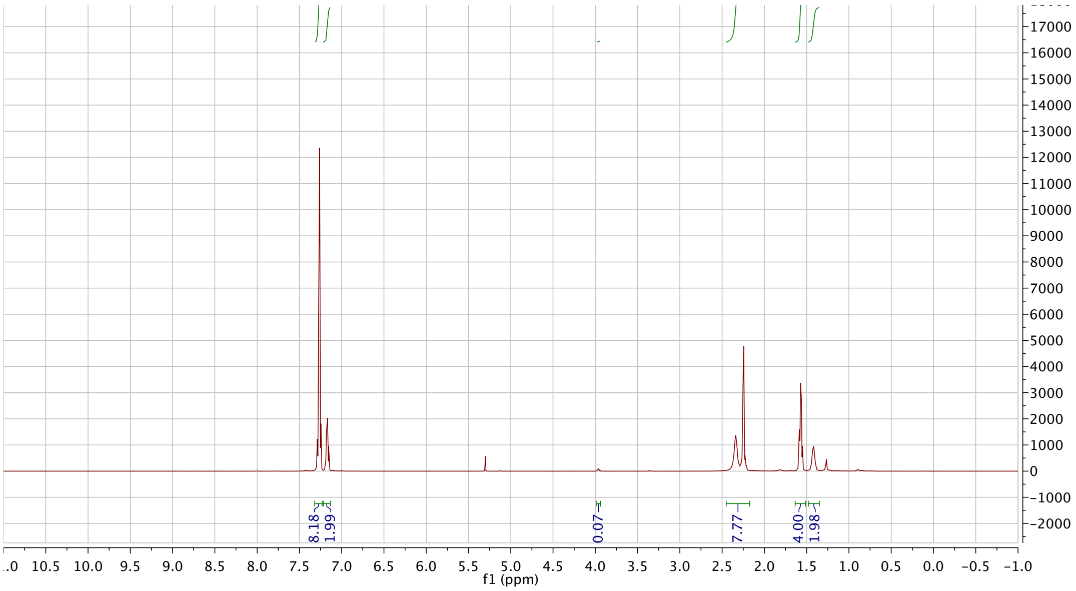

Supplementary Figure 3. ^{1}H NMR of the deuterated product

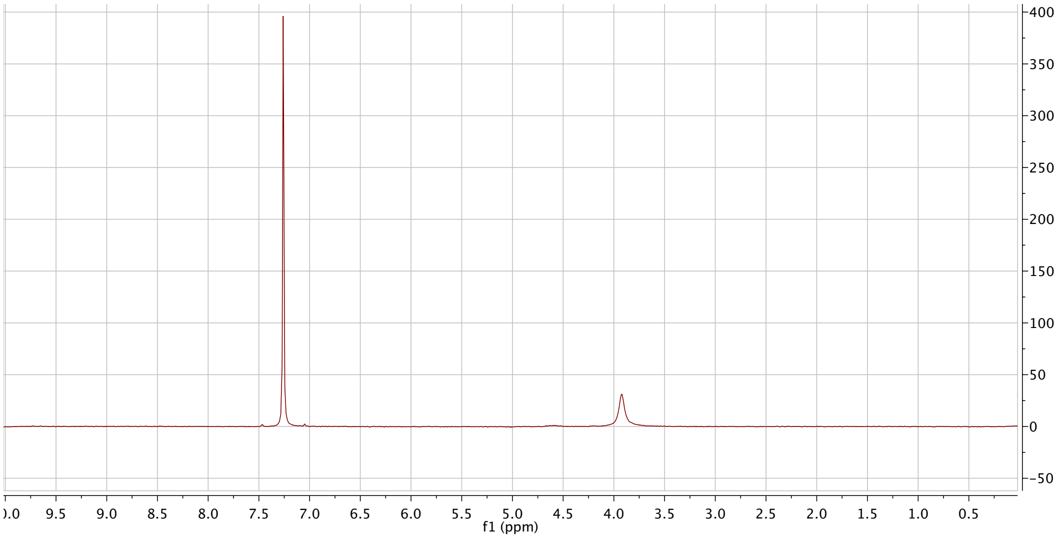

Supplementary Figure 4: ^{2}H NMR of the deuterated product

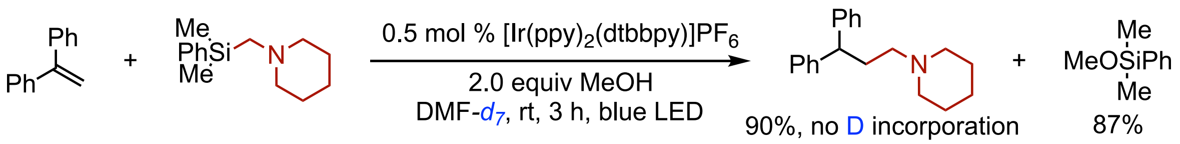

Procedure: $[Ir(ppy)_2(dtbbpy)]PF_6$ (**PC 3**, 0.9 mg, 0.001 mmol, 0.5 mol %), alkene (0.2 mmol, 1.0 equiv), α-TMS amine (0.22 mmol, 1.1 equiv), and DMF-d_7 (2.0 mL) were added to a 4 mL vial equipped with a stir bar in the glove box under nitrogen atmosphere. The vial was then taken out of glove box and injected 16 uL MeOH (0.4 mmol, 2.0 equiv). The resulting solution was allowed to stir inside the photo-reactor (described above) with the lamp and fan on for 3 hours. The reaction crude was directly analyzed by NMR spectroscopy in DMF-d_7 upon the addition of the internal standard. The product was obtained in 90% GC yield, and no deuterium incorporation.

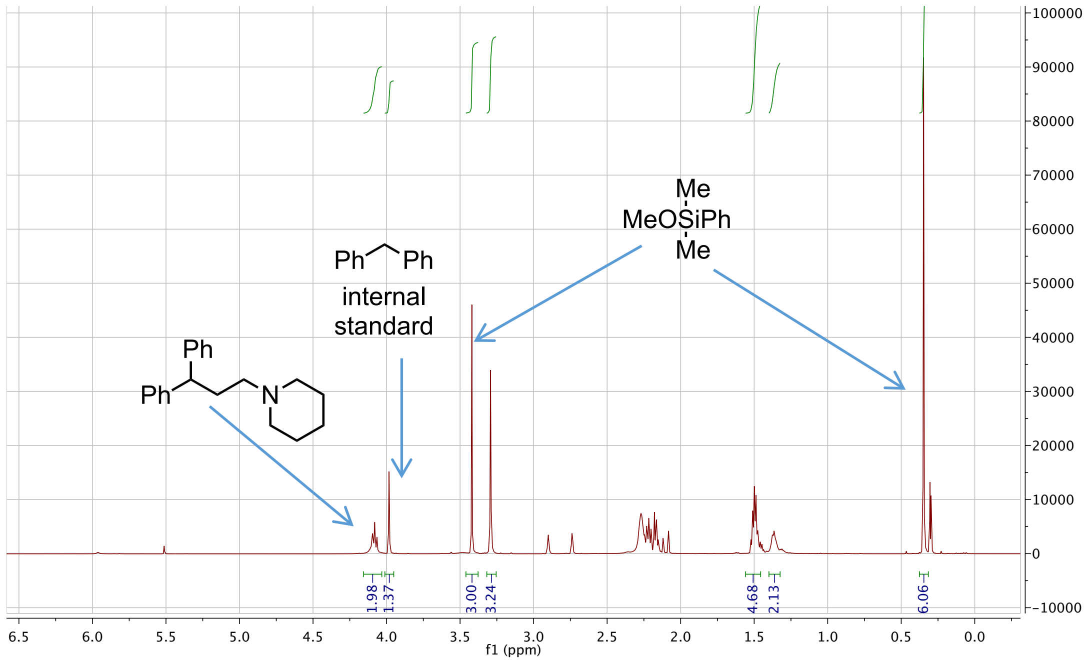

Supplementary Figure 5: ^{1}H NMR of the reaction crude in DMF-d7

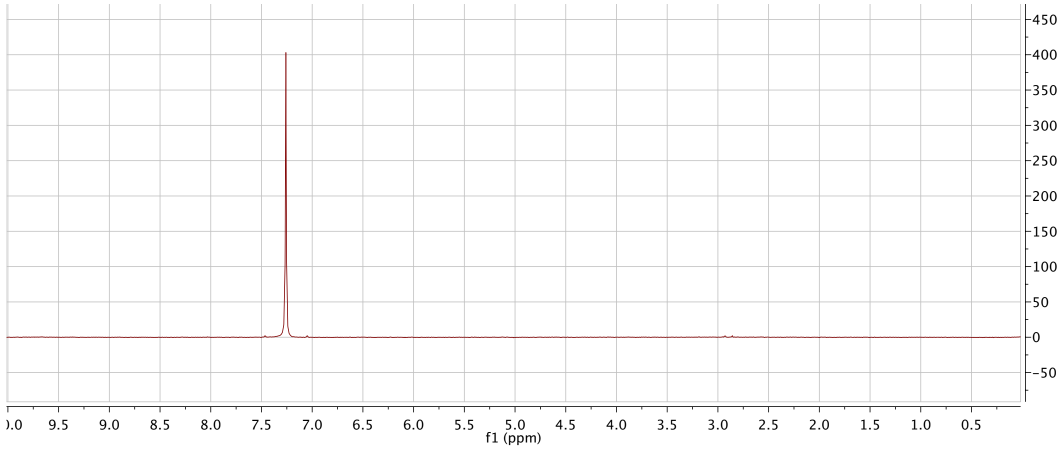

Supplementary Figure 6: ^{2}H NMR of the reaction crude in DMF-d7

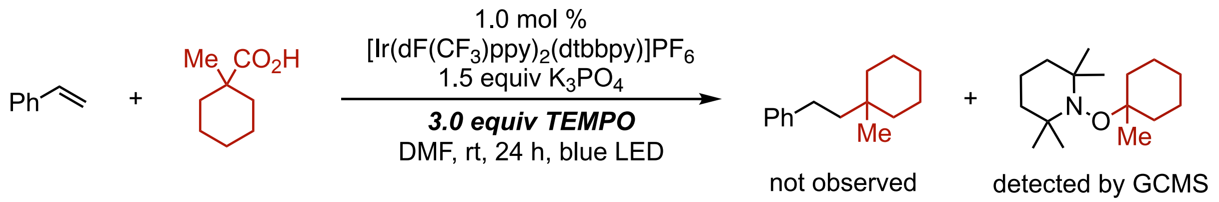

Procedure: In a nitrogen-filled glove box, an oven-dried 4-mL reaction vial equipped with a stir bar was charged with $[Ir(dF(CF_3)ppy)_2(dtbbpy)]PF_6$ (1.2 mg, 1 μmol, 1 mol %) and K_3PO_4 (31.8 mg, 0.15 mmol, 1.5 equiv). This was followed by addition of carboxylic acid (0.15 mmol, 1.5 equiv), 0.5 mL DMF, and styrene (11.5 μL, 0.10 mmol, 1.0 equiv). The vial was sealed with a teflon-lined cap, removed from the glove box, and irradiated with two 24 W blue LEDs with stirring at 800 rpm at rt for 24 h. The reaction mixture was diluted with ethyl acetate (2.5 mL) and the internal standard 1-methylnaphthalene (10 μL) was added. This mixture was then analyzed by GC and GC-MS.

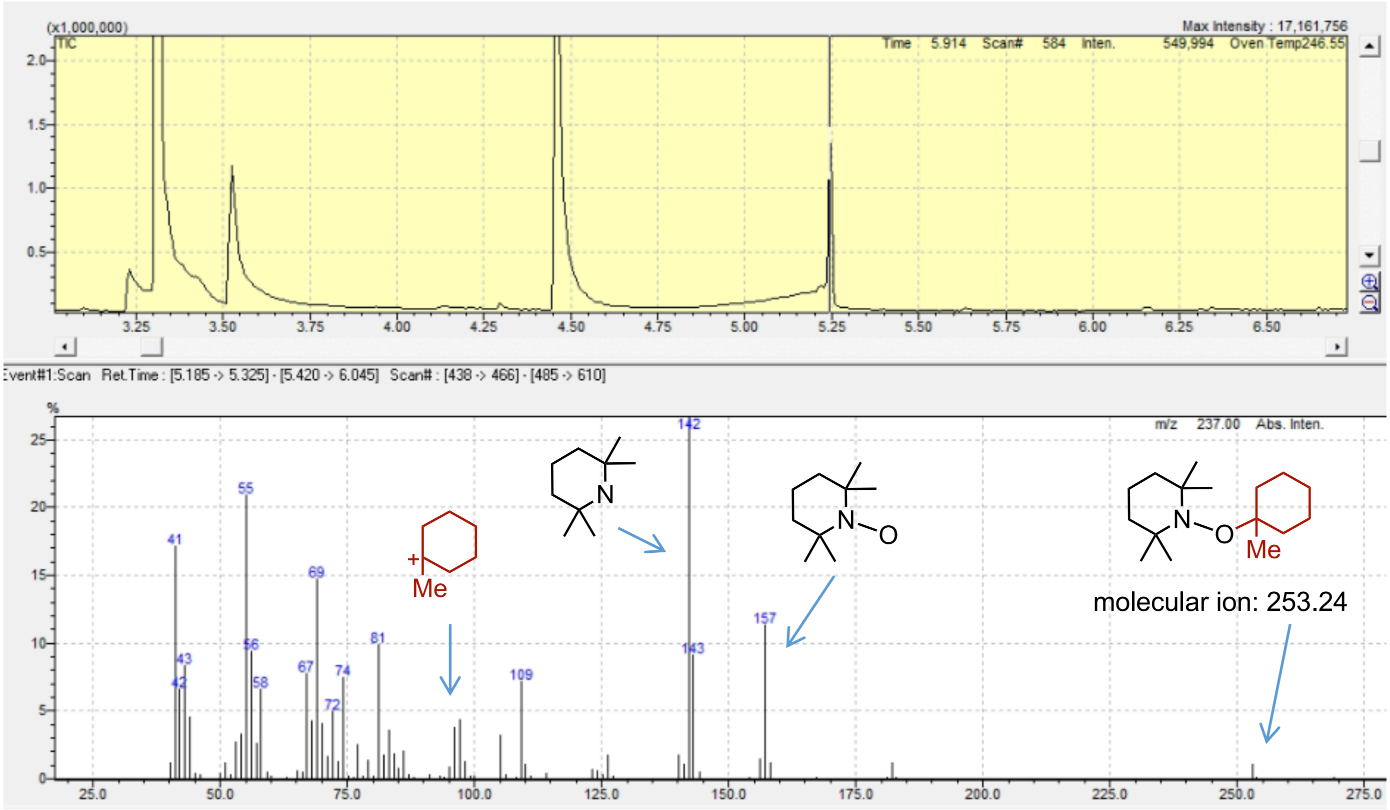

Supplementary Figure 7. GC-MS trace of the TEMPO quenching experiment

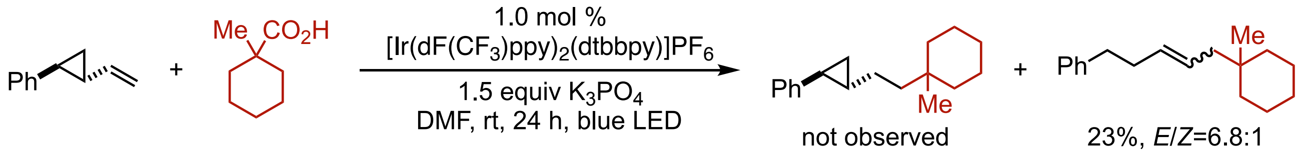

Procedure: In a nitrogen-filled glove box, an oven-dried 4-mL reaction vial equipped with a stir bar was charged with $[Ir(dF(CF_3)ppy)_2(dtbbpy)]PF_6$ (1.2 mg, 1 μmol, 1 mol %) and K_3PO_4 (31.8 mg, 0.15 mmol, 1.5 equiv). This was followed by addition of carboxylic acid (0.15 mmol, 1.5 equiv), 0.5 mL DMF, and alkene (0.10 mmol, 1.0 equiv). The vial was sealed with a teflon-lined cap, removed from the glove box, and irradiated with two 24 W blue LEDs with stirring at 800 rpm at rt for 24 h. The reaction mixture was diluted with ethyl acetate (2.5 mL) and the internal standard 1-methylnaphthalene (10 μL) was added. The mixture was then washed with water (3x2 mL) to remove most of the DMF. The organic layer was sampled and the solvent removed under vacuum. This mixture was then analyzed by GC-MS and ^{1}H NMR spectroscopy.

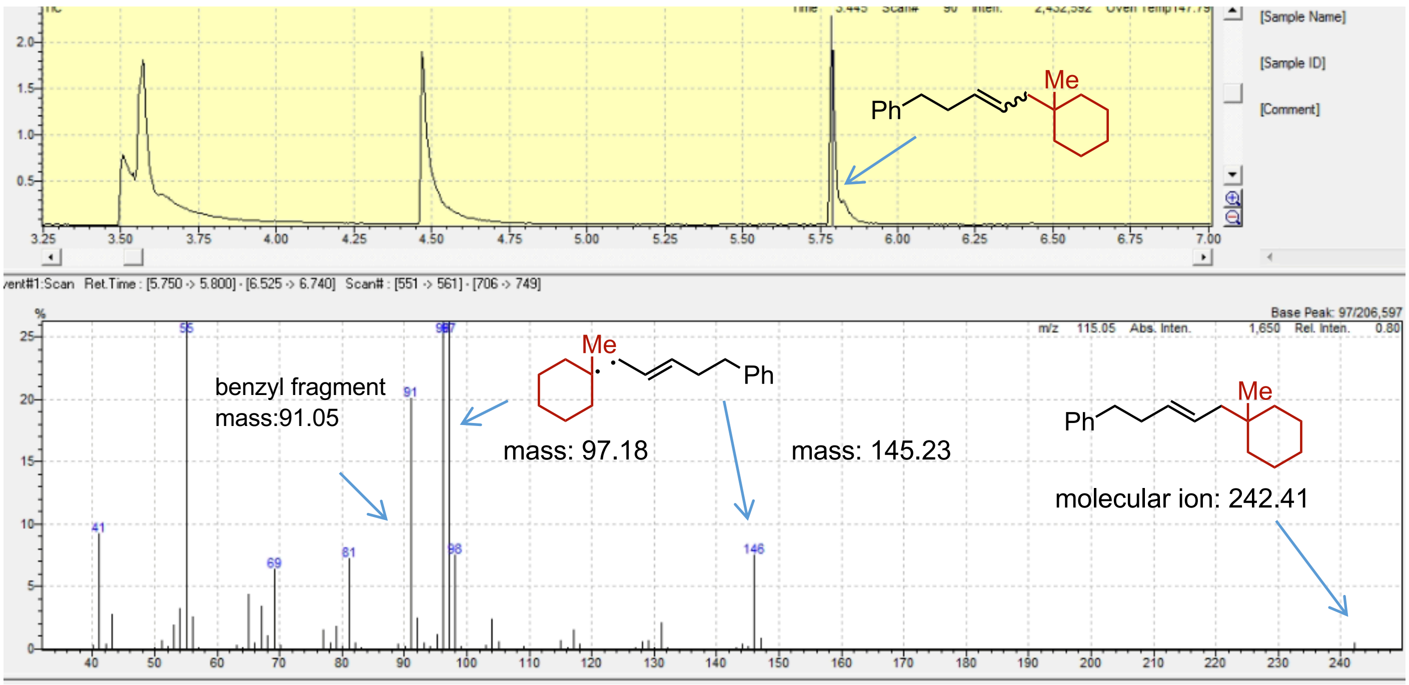

Supplementary Figure 8. GC-MS trace of the radical clock experiment

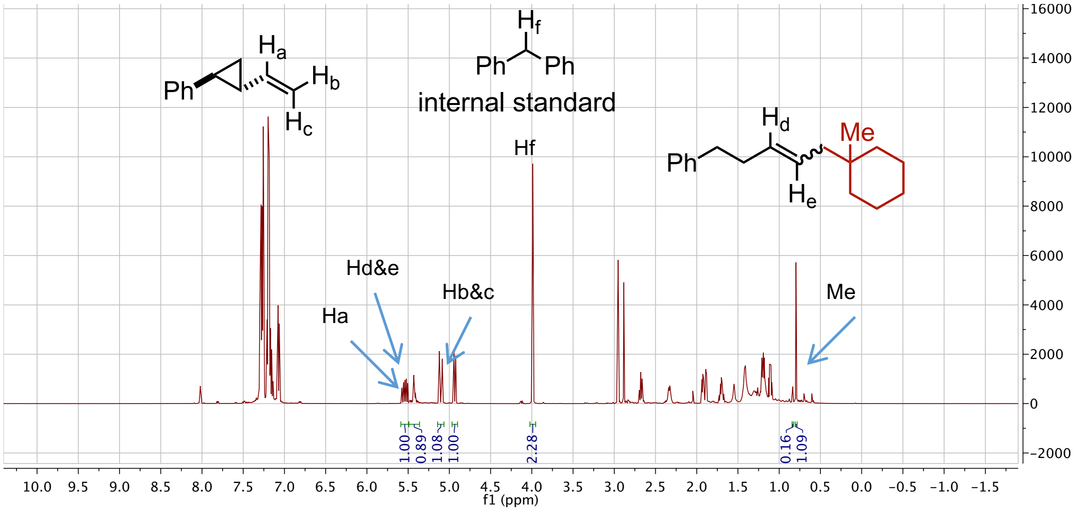

Supplementary Figure 9. ^{1}H NMR of the radical clock reaction crude

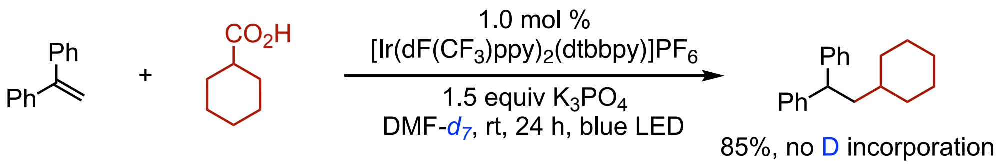

Procedure: In a nitrogen-filled glove box, an oven-dried 4-mL reaction vial equipped with a stir bar was charged with $[Ir(dF(CF_3)ppy)_2(dtbbpy)]PF_6$ (1.2 mg, 1 μmol, 1 mol %) and K_3PO_4 (31.8 mg, 0.15 mmol, 1.5 equiv). This was followed by addition of cyclohexane carboxylic acid (0.15 mmol, 1.5 equiv), 0.5 mL DMF-d_7, and 1,1-diphenylethylene (17.6 μL, 0.10 mmol, 1.0 equiv). The vial was sealed with a teflon-lined cap, removed from the glove box, and irradiated with two 24 W blue LEDs with stirring at 800 rpm at rt for 24 h. The reaction mixture was diluted with ethyl acetate (2.5 mL) and the internal standard 1-methylnaphthalene (10 μL) was added. The mixture was then washed with water (3x2 mL) to remove most of the DMF. The organic layer was sampled and the solvent removed under vacuum. This mixture was then analyzed by GC and ^1H NMR spectroscopy, and the yield was determined by GC (85%) by comparing to the calibration curve.

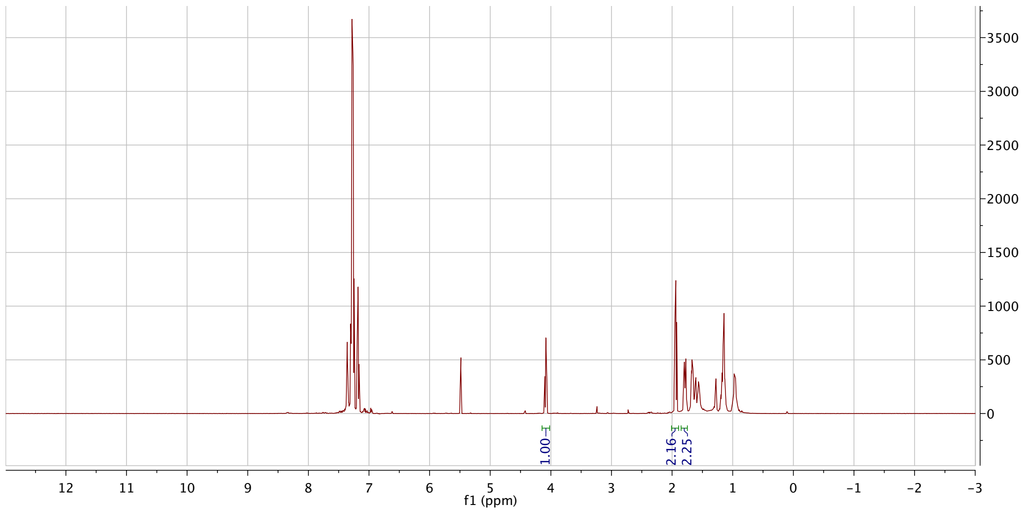

Supplementary Figure 10: ^1H NMR of the reaction crude in DMF-d7

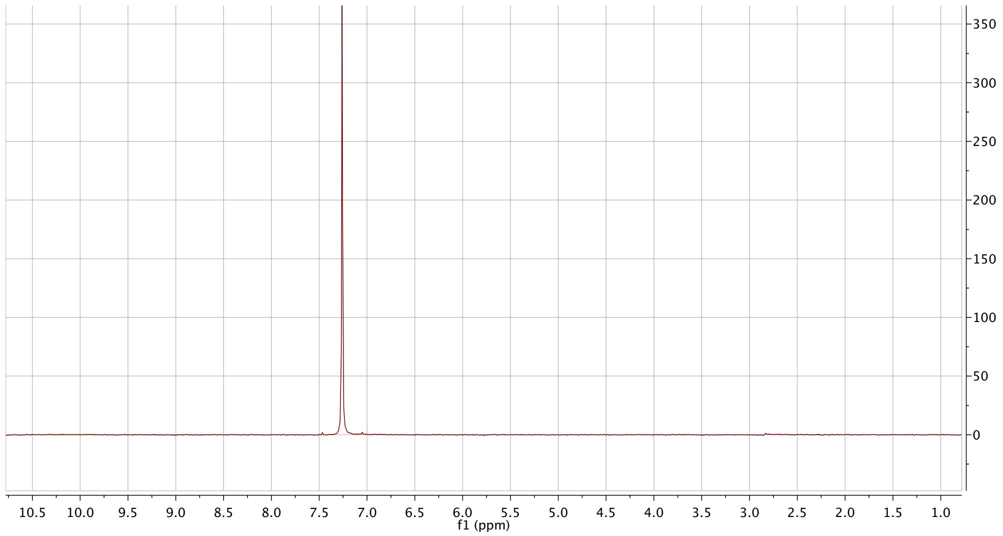

Supplementary Figure 11: ^2H NMR of the reaction crude in DMF-d7

F. Stern-Volmer Experiments

The fluorescence experiments were performed on a Horiba FluoroMax-4 fluorometer with FluorEssence (v3.5) software. All emission intensity data was measured using a quartz tube at 23°C. The samples were excited at 420 nm and emission was recorded at 581 nm.

The quenching experiments were run with freshly prepared solutions: 42uM $[Ir(ppy)_2(dtbbpy)]PF_6$, 0.1 M 1-(trimethylsilyl) methyl)piperidine, 0.1 M 1,1-diphenylethylene, and 0.1 M MeOH in DMF. I_0 was measured with 500uL of photocatalyst solution, and I was measured upon the addition of appropriate amount of quencher.

Supplementary Table 20: Fluorescence quenching $[Ir(ppy)_2(dtbbpy)]PF_6$ at variable concentrations of 1,1-diphenylethyl-ene.

Alkene (mM)	0	0.2	0.4	0.6	0.8
I/I_0	1	1.036	1.057	1.103	1.106

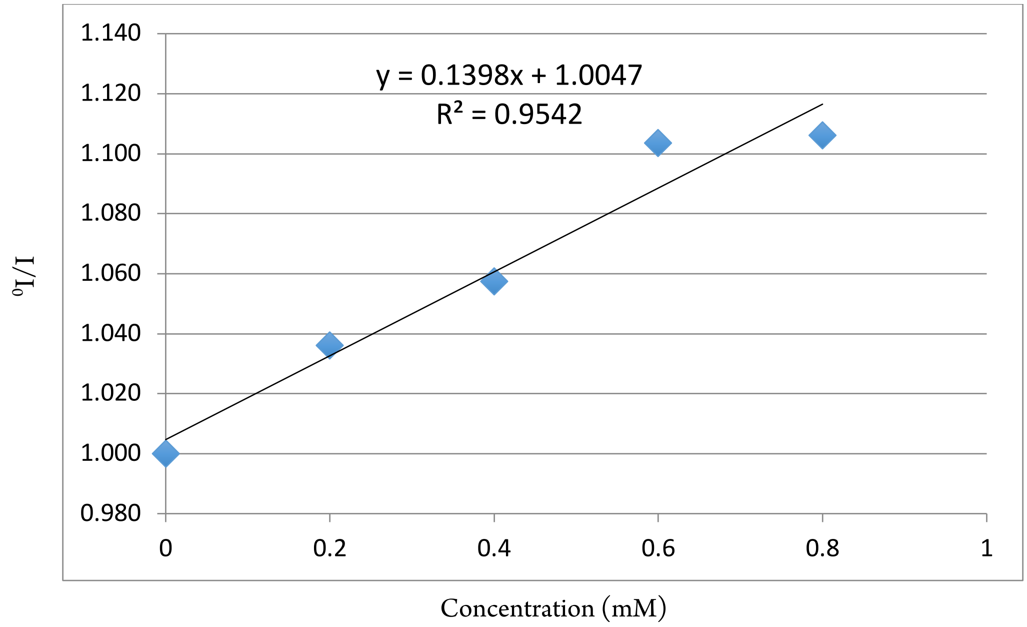

Supplementary Figure 12: Fluorescence quenching of photocatalyst at variable alkene concentrations

Supplementary Table 21: Fluorescence quenching $[Ir(ppy)_2(dtbbpy)]PF_6$ at variable concentrations of 1-(trimethylsilyl) methyl)piperidine.

Amine (mM)	0	0.2	0.4	0.6	0.8	1.0
I/I_0	1	1.154	1.192	1.234	1.282	1.314

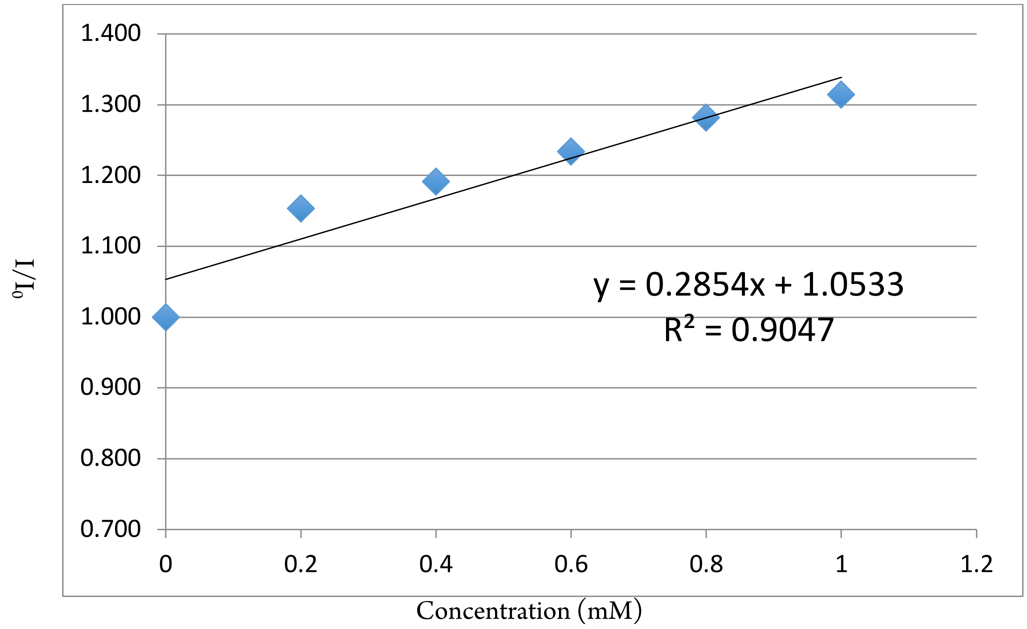

Supplementary Figure 13: Fluorescence quenching of photocatalyst at variable amine concentrations

Supplementary Table 22: Fluorescence quenching [Ir(ppy)$_2$(dtbbpy)]PF$_6$ at variable concentrations of 1-(trimethylsilyl) methyl)piperidine and MeOH

Amine (mM)	0	0.2	0.4	0.6
MeOH (mM)	0	0.4	0.8	1.2
I/I_0	1	1.050	1.121	1.188

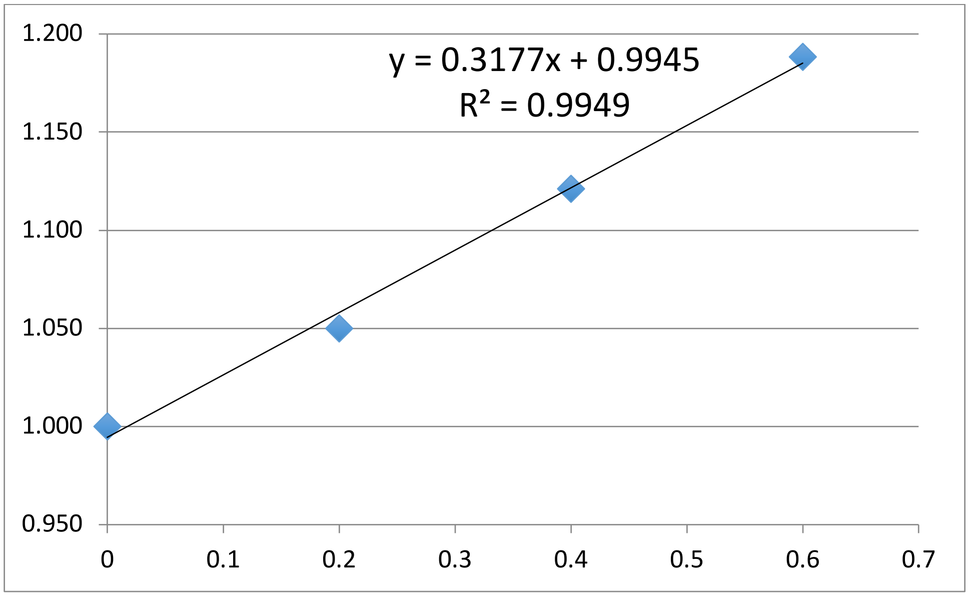

Supplementary Figure 14: Fluorescence quenching of photocatalyst at variable (amine+MeOH) concentrations

Although the 1,1-dipheylethylene quenches the photocatalyst reductively was well, the rate of quenching by amine or amine+MeOH is faster more than two folds.

G. Experimental Procedure, Isolation, and Characterization

Hydroalkylation Reactions:

1,1,3-triphenylpropane (1)

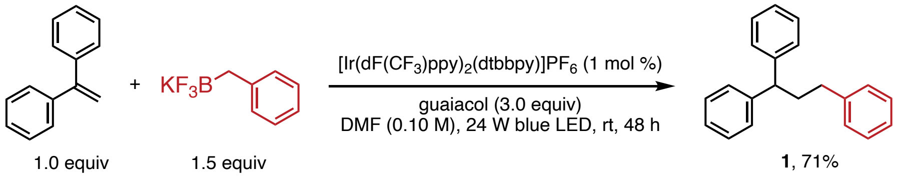

In a nitrogen-filled glove box, an oven-dried 4-mL reaction vial equipped with a stir bar was charged with [Ir(dF(CF_3)ppy)$_2$(dtbbpy)]PF_6 (2.3 mg, 2 µmol, 1 mol %) and potassium benzyltrifluoroborate (59.4 mg, 0.30 mmol, 1.5 equiv). This was followed by addition of anhydrous DMF (2000 µL, 0.10 M), 1,1-diphenylethylene (35.3 µL, 0.20 mmol, 1.0 equiv), and guaiacol (67 µL, 0.60 mmol, 3.0 equiv). The vial was sealed with a teflon-lined cap, removed from the glove box, and irradiated with two 24 W blue LEDs with stirring at 800 rpm at rt for 48 h. The crude mixture was then directly adsorbed onto diatomaceous earth (Celite®) and purified by flash column chromatography on silica (2% ethyl acetate in hexanes). The product **1** was obtained as a colorless oil (38.5 mg, 71%).

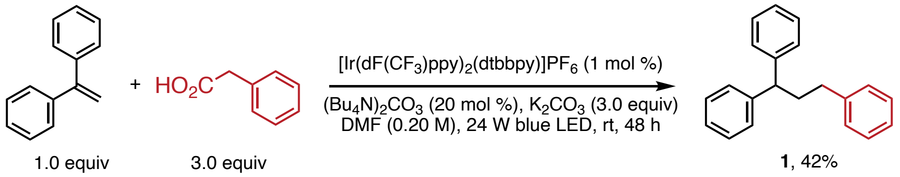

In a nitrogen-filled glove box, an oven-dried 4-mL reaction vial equipped with a stir bar was charged with [Ir(dF(CF_3)ppy)$_2$(dtbbpy)]PF_6 (1.2 mg, 1 µmol, 1 mol %), bis(tetrabutylammonium)carbonate (10.9 mg, 0.02 mmol, 0.20 equiv), potassium carbonate (41.4 mg, 0.30 mmol, 3.0 equiv), and phenylacetic acid (40.8 mg, 0.30 mmol, 3.0 equiv). This was followed by addition of anhydrous DMF (500 µL, 0.20 M) and 1,1-diphenylethylene (17.6 µL, 0.10 mmol, 1.0 equiv). The vial was sealed with a teflon-lined cap, removed from the glove box, and irradiated with two 24 W blue LEDs with stirring at 800 rpm at rt for 48 h. The reaction mixture was diluted with ethyl acetate (2.5 mL) and the internal standard 1-methylnaphthalene (10 µL) was added. The mixture was then washed with water (3x2 mL) to remove most of the DMF. The organic layer was sampled and the solvent removed under vacuum. This mixture was then analyzed by ^{1}H NMR spectroscopy and the yield of **1** was determined (45%, 39% for two independent runs, 42% average).

^{1}H NMR (400 MHz, $CDCl_3$) δ: 7.34 – 7.25 (m, 10H), 7.24 – 7.12 (m, 5H), 3.94 (t, J = 7.7 Hz, 1H), 2.60 (t, J = 7.7 Hz, 2H), 2.40 (q, J = 7.7 Hz, 2H).

^{13}C NMR (126 MHz, $CDCl_3$) δ: 145.09, 142.37, 128.75, 128.72, 128.61, 128.16, 126.44, 126.08, 50.96, 37.60, 34.38.

HRMS (ESI-TOF) m/z: [M+H$^+$] calculated for $C_{21}H_{20}$, 272.1565; found, 272.1566.

1,1,3-triphenylbutane (2)

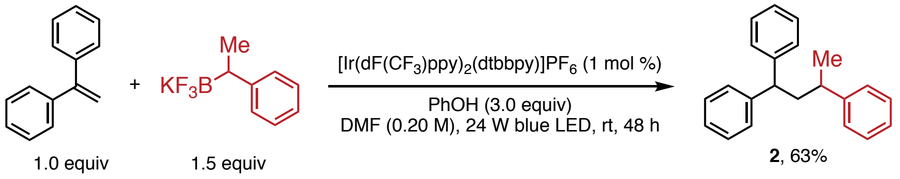

In a nitrogen-filled glove box, an oven-dried 4-mL reaction vial equipped with a stir bar was charged with [Ir(dF(CF_3)ppy)$_2$(dtbbpy)]PF_6 (2.3 mg, 2 μmol, 1 mol %), potassium 1-phenylethyl-1-trifluoroborate (63.6 mg, 0.30 mmol, 1.5 equiv), and phenol (56.5 mg, 0.60 mmol, 3.0 equiv). This was followed by addition of anhydrous DMF (1000 μL, 0.20 M) and 1,1-diphenylethylene (35.3 μL, 0.20 mmol, 1.0 equiv). The vial was sealed with a teflon-lined cap, removed from the glove box, and irradiated with two 24 W blue LEDs with stirring at 800 rpm at rt for 48 h. The crude mixture was then directly adsorbed onto diatomaceous earth (Celite®) and purified by flash column chromatography on silica (1% ethyl acetate in hexanes). The product **2** was obtained as a colorless oil (35.9 mg, 63%).

^{1}H NMR (400 MHz, $CDCl_3$): δ 7.35 – 7.28 (m, 4H), 7.28 – 7.24 (m, 2H), 7.24 – 7.18 (m, 6H), 7.18 – 7.12 (m, 3H), 3.79 (t, *J* = 7.9 Hz, 1H), 2.60 (sextet, *J* = 6.9 Hz, 1H), 2.36 (dd, *J* = 8.2, 7.0 Hz, 2H), 1.28 (d, *J* = 6.9 Hz, 3H).

^{13}C NMR (126 MHz, $CDCl_3$): δ 147.31, 145.53, 144.80, 128.73, 128.72, 128.67, 128.42, 127.97, 127.46, 126.44, 126.33, 126.30, 49.09, 44.53, 37.75, 23.05.

HRMS (ESI-TOF) *m/z*: [M+H$^+$] calculated for $C_{22}H_{22}$, 286.1722; found, 286.1726.

1,1-diphenyl-2-cyclohexylethane (3)

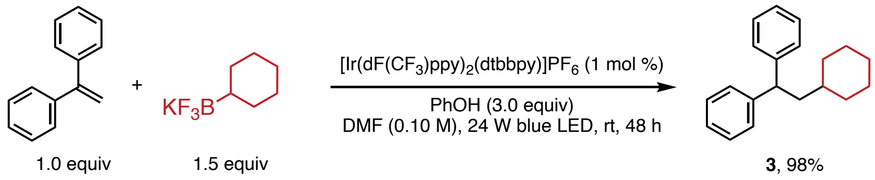

In a nitrogen-filled glove box, an oven-dried 4-mL reaction vial equipped with a stir bar was charged with [Ir(dF(CF_3)ppy)$_2$(dtbbpy)]PF_6 (2.3 mg, 2 μmol, 1 mol %), potassium cyclohexyltrifluoroborate (57.0 mg, 0.30 mmol, 1.5 equiv), and phenol (56.5 mg, 0.60 mmol, 3.0 equiv). This was followed by addition of anhydrous DMF (2000 μL, 0.10 M) and 1,1-diphenylethylene (35.3 μL, 0.20 mmol, 1.0 equiv). The vial was sealed with a teflon-lined cap, removed from the glove box, and irradiated with two 24 W blue LEDs with stirring at 800 rpm at rt for 48 h. The crude mixture was then directly adsorbed onto diatomaceous earth (Celite®) and purified by flash column chromatography on silica (1% ethyl acetate in hexanes). The product **3** was obtained as a colorless oil (51.9 mg, 98%).

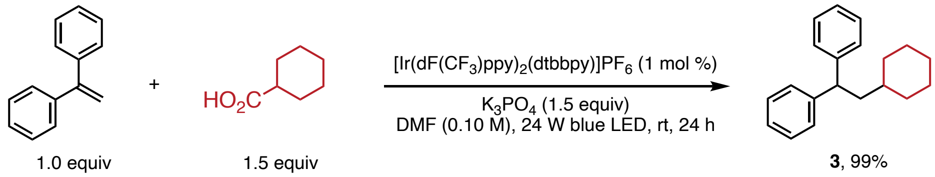

In a nitrogen-filled glove box, an oven-dried 4-mL reaction vial equipped with a stir bar was charged with [Ir(dF(CF_3)ppy)$_2$(dtbbpy)]PF_6 (1.2 mg, 1 μmol, 1 mol %) and K_3PO_4 (31.8 mg, 0.15 mmol, 1.5 equiv). This was followed by addition of 1000 μL of a stock solution of cyclohexane carboxylic acid (0.15 mmol, 1.5 equiv) in anhydrous DMF, and 1,1-diphenylethylene (17.6 μL, 0.10 mmol, 1.0 equiv). The vial was sealed with a teflon-lined cap, removed from the glove box, and irradiated with two 24 W blue LEDs with stirring at 800 rpm at rt for 24 h. The reaction mixture was diluted with ethyl acetate (2.5 mL) and the internal standard 1-methylnaphthalene (10 μL) was added. The mixture was then washed with water (3x2 mL) to remove most of the DMF. The organic layer was sampled and the solvent removed under vacuum. The product **3** was obtained in 99% yield.

^{1}H NMR (400 MHz, $CDCl_3$) δ: 7.34 – 7.23 (m, 8H), 7.23 – 7.16 (m, 2H), 4.10 (t, J = 8.0 Hz, 1H), 1.96 (dd, J = 7.9, 6.8 Hz, 2H), 1.86 – 1.73 (m, 2H), 1.71 – 1.59 (m, 3H), 1.24 – 1.09 (m, 4H), 1.07 – 0.90 (m, 2H).

^{13}C NMR (126 MHz, $CDCl_3$) δ: 145.72, 128.64, 128.16, 126.21, 48.24, 43.90, 35.14, 33.70, 26.93, 26.43.

HRMS (ESI-TOF) *m/z*: [M+H$^+$] calculated for $C_{20}H_{24}$, 264.1878; found, 264.1879.

1,1-diphenylhexane (4)

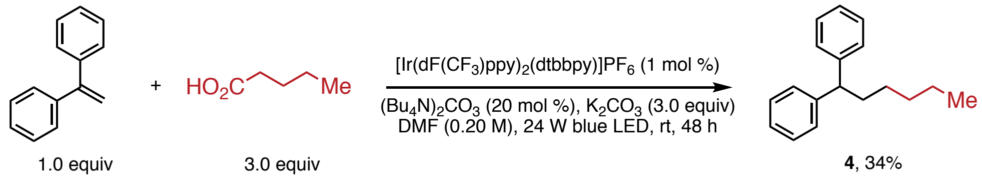

In a nitrogen-filled glove box, an oven-dried 4-mL reaction vial equipped with a stir bar was charged with [Ir(dF(CF_3)ppy)$_2$(dtbbpy)]PF_6 (1.2 mg, 1 μmol, 1 mol %), bis(tetrabutylammonium)carbonate (54.4 mg, 0.10 mmol, 1.0 equiv) and potassium carbonate (27.6 mg, 0.20 mmol, 2.0 equiv). This was followed by addition of anhydrous DMF (500 μL, 0.20 M), valeric acid (32.9 μL, 0.30 mmol, 3.0 equiv), and 1,1-diphenylethylene (17.6 μL, 0.10 mmol, 1.0 equiv). The vial was sealed with a teflon-lined cap, removed from the glove box, and irradiated with two 24 W blue LEDs with stirring at 800 rpm at rt for 48 h. The reaction mixture was diluted with ethyl acetate (2.5 mL) and the internal standard 1-methylnaphthalene (10 μL) was added. This mixture was then analyzed by GC and the yield of **4** was determined by comparison to a calibration curve prepared from an authentic sample (34%).

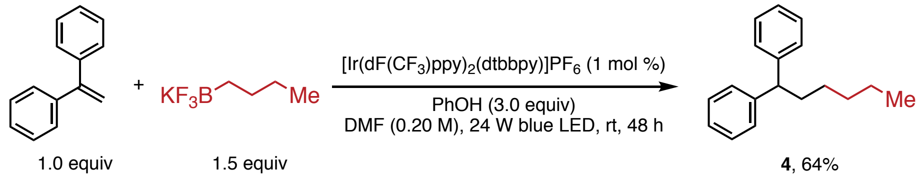

In a nitrogen-filled glove box, an oven-dried 4-mL reaction vial equipped with a stir bar was charged with [Ir(dF(CF_3)ppy)$_2$(dtbbpy)]PF_6 (2.3 mg, 2 μmol, 1 mol %), potassium butyltrifluoroborate (49.2 mg, 0.30 mmol, 1.5 equiv), and phenol (56.5 mg, 0.60 mmol, 3.0 equiv). This was followed by addition of anhydrous DMF (1000 μL, 0.20 M) and 1,1-diphenylethylene (35.3 μL, 0.20 mmol, 1.0 equiv). The vial was sealed with a teflon-lined cap, removed from the glove box, and irradiated with two 24 W blue LEDs with stirring at 800 rpm at rt for 48 h. The crude mixture was then directly adsorbed onto diatomaceous earth (Celite®) and purified by flash column chromatography on silica (pentane). The product **4** was obtained as a colorless oil (30.4 mg, 64%).

^{1}H NMR (500 MHz, $CDCl_3$) δ: 7.31 – 7.23 (m, 8H), 7.21 – 7.14 (m, 2H), 3.90 (t, *J* = 7.8 Hz, 1H), 2.05 (q, *J* = 7.8 Hz, 2H), 1.37 – 1.19 (m, 6H), 0.86 (t, *J* = 6.9 Hz, 3H).

^{13}C NMR (126 MHz, $CDCl_3$) δ: 145.64, 128.63, 128.14, 126.25, 51.66, 35.99, 32.15, 28.01, 22.82, 14.37.

HRMS (ESI-TOF) *m/z*: [M+H$^+$] calculated for $C_{18}H_{22}$, 238.1722; found, 238.1722.

methyl 5,5-diphenylpentanoate (5, R = Me)

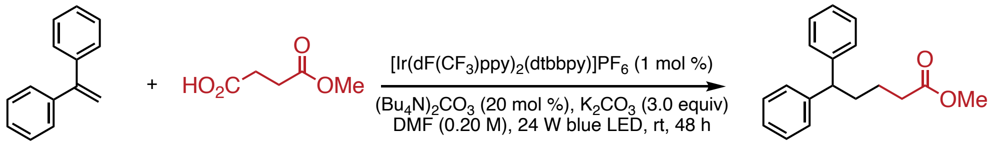

In a nitrogen-filled glove box, an oven-dried 4-mL reaction vial equipped with a stir bar was charged with $[Ir(dF(CF_3)ppy)_2(dtbbpy)]PF_6$ (1.2 mg, 1 µmol, 1 mol %), bis(tetrabutylammonium)carbonate (10.9 mg, 0.02 mmol, 0.20 equiv), potassium carbonate (41.4 mg, 0.30 mmol, 3.0 equiv), and 4-methoxy-4-oxobutanoic acid (39.6 mg, 0.30 mmol, 3.0 equiv). This was followed by addition of anhydrous DMF (500 µL, 0.20 M) and 1,1-diphenylethylene (17.6 µL, 0.10 mmol, 1.0 equiv). The vial was sealed with a teflon-lined cap, removed from the glove box, and irradiated with two 24 W blue LEDs with stirring at 800 rpm at rt for 48 h. The reaction mixture was diluted with ethyl acetate (2.5 mL) and the internal standard 1-methylnaphthalene (10 µL) was added. The mixture was then washed with water (3x2 mL) to remove most of the DMF. The organic layer was sampled and the solvent removed under vacuum. This mixture was then analyzed by ^1H NMR spectroscopy and the yield of **5** was determined (30%, 21% for two independent runs, 26% average).[12]

HRMS (ESI-TOF) *m/z*: $[M+H^+]$ calculated for $C_{18}H_{20}O_2$, 268.1463; found, 268.1461.

ethyl 5,5-diphenylpentanoate (5, R = Et)

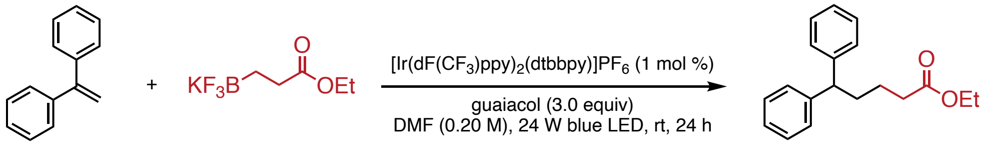

In a nitrogen-filled glove box, an oven-dried 4-mL reaction vial equipped with a stir bar was charged with $[Ir(dF(CF_3)ppy)_2(dtbbpy)]PF_6$ (1.2 mg, 1 µmol, 1 mol %) and potassium 4-ethoxy-4-oxopropyltrifluoroborate (31.2 mg, 0.15 mmol, 1.5 equiv). This was followed by addition of anhydrous DMF (500 µL, 0.20 M), 1,1-diphenylethylene (17.6 µL, 0.10 mmol, 1.0 equiv), and guaiacol (33.6 µL, 0.30 mmol, 3.0 equiv). The vial was sealed with a teflon-lined cap, removed from the glove box, and irradiated with two 24 W blue LEDs with stirring at 800 rpm at rt for 24 h. The reaction mixture was diluted with ethyl acetate (2.5 mL) and the internal standard 1-methylnaphthalene (10 µL) was added. GC and GCMS analysis indicated only trace product formation.

***tert*-butyl 4-(2-bromophenethyl)piperidine-1-carboxylate (6)**

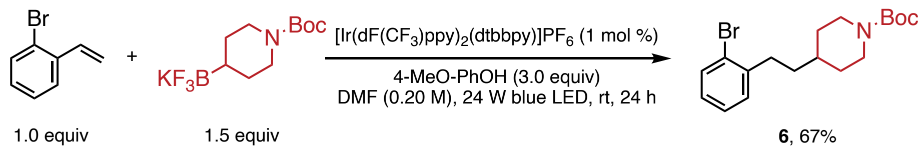

In a nitrogen-filled glove box, an oven-dried 4-mL reaction vial equipped with a stir bar was charged with $[Ir(dF(CF_3)ppy)_2(dtbbpy)]PF_6$ (2.3 mg, 2 µmol, 1 mol %), potassium *N*-boc-piperidine-4-trifluoroborate (87.3 mg, 0.30 mmol, 1.5 equiv), and 4-methoxyphenol (74.5 mg, 0.60 mmol, 3.0 equiv). This was followed by addition of anhydrous DMF (1000 µL, 0.20 M) and 2-bromostyrene (36.6 mg, 0.20 mmol, 1.0 equiv). The vial was sealed with a teflon-lined cap, removed from the glove box, and irradiated with two 24 W blue LEDs with stirring at 800 rpm at rt for 24 h. The crude mixture was then directly adsorbed onto diatomaceous earth (Celite®) and purified by flash column chromatography on silica (1:1:8 Et_2O:CH_2Cl_2:hexanes). The product **6** was obtained as a colorless oil, which was contaminated with ~25% of the protodeboronation byproduct. This could be removed under high vacuum with light heating to provide the pure product as a clear oil (49.2 mg, 67%).

^{1}H NMR (500 MHz, $CDCl_3$) δ: 7.59 – 7.45 (m, 1H), 7.25 – 7.17 (m, 2H), 7.12 – 7.01 (m, 1H), 4.09 (m, 2H), 2.89 – 2.59 (m, 4H), 1.79 – 1.68 (m, 2H), 1.61 – 1.50 (m, 2H), 1.46 (s, 9H), 1.45 – 1.42 (m, 1H), 1.22 – 1.11 (m, 2H).

^{13}C NMR (126 MHz, $CDCl_3$) δ: 155.16, 142.10, 133.09, 130.41, 127.78, 127.73, 124.64, 79.49, 44.16, 37.09, 36.16, 33.69, 32.33, 28.77.

HRMS (ESI-TOF) *m/z*: [M+H$^+$] calculated for $C_{18}H_{27}NO_2Br$, 368.1225; found, 368.1221.

4-(2-(cyclohex-2-en-1-yl)ethyl)pyridine (7)

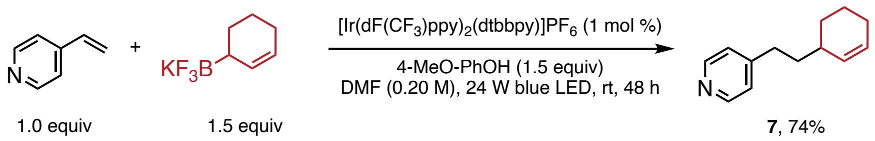

In a nitrogen-filled glove box, an oven-dried 4-mL reaction vial equipped with a stir bar was charged with $[Ir(dF(CF_3)ppy)_2(dtbbpy)]PF_6$ (2.3 mg, 2 µmol, 1 mol %), potassium 2-cyclohexenyltrifluoroborate (56.4 mg, 0.30 mmol, 1.5 equiv), and 4-methoxyphenol (37.2 mg, 0.30 mmol, 1.5 equiv). This was followed by addition of anhydrous DMF (1000 µL, 0.20 M) and 4-vinylpyridine (21.6 µL, 0.20 mmol, 1.0 equiv). The vial was sealed with a teflon-lined cap, removed from the glove box, and irradiated with two 24 W blue LEDs with stirring at 800 rpm at rt for 48 h. The crude mixture was then directly adsorbed onto diatomaceous earth (Celite®) and purified by flash column chromatography on silica (15-20% ethyl acetate in hexanes). The product **7** was obtained as a colorless oil (27.6 mg, 74%).

^{1}H NMR (500 MHz, $CDCl_3$) δ: 8.47 (d, *J* = 5.6 Hz, 2H), 7.11 (d, *J* = 5.5 Hz, 2H), 5.71 (ddd, *J* = 9.6, 6.1, 3.5 Hz, 1H), 5.63 – 5.50 (dd, *J* = 10.1, 1.5 Hz, 1H), 2.71 – 2.57 (m, 2H), 2.14 – 2.05 (m, 1H), 2.04 – 1.92 (m, 2H), 1.87 – 1.78 (m, 1H), 1.77 – 1.69 (m, 1H), 1.70 – 1.61 (m, 1H), 1.61 – 1.47 (m, 2H), 1.32 – 1.20 (m, 1H).

^{13}C NMR (126 MHz, $CDCl_3$) δ: 152.00, 149.92, 131.37, 127.89, 124.14, 37.20, 34.92, 32.85, 29.17, 25.59, 21.64.

HRMS (ESI-TOF) *m/z*: [M+H$^+$] calculated for $C_{13}H_{18}N$, 188.1439; found, 188.1443.

2-(2-(2-methylcyclohexyl)ethyl)pyridine (8)

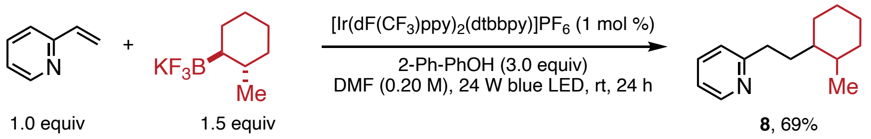

In a nitrogen-filled glove box, an oven-dried 4-mL reaction vial equipped with a stir bar was charged with [Ir(dF(CF$_3$)ppy)$_2$(dtbbpy)]PF$_6$ (2.3 mg, 2 µmol, 1 mol %), potassium *trans*-2-methylcyclohexyltrifluoroborate (61.2 mg, 0.30 mmol, 1.5 equiv), and 2-phenylphenol (102.1 mg, 0.60 mmol, 3.0 equiv). This was followed by addition of anhydrous DMF (1000 µL, 0.20 M) and 2-vinylpyridine (21.5 µL, 0.20 mmol, 1.0 equiv). The vial was sealed with a teflon-lined cap, removed from the glove box, and irradiated with two 24 W blue LEDs with stirring at 800 rpm at rt for 24 h. The crude mixture was then directly adsorbed onto diatomaceous earth (Celite®) and purified by flash column chromatography on alumina (100 mL alumina deactivated with 3 mL H_2O, eluting with 1:30 ethyl acetate:hexanes +0.5% MeOH ramping to 1:15 ethyl acetate:hexanes +1% MeOH). The product **8** was obtained as a colorless oil (28.1 mg, 69%). GC analysis of a crude reaction mixture indicated a diastereomeric ratio of 5:1.

^{1}H NMR (500 MHz, $CDCl_3$) δ: 8.51 (d, *J* = 5.5 Hz, 1H), 7.57 (t, *J* = 7.6 Hz, 1H), 7.14 (d, *J* = 7.8 Hz, 1H), 7.11 – 7.04 (m, 1H), 2.91 – 2.61 (m, 2H), 2.03 – 1.90 (m, 1H), 1.91 – 1.79 (m, 1H), 1.76 – 1.55 (m, 3H), 1.53 – 1.40 (m, 2H), 1.30 – 1.09 (m, 3H), 1.07 – 0.92 (m, 2H), 0.90 (d, *J* = 6.2 Hz, 3H).

^{13}C NMR (126 MHz, $CDCl_3$) δ: 163.35, 163.16, 149.40, 149.37, 136.55, 122.91, 122.88, 121.08, 121.05, 44.13, 40.14, 37.21, 36.60, 36.16, 35.60, 34.32, 32.89, 32.53, 32.00, 27.84, 26.91, 26.83, 25.39, 22.40, 20.61, 17.84, 14.18. (mixture of diastereomers).

HRMS (ESI-TOF) *m/z*: [M+H$^+$] calculated for $C_{14}H_{22}N$, 204.1752; found, 204.1757.

1-methoxy-4-(2-(1-methylcyclohexyl)ethyl)benzene (9)

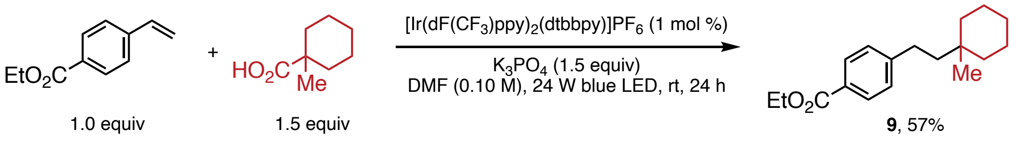

In a nitrogen-filled glove box, an oven-dried 4-mL reaction vial equipped with a stir bar was charged with [Ir(dF(CF_3)ppy)$_2$(dtbbpy)]PF_6 (2.3 mg, 2 μmol, 1 mol %), K_3PO_4 (63.7 mg, 0.30 mmol, 1.5 equiv), and 1-methylcyclohexane-1-carboxylic acid (42.7 mg, 0.30 mmol, 1.5 equiv). This was followed by addition of anhydrous DMF (2000 μL, 0.10 M) and 4-(ethoxycarbonyl)styrene (35.2 mg, 0.20 mmol, 1.0 equiv). The vial was sealed with a teflon-lined cap, removed from the glove box, and irradiated with two 24 W blue LEDs with stirring at 800 rpm at rt for 24 h. The crude mixture was then directly adsorbed onto diatomaceous earth (Celite®) and purified by flash column chromatography on silica (2.5% ethyl acetate in hexanes). The product **9** was obtained as a colorless oil (31.2 mg, 57%).

^{1}H NMR (500 MHz, $CDCl_3$) δ: 7.95 (d, J = 8.2 Hz, 2H), 7.24 (d, J = 8.2 Hz, 2H), 4.36 (q, J = 7.1 Hz, 2H), 2.63 – 2.55 (m, 2H), 1.56 – 1.50 (m, 2H), 1.49 – 1.43 (m, 5H), 1.39 (t, J = 7.1 Hz, 3H), 1.33 – 1.28 (m, 5H), 0.95 (s, 3H).

^{13}C NMR (126 MHz, $CDCl_3$) δ: 166.99, 149.61, 129.92, 128.61, 128.15, 61.00, 44.31, 38.06, 33.17, 30.49, 26.79, 25.25, 22.35, 14.65.

HRMS (ESI-TOF) *m/z*: [M+H$^+$] calculated for $C_{18}H_{27}O_2$, 275.2011; found, 275.2017.

1-methoxy-4-(2-(1-methylcyclohexyl)ethyl)benzene (10)

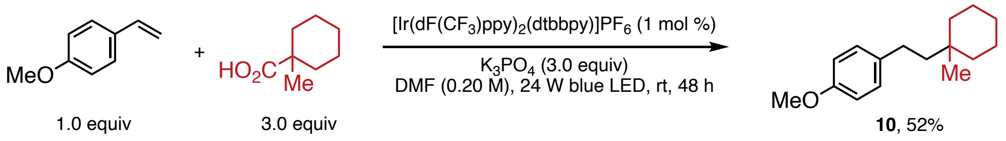

In a nitrogen-filled glove box, an oven-dried 4-mL reaction vial equipped with a stir bar was charged with [Ir(dF(CF_3)ppy)$_2$(dtbbpy)]PF_6 (2.3 mg, 2 μmol, 1 mol %), K_3PO_4 (127.4 mg, 0.60 mmol, 3.0 equiv), and 1-methylcyclohexane-1-carboxylic acid (85.4 mg, 0.60 mmol, 3.0 equiv). This was followed by addition of anhydrous DMF (1000 μL, 0.20 M) and 4-methoxystyrene (26.9 μL, 0.20 mmol, 1.0 equiv). The vial was sealed with a teflon-lined cap, removed from the glove box, and irradiated with two 24 W blue LEDs with stirring at 800 rpm at rt for 48 h. The crude mixture was then directly adsorbed onto diatomaceous earth (Celite®) and purified by flash column chromatography on silica (2.5% ethyl acetate in hexanes). The product **10** was obtained as a colorless oil, which was contaminated with ~10% yield of a dimer byproduct (31.2 mg, 57%). An analytically pure sample of **10** (26.2 mg) could be obtained by pulling the product into the upper half of the vial by applying high vacuum and heat. The heavier byproduct could then be rinsed away with pentane, leaving the pure product behind.

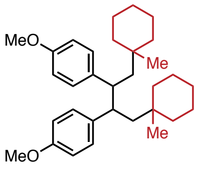

dimer byproduct

^{1}H NMR (500 MHz, $CDCl_3$) δ: 7.10 (d, J = 8.6 Hz, 2H), 6.83 (d, J = 8.6 Hz, 2H), 3.79 (s, 3H), 2.52 – 2.45 (m, 2H), 1.53 – 1.41 (m, 7H), 1.37 – 1.20 (m, 5H), 0.94 (s, 3H).

^{13}**C NMR** (126 MHz, $CDCl_3$) δ: 157.83, 136.16, 129.45, 114.04, 55.57, 44.90, 38.12, 33.08, 29.33, 26.86, 25.39, 22.38.

HRMS (ESI-TOF) *m/z*: [M+H$^+$] calculated for $C_{16}H_{25}O$, 233.1905; found, 233.1901.

1-methyl-4-(2-(1-methylcyclohexyl)ethyl)benzene (11)

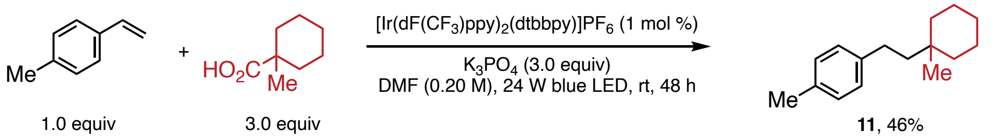

In a nitrogen-filled glove box, an oven-dried 4-mL reaction vial equipped with a stir bar was charged with [Ir(dF(CF_3)ppy)$_2$(dtbbpy)]PF_6 (2.3 mg, 2 μmol, 1 mol %), K_3PO_4 (127.4 mg, 0.60 mmol, 3.0 equiv), and 1-methylcyclohexane-1-carboxylic acid (85.4 mg, 0.60 mmol, 3.0 equiv). This was followed by addition of anhydrous DMF (1000 μL, 0.20 M) and 4-methylstyrene (26.4 μL, 0.20 mmol, 1.0 equiv). The vial was sealed with a teflon-lined cap, removed from the glove box, and irradiated with two 24 W blue LEDs with stirring at 800 rpm at rt for 48 h. The crude mixture was then directly adsorbed onto diatomaceous earth (Celite®) and purified by flash column chromatography on silica (pentane). The product **11** was obtained as a colorless oil, which was contaminated with ~10% yield of a dimer byproduct (20.0 mg, 46%). An analytically pure sample of **11** (14.0 mg) could be obtained by pulling the product into the upper half of the vial by applying high vacuum and heat. The heavier byproduct could then be rinsed away with pentane, leaving the pure product behind.

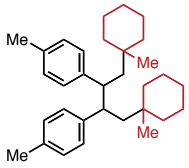

dimer byproduct

^{1}**H NMR** (500 MHz, $CDCl_3$) δ: 7.11 – 7.06 (m, 4H), 2.57 – 2.46 (m, 2H), 2.32 (s, 3H), 1.58 – 1.40 (m, 7H), 1.38 – 1.25 (m, 5H), 0.95 (s, 3H).

^{13}**C NMR** (126 MHz, $CDCl_3$) δ: 141.02, 135.14, 129.27, 128.50, 44.78, 38.12, 33.10, 29.84, 26.86, 25.40, 22.38, 21.27.

HRMS (ESI-TOF) *m/z*: [M+H$^+$] calculated for $C_{16}H_{24}$, 216.1878; found, 216.1877.

(2-(1-methylcyclohexyl)ethyl)benzene (12)

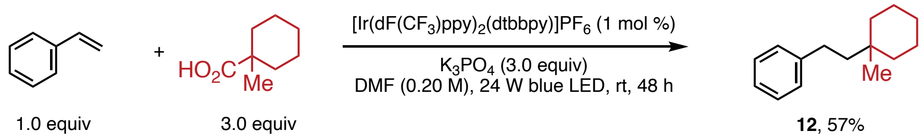

12, 57%

In a nitrogen-filled glove box, an oven-dried 4-mL reaction vial equipped with a stir bar was charged with [Ir(dF(CF_3)ppy)$_2$(dtbbpy)]PF_6 (2.3 mg, 2 μmol, 1 mol %), K_3PO_4 (127.4 mg, 0.60 mmol, 3.0 equiv), and 1-methylcyclohexane-1-carboxylic acid (85.4 mg, 0.60 mmol, 3.0 equiv). This was followed by addition of anhydrous DMF (1000 μL, 0.20 M) and styrene (22.9 μL, 0.20 mmol, 1.0 equiv). The vial was sealed with a teflon-lined cap, removed from the glove box, and irradiated with two 24 W blue LEDs with stirring at 800 rpm at rt for 48 h. The crude mixture was then directly adsorbed onto diatomaceous earth (Celite®) and purified by flash column chromatography on silica (pentane). The product **12** was obtained as a colorless oil (29.2 mg, 72%).

^{1}H NMR (500 MHz, $CDCl_3$) δ: 7.31 – 7.25 (m, 2H), 7.22 – 7.15 (m, 3H), 2.58 – 2.51 (m, 2H), 1.57 – 1.51 (m, 3H), 1.48 – 1.43 (m, 5H), 1.39 – 1.25 (m, 4H), 0.96 (s, 3H).

^{13}C NMR (126 MHz, $CDCl_3$) δ: 144.11, 128.64, 128.57, 125.74, 44.63, 38.11, 33.12, 30.33, 26.85, 25.38, 22.38.

HRMS (ESI-TOF) *m/z*: [M+H$^+$] calculated for $C_{15}H_{22}$, 202.1722; found, 202.1721.

***tert*-butyl ((4-(2,2-diphenylethyl)cyclohexyl)methyl)carbamate (13)**

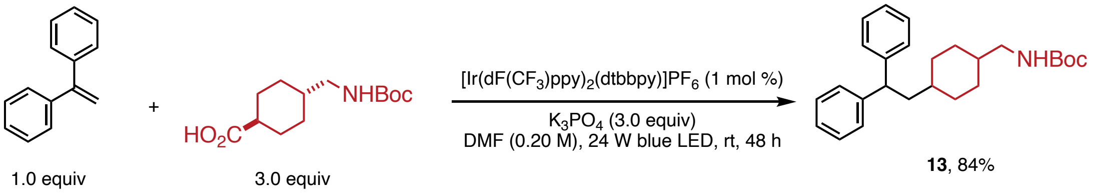

13, 84%

In a nitrogen-filled glove box, an oven-dried 4-mL reaction vial equipped with a stir bar was charged with [Ir(dF(CF_3)ppy)$_2$(dtbbpy)]PF_6 (2.3 mg, 2 μmol, 1 mol %), K_3PO_4 (127.4 mg, 0.60 mmol, 3.0 equiv), and *N*-boc-tranexamic acid (154.4 mg, 0.60 mmol, 3.0 equiv). This was followed by addition of anhydrous DMF (1000 μL, 0.20 M) and 1,1-diphenylethylene (35.3 μL, 0.20 mmol, 1.0 equiv). The vial was sealed with a teflon-lined cap, removed from the glove box, and irradiated with two 24 W blue LEDs with stirring at 800 rpm at rt for 48 h. The crude mixture was then directly adsorbed onto diatomaceous earth (Celite®) and purified by flash column chromatography on alumina (115 mL alumina deactivated with 10 mL H_2O, eluting with 3% ethyl acetate in hexanes + 1% MeOH). The product **13** was obtained as a colorless oil (64.0 mg, 81%). ^{1}H NMR of a crude reaction indicated the diastereomeric ratio to be 1:1.

^{1}H NMR (500 MHz, $CDCl_3$) δ: 7.32 – 7.23 (m, 8H), 7.18 (t, J = 6.9 Hz, 2H), 4.56 (br s, 1H), 4.08 (t, J = 7.9 Hz, 0.5H), 4.02 (t, J = 8.0 Hz, 0.5H), 3.07 (t, J = 6.3 Hz, 1H), 2.94 (t, J = 5.9 Hz, 1H), 2.04 (t, J = 7.5 Hz, 1H), 1.95 (t, J = 7.3 Hz, 1H), 1.86 (d, J = 12.3 Hz, 1H),

1.72 (d, J = 11.3 Hz, 1H), 1.57 (m, 1H), 1.46 (apparent doublet, 9H), 1.40 – 1.33 (m, 2H), 1.27 – 1.07 (m, 1H), 1.04 – 0.91 (m, 1H), 0.89 – 0.77 (m, 1H). (1:1 mixture of diastereomers)

^{13}C NMR (126 MHz, $CDCl_3$) δ: 156.36, 145.46, 128.65, 128.10, 126.26, 79.24, 48.76, 48.36, 47.05, 44.81, 43.70, 40.23, 38.67, 36.58, 35.21, 32.98, 32.55, 30.62, 28.94, 28.70, 26.57. (1:1 mixture of diastereomers)

HRMS (ESI-TOF) *m/z*: [M+Na$^+$] calculated for $C_{26}H_{35}NO_2Na$, 416.2565; found, 416.2558.

2-((4,4-dimethyl-6-phenylhexyl)oxy)-1,4-dimethylbenzene (14)

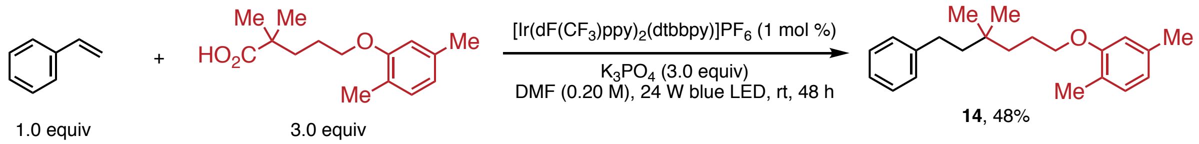

In a nitrogen-filled glove box, an oven-dried 4-mL reaction vial equipped with a stir bar was charged with $[Ir(dF(CF_3)ppy)_2(dtbbpy)]PF_6$ (2.3 mg, 2 μmol, 1 mol %), K_3PO_4 (127.4 mg, 0.60 mmol, 3.0 equiv), and gemfibrozil (150.2 mg, 0.60 mmol, 3.0 equiv). This was followed by addition of anhydrous DMF (1000 μL, 0.20 M) and styrene (22.9 μL, 0.20 mmol, 1.0 equiv). The vial was sealed with a teflon-lined cap, removed from the glove box, and irradiated with two 24 W blue LEDs with stirring at 800 rpm at rt for 48 h. The crude mixture was then directly adsorbed onto diatomaceous earth (Celite®) and purified by flash column chromatography on silica (1-2.5% ethyl acetate in hexanes). The product **14** was obtained as a colorless oil, which was contaminated with 5% of a dimer byproduct and 5% of a double-addition byproduct (33.2 mg, 48%).

^{1}H NMR (400 MHz, $CDCl_3$) δ: 7.38 – 7.32 (m, 2H), 7.24 (m, 3H), 7.11 – 7.05 (m, 1H), 6.75 – 6.68 (m, 2H), 4.01 (t, J = 6.4 Hz, 2H), 2.68 – 2.59 (m, 2H), 2.38 (s, 3H), 2.26 (s, 3H), 1.90 – 1.79 (m, 2H), 1.65 – 1.56 (m, 2H), 1.54 – 1.48 (m, 2H), 1.05 (s, 6H).

^{13}C NMR (126 MHz, $CDCl_3$) δ: 157.34, 143.74, 136.72, 130.56, 128.60, 128.59, 125.82, 123.86, 120.86, 112.22, 68.81, 44.43, 38.20, 33.03, 31.02, 27.52, 24.59, 21.72, 16.13.

HRMS (ESI-TOF) *m/z*: [M+H$^+$] calculated for $C_{22}H_{31}O$, 311.2375; found, 311.2374.

(6-(2,5-dimethylphenoxy)-3,3-dimethylhexane-1,1-diyl)dibenzene (15)

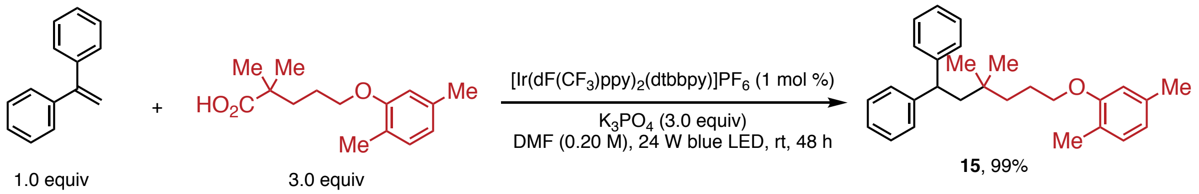

In a nitrogen-filled glove box, an oven-dried 4-mL reaction vial equipped with a stir bar was charged with $[Ir(dF(CF_3)ppy)_2(dtbbpy)]PF_6$ (2.3 mg, 2 μmol, 1 mol %), K_3PO_4 (127.4 mg, 0.60 mmol, 3.0 equiv), and gemfibrozil (150.2 mg, 0.60 mmol, 3.0 equiv). This was followed by addition of anhydrous DMF (1000 μL, 0.20 M) and 1,1-diphenylethylene (35.3 μL, 0.20 mmol, 1.0 equiv). The vial was sealed with a teflon-lined cap, removed from the glove box, and irradiated with two 24 W blue LEDs with stirring at 800 rpm at rt for 48 h. The crude mixture was then directly adsorbed onto diatomaceous earth (Celite®) and purified by flash column chromatography on silica (2.5% ethyl acetate in hexanes). The product **15** was obtained as a colorless oil (76.4 mg, 99%).

^{1}H NMR (400 MHz, $CDCl_3$) δ: 7.33 – 7.29 (m, 4H), 7.28 – 7.21 (m, 4H), 7.16 – 7.10 (m, 2H), 7.01 (d, *J* = 7.5 Hz, 1H), 6.66 (d, *J* = 7.5 Hz, 1H), 6.59 (s, 1H), 4.06 (t, *J* = 6.6 Hz, 1H), 3.79 (t, *J* = 6.4 Hz, 2H), 2.31 (s, 3H), 2.19 (s, 3H), 2.14 (d, *J* = 6.6 Hz, 2H), 1.75 – 1.63 (m, 2H), 1.39 – 1.32 (m, 2H), 0.82 (s, 6H).

^{13}C NMR (126 MHz, $CDCl_3$) δ: 157.35, 146.98, 136.71, 130.55, 128.72, 128.05, 126.18, 123.88, 120.87, 112.34, 68.80, 48.27, 47.39, 38.92, 34.09, 28.32, 24.58, 21.71, 16.15.

HRMS (ESI-TOF) *m/z*: [M+H$^+$] calculated for $C_{28}H_{35}O$, 387.2688; found, 387.2683.

Hydroaminoalkylation reactions:

***tert*-butyl 2-phenethylpyrrolidine-1-carboxylate [(±)-16]**

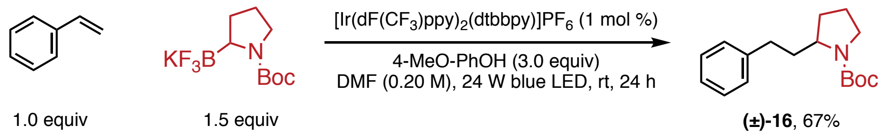

In a nitrogen-filled glove box, an oven-dried 4-mL reaction vial equipped with a stir bar was charged with [Ir(dF(CF_3)ppy)$_2$(dtbbpy)]PF_6 (2.3 mg, 2 μmol, 1 mol %), potassium *N*-boc-pyrrolidine-2-trifluoroborate (83.1 mg, 0.30 mmol, 1.5 equiv), and 4-methoxyphenol (74.5 mg, 0.60 mmol, 3.0 equiv). This was followed by addition of anhydrous DMF (1000 μL, 0.20 M) and styrene (22.9 μL, 0.20 mmol, 1.0 equiv). The vial was sealed with a teflon-lined cap, removed from the glove box, and irradiated with two 24 W blue LEDs with stirring at 800 rpm at rt for 24 h. The crude mixture was then directly adsorbed onto diatomaceous earth (Celite®) and purified by flash column chromatography on silica (1:1:8 Et_2O:CH_2Cl_2:hexanes). The product **(±)-16** was obtained as a colorless oil (36.7 mg, 67%).

^{1}H NMR (500 MHz, $CDCl_3$) δ: 7.32 – 7.24 (m, 2H), 7.23 – 7.16 (m, 3H), 3.95 – 3.68 (m, 1H), 3.56 – 3.23 (m, 2H), 2.97 – 2.41 (m, 2H), 2.25 – 1.88 (m, 2H), 1.92 – 1.74 (m, 2H), 1.77 – 1.54 (m, 2H), 1.45 (s, 9H).

^{13}C NMR (126 MHz, $CDCl_3$) δ: 154.92, 142.25, 128.59, 128.12, 126.03, 79.32, 79.21, 57.56, 57.18, 46.79, 46.42, 36.72, 36.27, 33.96, 33.10, 30.92, 30.34, 28.91, 28.85, 24.16, 23.46. (mixture of rotamers, see VT-NMR studies elsewhere in this document for more information).

^{13}C NMR (126 MHz, toluene-*d8*, rt) δ: 154.30, 154.16, 142.50, 142.26, 137.46, 128.57, 128.52, 126.07, 125.93, 78.36, 57.42, 56.93, 46.68, 46.56, 36.98, 36.25, 33.13, 30.77, 30.13, 28.63, 24.10, 23.28. (mixture of rotamers, see VT-NMR studies elsewhere in this document for more information).

^{13}C NMR (126 MHz, toluene-*d8*, 100 °C) δ: 154.60, 142.80, 137.73, 128.75, 126.14, 78.68, 57.68, 46.88, 36.85, 33.26, 31.06, 28.94, 24.00. (rotamers resolved, see VT-NMR studies elsewhere in this document for more information).

HRMS (ESI-TOF) *m/z*: [M+H$^+$] calculated for $C_{17}H_{26}NO_2$, 276.1964; found, 276.1972.

tert-butyl 2-(1-phenylpropan-2-yl)pyrrolidine-1-carboxylate [(±)-**17**]

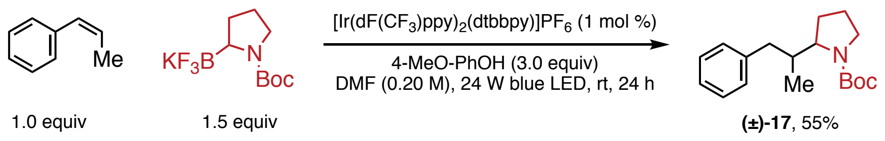

In a nitrogen-filled glove box, an oven-dried 4-mL reaction vial equipped with a stir bar was charged with $[Ir(dF(CF_3)ppy)_2(dtbbpy)]PF_6$ (2.3 mg, 2 µmol, 1 mol %), potassium *N*-boc-pyrrolidine-2-trifluoroborate (83.1 mg, 0.30 mmol, 1.5 equiv), and 4-methoxyphenol (74.5 mg, 0.60 mmol, 3.0 equiv). This was followed by addition of anhydrous DMF (1000 µL, 0.20 M) and *(Z)*-β-methylstyrene (26.0 µL, 0.20 mmol, 1.0 equiv). The vial was sealed with a teflon-lined cap, removed from the glove box, and irradiated with two 24 W blue LEDs with stirring at 800 rpm at rt for 24 h. The crude mixture was then directly adsorbed onto diatomaceous earth (Celite®) and purified by flash column chromatography on silica (1:1:8 Et_2O:CH_2Cl_2:hexanes). The product (±)-**17** was obtained as a colorless oil (32.0 mg, 55%).

^{1}H NMR (500 MHz, $CDCl_3$) δ: 7.28 – 7.23 (m, 2H), 7.20 – 7.10 (m, 3H), 3.94 – 3.68 (m, 1H), 3.67 – 3.42 (m, 1H), 3.34 – 3.08 (m, 1H), 2.91 – 2.51 (m, 1H), 2.51 – 2.34 (m, 1H), 2.29 – 2.08 (m, 1H), 1.99 – 1.71 (m, 4H), 1.47 (apparent doublet, 9H), 0.78 (apparent doublet, 3H).

^{13}C NMR (126 MHz, $CDCl_3$) δ: 155.30, 155.16, 141.75, 141.30, 129.34, 129.21, 128.48, 128.43, 126.05, 125.90, 79.40, 79.13, 62.39, 61.58, 60.75, 47.69, 47.34, 40.99, 40.71, 38.98, 38.02, 37.91, 37.08, 30.61, 29.98, 28.85, 27.57, 26.73, 26.26, 24.68, 24.20, 16.35, 13.98. (mixture of diastereomers and rotamers, see VT-NMR studies elsewhere in this document for more information).

^{13}C NMR (126 MHz, toluene-*d8*, rt) δ: 154.57, 154.47, 141.41, 141.39, 129.45, 129.29, 128.52, 128.49, 125.99, 125.96, 78.48, 62.30, 61.37, 47.62, 47.52, 41.05, 40.86, 38.10, 28.63, 26.65, 25.72, 24.54, 24.09, 16.22, 13.81. (mixture of diastereomers and rotamers, see VT-NMR studies elsewhere in this document for more information).

^{13}C NMR (126 MHz, toluene-*d8*, 100 °C) δ: 154.98, 154.85, 142.20, 141.76, 129.60, 129.47, 128.66, 128.63, 126.20, 126.12, 78.76, 62.72, 61.59, 47.81, 47.75, 41.31, 38.97, 28.94, 27.79, 26.63, 24.61, 24.51, 16.49, 14.27. (rotamers resolved, still a mixture of diastereomers, see VT-NMR studies elsewhere in this document for more information).

HRMS (ESI-TOF) *m/z*: [M+H$^+$] calculated for $C_{18}H_{28}NO_2$, 290.2120; found, 290.2123.

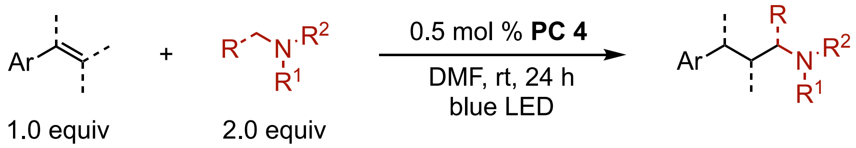

General procedure C: [Ir(dF(CF_3)ppy)$_2$(dtbbpy)]PF_6 (**PC4**, 1.2 mg, 0.001 mmol, 0.5 mol %), alkene (0.2 mmol, 1.0 equiv), tertiary amine (0.4 mmol, 2.0 equiv), and DMF (1.0 mL) were added to a 4 mL vial equipped with a stir bar in the glove box under nitrogen atmosphere. The vial was then taken out of glove box and allowed to stir inside the photo-reactor (described above) with the lamp and fan on for 24 hours. The reaction crude was quenched by the addition of DCM, concentrated *in vacuo* and then purified by basic alumina chromatography to afford the desired product.

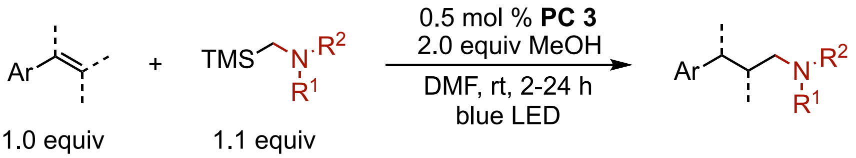

General procedure D: [Ir(ppy)$_2$(dtbbpy)]PF_6 (**PC 3**, 0.9 mg, 0.001 mmol, 0.5 mol %), alkene (0.2 mmol, 1.0 equiv), α-TMS amine (0.22 mmol, 1.1 equiv), and DMF (2.0 mL) were added to a 4 mL vial equipped with a stir bar in the glove box under nitrogen atmosphere. The vial was then taken out of glove box and injected 8 uL MeOH (0.4 mmol, 2.0 equiv). The resulting solution was allowed to stir inside the photo-reactor (described above) with the lamp and fan on for 2 to 24 hours. The reaction crude was quenched by the addition of DCM, concentrated *in vacuo* and then purified by basic alumina chromatography to afford the desired product.

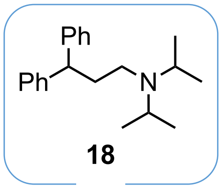

***N,N*-diisopropyl-3,3-diphenylpropan-1-amine (Diisopromine, 18, Fig 3):** Prepared according to General procedure C from 1,1-diphenylethylene with diisopropylmethylamine for 20 hours in 76% isolated yield as a colorless liquid.

Column Chromatography Condition: 100 g Al_2O_3 + 3 g H_2O, 99 : 1 hexanes/EtOAc with 0.5% MeOH to 60 : 1 hexanes/EtOAc with 1.0% MeOH as gradient eluent.

^{1}H NMR (500 MHz, $CDCl_3$) δ: 7.31 – 7.23 (m, 8H), 7.20 – 7.13 (m, 2H), 3.96 (t, J = 7.6 Hz, 1H), 2.98 (hept, J = 6.6 Hz, 2H), 2.43 – 2.31 (m, 2H), 2.18 (q, J = 7.6 Hz, 2H) 0.93 (d, J = 6.6 Hz, 12H).

^{13}C NMR (125 MHz, $CDCl_3$) δ: 145.38, 128.50, 128.00, 126.13, 49.32, 48.79, 44.08, 37.45, 20.78.

HRMS (ESI-TOF) *m/z*: [M+H$^+$] calculated for $C_{21}H_{30}N$, 296.2378; found, 296.2368.

(±)-**19**

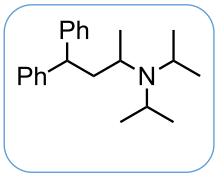

***N,N*-diisopropyl-4,4-diphenylbutan-2-amine ((±)-19, Fig 3):** Prepared according to General procedure C from 1,1-diphenylethylene with diisopropylethylamine for 20 hours in 78% isolated yield as a colorless liquid.

Column Chromatography Condition: 100 g Al_2O_3 + 1.5 g H_2O, 99 : 1 hexanes/EtOAc with 0.2% MeOH as eluent.

^{1}H NMR (500 MHz, $CDCl_3$) δ: 7.37 – 7.27 (m, 4H), 7.25 – 7.10 (m, 6H), 4.14 (dd, J = 9.2, 5.9 Hz, 1H), 3.13 (hept, J = 6.6 Hz, 2H), 2.86 – 2.69 (m, 1H), 2.10 – 1.91 (m, 2H), 1.05 (d, J = 6.5 Hz, 3H), 0.94 (d, J = 6.6 Hz, 6H), 0.91 (d, J = 6.7 Hz, 6H).

^{13}C NMR (125 MHz, $CDCl_3$) δ: 146.90, 144.69, 128.50, 128.46, 128.37, 128.00, 126.02, 125.88, 48.39, 47.18, 44.44, 42.80, 24.41, 22.39, 21.04.

HRMS (ESI-TOF) *m/z*: [M+H$^+$] calculated for $C_{22}H_{32}N$, 310.2535; found, 310.2548.

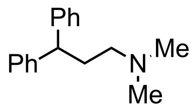

20

***N,N*-dimethyl-3,3-diphenylpropan-1-amine (20, Fig 3):** Prepared according to General procedure C from 1,1-diphenylethylene with 3 equiv trimethylamine•HCl and 3 equiv DBU for 24 hours in 71% isolated yield as a white solid. **m.p.** = 41–43 °C.

Column Chromatography Condition: 100 g Al_2O_3 + 9 g H_2O, 30 : 1 hexanes/EtOAc with 0.5% MeOH to 15 : 1 hexanes/EtOAc with 1.0% MeOH as gradient eluent.

^{1}H NMR (500 MHz, $CDCl_3$) δ: 7.31 – 7.22 (m, 8H), 7.20 – 7.15 (m, 2H), 3.99 (t, J = 6.6 Hz, 1H), 2.42 – 2.01 (m, 10H).

^{13}C NMR (125 MHz, $CDCl_3$) δ: 145.03, 128.58, 127.96, 126.26, 58.22, 49.14, 45.74, 33.82.

HRMS (ESI-TOF) *m/z*: [M+H$^+$] calculated for $C_{17}H_{22}N$, 240.1752; found, 240.1747.

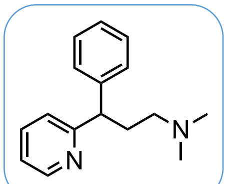

21

***N,N*-dimethyl-3-phenyl-3-(pyridin-2-yl)propan-1-amine (Pheniramine, 21, Fig 3):** Prepared according to General procedure C from 2-(1-phenylvinyl)pyridine with 3 equiv trimethylamine•HCl and 3 equiv DBU for 24 hours in 76% isolated yield as a colorless liquid.

Column Chromatography Condition: 100 g Al_2O_3 + 6 g H_2O, 6 : 1 hexanes/EtOAc with 0.5% MeOH to 3 : 1 hexanes/EtOAc with 1.0% MeOH as gradient eluent.

^{1}H NMR (500 MHz, $CDCl_3$) δ: 8.60 – 8.51 (m, 1H), 7.52 (td, J = 7.7, 1.6 Hz, 1H), 7.36 – 7.30 (m, 2H), 7.29 – 7.22 (m, 2H), 7.20 – 7.11 (m, 2H), 7.08 – 7.02 (m, 1H), 4.12 (t, J = 7.3 Hz, 1H), 2.49 – 2.36 (m, 1H), 2.27 – 2.13 (m, 9H).

^{13}C NMR (125 MHz, $CDCl_3$) δ: 163.83, 149.37, 143.83, 136.48, 128.61, 128.16, 126.52, 122.92, 121.38, 58.04, 51.51, 45.66, 33.06.

HRMS (ESI-TOF) *m/z*: [M+H$^+$] calculated for $C_{16}H_{21}N_2$, 241.1705; found, 241.1703.

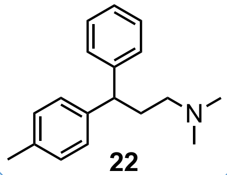

22

***N,N*-dimethyl-3-phenyl-3-(p-tolyl)propan-1-amine (Tolpropamine, 22, Fig 3):** Prepared according to General procedure C from 1-methyl-4-(1-phenylvinyl)benzene with 3 equiv trimethylamine•HCl and 3 equiv DBU for 24 hours in 71% isolated yield as a colorless liquid.

Column Chromatography Condition: 100 g Al_2O_3 + 6 g H_2O, 30 : 1 hexanes/EtOAc with 0.5% MeOH to 15 : 1 hexanes/EtOAc with 1.0% MeOH as gradient eluent.

^{1}H NMR (500 MHz, $CDCl_3$) δ: δ 7.32 – 7.22 (m, 4H), 7.20 – 7.13 (m, 3H), 7.10 (d, J = 7.8 Hz, 2H), 3.96 (m, 1H), 2.31 (s, 3H), 2.26 – 2.16 (m, 10H).

^{13}C NMR (125 MHz, $CDCl_3$) δ: 145.31, 142.04, 135.73, 129.28, 128.56, 127.90, 127.81, 126.17, 58.29, 48.79, 45.73, 33.86, 21.12.

HRMS (ESI-TOF) *m/z*: [M+H$^+$] calculated for $C_{18}H_{24}N$, 254.1909; found, 254.1917.

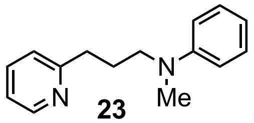

23

***N*-methyl-N-(3-(pyridin-2-yl)propyl)aniline (23, Fig 3):** Prepared according to General procedure C from 2-vinylpyridine with *N,N*-dimethylaniline for 40 hours in 61% isolated yield as a colorless liquid.

Column Chromatography Condition: 100 g Al_2O_3 + 8 g H_2O, 30 : 1 hexanes/EtOAc with 0.5% MeOH to 15 : 1 hexanes/EtOAc with 1.0% MeOH as gradient eluent.

^{1}H NMR (500 MHz, $CDCl_3$) δ: 8.58 – 8.47 (m, 1H), 7.58 (td, J = 7.7, 1.8 Hz, 1H), 7.24 – 7.18 (m, 2H), 7.14 (dt, J = 7.8, 1.1 Hz, 1H), 7.11 (ddd, J = 7.4, 4.9, 1.2 Hz, 1H), 6.81 – 6.51 (m, 3H), 3.39 (t, J = 7.6 Hz, 2H), 2.93 (s, 3H), 2.83 (dd, J = 8.9, 6.7 Hz, 2H), 2.04 (tt, J = 9.0, 6.8 Hz, 2H).

^{13}C NMR (125 MHz, $CDCl_3$) δ: 161.69, 149.45, 149.43, 136.45, 129.26, 122.85, 121.21, 116.14, 112.35, 52.45, 38.43, 35.93, 26.89.

HRMS (ESI-TOF) *m/z*: [M+H$^+$] calculated for $C_{15}H_{19}N_2$, 227.1548; found, 227.1543.

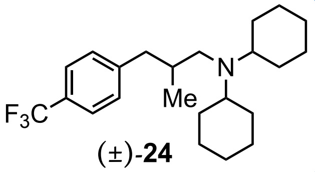

(±)-**24**

***N*-cyclohexyl-*N*-(2-methyl-3-(4-(trifluoromethyl)phenyl)propyl) cyclohexan-amine ((±)-24, Fig 3):** Prepared according to General procedure C from (*E*)-1-(prop-1-en-1-yl)-4-(trifluoromethyl)benzene with *N*-cyclohexyl-*N*-methylcyclohexanamine for 24 hours in 73% isolated yield as a colorless liquid.

Column Chromatography Condition: 100 g Al_2O_3 + 3 g H_2O, 99 : 1 hexanes/EtOAc with 0.5% MeOH as eluent.

^{1}H NMR (500 MHz, $CDCl_3$) δ: 7.52 (d, J = 8.0 Hz, 2H), 7.25 (d, J = 8.0 Hz, 2H), 3.09 (dd, J = 13.3, 4.0 Hz, 1H), 2.55 – 2.41 (m, 3H), 2.35 (dd, J = 13.4, 8.0 Hz, 1H), 2.07 (dd, J = 13.2, 9.9 Hz, 1H), 1.88 – 1.56 (m, 11H), 1.34 – 1.16 (m, 8H), 1.14 – 0.97 (m, 2H), 0.76 (d, J = 6.6 Hz, 3H).

^{13}C NMR (125 MHz, $CDCl_3$) δ: 147.03, 129.48, 127.89 (q, J = 32.2 Hz), 125.09 (q, J = 3.8 Hz), 124.65 (q, J = 271.5 Hz). 58.21, 52.94, 41.42, 35.59, 32.69, 32.16, 26.91, 26.86, 26.49, 17.75.

^{19}F NMR (471 MHz, CDC13) δ -62.21 (s).

HRMS (ESI-TOF) *m/z*: [M+H$^+$] calculated for $C_{23}H_{35}NF_3$, 382.2722; found, 382.2715.

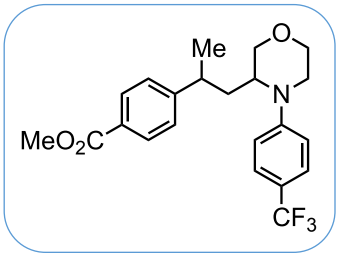

25

Methyl 4-(1-(4-(4-(trifluoromethyl)phenyl)morpholin-3-yl)propan-2-yl) benzo-ate (25, Fig 3): Prepared according to General procedure C from methyl 4-(prop-1-en-2-yl)benzoate with 4-(4-(trifluoromethyl)phenyl)morpholine for 16 hours in 62% isolated yield and 1.7:1 dr as a colorless liquid.

Column Chromatography Condition: silica, 6 : 1 hexanes/EtOAc as eluent.

^{1}H NMR (500 MHz, $CDCl_3$) δ: major diastereomer 8.00 (d, J = 8.2 Hz, 2H), 7.34 (d, J = 8.5 Hz, 2H), 7.18 (d, J = 8.2 Hz, 2H), 6.41 (d, J = 8.5 Hz, 2H), 4.01 (d, J = 11.8 Hz, 2H), 3.95 (s, 3H), 3.68 – 3.59 (m, 2H), 3.25 (dd, J = 8.3, 5.5 Hz, 1H), 3.22 – 3.16 (m, 2H), 2.84 – 2.74 (m, 1H), 2.28 (ddd, J = 13.5, 11.2, 3.9 Hz, 1H), 1.54 – 1.46 (m, 1H), 1.24 (d, J = 7.0 Hz, 3H); minor diastereomer 7.92 (d, J = 8.0 Hz, 2H), 7.44 (d, J = 8.5 Hz, 2H), 7.16 (d, J = 8.3 Hz, 2H), 6.73 (d, J = 8.5 Hz, 2H), 3.98 – 3.95 (m, 2H), 3.90 (s, 3H), 3.78 – 3.70 (m, 2H), 3.68 – 3.65 (m, 2H), 3.25 – 3.22 (m, 1H), 2.22 – 2.10 (m, 1H), 1.78 (ddd, J = 13.5, 7.8, 4.9 Hz, 1H), 1.54 – 1.51 (m, 1H), 1.26 (d, J = 8.4 Hz, 3H).

^{13}C NMR (125 MHz, $CDCl_3$) δ: major diastereomer 167.08, 151.41, 151.17, 130.14, 128.88, 127.39, 126.51 (q, J = 3.7 Hz), 124.87 (q, J = 270.3 Hz), 120.09 (q, J = 32.6 Hz), 113.68, 67.73, 66.85, 52.40, 52.27, 42.36, 37.51, 33.16, 23.49; minor diastereomer 167.12, 152.24, 151.90, 130.06, 128.43, 127.10, 126.73 (q, J = 3.7 Hz), 124.88 (q, J = 270.3 Hz), 120.05 (q, J = 32.9 Hz), 114.02, 68.86, 66.64, 53.33, 52.16, 42.36, 37.40, 34.59, 22.11.

^{19}F NMR (471 MHz, $CDCl_3$) δ -61.28.

HRMS (ESI-TOF) *m/z*: [M+H$^+$] calculated for $C_{22}H_{25}NO_3F_3$, 408.1787; found, 408.1785.

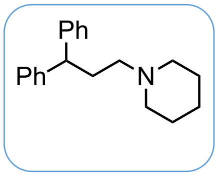

26

1-(3,3-diphenylpropyl)piperidine (26, Fig 4): Prepared according to General procedure D from 1,1-diphenylethylene with 1-((trimethylsilyl)methyl)piperidine for 2 hours in 90% isolated yield as a white solid. **m.p.** = 41–42 °C.

Column Chromatography Condition: 100 g Al_2O_3 + 4.5 g H_2O, 50 : 1 hexanes/EtOAc with 0.5% MeOH eluent.

^{1}H NMR (500 MHz, $CDCl_3$) δ: 7.30 – 7.21 (m, 8H), 7.20 – 7.13 (m, 2H), 3.95 (t, J = 7.0 Hz, 1H), 2.34 (br, 4H), 2.29 – 2.19 (m, 4H), 1.57 (p, J = 5.7 Hz, 4H), 1.47 – 1.35 (m, 2H).

^{13}C NMR (125 MHz, $CDCl_3$) δ: 145.13, 128.54, 128.00, 126.22, 57.98, 54.86, 49.55, 32.99, 26.20, 24.65.

HRMS (ESI-TOF) *m/z*: [M+H$^+$] calculated for $C_{20}H_{26}N$, 280.2065; found, 280.2065.

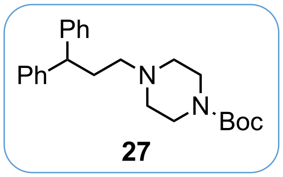

***tert*-butyl 4-(3,3-diphenylpropyl)piperazine-1-carboxylate (27, Fig 4):** Prepared according to General procedure D from 1,1-diphenylethylene with tert-butyl 4-((trimethylsilyl)methyl) piperazine-1-carboxylate for 2 hours in 95% isolated yield as a white solid. **m.p.** =110–113 °C.

Column Chromatography Condition: 100 g Al_2O_3 + 9 g H_2O, 30 : 1 hexanes/EtOAc with 0.5% MeOH to 15 : 1 hexanes/EtOAc with 1.0% MeOH as gradient eluent.

^{1}H NMR (500 MHz, $CDCl_3$) δ: 7.32 – 7.21 (m, 8H), 7.20 – 7.14 (m, 2H), 4.00 (t, J = 7.2 Hz, 1H), 3.53 – 3.33 (m, 4H), 2.40 – 2.30 (m, 4H), 2.30 – 2.18 (m, 4H), 1.45 (s, 9H).

^{13}C NMR (125 MHz, $CDCl_3$) δ: 154.88, 144.88, 128.60, 127.96, 126.33, 79.71, 56.96, 53.20, 49.14, 44.17, 32.79, 28.58.

HRMS (ESI-TOF) *m/z*: [M+H$^+$] calculated for $C_{24}H_{33}N_2O_2$, 381.2542; found, 381.2553.

28

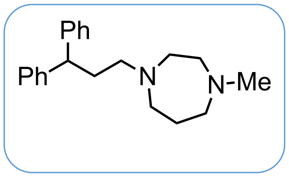

1-(3,3-diphenylpropyl)-4-methyl-1,4-diazepane (28, Fig 4): Prepared according to General procedure D from 1,1-diphenylethylene with 1-methyl-4-((trimethylsilyl)methyl)-1,4-diazepane for 2 hours in 89% isolated yield as a colorless liquid.

Column Chromatography Condition: 100 g Al_2O_3 + 9 g H_2O, 15 : 1 hexanes/EtOAc with 0.5% MeOH to 8 : 1 hexanes/EtOAc with 1.0% MeOH as gradient eluent.

^{1}H NMR (500 MHz, $CDCl_3$) δ: 7.32 – 7.22 (m, 8H), 7.21 – 7.13 (m, 2H), 4.02 (t, J = 7.7 Hz, 1H), 2.70 – 2.65 (m, 4H), 2.65 – 2.61 (m, 2H), 2.61 – 2.55 (m, 2H), 2.41 (dd, J = 8.6, 6.0 Hz, 2H), 2.35 (s, 3H), 2.21 (q, J = 7.5 Hz, 2H), 1.79 (p, J = 6.0 Hz, 2H).

^{13}C NMR (125 MHz, $CDCl_3$) δ: 145.16, 128.54, 128.03, 126.21, 58.34, 57.09, 56.87, 54.93, 54.42, 49.05, 47.26, 33.68, 27.65.

HRMS (ESI-TOF) *m/z*: [M+H$^+$] calculated for $C_{21}H_{29}N_2$, 309.2331; found, 309.2323.

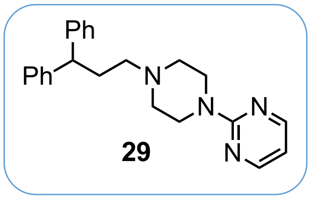

2-(4-(3,3-diphenylpropyl)piperazin-1-yl)pyrimidine (29, Fig 4): Prepared according to General procedure D from 1,1-diphenylethylene with 2-(4-((trimethylsilyl)methyl) piperazin-1-yl)pyrimidine for 2 hours in 91% isolated yield as a white solid. **m.p.** =111–112 °C.

Column Chromatography Condition: 100 g Al_2O_3 + 4.5 g H_2O, 15 : 1 hexanes/EtOAc with 0.5% MeOH to 8 : 1 hexanes/EtOAc with 1.0% MeOH as gradient eluent.

^{1}H NMR (500 MHz, $CDCl_3$) δ: 8.29 (d, J = 4.7 Hz, 2H), 7.31 – 7.26 (m, 7H), 7.26 – 7.24 (m, 1H), 7.21 – 7.15 (m, 2H), 6.47 (t, J = 4.7 Hz, 1H), 4.01 (t, J = 7.1 Hz, 1H), 3.82 (dd, J = 6.2, 4.0 Hz, 4H), 2.52 – 2.43 (m, 4H), 2.36 – 2.24 (m, 4H).

^{13}C NMR (125 MHz, $CDCl_3$) δ: 161.74, 157.83, 144.89, 128.60, 127.95, 126.33, 109.92, 57.13, 53.32, 49.23, 43.82, 32.86.

HRMS (ESI-TOF) *m/z*: [M+H$^+$] calculated for $C_{23}H_{27}N_4$, 359.2236; found, 359.2236.

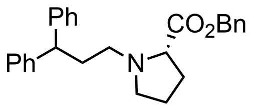

30

benzyl (3,3-diphenylpropyl)-L-prolinate (30, Fig 4): Prepared according to General procedure D from 1,1-diphenylethylene with benzyl ((trimethylsilyl)methyl)-L-prolinate for 2 hours in 88% isolated yield as a colorless liquid.

Column Chromatography Condition: 100 g Al_2O_3 + 8 g H_2O, 30 : 1 hexanes/EtOAc with 0.5% MeOH to 15 : 1 hexanes/EtOAc with 1.0% MeOH as gradient eluent.

^{1}H NMR (500 MHz, $CDCl_3$) δ: 7.37 – 7.32 (m, 3H), 7.31 – 7.27 (m, 5H), 7.26 (s, 1H), 7.25 – 7.22 (m, 4H), 7.20 – 7.15 (m, 2H), 5.11 (d, J = 12.4 Hz, 1H), 5.08 (d, J = 12.3 Hz, 1H), 3.97 (t, J = 7.7 Hz, 1H), 3.25 – 3.13 (m, 2H), 2.65 (dt, J = 11.7, 7.5 Hz, 1H), 2.45 – 2.32 (m, 2H), 2.27 (q, J = 7.7 Hz, 2H), 2.16 – 2.04 (m, 1H), 2.01 – 1.89 (m, 2H), 1.86 – 1.76 (m, 1H).

^{13}C NMR (125 MHz, $CDCl_3$) δ: 174.34, 144.94, 144.85, 136.03, 128.65, 128.55, 128.39, 128.32, 128.00, 127.87, 126.25, 126.23, 66.41, 66.05, 53.76, 53.55, 49.19, 34.63, 29.51, 23.41.

HRMS (ESI-TOF) *m/z*: [M+H$^+$] calculated for $C_{27}H_{30}N_4O_2$, 400.2277; found, 400.2278.

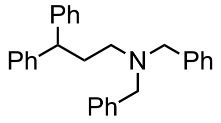

31

***N,N*-dibenzyl-3,3-diphenylpropan-1-amine (31, Fig 4):** Prepared according to General procedure D from 1,1-diphenylethylene with *N,N*-dibenzyl-1-(trimethylsilyl)methanamine for 2 hours in 95% isolated yield as a colorless liquid..

Column Chromatography Condition: 100 g Al_2O_3 + 4.5 g H_2O, 60 : 1 hexanes/EtOAc with 0.5% MeOH as eluent.

^{1}H NMR (500 MHz, $CDCl_3$) δ: 7.31 – 7.27 (m, 5H), 7.27 – 7.25 (m, 2H), 7.25 – 7.23 (m, 1H), 7.22 – 7.16 (m, 6H), 7.13 – 7.07 (m, 6H), 3.93 (t, J = 7.6 Hz, 1H), 3.53 (s, 4H), 2.41 (dd, J = 8.2, 6.0 Hz, 2H), 2.24 (td, J = 8.0, 6.0 Hz, 2H).

^{13}C NMR (125 MHz, $CDCl_3$) δ: 145.06, 139.81, 128.95, 128.48, 128.27, 127.93, 126.87, 126.10, 58.41, 51.73, 48.84, 33.32.

HRMS (ESI-TOF) *m/z*: [M+H$^+$] calculated for $C_{29}H_{30}N$, 392.2378; found, 392.2372.

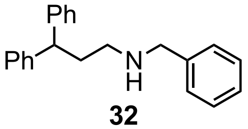

32

***N*-benzyl-3,3-diphenylpropan-1-amine (32, Fig 4):** Prepared according to General procedure D from 1,1-diphenylethylene with *N*-benzyl-1-(trimethylsilyl)methanamine for 2 hours in 85% isolated yield as a white solid. **m.p.** =57–59 °C.

Column Chromatography Condition: 100 g Al_2O_3 + 9 g H_2O, 15 : 1 hexanes/EtOAc with 0.5% MeOH to 8 : 1 hexanes/EtOAc with 1.0% MeOH as gradient eluent.

^{1}H NMR (500 MHz, $CDCl_3$) δ: 7.34 – 7.20 (m, 13H), 7.20 – 7.15 (m, 2H), 4.04 (t, J = 7.8 Hz, 1H), 3.73 (s, 2H), 2.62 (t, J = 7.2 Hz, 2H), 2.27 (q, J = 7.4 Hz, 2H), 1.37 (brs, 1H).

^{13}C NMR (125 MHz, $CDCl_3$) δ: 144.95, 140.56, 128.58, 128.49, 128.19, 127.97, 127.00, 126.29, 54.07, 49.15, 47.90, 36.02.

HRMS (ESI-TOF) *m/z*: [M+H$^+$] calculated for $C_{22}H_{24}N$, 302.1909; found, 302.1918.

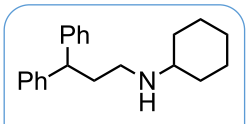

33

***N*-(3,3-diphenylpropyl)cyclohexanamine (33, Fig 4):** Prepared according to General procedure D from 1,1-diphenylethylene with *N*-((trimethylsilyl)methyl)cyclohexanamine for 10 hours in 80% isolated yield as a colorless liquid.

Column Chromatography Condition: 100 g Al_2O_3 + 9 g H_2O, 15 : 1 hexanes/EtOAc with 0.5% MeOH to 8 : 1 hexanes/EtOAc with 1.0% MeOH as gradient eluent.

^{1}H NMR (500 MHz, $CDCl_3$) δ: 7.32 – 7.22 (m, 8H), 7.21 – 7.13 (m, 2H), 3.99 (t, J = 7.8 Hz, 1H), 2.59 (dd, J = 8.4, 6.5 Hz, 2H), 2.35 (tt, J = 10.6, 3.8 Hz, 1H), 2.30 – 2.19 (m, 2H), 1.84 – 1.76 (m, 2H), 1.68 (dt, J = 13.0, 3.4 Hz, 2H), 1.63 – 1.52 (m, 1H), 1.25 – 1.08 (m, 3H), 1.06 – 0.94 (m, 2H).

^{13}C NMR (125 MHz, $CDCl_3$) δ: 144.87, 128.57, 127.89, 126.29, 56.88, 49.45, 45.46, 36.05, 33.44, 26.20, 25.16.

HRMS (ESI-TOF) *m/z*: [M+H$^+$] calculated for $C_{21}H_{28}N$, 294.2222; found, 294.2225.

34

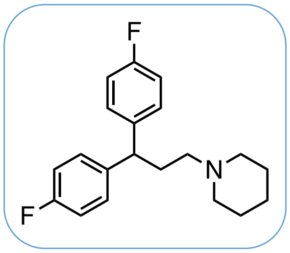

1-(3,3-bis(4-fluorophenyl)propyl)piperidine (34, Fig 4): Prepared according to General procedure D from 4,4'-(ethene-1,1-diyl)bis(fluorobenzene) with 1-((trimethylsilyl) methyl) piperidine for 2 hours in 84% isolated yield as a yellow liquid.

Column Chromatography Condition: 100 g Al_2O_3 + 4.5 g H_2O, 50 : 1 hexanes/EtOAc with 0.5% MeOH to 30 : 1 hexanes/EtOAc with 1.0% MeOH as gradient eluent.

^{1}H NMR (500 MHz, $CDCl_3$) δ: 7.20 – 7.13 (m, 4H), 6.99 – 6.92 (m, 4H), 3.94 (t, J = 7.5 Hz, 1H), 2.42 – 2.25 (m, 4H), 2.23 – 2.15 (m, 4H), 1.57 (p, J = 5.6 Hz, 4H), 1.47 – 1.35 (m, 2H).

^{13}C NMR (125 MHz, $CDCl_3$) δ: 161.46 (d, J = 244.4 Hz), 140.64 (d, J = 3.1 Hz), 129.26 (d, J = 7.8 Hz), 115.36 (d, J = 21.1 Hz), 57.64 , 54.86 , 47.81 , 33.26 , 26.19 , 24.61.

^{19}F NMR (471 MHz, $CDCl_3$) δ: -117.07 (m).

HRMS (ESI-TOF) *m/z*: [M+H$^+$] calculated for $C_{20}H_{24}NF_2$, 316.1877; found, 316.1870.

35

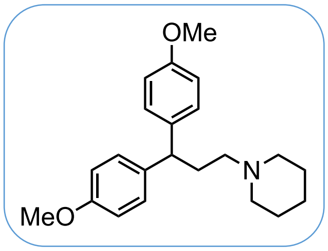

1-(3,3-bis(4-methoxyphenyl)propyl)piperidine (35, Fig 4): Prepared according to General procedure D from 4,4'-(ethene-1,1-diyl)bis(methoxybenzene) with 1-((trimethylsilyl) methyl)piperidine for 24 hours in 69% isolated yield as a light yellow liquid.

Column Chromatography Condition: 100 g Al_2O_3 + 9 g H_2O, 30 : 1 hexanes/EtOAc with 0.5% MeOH to 15 : 1 hexanes/EtOAc with 1.0% MeOH as gradient eluent.

^{1}H NMR (500 MHz, $CDCl_3$) δ: 7.13 (d, J = 8.5 Hz, 4H), 6.80 (d, J = 8.5 Hz, 4H), 3.85 (t, J = 7.0 Hz, 1H), 3.76 (s, 6H), 2.42 – 2.28 (m, 4H), 2.25 – 2.14 (m, 4H), 1.57 (p, J = 5.6 Hz, 4H), 1.47 – 1.36 (m, 2H).

^{13}C NMR (125 MHz, $CDCl_3$) δ: 157.92, 137.70, 128.76, 113.89, 58.08, 55.34, 54.89, 47.85, 33.34, 26.20, 24.66.

HRMS (ESI-TOF) *m/z*: [M+H$^+$] calculated for $C_{22}H_{30}NO_2$, 340.2277; found, 340.2272.

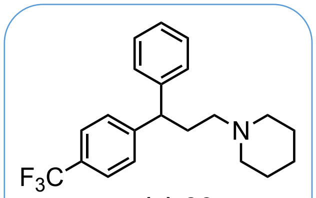

(±)-36

1-(3-phenyl-3-(4-(trifluoromethyl)phenyl)propyl)piperidine ((±)-36, Fig 4): Pre-pared according to General procedure D from 1-(1-phenylvinyl)-4-(trifluoromethyl) benzene with 1-((trimethylsilyl)methyl)piperidine for 2 hours in 80% isolated yield as a colorless liquid.

Column Chromatography Condition: 100 g Al_2O_3 + 4 g H_2O, 50 : 1 hexanes/EtOAc with 0.5% MeOH to 30 : 1 hexanes/EtOAc with 1.0% MeOH as gradient eluent.

^{1}H NMR (500 MHz, $CDCl_3$) δ: 7.52 (d, J = 8.1 Hz, 2H), 7.36 (d, J = 8.1 Hz, 2H), 7.32 – 7.27 (m, 2H), 7.25 – 7.16 (m, 3H), 4.04 (t, J = 7.3 Hz, 1H), 2.58 – 2.11 (m, 8H), 1.57 (p, J = 5.6 Hz, 4H), 1.46 – 1.36 (m, 2H).

^{13}C NMR (125 MHz, $CDCl_3$) δ: 149.18, 144.00, 128.74, 128.49 (q, J = 32.3 Hz), 128.29, 127.93, 126.63, 125.50 (q, J = 3.9 Hz), 124.37 (q, J = 272.0 Hz), 57.59, 54.80, 49.22, 32.68, 26.09, 24.55.

^{19}F NMR (471 MHz, $CDCl_3$) δ: -62.32 (s).

HRMS (ESI-TOF) *m/z*: [M+H$^+$] calculated for $C_{21}H_{25}NF_3$, 348.1939; found, 348.1928.

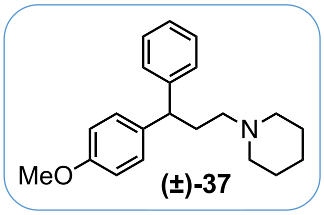

(±)-37

1-(3-(4-methoxyphenyl)-3-phenylpropyl)piperidine ((±)-37, Fig 4): Prepared according to General procedure D from 1-methoxy-4-(1-phenylvinyl)benzene with 1-((trimethylsilyl) methyl)piperidine for 8 hours in 85% isolated yield as a white solid. **m.p.** =54–55 °C.

Column Chromatography Condition: 100 g Al_2O_3 + 7 g H_2O, 30 : 1 hexanes/EtOAc with 0.5% MeOH to 15 : 1 hexanes/EtOAc with 1.0% MeOH as gradient eluent

^{1}H NMR (500 MHz, $CDCl_3$) δ: 7.30 – 7.19 (m, 4H), 7.19 – 7.12 (m, 3H), 6.85 – 6.77 (m, 2H), 3.90 (t, J = 3.7 Hz, 1H), 3.77 (s, 3H), 2.44 – 2.26 (m, 3H), 2.26 – 2.19 (m, 4H), 1.57 (p, J = 5.6 Hz, 4H), 1.46 – 1.35 (m, 2H), 1.28 – 1.26 (m, 1H).

^{13}C NMR (125 MHz, $CDCl_3$) δ: 157.92, 145.49, 137.23, 128.84, 128.51, 127.84, 126.11, 113.86, 58.06, 55.32, 54.87, 48.69, 33.14, 26.17, 24.62.

HRMS (ESI-TOF) *m/z*: [M+H$^+$] calculated for $C_{21}H_{28}NO$, 310.2171; found, 310.2162.

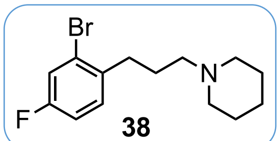

38

1-(3-(2-bromo-4-fluorophenyl)propyl)piperidine (38, Fig 4): Prepared according to General procedure D from 2-bromo-4-fluoro-1-vinylbenzene with 1-((trimethylsilyl)methyl) piperidine for 3 hours in 82% isolated yield as a colorless liquid.

Column Chromatography Condition: 100 g Al_2O_3 + 3 g H_2O, 50 : 1 hexanes/EtOAc with 0.5% MeOH to 30 : 1 hexanes/EtOAc with 1.0% MeOH as gradient eluent.

^{1}H NMR (500 MHz, $CDCl_3$) δ: 7.26 (dd, J = 8.3, 2.7 Hz, 1H), 7.18 (dd, J = 8.5, 6.1 Hz, 1H), 6.94 (td, J = 8.3, 2.7 Hz, 1H), 2.74 – 2.65 (m, 2H), 2.46 – 2.24 (m, 6H), 1.78 (tt, J = 9.7, 6.6 Hz, 2H), 1.58 (p, J = 5.7 Hz, 4H), 1.48 – 1.36 (m, 2H).

^{13}C NMR (125 MHz, $CDCl_3$) δ: 160.78 (d, J = 248.0 Hz), 137.54 (d, J = 3.4 Hz), 131.02 (d, J = 8.2 Hz), 124.16 (d, J = 9.5 Hz), 119.87 (d, J = 24.2 Hz), 114.49 (d, J = 20.7 Hz), 58.79, 54.73, 33.45, 27.37, 26.16, 24.63.

^{19}F NMR (471 MHz, $CDCl_3$) δ: -115.57 (q, J = 7.7 Hz).

HRMS (ESI-TOF) *m/z*: [M+H$^+$] calculated for $C_{14}H_{20}NBrF$, 300.0763; found, 300.0769.

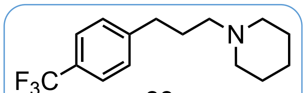

39

1-(3-(4-(trifluoromethyl)phenyl)propyl)piperidine (39, Fig 4): Prepared according to General procedure D from 1-(trifluoromethyl)-4-vinylbenzene with 1-((trimethylsilyl)methyl) piperidine for 2 hours in 84% isolated yield as a yellow liquid.

Column Chromatography Condition: 100 g Al_2O_3 + 3 g H_2O, 50 : 1 hexanes/EtOAc with 0.5% MeOH to 30 : 1 hexanes/EtOAc with 1.0% MeOH as gradient eluent.

^{1}H NMR (500 MHz, $CDCl_3$) δ: 7.52 (d, J = 8.0 Hz, 2H), 7.29 (d, J = 8.0 Hz, 2H), 2.67 (t, J = 7.8 Hz, 2H), 2.46 – 2.23 (m, 6H), 1.83 (tt, J = 9.7, 6.7 Hz, 2H), 1.58 (p, J = 5.6 Hz, 4H), 1.47 – 1.37 (m, 2H).

^{13}C NMR (125 MHz, $CDCl_3$) δ: 146.54 (q, J = 1.3 Hz), 128.81, 128.18 (q, J = 32.3 Hz), 125.32 (q, J = 3.8 Hz), 124.50 (q, J = 271.7 Hz), 58.71, 54.68, 33.81, 28.51, 26.08, 24.56.

^{19}F NMR (471 MHz, $CDCl_3$) δ: -62.25 (s).

HRMS (ESI-TOF) *m/z*: [M+H$^+$] calculated for $C_{15}H_{21}NF_3$, 272.1626; found, 272.1628.

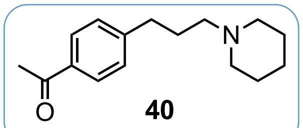

40

1-(4-(3-(piperidin-1-yl)propyl)phenyl)ethan-1-one (40, Fig 4): Prepared according to General procedure D from 1-(4-vinylphenyl)ethan-1-one with 1-((trimethylsilyl)methyl) piperidine for 3 hours in 83% isolated yield as a colorless liquid.

Column Chromatography Condition: 100 g Al_2O_3 + 9 g H_2O, 15 : 1 hexanes/EtOAc with 0.5% MeOH to 8 : 1 hexanes/EtOAc with 1.0% MeOH as gradient eluent.

^{1}H NMR (500 MHz, $CDCl_3$) δ: 7.87 (d, J = 8.2 Hz, 2H), 7.27 (d, J = 8.0 Hz, 2H), 2.74 – 2.62 (m, 2H), 2.58 (s, 3H), 2.44 – 2.24 (m, 6H), 1.83 (tt, J = 9.7, 6.6 Hz, 2H), 1.57 (p, J = 5.6 Hz, 4H), 1.47 – 1.38 (m, 2H).

^{13}C NMR (125 MHz, $CDCl_3$) δ: 198.06, 148.35, 135.07, 128.74, 128.62, 58.82, 54.73, 34.01, 28.48, 26.74, 26.12, 24.58.

HRMS (ESI-TOF) *m/z*: [M+H$^+$] calculated for $C_{16}H_{24}NO$, 246.1858; found, 246.1851.

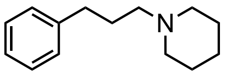

41

1-(3-phenylpropyl)piperidine (41, Fig 4): Prepared according to General procedure D from styrene with 1-((trimethylsilyl)methyl) piperidine for 24 hours in 63% isolated yield as a colorless liquid.

Column Chromatography Condition: 100 g Al_2O_3 + 6 g H_2O, 60 : 1 hexanes/EtOAc with 0.5% MeOH to 30 : 1 hexanes/EtOAc with 1.0% MeOH as gradient eluent.

^{1}H NMR (500 MHz, $CDCl_3$) δ: 7.27 (t, J = 7.7 Hz, 2H), 7.23 – 7.14 (m, 3H), 2.62 (t, J = 7.8 Hz, 2H), 2.45 – 2.27 (m, 6H), 1.83 (tt, J = 10.1, 6.6 Hz, 2H), 1.58 (p, J = 5.7 Hz, 4H), 1.48 – 1.38 (m, 2H).

^{13}C NMR (125 MHz, $CDCl_3$) δ: 142.58, 128.62, 128.50, 125.92, 59.22, 54.87, 34.19, 28.96, 26.29, 24.77.

HRMS (ESI-TOF) *m/z*: [M+H$^+$] calculated for $C_{14}H_{22}N$, 204.1752; found, 204.1762.

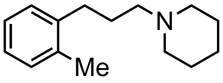

42

1-(3-(o-tolyl)propyl)piperidine (42, Fig 4): Prepared according to General procedure D from 1-methyl-2-vinylbenzene with 1-((trimethylsilyl)methyl) piperidine for 24 hours in 50% isolated yield as a colorless liquid.

Column Chromatography Condition: 100 g Al_2O_3 + 4 g H_2O, 60 : 1 hexanes/EtOAc with 0.5% MeOH to 30 : 1 hexanes/EtOAc with 1.0% MeOH as gradient eluent.

^{1}H NMR (500 MHz, $CDCl_3$) δ: 7.18 – 7.03 (m, 4H), 2.60 (t, J = 7.8 Hz, 2H), 2.45 – 2.33 (m, 6H), 2.31 (s, 3H), 1.85 – 1.72 (m, 2H), 1.59 (p, J = 5.7 Hz, 4H), 1.44 (q, J = 6.0 Hz, 2H).

^{13}C NMR (125 MHz, $CDCl_3$) δ: 140.67, 136.01, 130.23, 128.91, 125.99, 125.95, 59.39, 54.79, 31.36, 27.68, 26.20, 24.68, 19.43.

HRMS (ESI-TOF) *m/z*: [M+H$^+$] calculated for $C_{15}H_{24}N$, 218.1909; found, 218.1917.

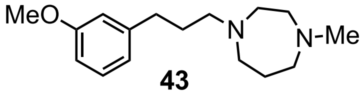

43

1-(3-(3-methoxyphenyl)propyl)-4-methyl-1,4-diazepane (43, Fig 4): Prepared according to General procedure D from 1-methoxy-3-vinylbenzene with 1-methyl-4-((trimethylsilyl)methyl)-1,4-diazepane for 24 hours in 57% isolated yield as a colorless liquid.

Column Chromatography Condition: 100 g Al_2O_3 + 9 g H_2O, 10 : 1 hexanes/EtOAc with 0.5% MeOH to 5 : 1 hexanes/EtOAc with 1.0% MeOH as gradient eluent.

^{1}H NMR (500 MHz, $CDCl_3$) δ: 7.21 (t, J = 7.8 Hz, 1H), 6.80 (dt, J = 7.6, 1.2 Hz, 1H), 6.78 – 6.71 (m, 2H), 3.81 (s, 3H), 2.77 – 2.69 (m, 4H), 2.67 – 2.59 (m, 6H), 2.55 – 2.48 (m, 2H), 2.37 (s, 3H), 1.87 – 1.73 (m, 4H).

^{13}C NMR (125 MHz, $CDCl_3$) δ: 159.70, 144.19, 129.34, 120.97, 114.29, 111.09, 58.23, 58.05, 57.12, 55.25, 54.87, 54.34, 47.28, 33.82, 29.39, 27.58.

HRMS (ESI-TOF) *m/z*: [M+H$^+$] calculated for $C_{16}H_{27}N_2O$, 263.2123; found, 263.2126.

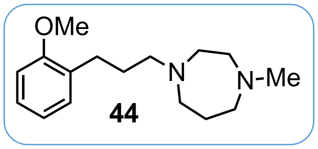

1-(3-(2-methoxyphenyl)propyl)-4-methyl-1,4-diazepane (44, Fig 4): Prepared according to General procedure D from 1-methoxy-2-vinylbenzene with 1-methyl-4-((trimethylsilyl) methyl)-1,4-diazepane for 24 hours in 31% isolated yield as a colorless liquid.

Column Chromatography Condition: 100 g Al_2O_3 + 9 g H_2O, 10 : 1 hexanes/EtOAc with 0.5% MeOH to 5 : 1 hexanes/EtOAc with 1.0% MeOH as gradient eluent.

^{1}H NMR (500 MHz, $CDCl_3$) δ: 7.16 (td, J = 7.8, 1.8 Hz, 1H), 7.13 (dd, J = 7.4, 1.8 Hz, 1H), 6.87 (td, J = 7.4, 1.2 Hz, 1H), 6.83 (dd, J = 8.1, 1.1 Hz, 1H), 3.81 (s, 3H), 2.75 – 2.67 (m, 4H), 2.65 – 2.57 (m, 6H), 2.54 – 2.47 (m, 2H), 2.35 (s, 3H), 1.90 – 1.65 (m, 4H).

^{13}C NMR (125 MHz, $CDCl_3$) δ: 157.58, 130.95, 129.95, 127.02, 120.43, 110.32, 58.47, 58.25, 57.17, 55.36, 54.85, 54.36, 47.28, 28.25, 27.81, 27.59.

HRMS (ESI-TOF) *m/z*: [M+H$^+$] calculated for $C_{16}H_{27}N_2O$, 263.2123; found, 263.2126.

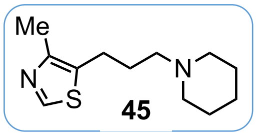

4-methyl-5-(3-(piperidin-1-yl)propyl)thiazole (45, Fig 4): Prepared according to General procedure D from 4-methyl-5-vinylthiazole with 1-((trimethylsilyl)methyl)piperidine for 4 hours in 47% isolated yield as a colorless liquid.

Column Chromatography Condition: 100 g Al_2O_3 + 3 g H_2O, 30 : 1 hexanes/EtOAc with 0.5% MeOH to 15 : 1 hexanes/EtOAc with 1.0% MeOH as gradient eluent.

^{1}H NMR (500 MHz, $CDCl_3$) δ: 8.53 (s, 1H), 2.76 (t, J = 7.6 Hz, 2H), 2.37 (s, 3H), 2.35 – 2.24 (m, 6H), 1.87 – 1.72 (m, 2H), 1.56 (p, J = 5.7 Hz, 4H), 1.47 – 1.36 (m, 2H).

^{13}C NMR (125 MHz, $CDCl_3$) δ: 149.00, 148.60, 131.77, 58.25, 54.71, 29.01, 26.13, 24.59, 24.19, 15.00.

HRMS (ESI-TOF) *m/z*: [M+H$^+$] calculated for $C_{12}H_{21}N_2S$, 225.1425; found, 225.1420.

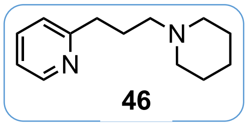

2-(3-(piperidin-1-yl)propyl)pyridine (46, Fig 4): Prepared according to General procedure D from 2-vinylpyridine with 1-((trimethylsilyl)methyl)piperidine for 2 hours in 94% isolated yield as a yellow liquid.

Column Chromatography Condition: 100 g Al_2O_3 + 7 g H_2O, 8 : 1 hexanes/EtOAc with 0.5% MeOH to 4 : 1 hexanes/EtOAc with 1.0% MeOH as gradient eluent.

^{1}H NMR (500 MHz, $CDCl_3$) δ: δ 8.63 – 8.36 (m, 1H), 7.57 (td, J = 7.6, 1.9 Hz, 1H), 7.14 (d, J = 7.8 Hz, 1H), 7.08 (dd, J = 7.5, 4.8 Hz, 1H), 2.85 – 2.71 (m, 2H), 2.49 – 2.24 (m, 6H), 2.00 – 1.84 (m, 2H), 1.56 (p, J = 5.7 Hz, 4H), 1.47 – 1.34 (m, 2H).

^{13}C NMR (125 MHz, $CDCl_3$) δ: 162.16, 149.31, 136.36, 122.85, 121.05, 58.98, 54.66, 36.54, 27.17, 26.10, 24.60.

HRMS (ESI-TOF) *m/z*: [M+H$^+$] calculated for $C_{13}H_{21}N_2$, 205.1705; found, 205.1695.

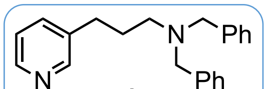

47

***N,N*-dibenzyl-3-(pyridin-3-yl)propan-1-amine (47, Fig 4):** Prepared according to General procedure D from 3-vinylpyridine with *N,N*-dibenzyl-1-(trimethylsilyl)methanamine for 7 hours in 91% isolated yield as a light yellow liquid.

Column Chromatography Condition: 100 g Al_2O_3 + 9 g H_2O, 30 : 1 hexanes/EtOAc with 0.5% MeOH to 15 : 1 hexanes/EtOAc with 1.0% MeOH as gradient eluent.

^{1}H NMR (500 MHz, $CDCl_3$) δ: 8.40 (dd, J = 4.9, 1.7 Hz, 1H), 8.35 (d, J = 2.3 Hz, 1H), 7.36 (d, J = 7.1 Hz, 4H), 7.34 – 7.29 (m, 5H), 7.28 – 7.21 (m, 2H), 7.12 (dd, J = 7.8, 4.8 Hz, 1H), 3.57 (s, 4H), 2.58 (dd, J = 8.8, 6.8 Hz, 2H), 2.48 (t, J = 6.9 Hz, 2H), 1.90 – 1.67 (m, 2H).

^{13}C NMR (125 MHz, $CDCl_3$) δ: 150.09, 147.31, 139.85, 137.80, 135.84, 128.96, 128.35, 127.01, 123.28, 58.68, 52.88, 30.68, 28.92.

HRMS (ESI-TOF) *m/z*: [M+H$^+$] calculated for $C_{22}H_{25}N_2$, 317.2018; found, 317.2004.

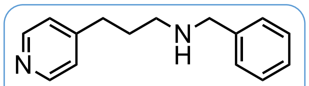

48

***N*-benzyl-3-(pyridin-4-yl)propan-1-amine (48, Fig 4):** Prepared according to General procedure D from 4-vinylpyridine with *N*-benzyl-1-(trimethylsilyl)methanamine for 3 hours in 91% isolated yield as a colorless liquid.

Column Chromatography Condition: 100 g Al_2O_3 + 9 g H_2O, 8 : 1 hexanes/EtOAc with 1.0% MeOH to 4 : 1 hexanes/EtOAc with 2.0% MeOH as gradient eluent.

^{1}H NMR (500 MHz, $CDCl_3$) δ: 8.47 (d, J = 6.1 Hz, 2H), 7.39 – 7.19 (m, 5H), 7.09 (d, J = 6.1 Hz, 2H), 3.78 (s, 2H), 2.67 (m, 4H), 1.88 – 1.79 (m, 2H), 1.30 (brs, 1H).

^{13}C NMR (125 MHz, $CDCl_3$) δ: 151.21, 149.85, 140.51, 128.54, 128.21, 127.09, 123.97, 54.13, 48.68, 33.00, 30.76.

HRMS (ESI-TOF) *m/z*: [M+H$^+$] calculated for $C_{15}H_{19}N_2$, 227.1548; found, 227.1547.

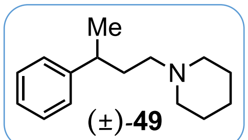

(±)-49

1-(3-phenylbutyl)piperidine ((±)-49, Fig 4): Prepared according to General procedure D from α-methyl styrene with 1.5 equiv 1-((trimethylsilyl)methyl)piperidine and 1.0 equiv BHT as hydrogen donor for 24 hours in 57% isolated yield as a colorless liquid.

Column Chromatography Condition: 100 g Al_2O_3 + 6 g H_2O, 60 : 1 hexanes/EtOAc with 1.0% MeOH to 30 : 1 hexanes/EtOAc with 2.0% MeOH as gradient eluent.

^{1}H NMR (500 MHz, $CDCl_3$) δ: 7.28 (dd, J = 8.2, 6.9 Hz, 2H), 7.22 – 7.13 (m, 3H), 2.71 (h, J = 7.0 Hz, 1H), 2.31 (brs, 4H), 2.25 (ddd, J = 12.3, 10.1, 5.9 Hz, 1H), 2.14 (ddd, J = 12.2, 10.1, 5.5 Hz, 1H), 1.89 – 1.70 (m, 2H), 1.56 (p, J = 5.7 Hz, 4H), 1.40 (p, J = 6.3 Hz, 2H), 1.25 (d, J = 7.0 Hz, 3H).

^{13}C NMR (125 MHz, $CDCl_3$) δ: 147.60, 128.44, 127.09, 126.00, 57.98, 54.85, 38.60, 35.58, 26.21, 24.68, 22.68.

HRMS (ESI-TOF) *m/z*: [M+H$^+$] calculated for $C_{15}H_{24}N$, 218.1909; found, 218.1904.

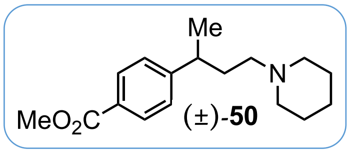

Methyl 4-(4-(piperidin-1-yl)butan-2-yl)benzoate ((±)-50, Fig 4): Prepared according to General procedure D from methyl 4-(prop-1-en-2-yl)benzoate with 1-((trimethylsilyl) methyl)piperidine for 4 hours in 80% isolated yield as a colorless liquid.

Column Chromatography Condition: 100 g Al_2O_3 + 9 g H_2O, 30 : 1 hexanes/EtOAc with 1.0% MeOH to 15 : 1 hexanes/EtOAc with 2.0% MeOH as gradient eluent.

^{1}H NMR (500 MHz, $CDCl_3$) δ: δ 7.97 (d, J = 8.2 Hz, 2H), 7.27 (d, J = 8.3 Hz, 2H), 3.92 (s, 3H), 2.81 (h, J = 7.1 Hz, 1H), 2.39 – 2.23 (m, 4H), 2.28 – 2.20 (m, 1H), 2.18 – 2.07 (m, 1H), 1.82 (q, J = 7.6 Hz, 2H), 1.58 (p, J = 5.7 Hz, 4H), 1.47 – 1.37 (m, 2H), 1.28 (d, J = 6.9 Hz, 3H).

^{13}C NMR (125 MHz, $CDCl_3$) δ: 167.31, 153.12, 129.88, 128.07, 127.18, 57.71, 54.83, 52.10, 38.63, 35.38, 26.18, 24.64, 22.38.

HRMS (ESI-TOF) *m/z*: [M+H$^+$] calculated for $C_{17}H_{26}NO_2$, 276.1964; found, 276.1963.

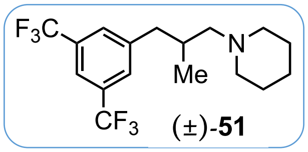

1-(3-(3,5-bis(trifluoromethyl)phenyl)-2-methylpropyl)piperidine ((±)-51, Fig 4): Prepared according to General procedure D from (*E*)-1-(prop-1-en-1-yl)-3,5-bis (trifluoro methyl)benzene with *1*-((trimethylsilyl)methyl)piperidine for 24 hours in 72% isolated yield as a colorless liquid.

Column Chromatography Condition: 100 g Al_2O_3 + 3 g H_2O, 99 : 1 hexanes/EtOAc with 0.5% MeOH as eluent.

^{1}H NMR (500 MHz, $CDCl_3$) δ: 7.70 (s, 1H), 7.64 (s, 2H), 2.88 (dd, J = 13.4, 3.4 Hz, 1H), 2.67 – 2.50 (m, 1H), 2.44 -2.18 (m, 4H), 2.12 – 1.99 (m, 3H), 1.65 – 1.48 (m, 4H), 1.47 – 1.37 (m, 2H), 0.82 (d, J = 5.6 Hz, 3H).

^{13}C NMR (125 MHz, $CDCl_3$) δ: 143.59, 131.21 (q, J = 32.9 Hz), 129.79 (m), 123.70 (q, J = 272.5 Hz), 119.79 (sept, J = 3.8 Hz), 64.98, 55.04, 40.51, 31.97, 26.23, 24.72, 17.90.

^{19}F NMR (471 MHz, $CDCl_3$) δ: -62.82 (s).

HRMS (ESI-TOF) *m/z*: [M+H$^+$] calculated for $C_{17}H_{22}NF_6$, 354.1656; found, 356.1652.

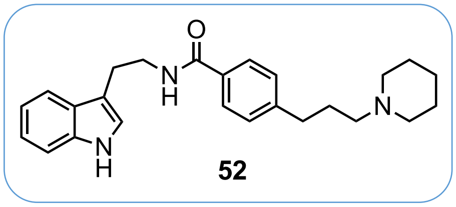

***N*-(2-(1H-indol-3-yl)ethyl)-4-(3-(piperidin-1-yl)propyl)benzamide (52, Fig 4):** Prepared according to General procedure D from *N*-(2-(1H-indol-3-yl)ethyl)-4-vinylbenzamide with 1-((trimethylsilyl)methyl)piperidine for 3 hours in 85% isolated yield as a light yellow solid. **m.p.** = 131–132 °C.

Column Chromatography Condition: 100 g Al_2O_3 + 9 g H_2O, 2 : 1 hexanes/EtOAc with 0.5% MeOH to 1 : 1 hexanes/EtOAc with 1.0% MeOH as gradient eluent.

^{1}H NMR (500 MHz, $CDCl_3$) δ: 8.19 (brs, 1H), 7.65 (d, J = 7.9 Hz, 1H), 7.58 (d, J = 8.0 Hz, 2H), 7.41 – 7.36 (m, 1H), 7.24 – 7.20 (m, 1H), 7.18 (d, J = 8.1 Hz, 2H), 7.16 – 7.10 (m, 1H), 7.08 – 7.05 (m, 1H), 6.18 (t, J = 5.8 Hz, 1H), 3.80 (q, J = 6.4 Hz, 2H), 3.09 (t, J = 6.7 Hz, 2H), 2.63 (t, J = 7.7 Hz, 2H), 2.35 (s, 4H), 2.32 – 2.25 (m, 2H), 1.80 (dq, J = 12.8, 7.6 Hz, 2H), 1.57 (p, J = 5.6 Hz, 4H), 1.47 – 1.36 (m, 2H).

^{13}C NMR (125 MHz, $CDCl_3$) δ: 167.51, 146.23, 136.60, 132.35, 128.68, 127.50, 127.02, 122.41, 122.24, 119.70, 118.94, 113.27, 111.41, 58.83, 54.77, 40.34, 33.85, 28.55, 26.18, 25.51, 24.64.

HRMS (ESI-TOF) *m/z*: [M+H$^+$] calculated for $C_{25}H_{32}N_3O$, 390.2540; found, 390.2540.

53

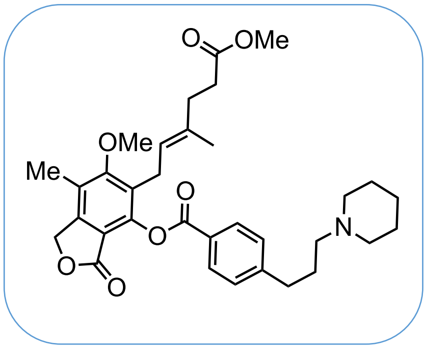

mycophenolic acid derivative **(53, Fig 4):** Prepared according to General procedure D from the corresponding alkene with 1-((trimethylsilyl)methyl)piperidine for 5 hours in 75% isolated yield as a white solid. **m.p.** = 128–130 °C.

Column Chromatography Condition: 100 g Al_2O_3 + 6 g H_2O, 5 : 1 hexanes/EtOAc with 0.5% MeOH to 3 : 1 hexanes/EtOAc with 1.0% MeOH as gradient eluent.

^{1}H NMR (500 MHz, $CDCl_3$) δ: 8.11 (d, J = 7.9 Hz, 2H), 7.32 (d, J = 7.9 Hz, 2H), 5.16 (s, 2H), 5.14 (t, J = 6.4 Hz, 1H), 3.81 (s, 3H), 3.60 (s, 3H), 3.53 – 3.27 (m, 2H), 2.71 (t, J = 7.7 Hz, 2H), 2.45 – 2.27 (m, 8H), 2.25 (s, 3H), 2.23 – 2.15 (m, 2H), 1.86 (p, J = 7.7 Hz, 2H), 1.66 – 1.53 (m, 7H), 1.49 – 1.37 (m, 2H).

^{13}C NMR (125 MHz, $CDCl_3$) δ: 173.82, 168.14, 164.64, 162.74, 149.08, 146.40, 146.31, 134.68, 130.67, 129.61, 128.80, 126.51, 123.07, 122.38, 114.07, 68.40, 61.36, 58.82, 54.77, 51.61, 34.53, 34.18, 32.76, 28.48, 26.21, 24.66, 23.87, 16.35, 11.95.

HRMS (ESI-TOF) *m/z*: [M+H$^+$] calculated for $C_{33}H_{42}NO_7$, 564.2961; found, 564.2961.

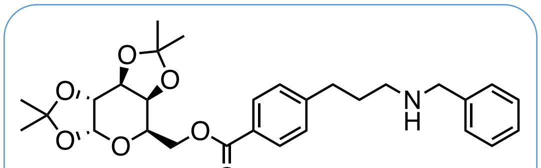

54

α-D-galactopyranose derivative **(54, Fig 4):** Prepared according to General procedure D from the corresponding alkene with *N*-benzyl-1-(trimethylsilyl) methanamine for 4 hours in 87% isolated yield as a light yellow liquid.

Column Chromatography Condition: 100 g Al_2O_3 + 6 g H_2O, 5 : 1 hexanes/EtOAc with 0.5% MeOH to 2.5 : 1 hexanes/EtOAc with 1.0% MeOH as gradient eluent.

^{1}H NMR (500 MHz, $CDCl_3$) δ: 7.96 (d, J = 8.2 Hz, 2H), 7.37 – 7.29 (m, 4H), 7.26 – 7.24 (m, 1H), 7.23 (d, J = 8.0 Hz, 2H), 5.57 (d, J = 4.9 Hz, 1H), 4.65 (dd, J = 7.9, 2.5 Hz, 1H), 4.52 (dd, J = 11.5, 5.0 Hz, 1H), 4.41 (dd, J = 11.5, 7.5 Hz, 1H), 4.37 – 4.30 (m, 2H), 4.18 (ddd, J = 7.2, 5.1, 1.9 Hz, 1H), 3.78 (s, 2H), 2.72 (t, J = 7.7 Hz, 2H), 2.66 (t, J = 7.1 Hz, 2H), 1.84 (p, J = 7.3 Hz, 2H), 1.52 (s, 3H), 1.48 (s, 3H), 1.37 – 1.27 (brs, 1H), 1.36 (s, 3H), 1.33 (s, 3H).

^{13}C NMR (125 MHz, $CDCl_3$) δ: 166.56, 147.98, 140.58, 129.97, 128.54, 128.52, 128.23, 127.84, 127.05, 109.80, 108.92, 96.47, 71.29, 70.88, 70.70, 66.30, 63.84, 54.16, 48.85, 33.79, 31.56, 26.18, 26.13, 25.13, 24.65.

HRMS (ESI-TOF) *m/z*: [M+H$^+$] calculated for $C_{29}H_{38}NO_7$, 512.2648; found, 512.2650.

Gram-scale synthesis of biologically active compounds

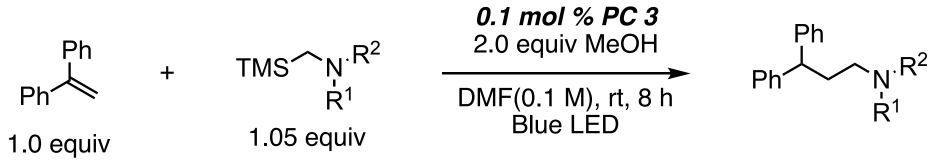

Procedure: [Ir(ppy)$_2$(dtbbpy)]PF$_6$ (**PC 3**, 3.0 mg, 0.003 mmol, 0.1 mol %), alkene (3.0 mmol, 1.0 equiv), α-TMS amine (3.15 mmol, 1.05 equiv), and DMF (30 mL) were added to a 50 mL round-bottom flask equipped with a stir bar in the glove box under nitrogen atmosphere. The flask was then taken out of glove box and injected 0.24 mL MeOH (6.0 mmol, 2.0 equiv). The resulting solution was allowed to stir inside the photo-reactor (described above) with the lamp and fan on for 8 hours. The reaction crude was quenched by the addition of water, followed by the extraction with EtOAc three times. The combined organic layers were washed with brine and water, dried over $MgSO_4$, concentrated *in vacuo* and then purified by basic alumina chromatography to afford the desired product.

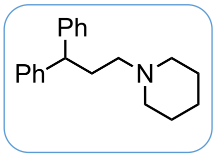

1-(3,3-diphenylpropyl)piperidine (Fenpiprane, Fig 5): Prepared according to previously described procedure from 1,1-diphenylethylene with 1-((trimethylsilyl)methyl)piperidine in 87% isolated yield as a white solid. **m.p.** =41–42 °C.

Column Chromatography Condition: 150 g Al_2O_3 + 6.8 g H_2O, 50 : 1 hexanes/EtOAc with 0.5% MeOH as eluent.

^{1}H NMR (500 MHz, $CDCl_3$) δ: 7.30 – 7.21 (m, 8H), 7.20 – 7.13 (m, 2H), 3.95 (t, J = 7.0 Hz, 1H), 2.34 (br, 4H), 2.29 – 2.19 (m, 4H), 1.57 (p, J = 5.7 Hz, 4H), 1.47 – 1.35 (m, 2H).

^{13}C NMR (125 MHz, $CDCl_3$) δ: 145.13, 128.54, 128.00, 126.22, 57.98, 54.86, 49.55, 32.99, 26.20, 24.65.

HRMS (ESI-TOF) *m/z*: [M+H$^+$] calculated for $C_{20}H_{26}N$, 280.2065; found, 280.2065.

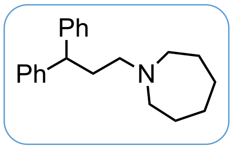

1-(3,3-diphenylpropyl)azepane (Prozapine, Fig 5): Prepared according to previously described procedure from 1,1-diphenylethylene with 1-((trimethylsilyl)methyl)azepane in 84% isolated yield as a colorless liquid.

Column Chromatography Condition: 150 g Al_2O_3 + 7.5 g H_2O, 60 : 1 hexanes/EtOAc with 0.5% MeOH to 30 : 1 hexanes/EtOAc with 1.0% MeOH as gradient eluent.

^{1}H NMR (500 MHz, $CDCl_3$) δ: 7.31 – 7.22 (m, 8H), 7.19 – 7.13 (m, 2H), 4.03 (t, J = 7.7 Hz, 1H), 2.67 – 2.51 (m, 4H), 2.40 (dd, J = 8.7, 6.0 Hz, 2H), 2.22 (q, J = 7.5 Hz, 2H), 1.70 – 1.53 (m, 8H).

^{13}C NMR (125 MHz, $CDCl_3$) δ: 145.28, 128.52, 128.06, 126.17, 56.56, 55.72, 49.16, 33.78, 28.40, 27.18.

HRMS (ESI-TOF) *m/z*: [M+H$^+$] calculated for $C_{21}H_{28}N$, 294.2222; found, 294.2224.

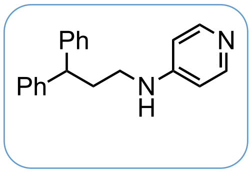

***N*-(3,3-diphenylpropyl)pyridin-4-amine (Phenpyramine, Fig 5):** Prepared according to previously described procedure from 1,1-diphenylethylene with *N*-((trimethylsilyl)methyl)pyridin-4-aminefor 24 hours in 87% isolated yield as a white solid. **m.p.** = 123–124 °C.

Column Chromatography Condition: 150 g Al_2O_3 + 13.5 g H_2O, 3 : 1 hexanes/EtOAc with 0.5% MeOH to 1.5 : 1 hexanes/EtOAc with 1.0% MeOH as gradient eluent.

^{1}H NMR (500 MHz, $CDCl_3$) δ: 8.21 – 8.10 (m, 2H), 7.30 (t, J = 7.6 Hz, 4H), 7.28 – 7.22 (m, 4H), 7.23 – 7.18 (m, 2H), 6.33 – 6.26 (m, 2H), 4.12 – 4.05 (brs, 1H), 4.03 (t, J = 7.6 Hz, 1H), 3.14 (q, J = 7.2, 6.8 Hz, 2H), 2.37 (q, J = 7.4 Hz, 2H).

^{13}C NMR (125 MHz, $CDCl_3$) δ: 153.27, 150.20, 144.18, 128.84, 127.84, 126.71, 107.66, 49.05, 41.38, 35.00.

HRMS (ESI-TOF) *m/z*: [M+H$^+$] calculated for $C_{20}H_{21}N_2$, 289.1705; found, 289.1698.

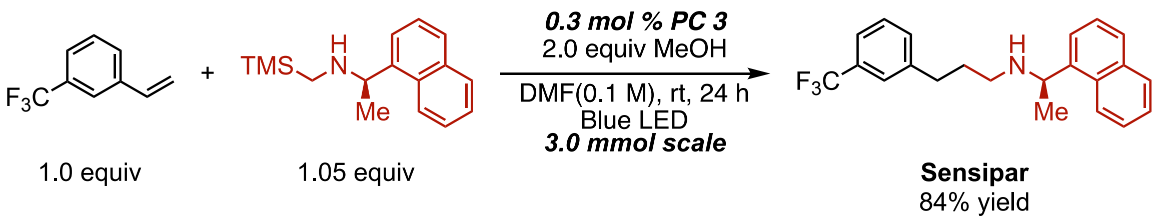

Procedure: $[Ir(ppy)_2(dtbbpy)]PF_6$ (**PC 3**, 9.0 mg, 0.009 mmol, 0.3 mol %), alkene (3.0 mmol, 1.0 equiv), α-TMS amine (3.15 mmol, 1.05 equiv), and DMF (30 mL) were added to a 50 mL round-bottom flask equipped with a stir bar in the glove box under nitrogen atmosphere. The flask was then taken out of glove box and injected 0.24 mL MeOH (6.0 mmol, 2.0 equiv). The resulting solution was allowed to stir inside the photo-reactor (described above) with the lamp and fan on for 24 hours. The reaction crude was quenched by the addition of water, followed by the extraction with EtOAc three times. The combined organic layers were washed with brine and water, dried over $MgSO_4$, concentrated *in vacuo* and then purified by basic alumina chromatography to afford **Sensipar** in 87% yield as a colorless liquid.

Specific optical rotation: $[\alpha]_D^{23}$ = 24.57 (*c* = 1.05 in $CHCl_3$), known in literature as $[\alpha]_D^{24}$ = 21.8 (*c* = 1.0 in $CHCl_3$)[13]

Column Chromatography Condition: 150 g Al_2O_3 + 10 g H_2O, 30 : 1 hexanes/EtOAc with 0.5% MeOH to 15 : 1 hexanes/EtOAc with 1.0% MeOH as gradient eluent.

^{1}H NMR (500 MHz, $CDCl_3$) δ: 8.20 (d, J = 8.2 Hz, 1H), 7.97 – 7.85 (m, 1H), 7.75 (d, J = 8.2 Hz, 1H), 7.65 (d, J = 7.1 Hz, 1H), 7.55 – 7.46 (m, 3H), 7.42 (d, J = 8.2 Hz, 2H), 7.38 – 7.29 (m, 2H), 4.63 (q, J = 6.6 Hz, 1H), 2.92 – 2.46 (m, 4H), 1.84 (p, J = 7.4 Hz, 2H), 1.50 (d, J = 6.5 Hz, 3H), 1.42 (brs, 1H).

^{13}C NMR (125 MHz, $CDCl_3$) δ: 143.24, 141.39, 134.14, 131.90, 131.45, 130.73 (q, J = 31.9 Hz), 129.13, 128.80, 127.33, 125.89, 125.83, 125.46, 125.19 (q, J = 3.8 Hz), 124.41 (q, J = 271.2 Hz), 123.07, 122.81, 122.77 (q, J = 3.8 Hz), 53.94, 47.45, 33.59, 32.06, 23.79.

^{19}F NMR (471 MHz, $CDCl_3$) δ: -62.56 (s).

HRMS (ESI-TOF) *m/z*: [M+H$^+$] calculated for $C_{22}H_{22}F_3N$, 358.1777; found, 358.1779.

H. Spectra

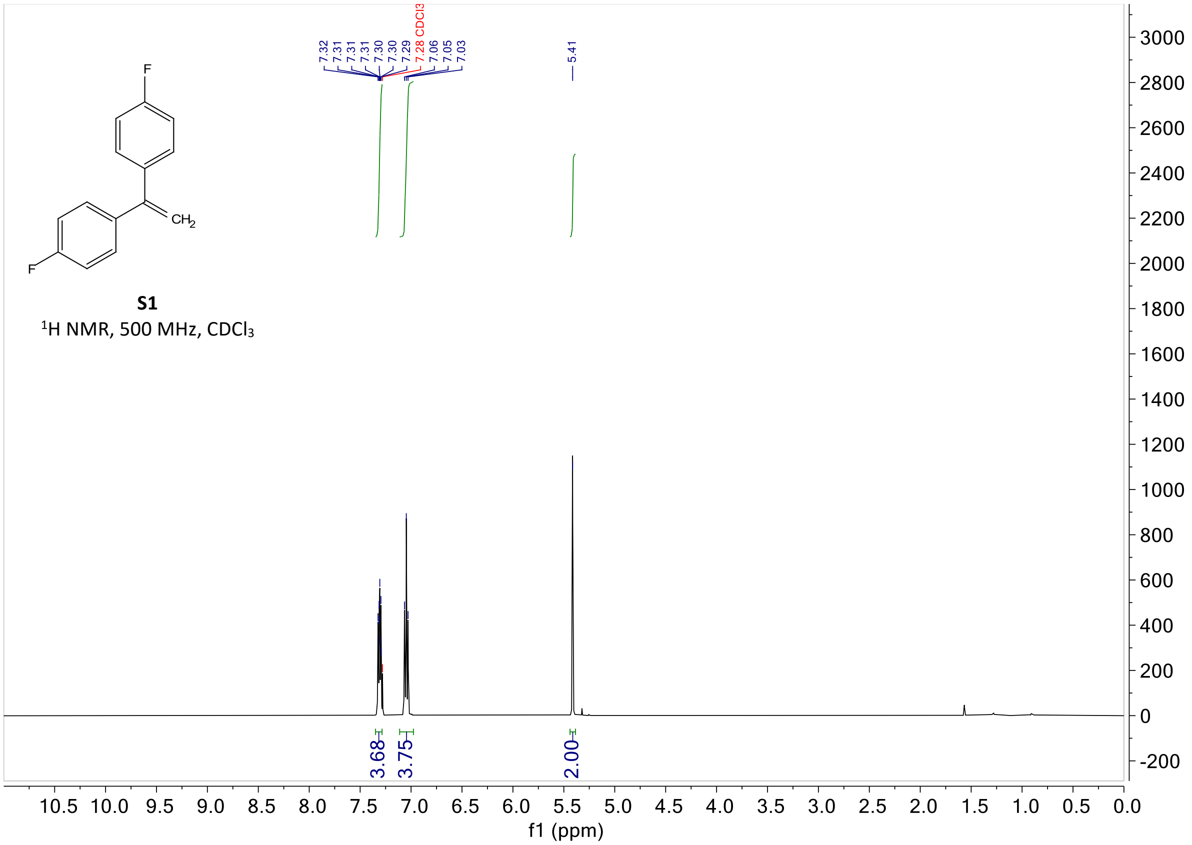

Supplementary Figure 15. ^{1}H NMR spectra of compound **S1**

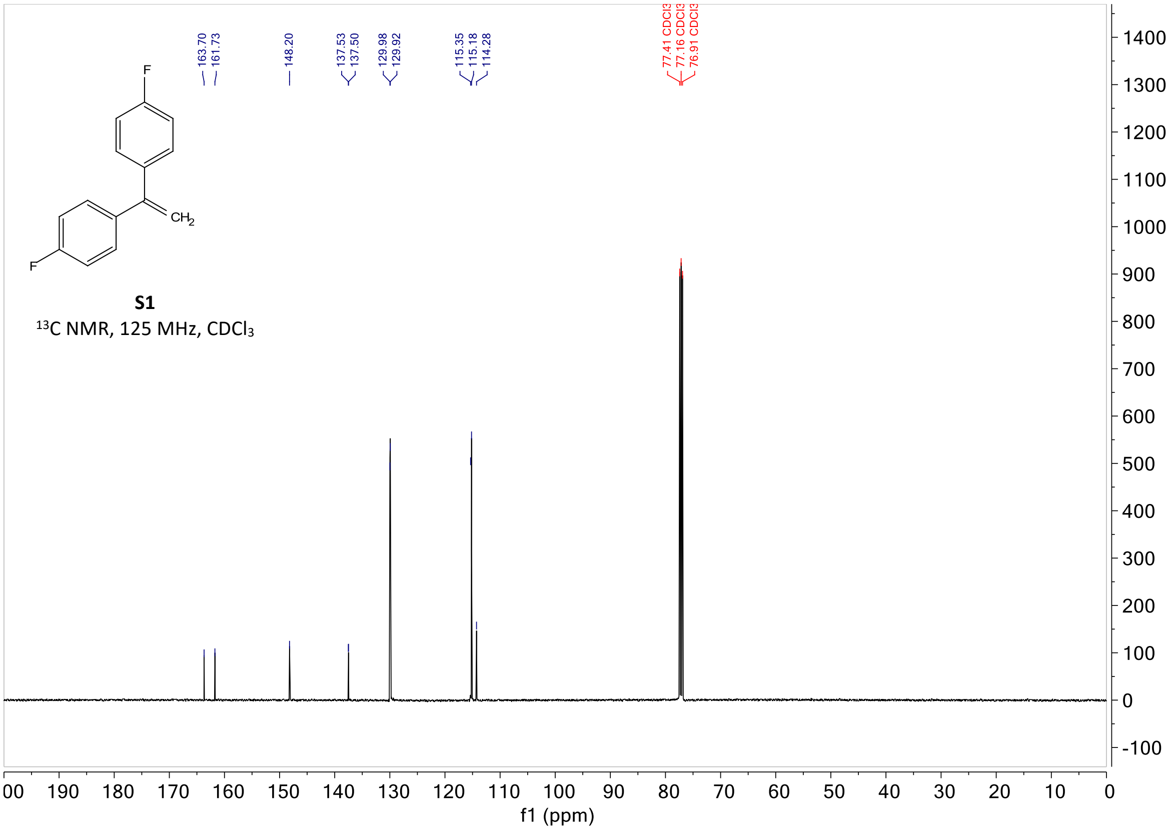

Supplementary Figure 16. ^{13}C NMR spectra of compound **S1**

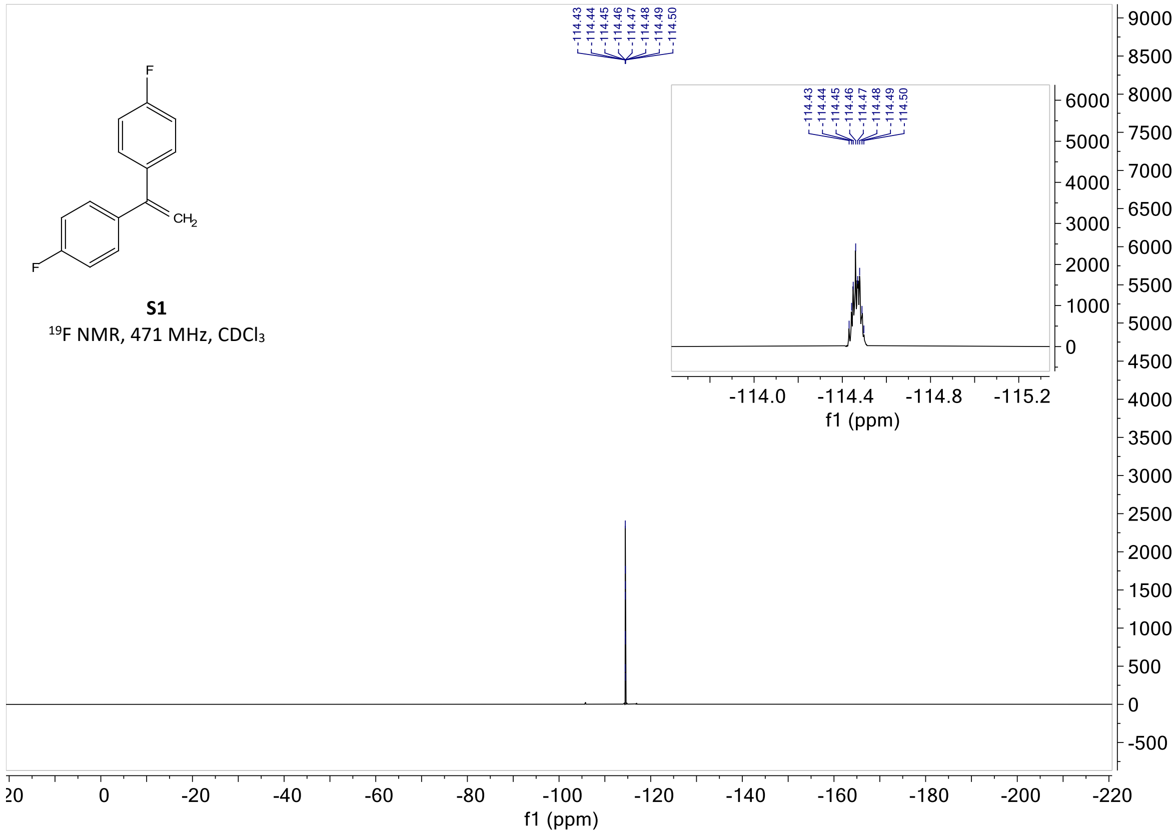

Supplementary Figure 17. ^{19}F NMR spectra of compound **S1**

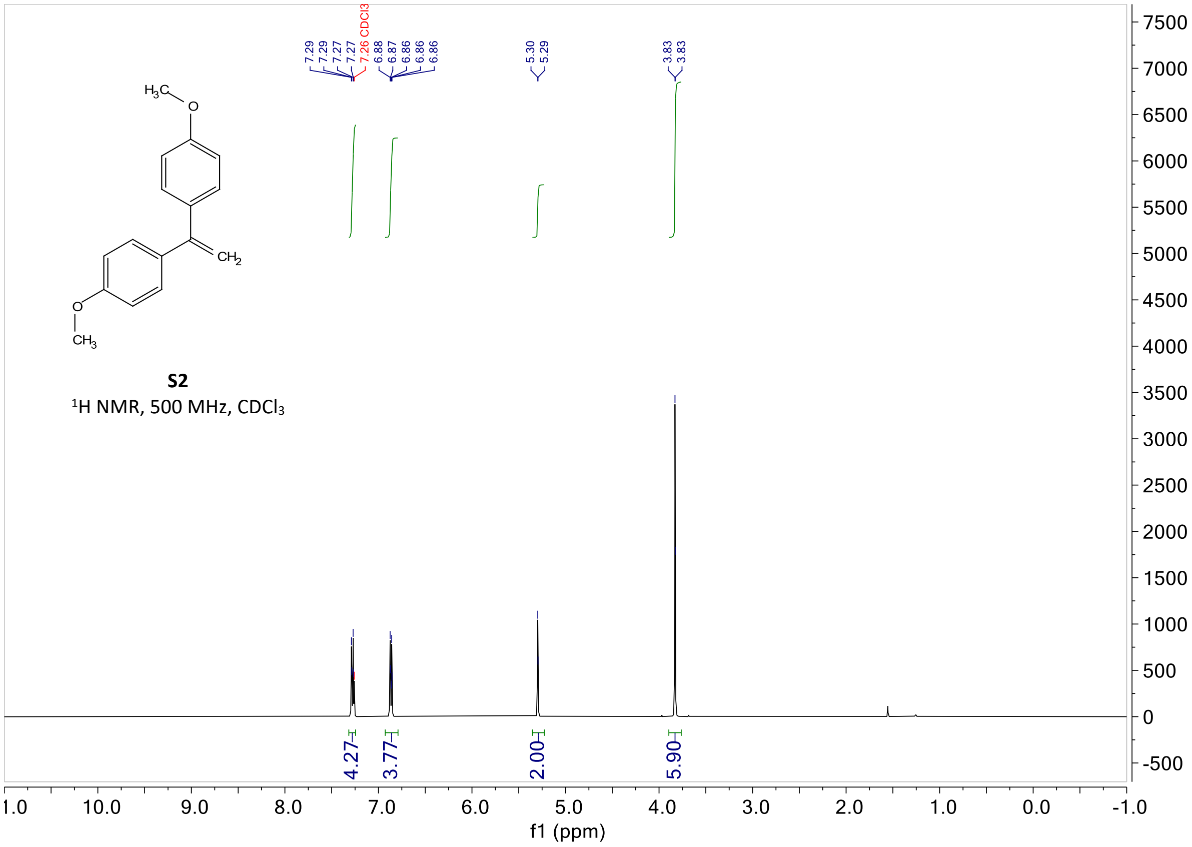

Supplementary Figure 18. ^{1}H NMR spectra of compound **S2**

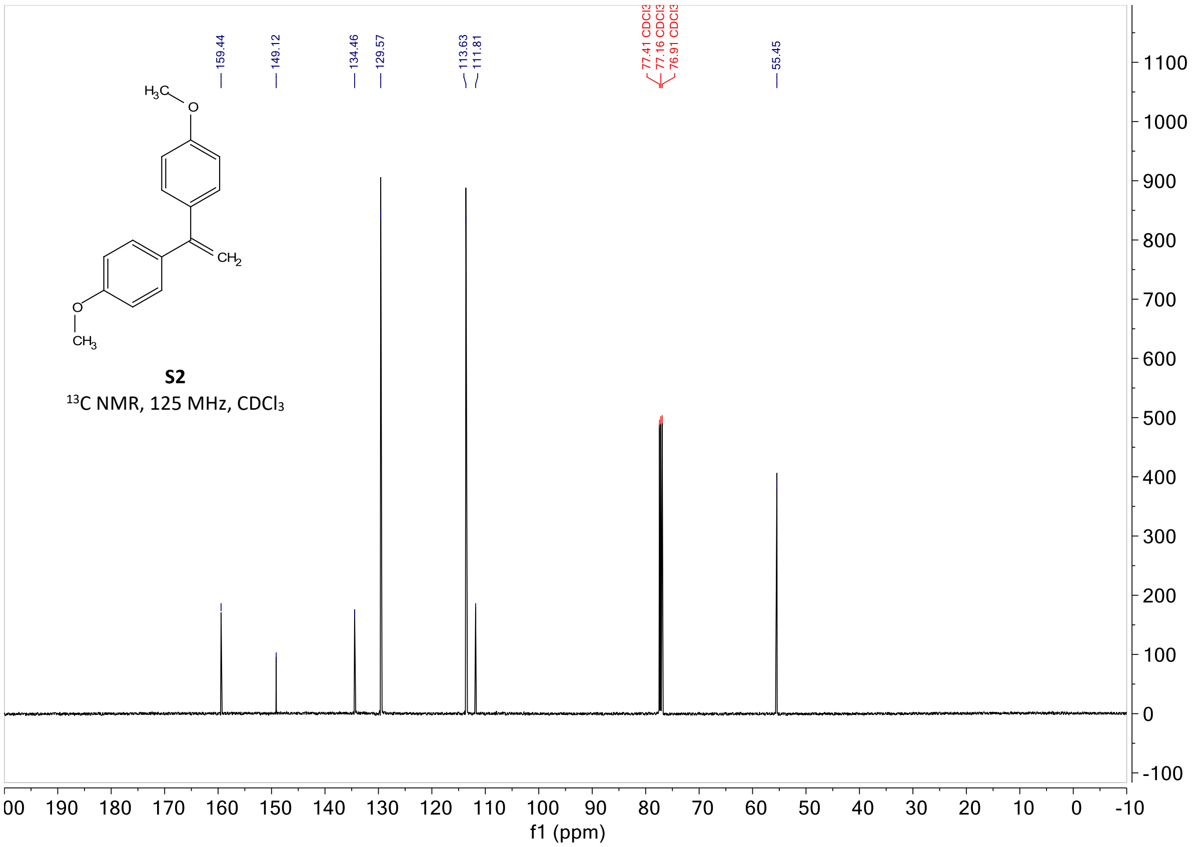

Supplementary Figure 19. ^{13}C NMR spectra of compound **S2**

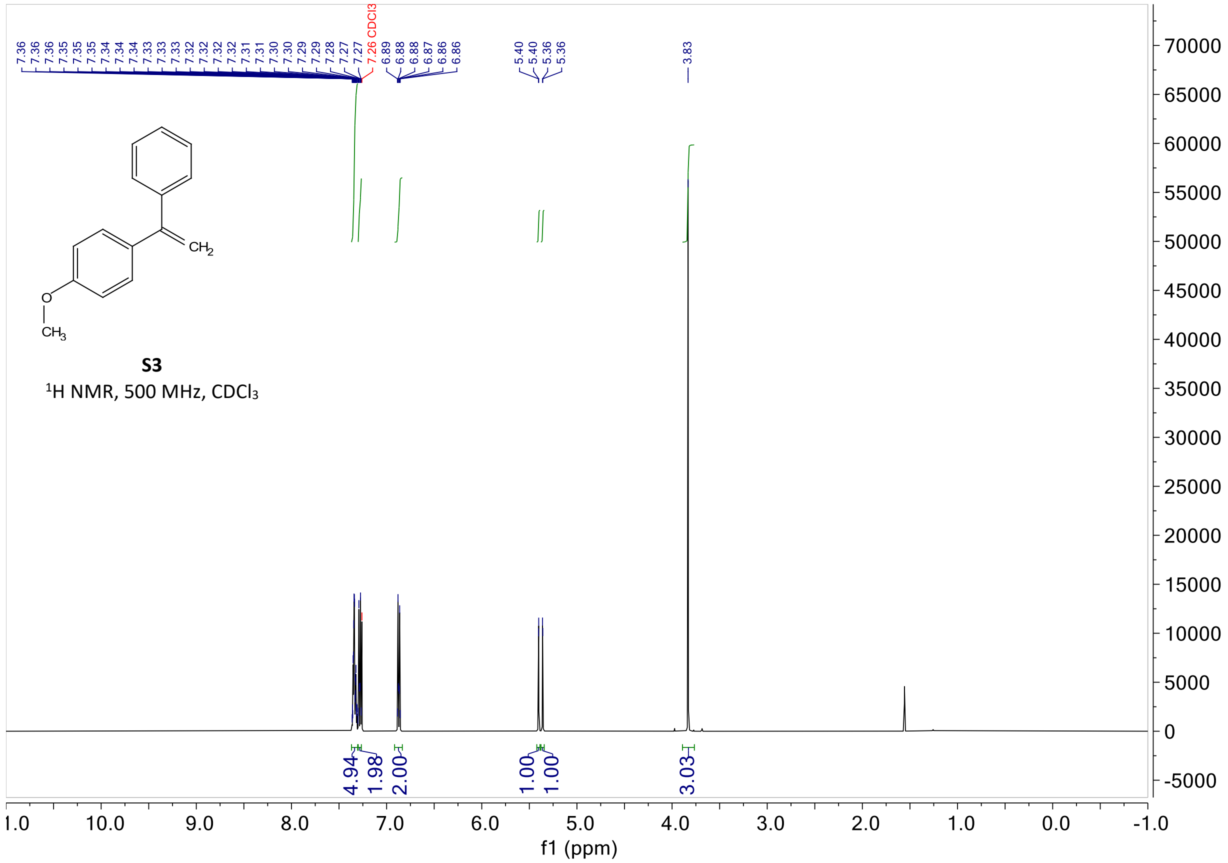

Supplementary Figure 20. ^{1}H NMR spectra of compound **S3**

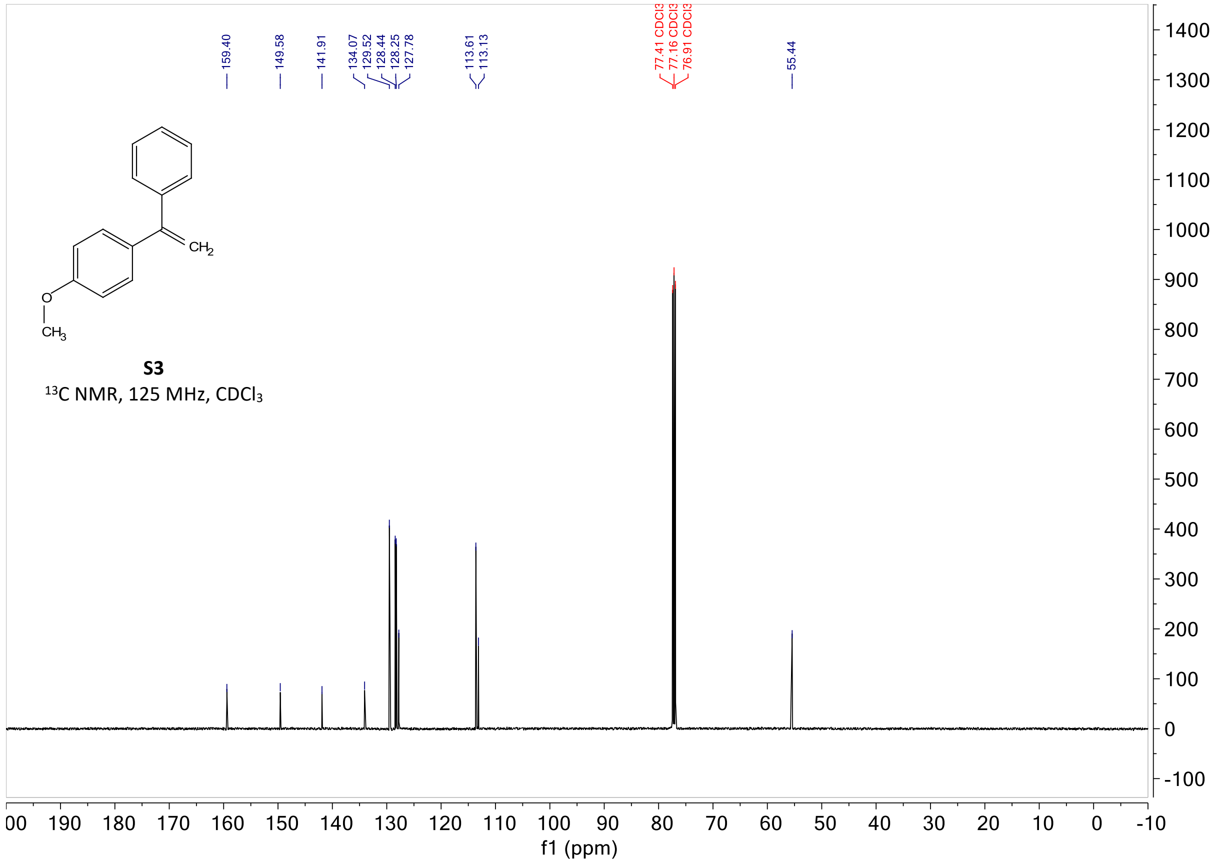

Supplementary Figure 21. ^{13}C NMR spectra of compound **S3**

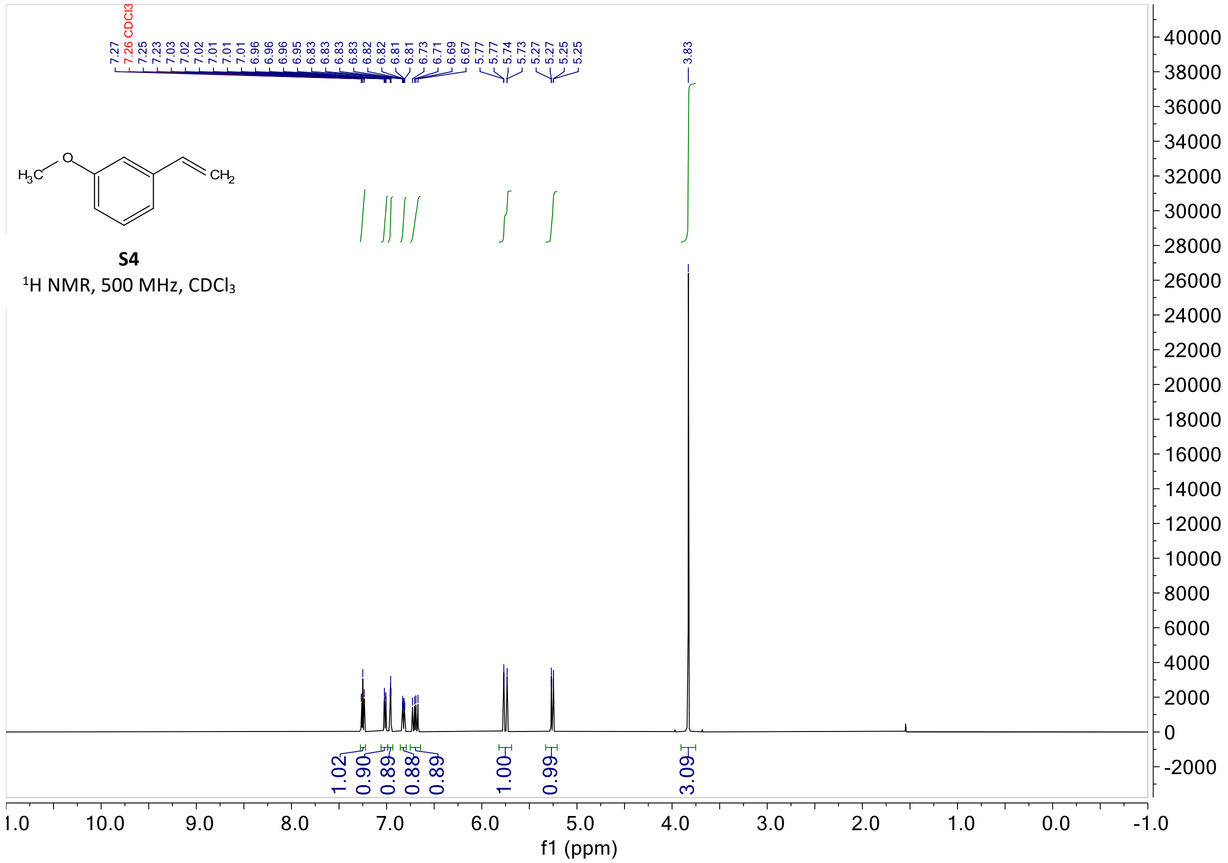

Supplementary Figure 22. ^{1}H NMR spectra of compound **S4**

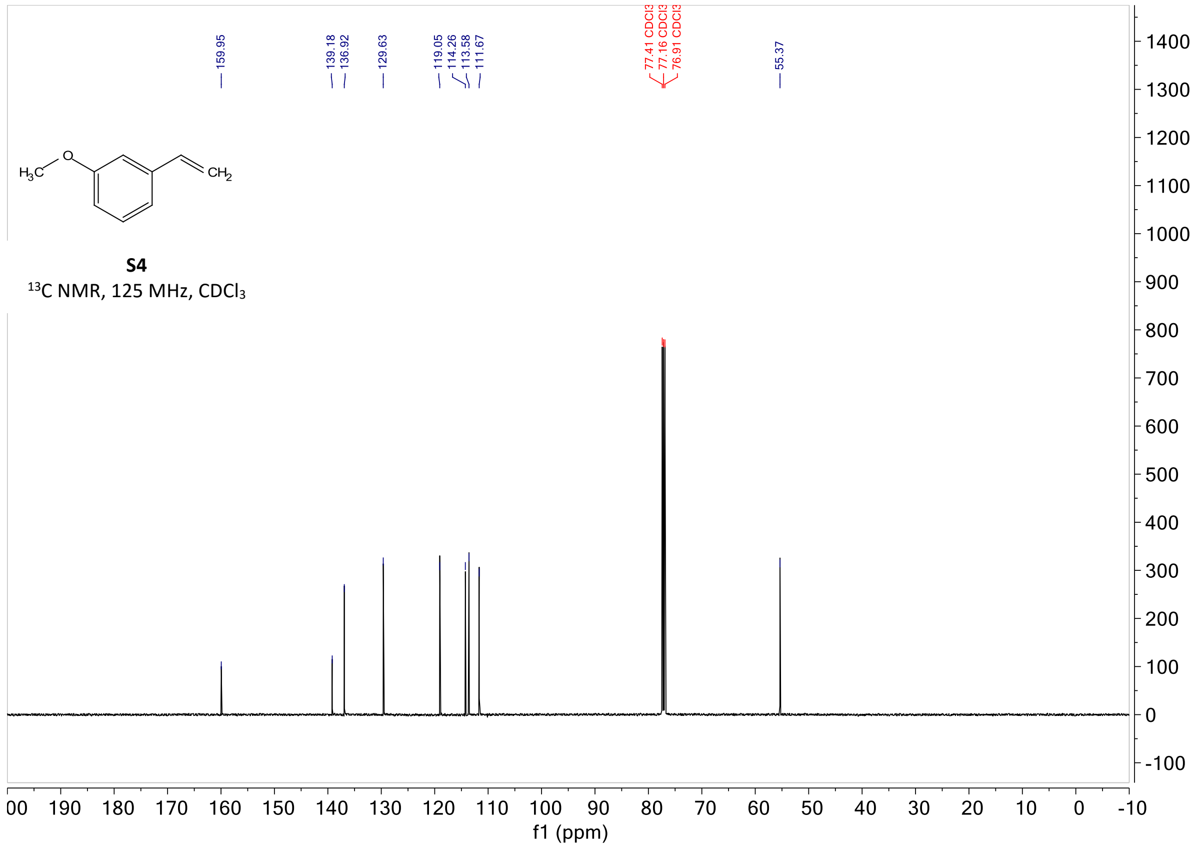

Supplementary Figure 23. ^{13}C NMR spectra of compound **S4**

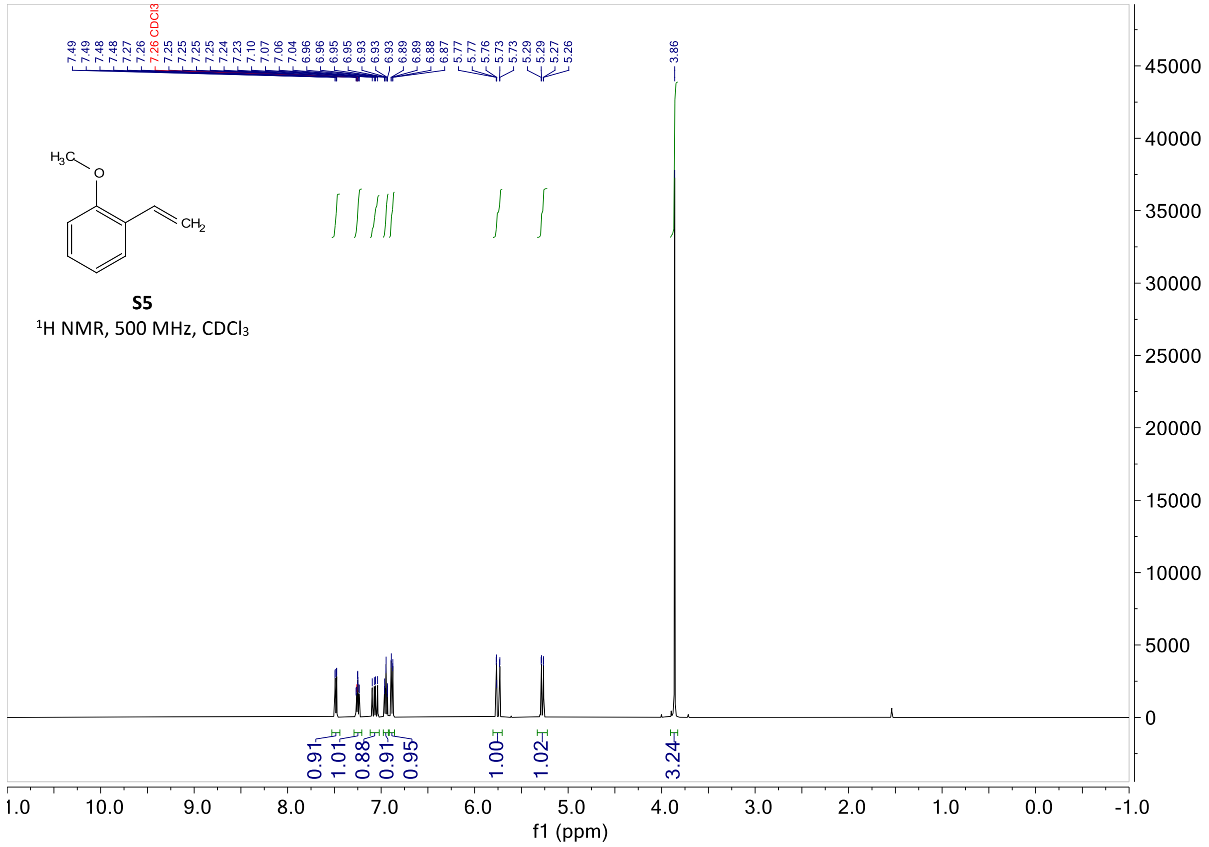

Supplementary Figure 24. ^{1}H NMR spectra of compound **S5**

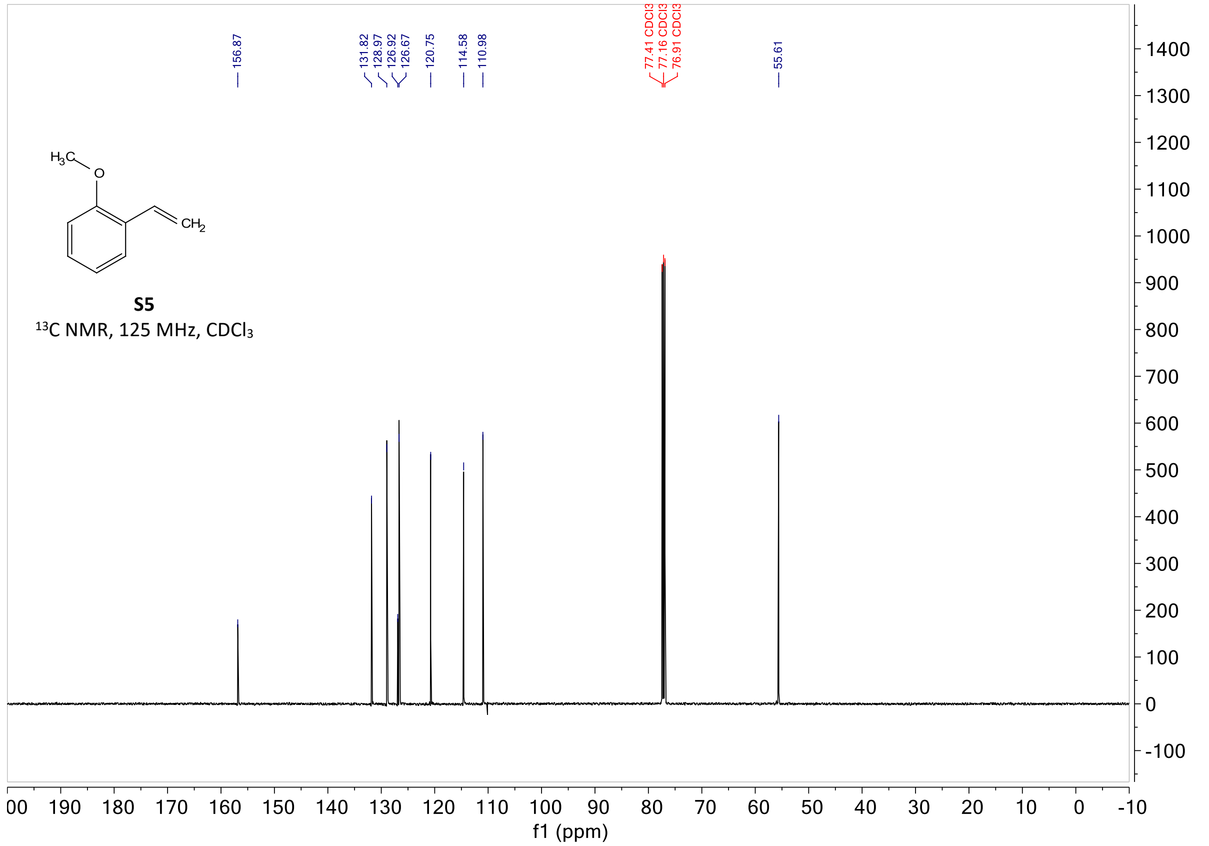

Supplementary Figure 25. ^{13}C NMR spectra of compound **S5**

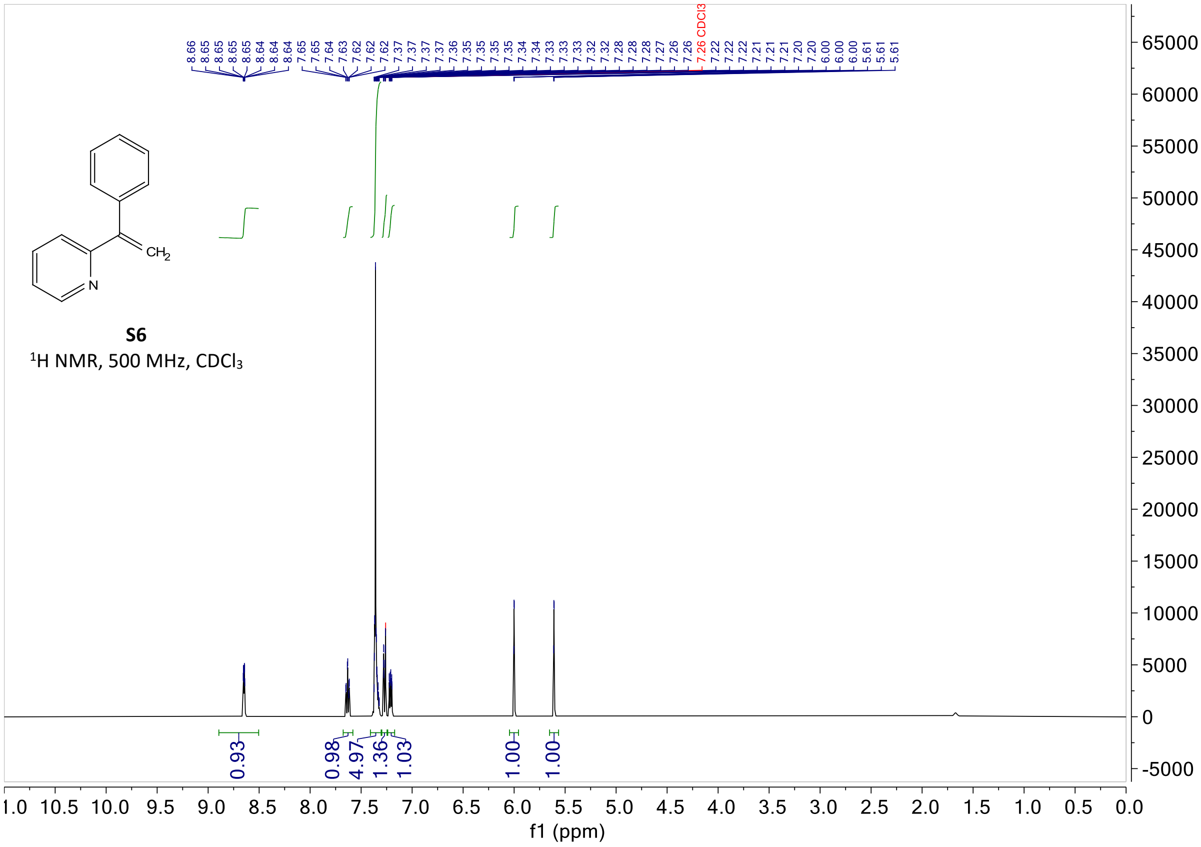

Supplementary Figure 26. ^{1}H NMR spectra of compound **S6**

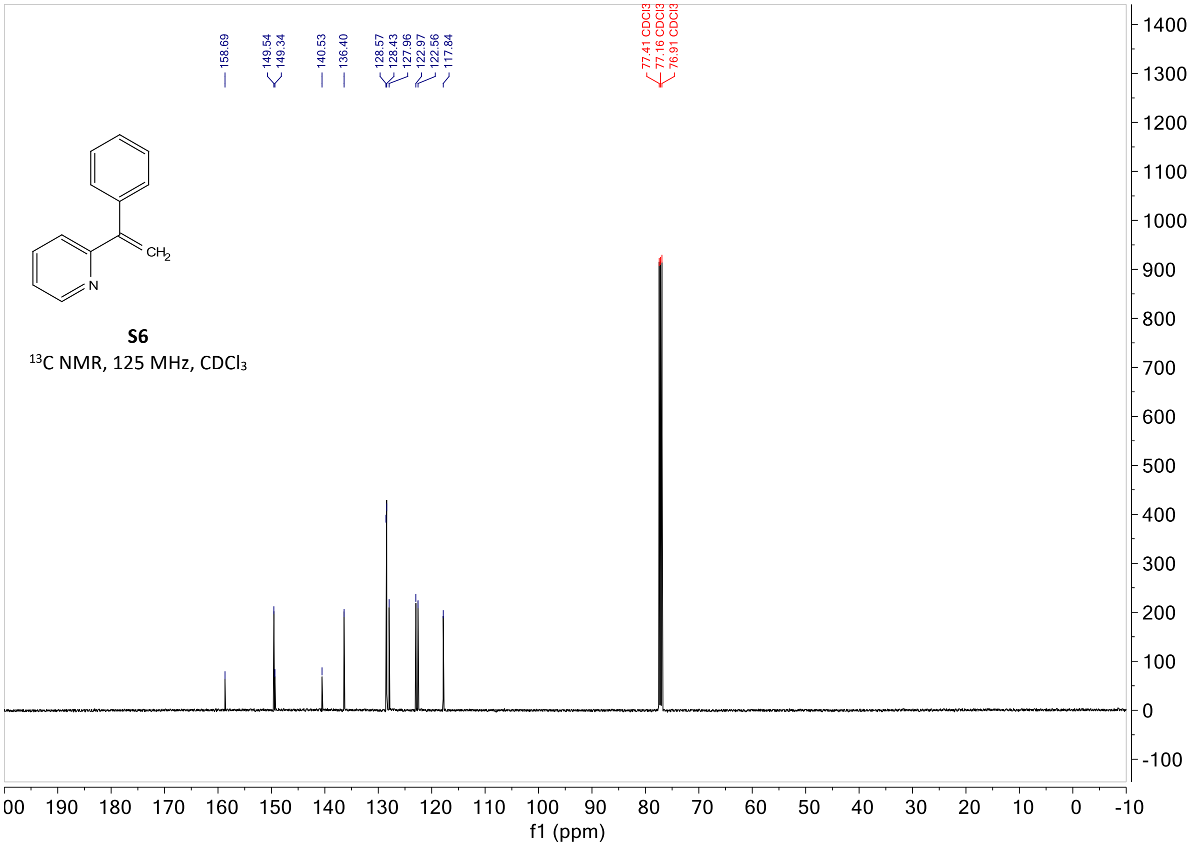

Supplementary Figure 27. ^{13}C NMR spectra of compound **S6**

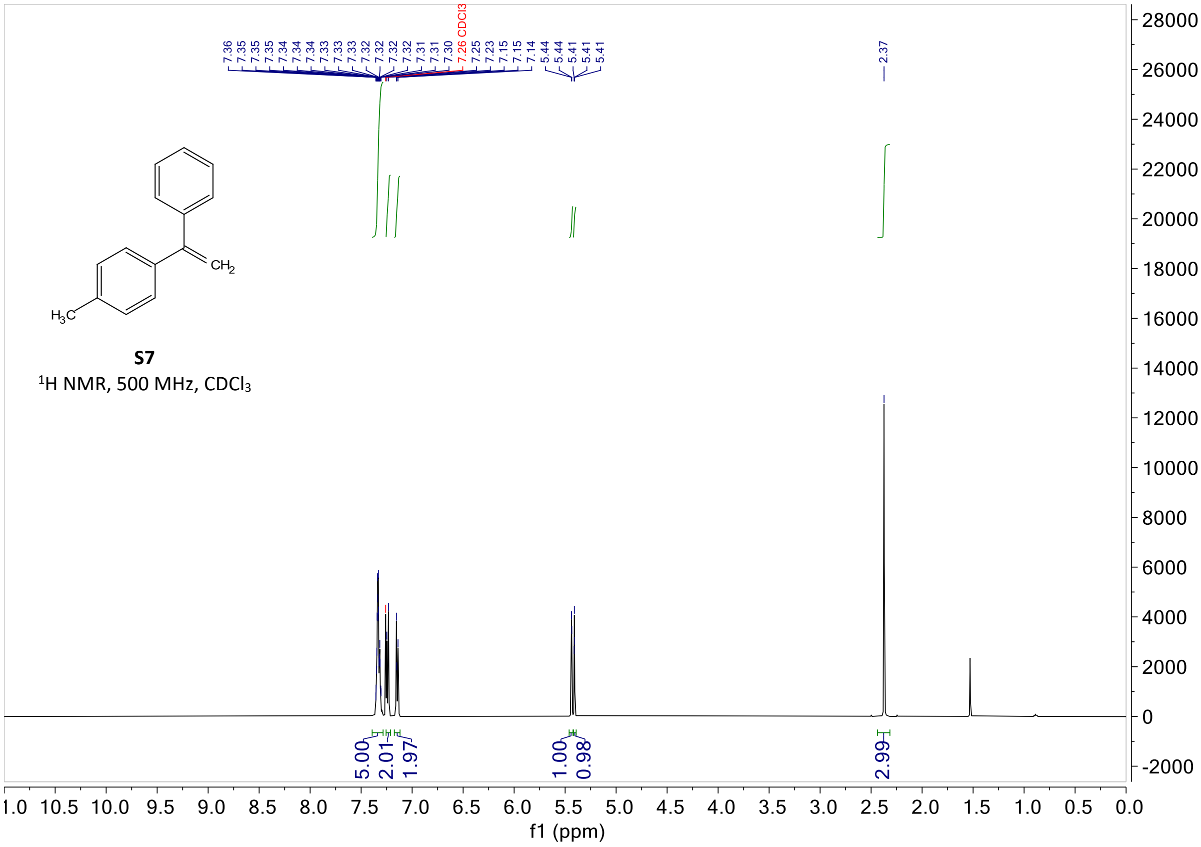

Supplementary Figure 28. ^{1}H NMR spectra of compound **S7**

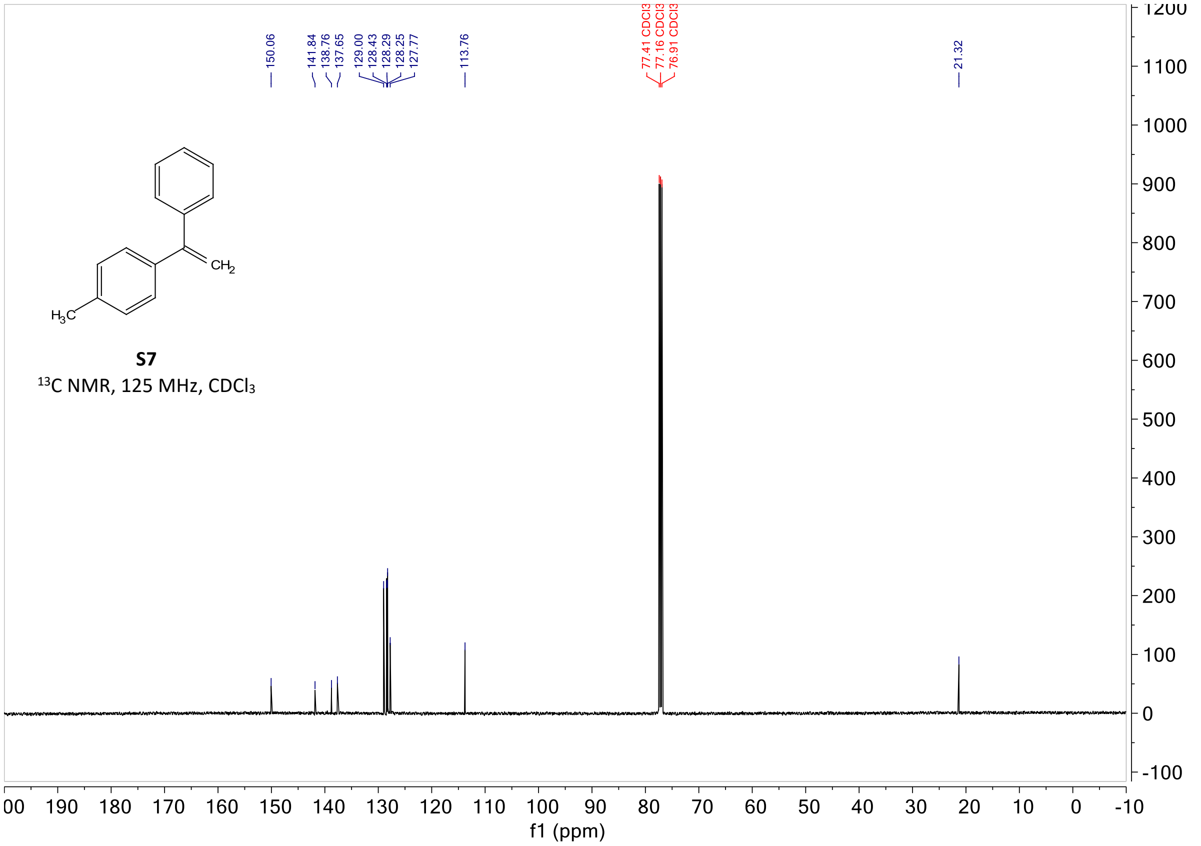

Supplementary Figure 29. ^{13}C NMR spectra of compound **S7**

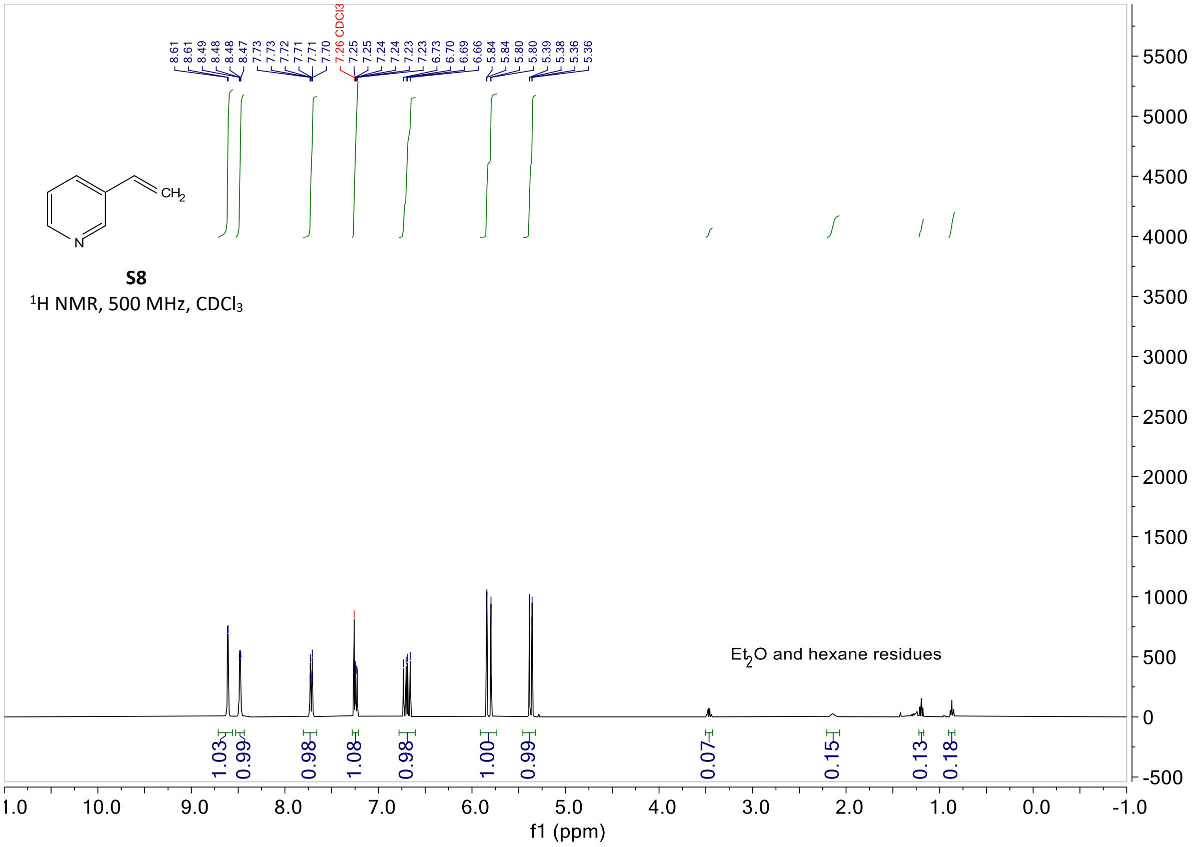

Supplementary Figure 30. ^{1}H NMR spectra of compound **S8**

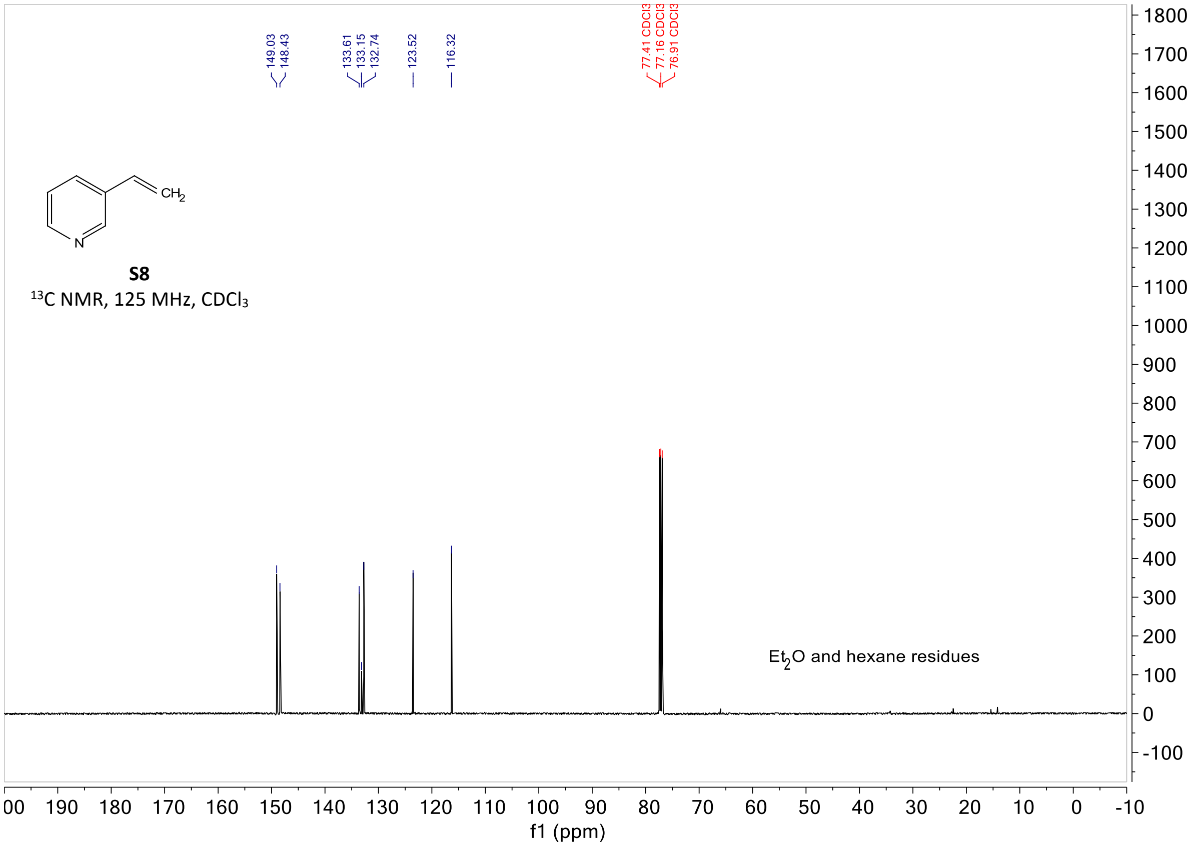

Supplementary Figure 31. ^{13}C NMR spectra of compound **S8**

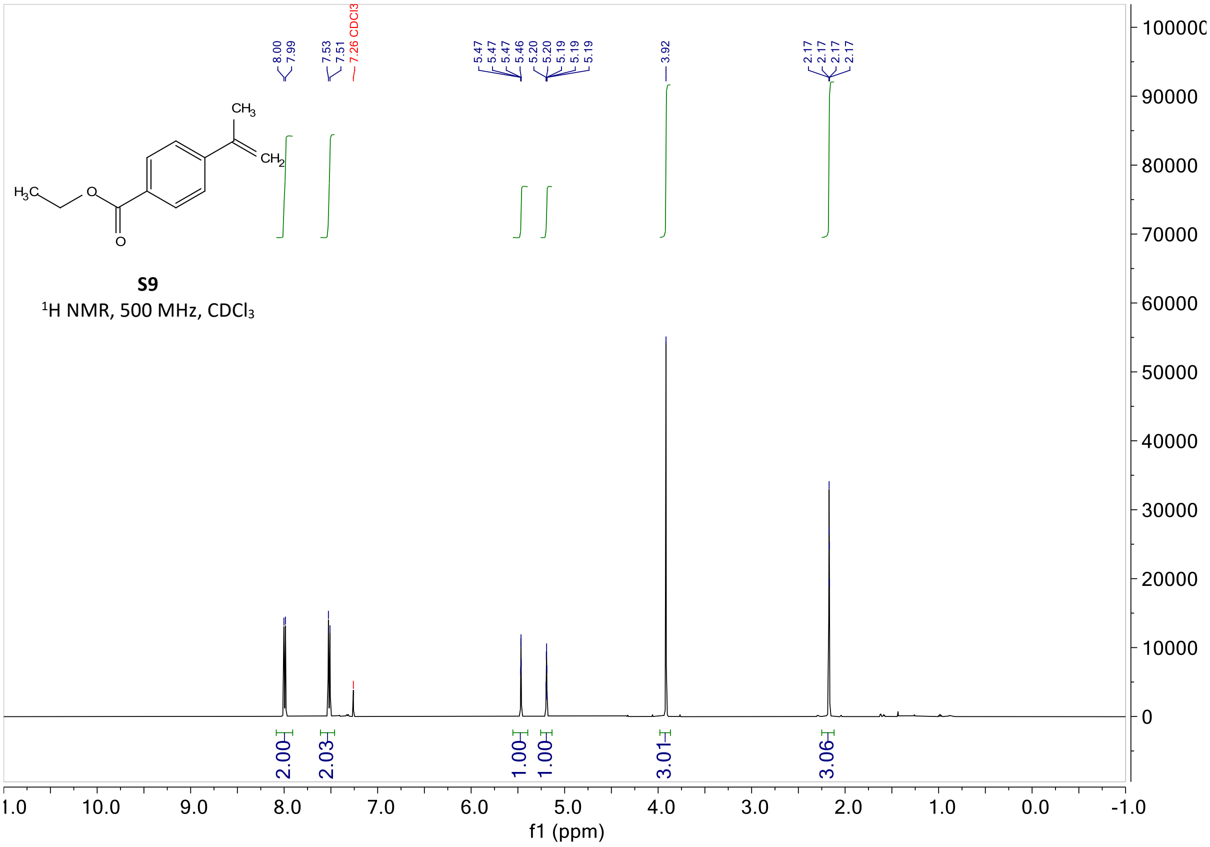

Supplementary Figure 32. ^{1}H NMR spectra of compound **S9**

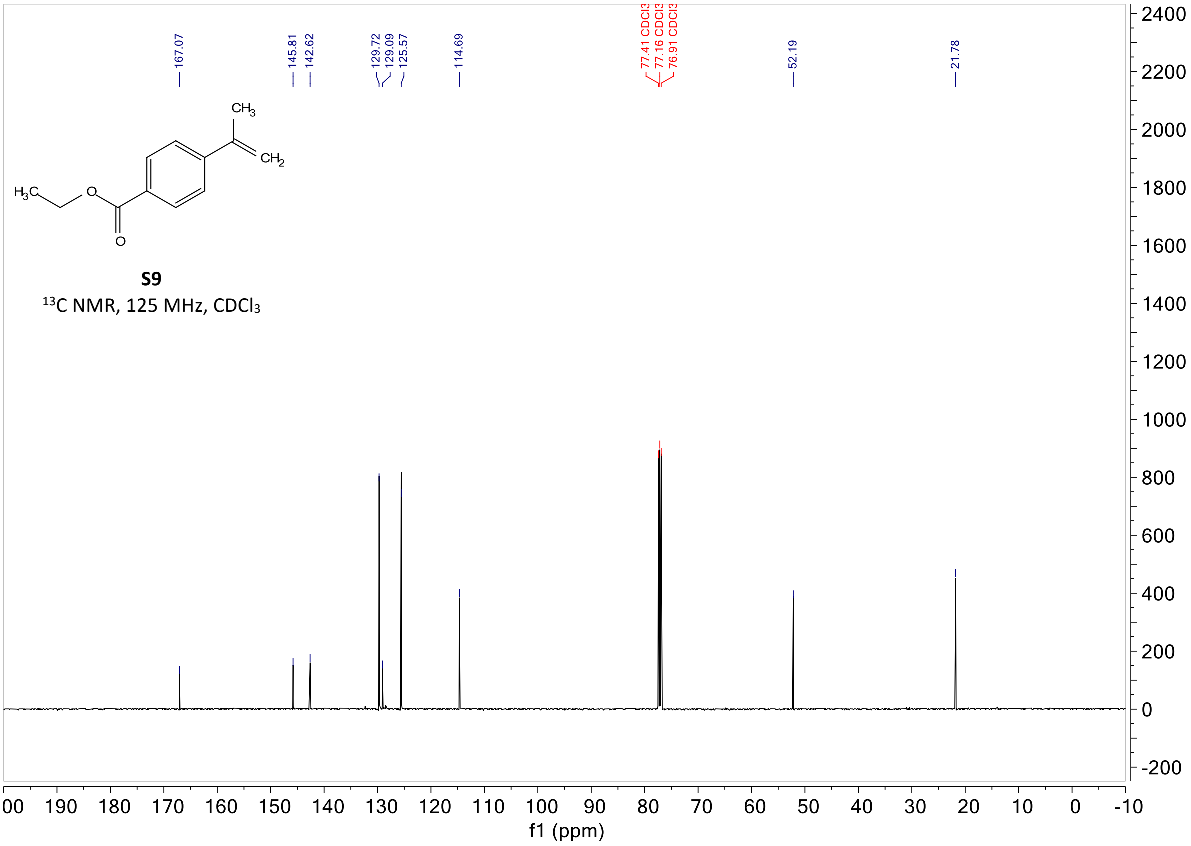

Supplementary Figure 33. ^{13}C NMR spectra of compound **S9**

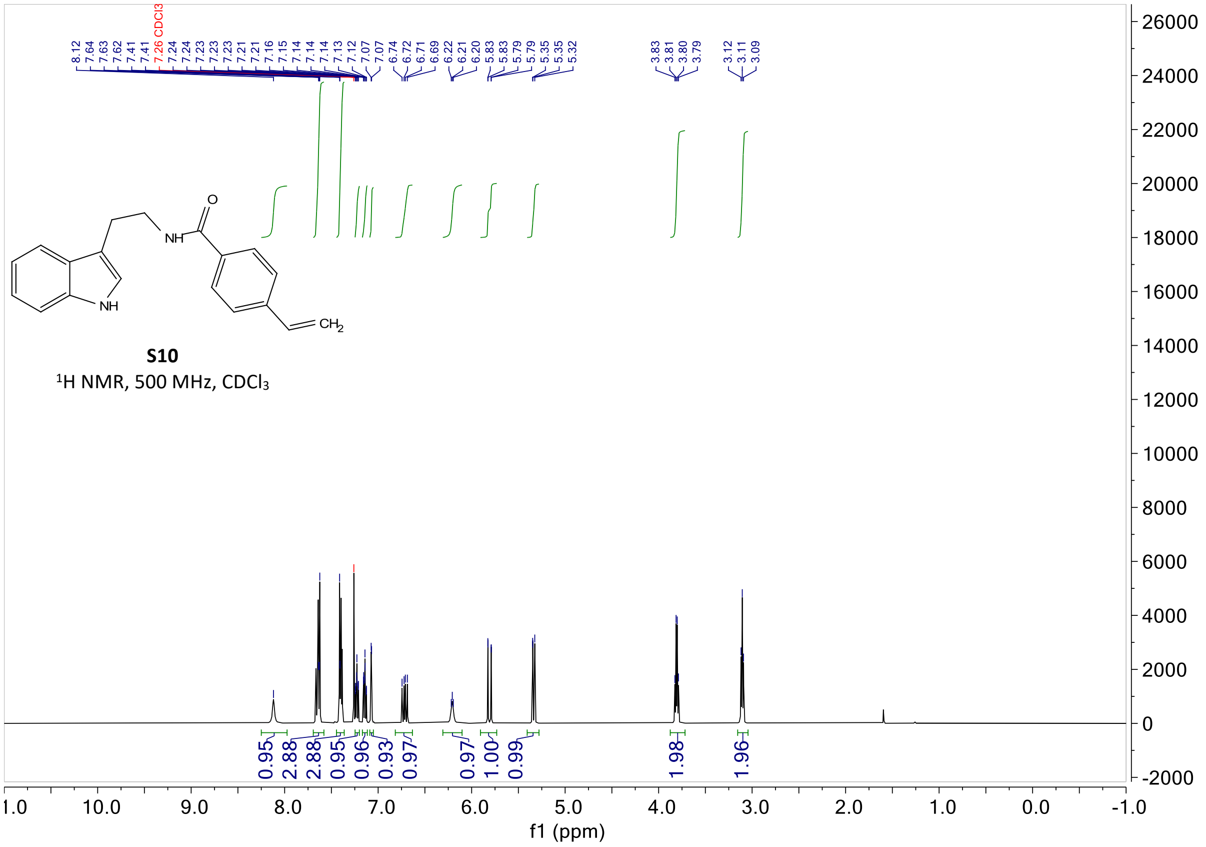

Supplementary Figure 34. ^{1}H NMR spectra of compound **S10**

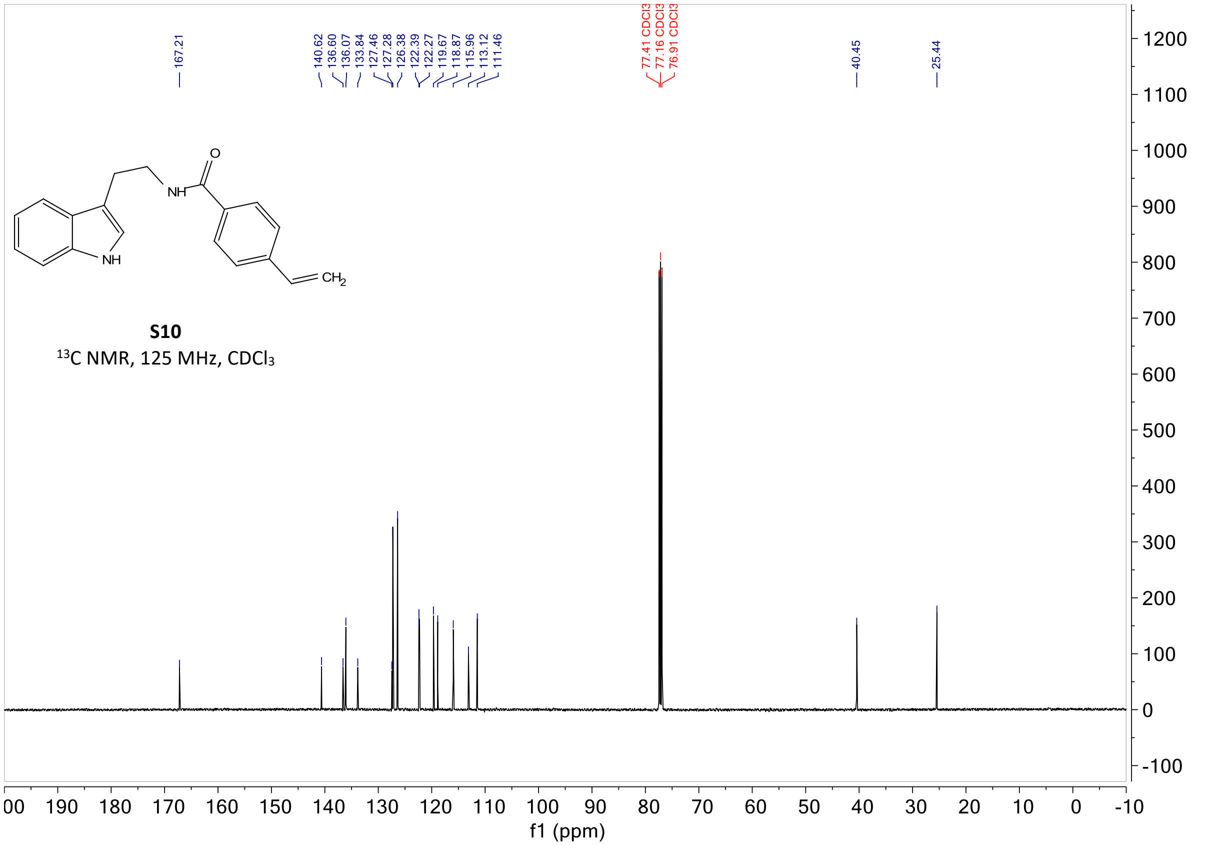

Supplementary Figure 35. ^{13}C NMR spectra of compound **S10**

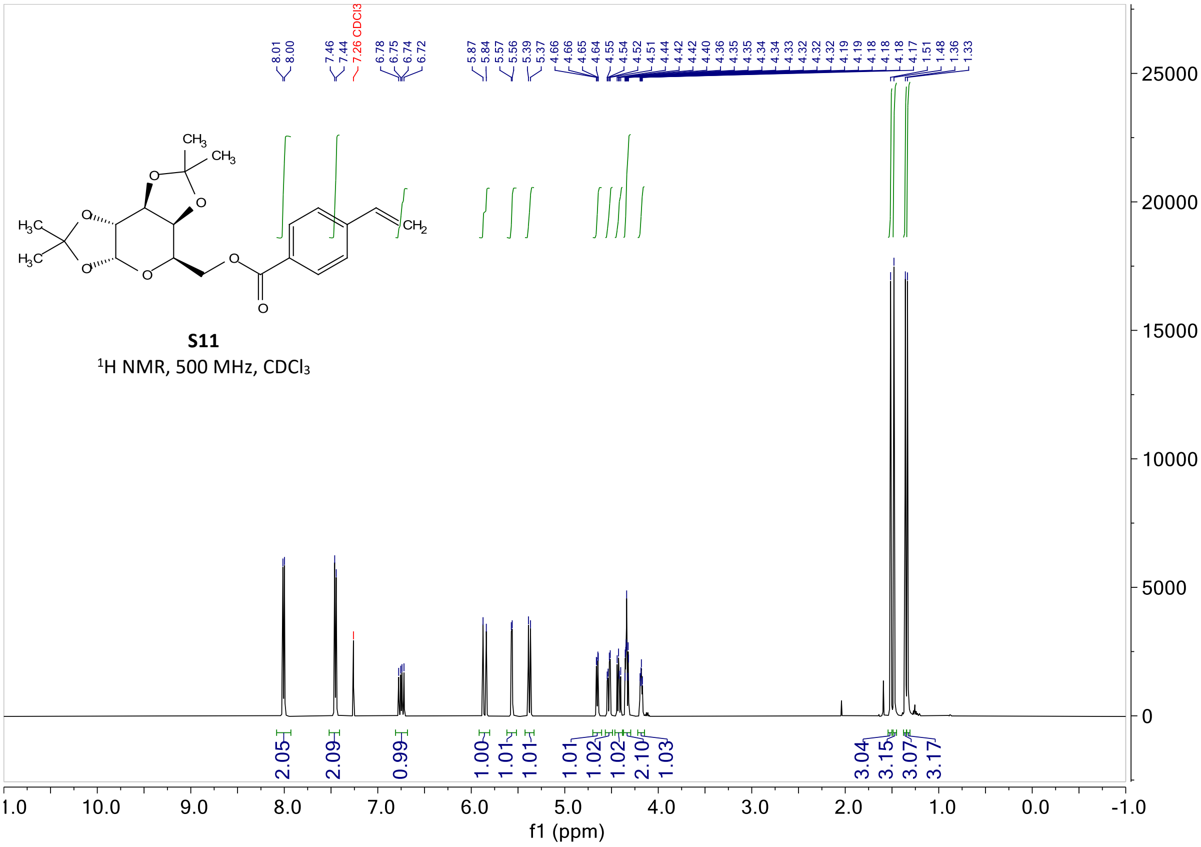

Supplementary Figure 36. ^{1}H NMR spectra of compound **S11**

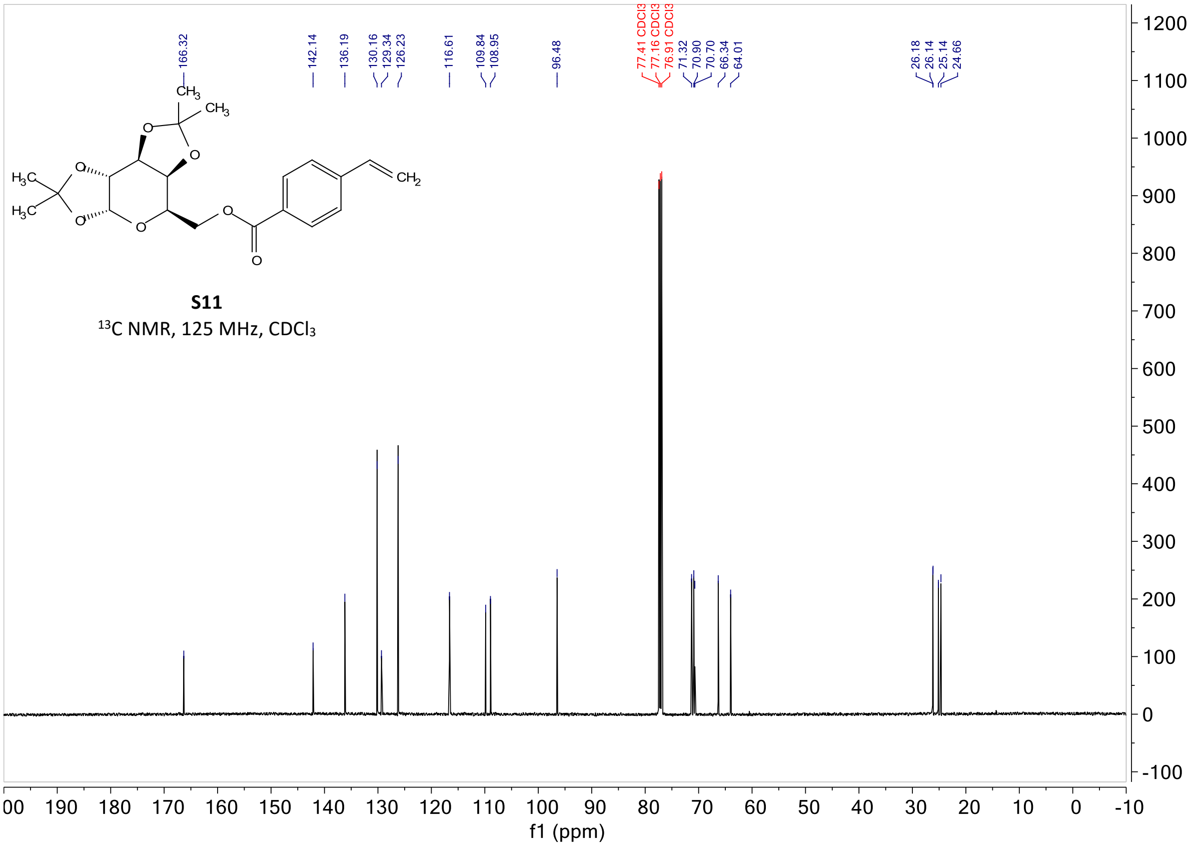

Supplementary Figure 37. ^{13}C NMR spectra of compound **S11**

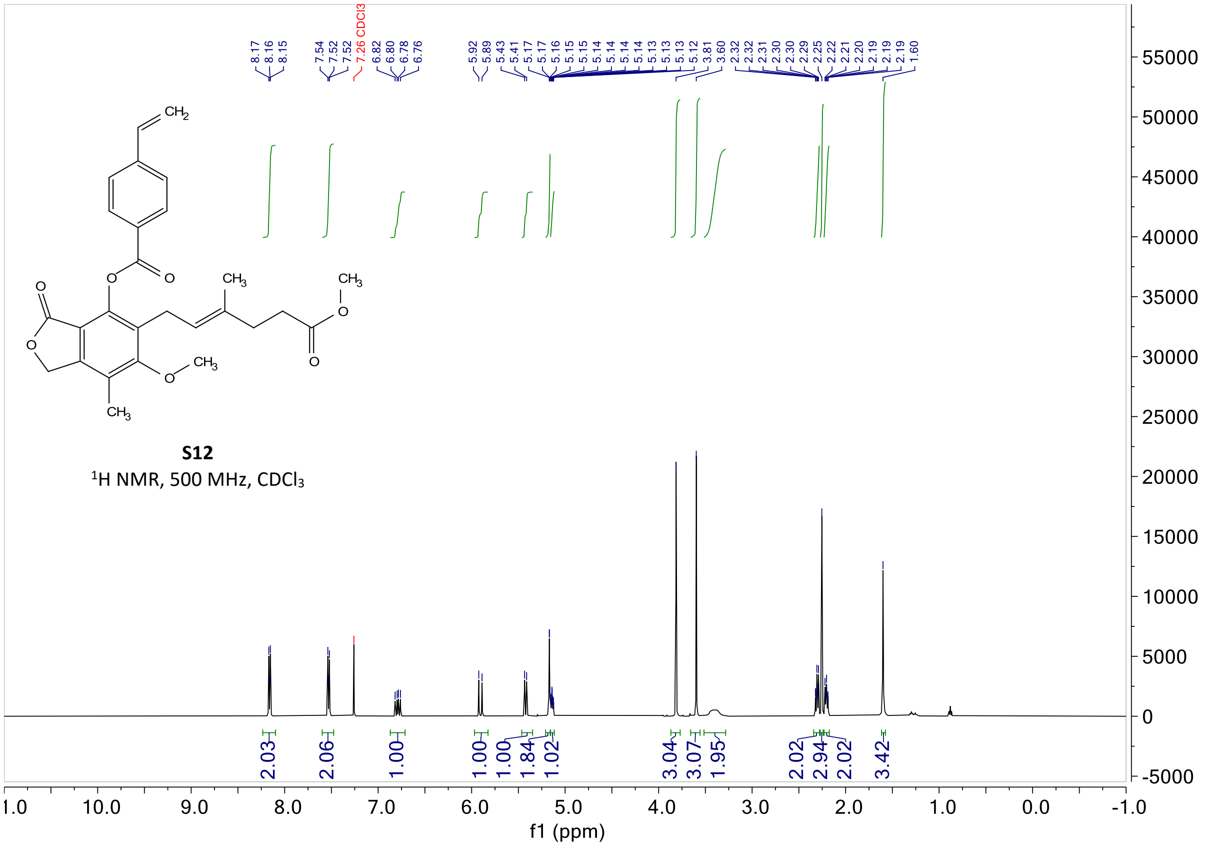

Supplementary Figure 38. ^{1}H NMR spectra of compound **S12**

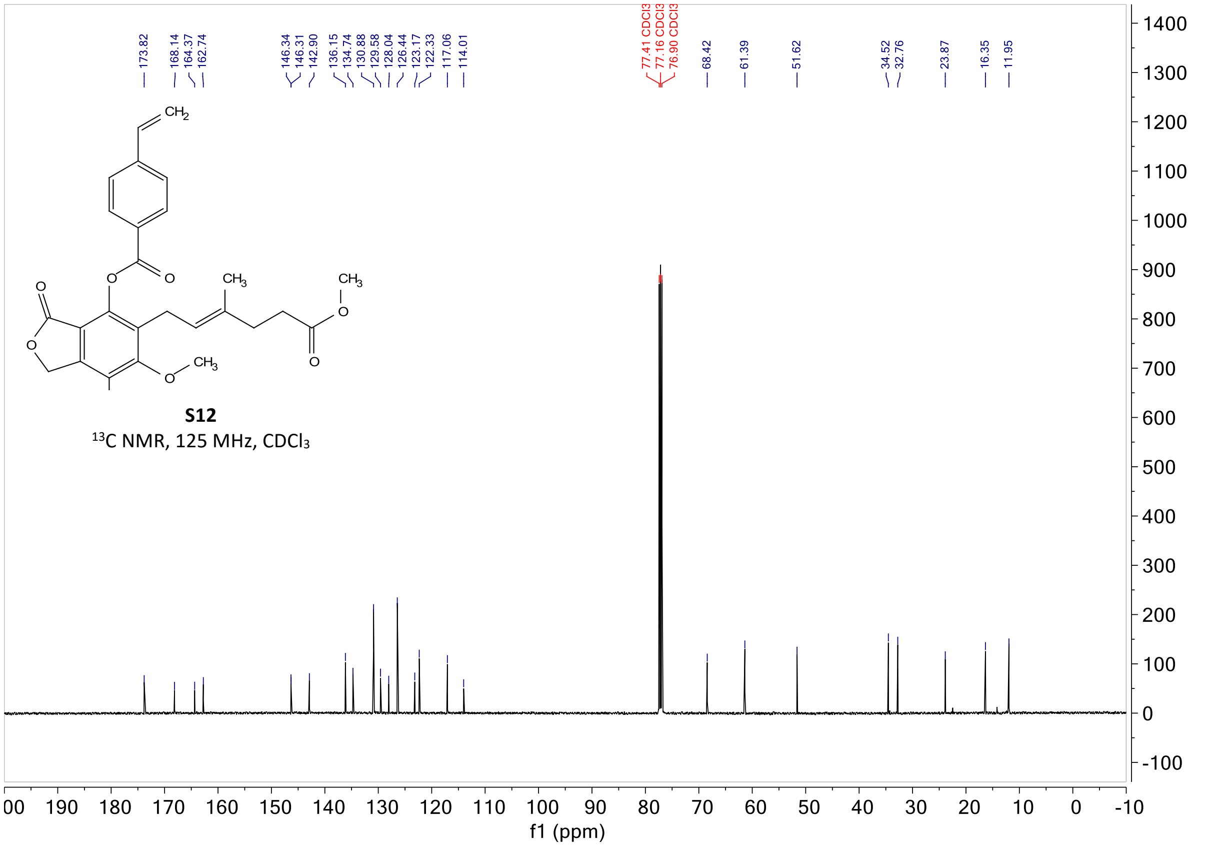

Supplementary Figure 39. ^{13}C NMR spectra of compound **S12**

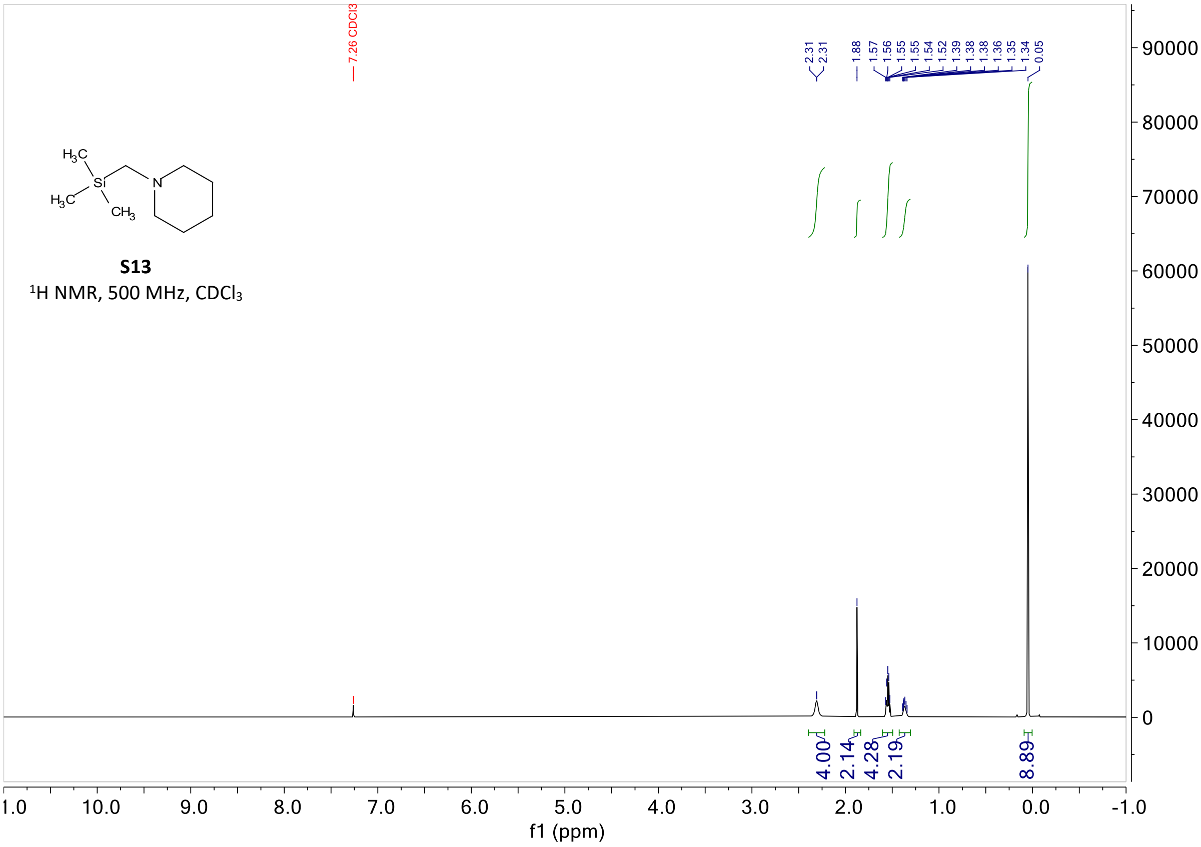

Supplementary Figure 40. ^{1}H NMR spectra of compound **S13**

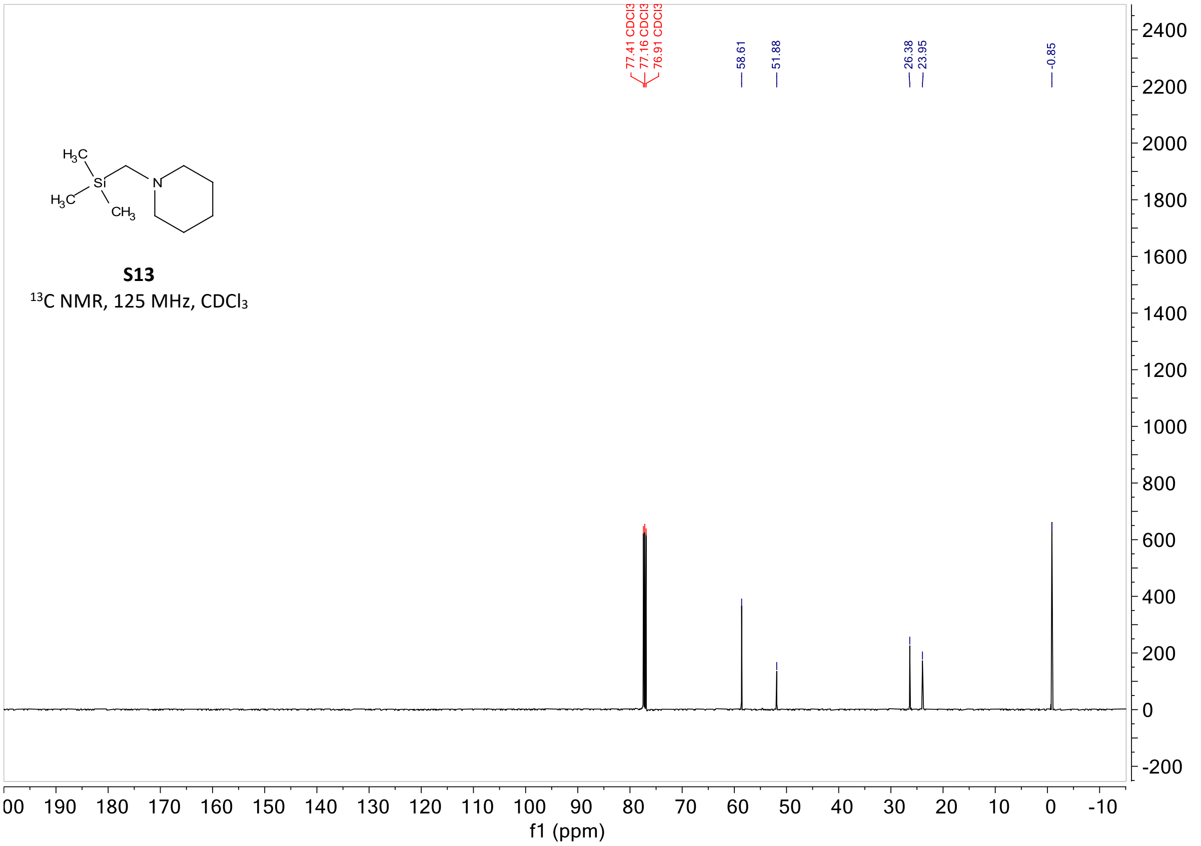

Supplementary Figure 41. ^{13}C NMR spectra of compound **S13**

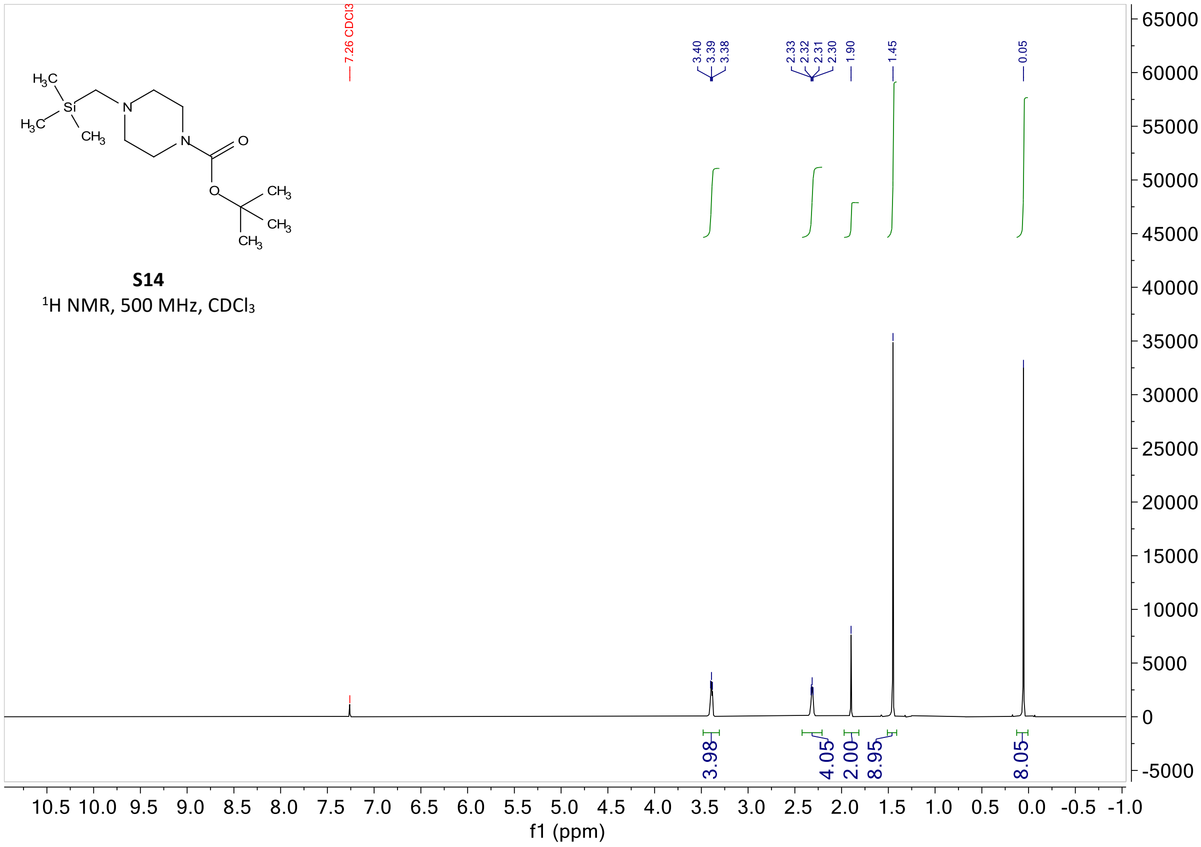

Supplementary Figure 42. ^{1}H NMR spectra of compound **S14**

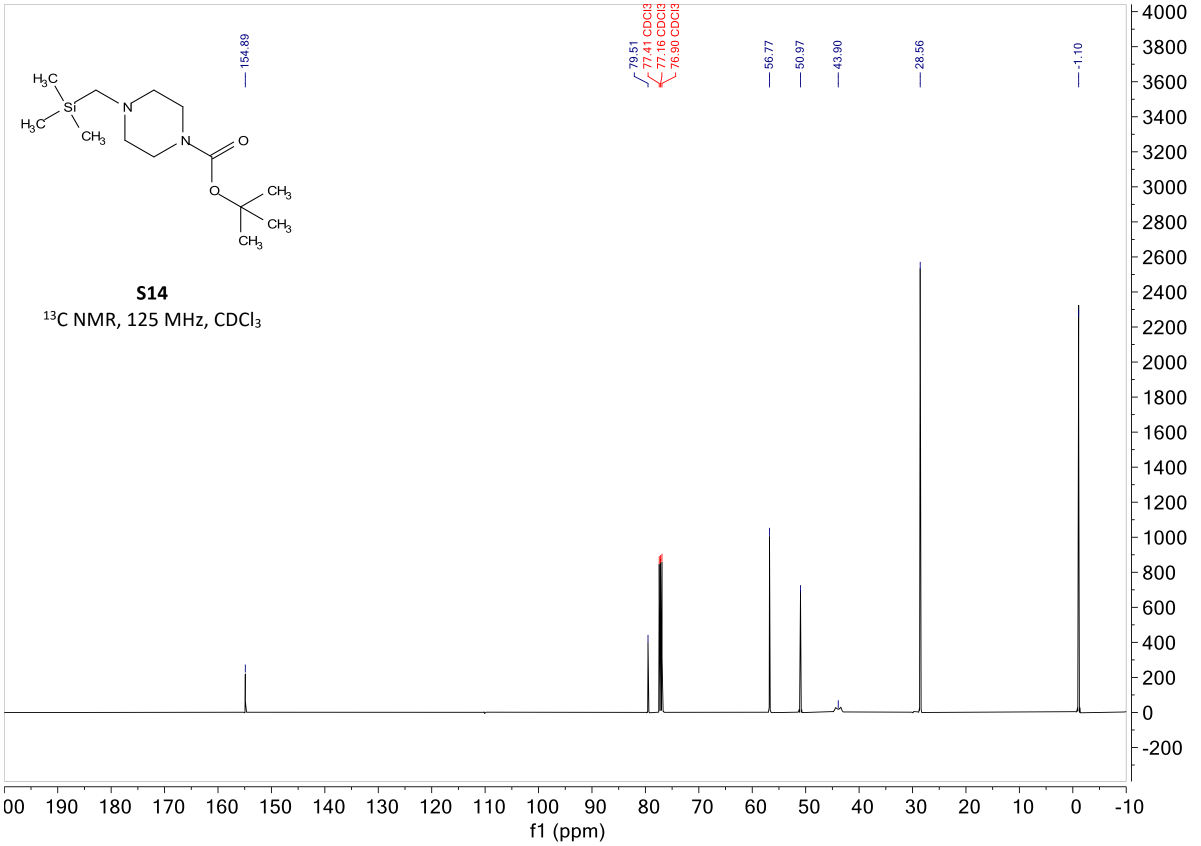

Supplementary Figure 43. ^{13}C NMR spectra of compound **S14**

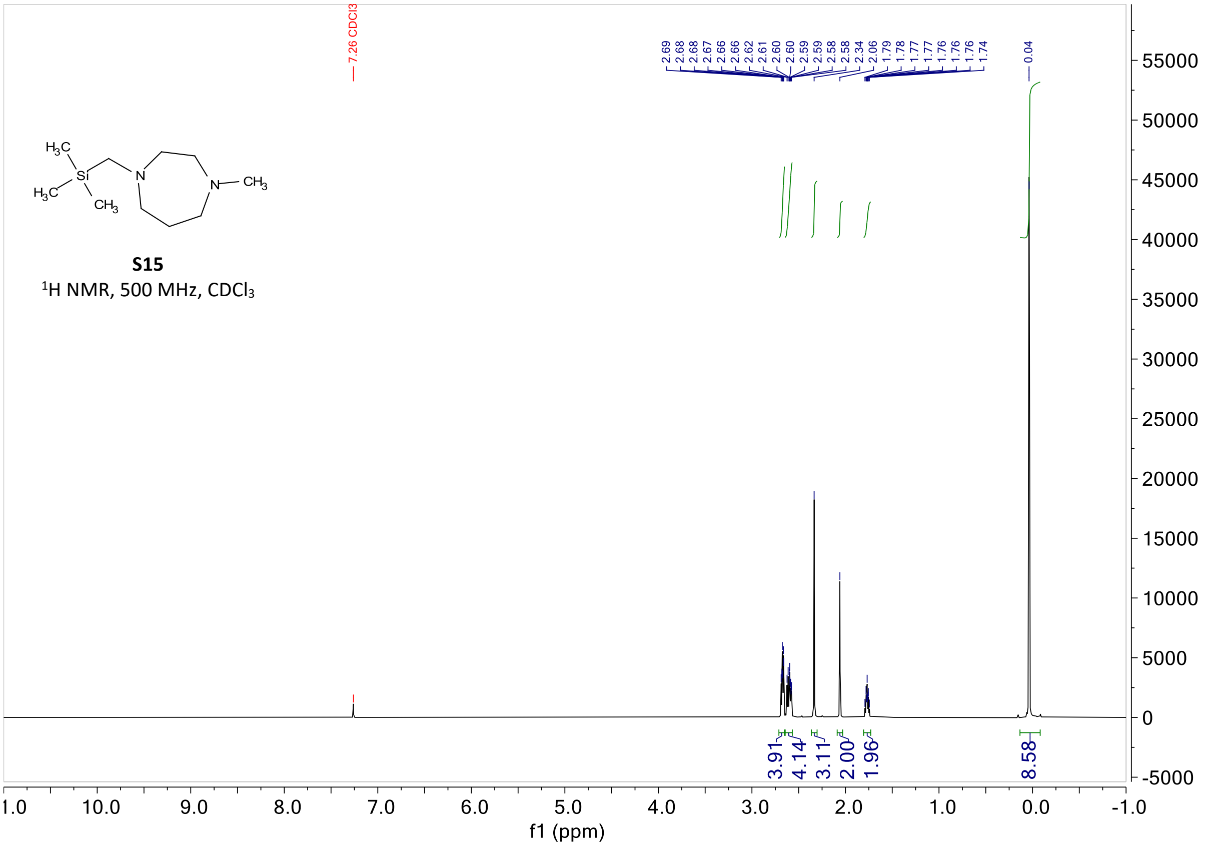

Supplementary Figure 44. ^{1}H NMR spectra of compound **S15**

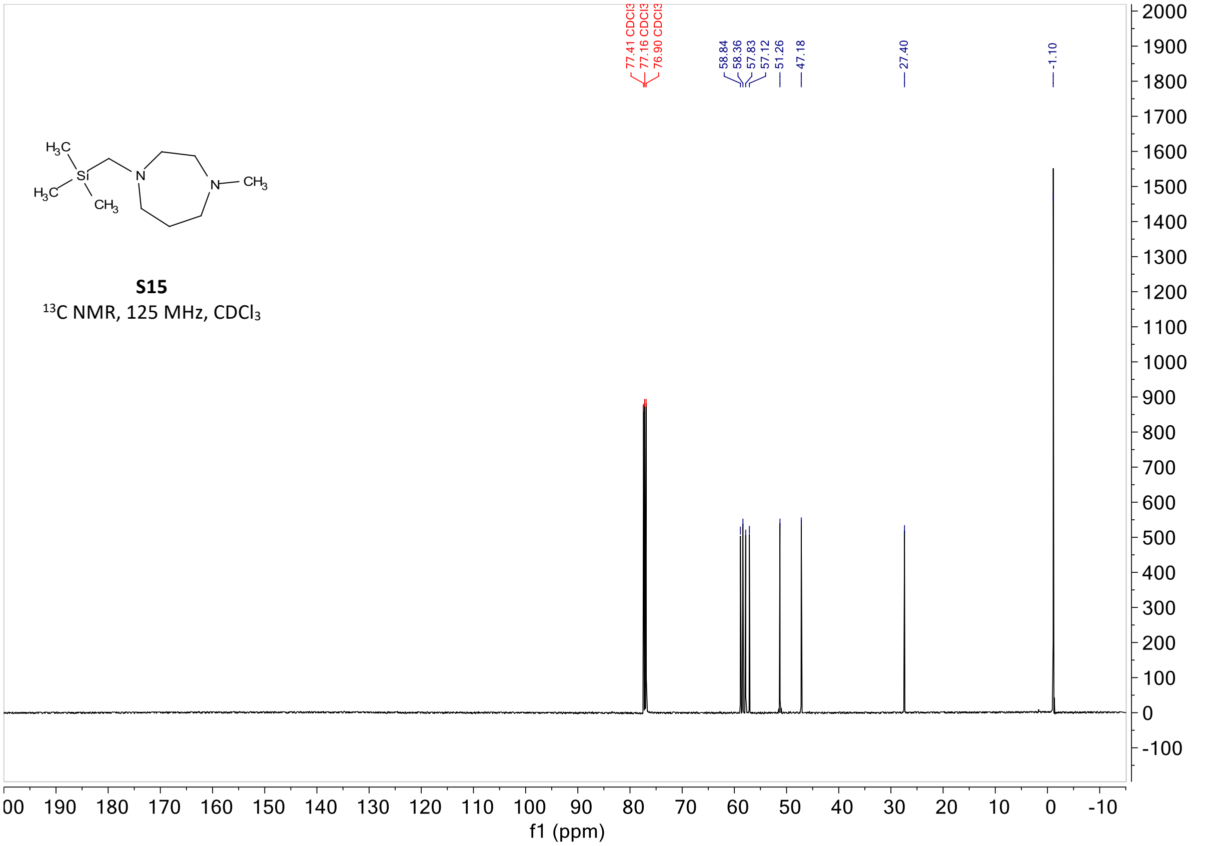

Supplementary Figure 45. ^{13}C NMR spectra of compound **S15**

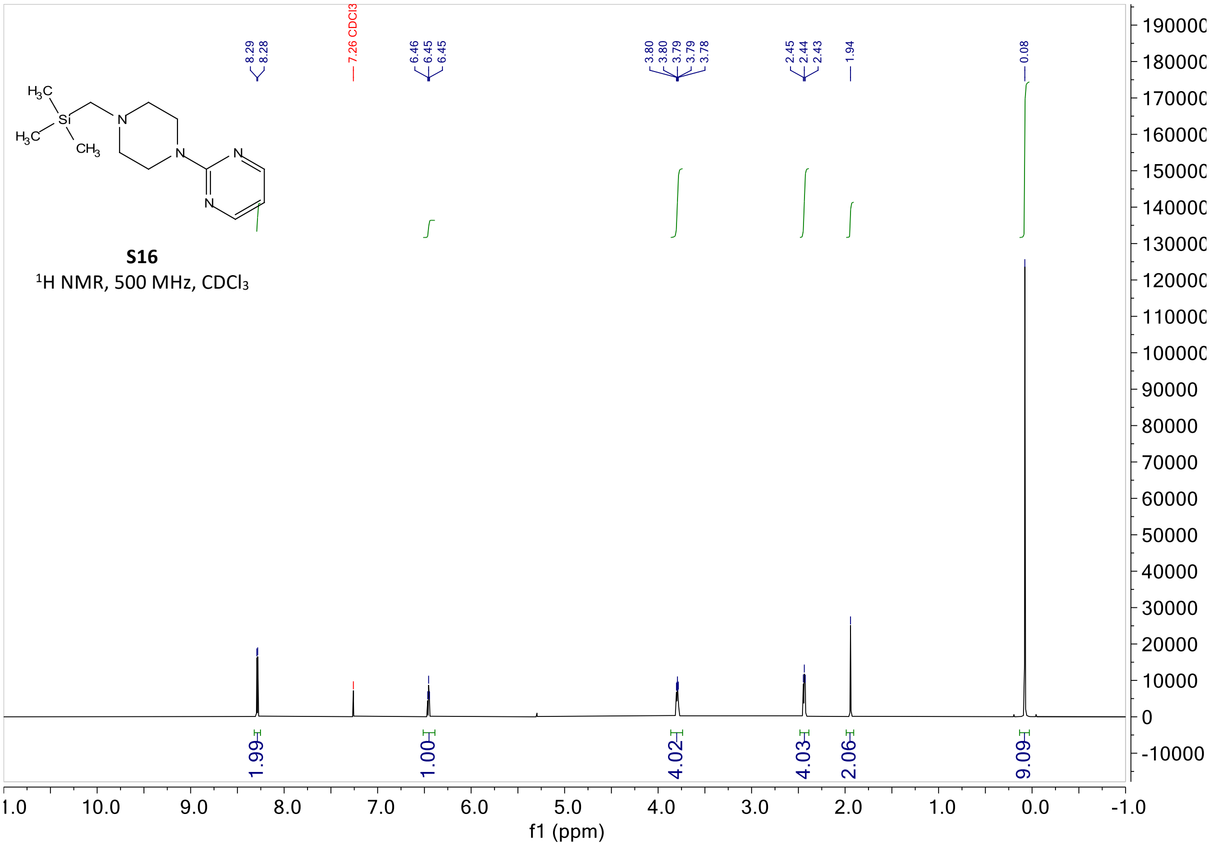

Supplementary Figure 46. ^{1}H NMR spectra of compound **S16**

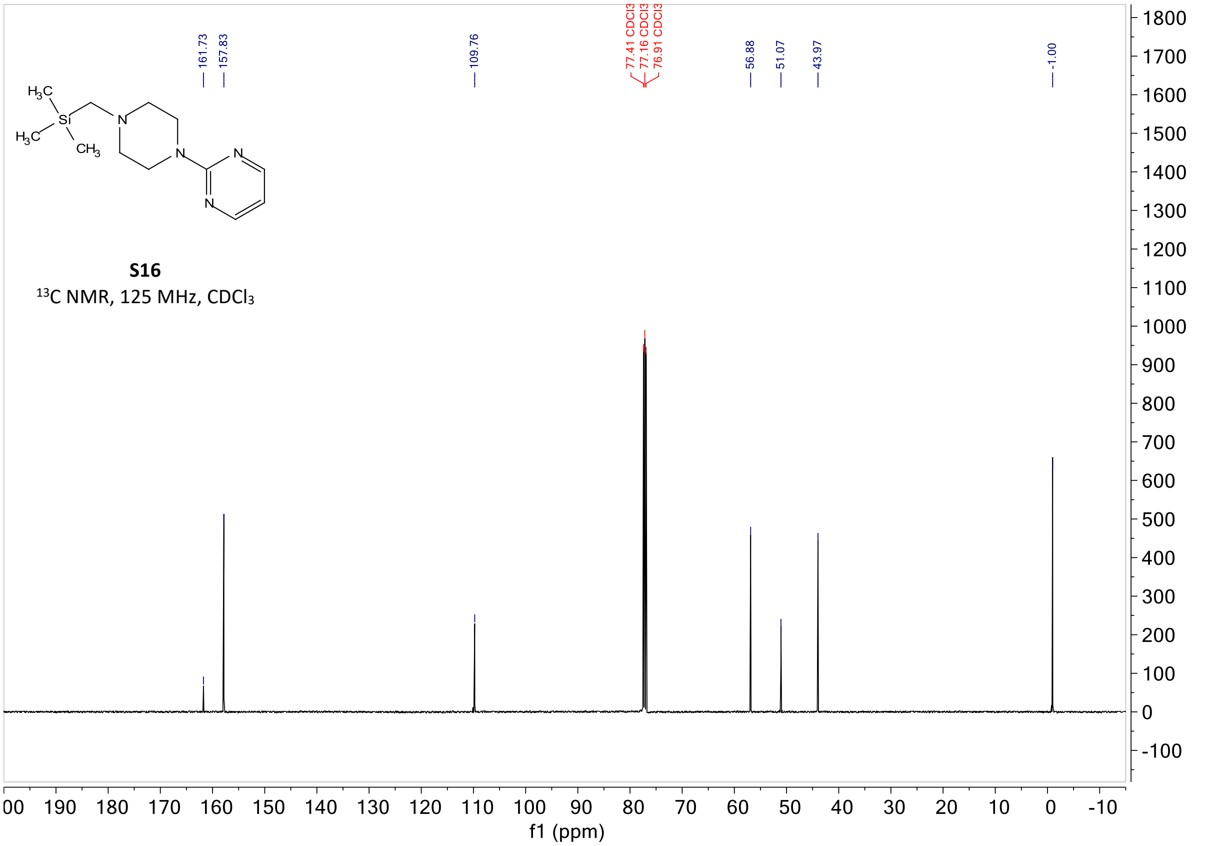

Supplementary Figure 47. ^{13}C NMR spectra of compound **S16**

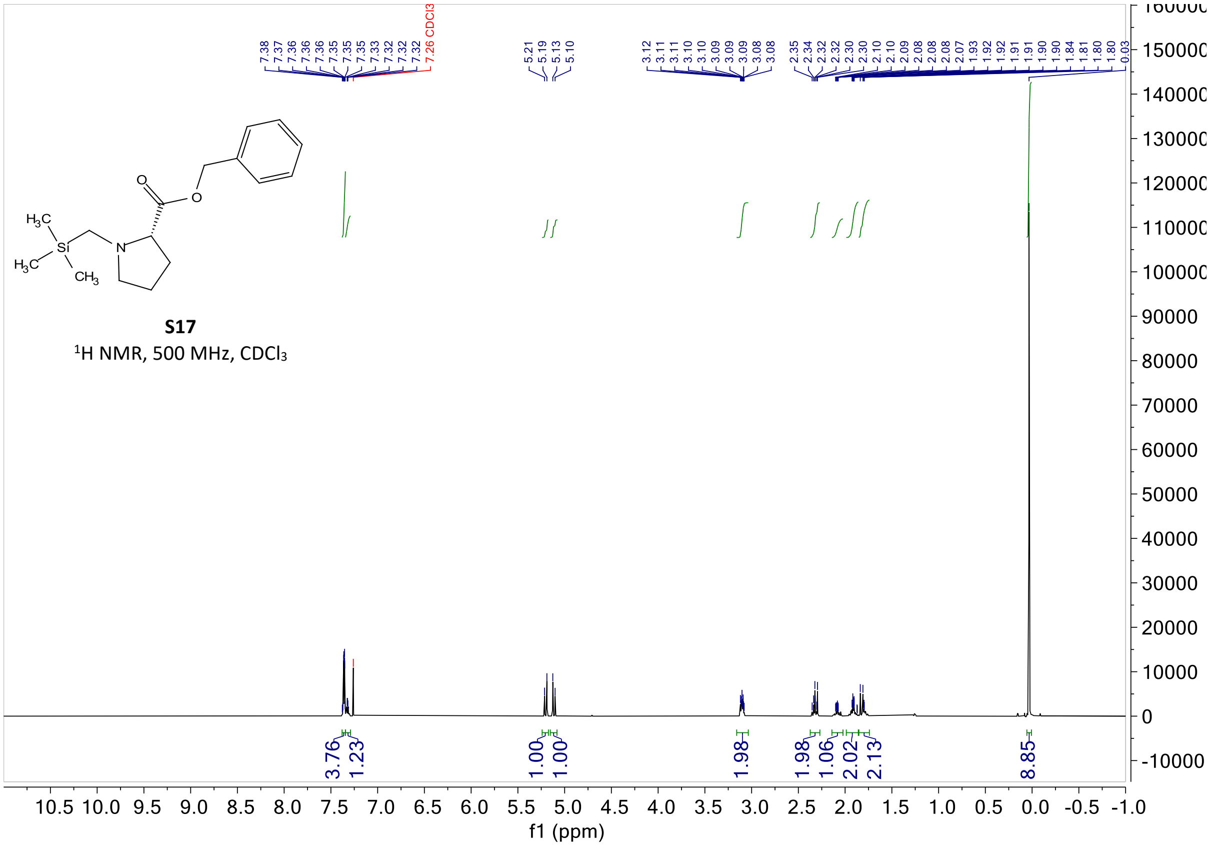

Supplementary Figure 48. ^{1}H NMR spectra of compound **S17**

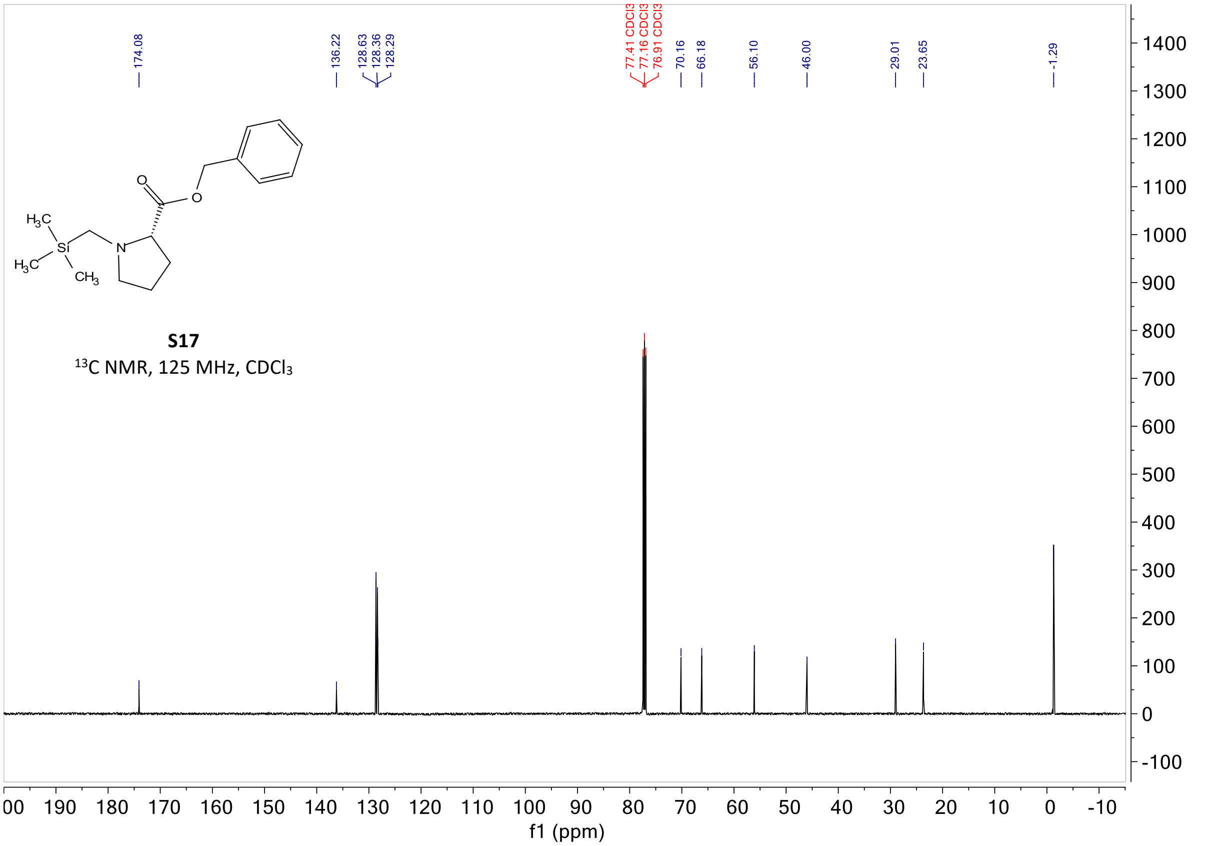

Supplementary Figure 49. ^{13}C NMR spectra of compound **S17**

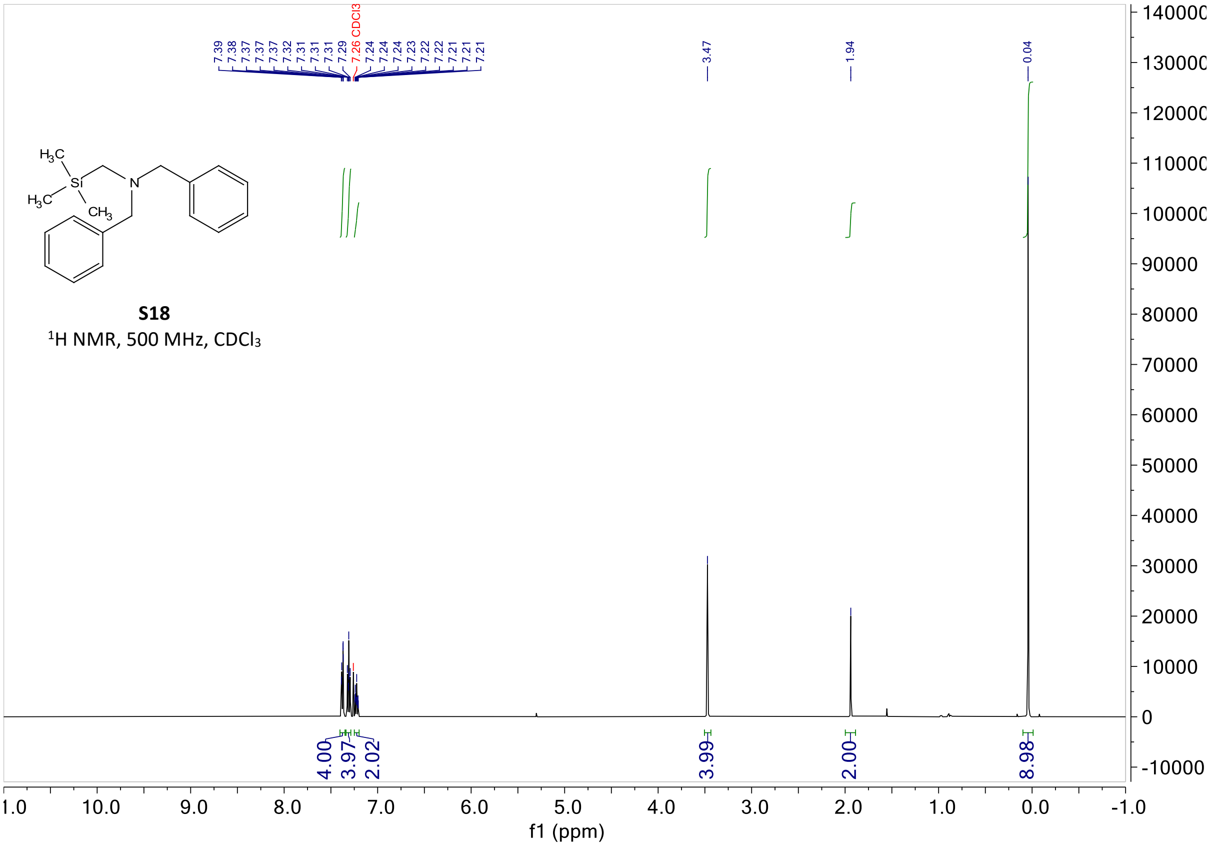

Supplementary Figure 50. ^{1}H NMR spectra of compound **S18**

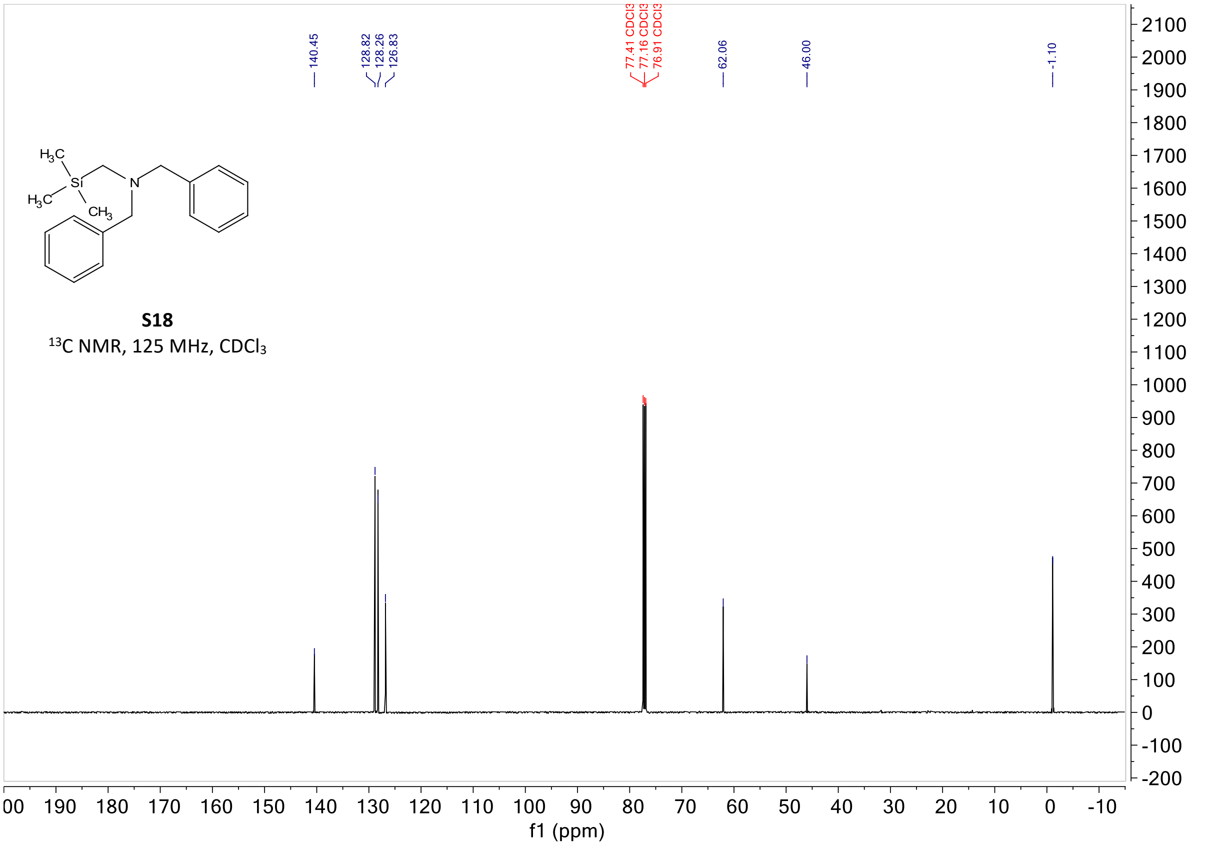

Supplementary Figure 51. ^{13}C NMR spectra of compound **S18**

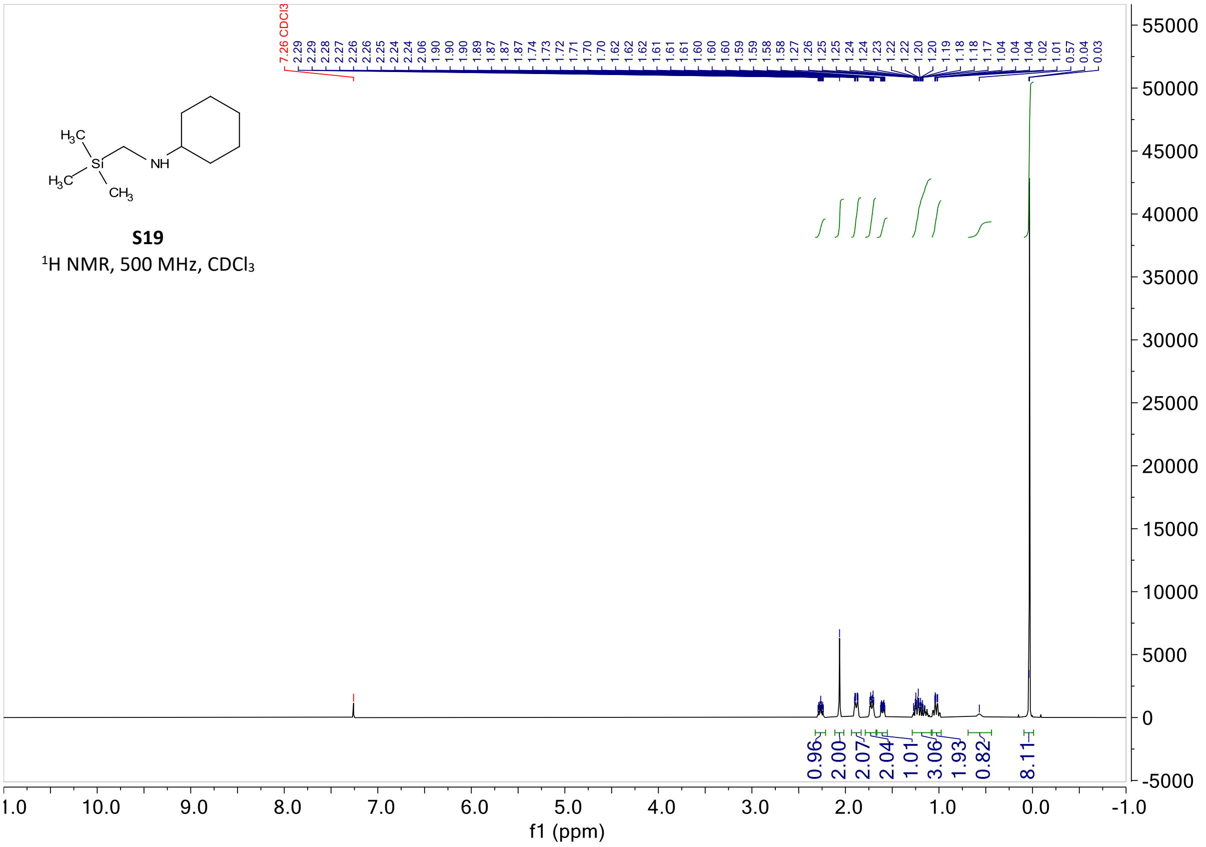

Supplementary Figure 52. ^{1}H NMR spectra of compound **S19**

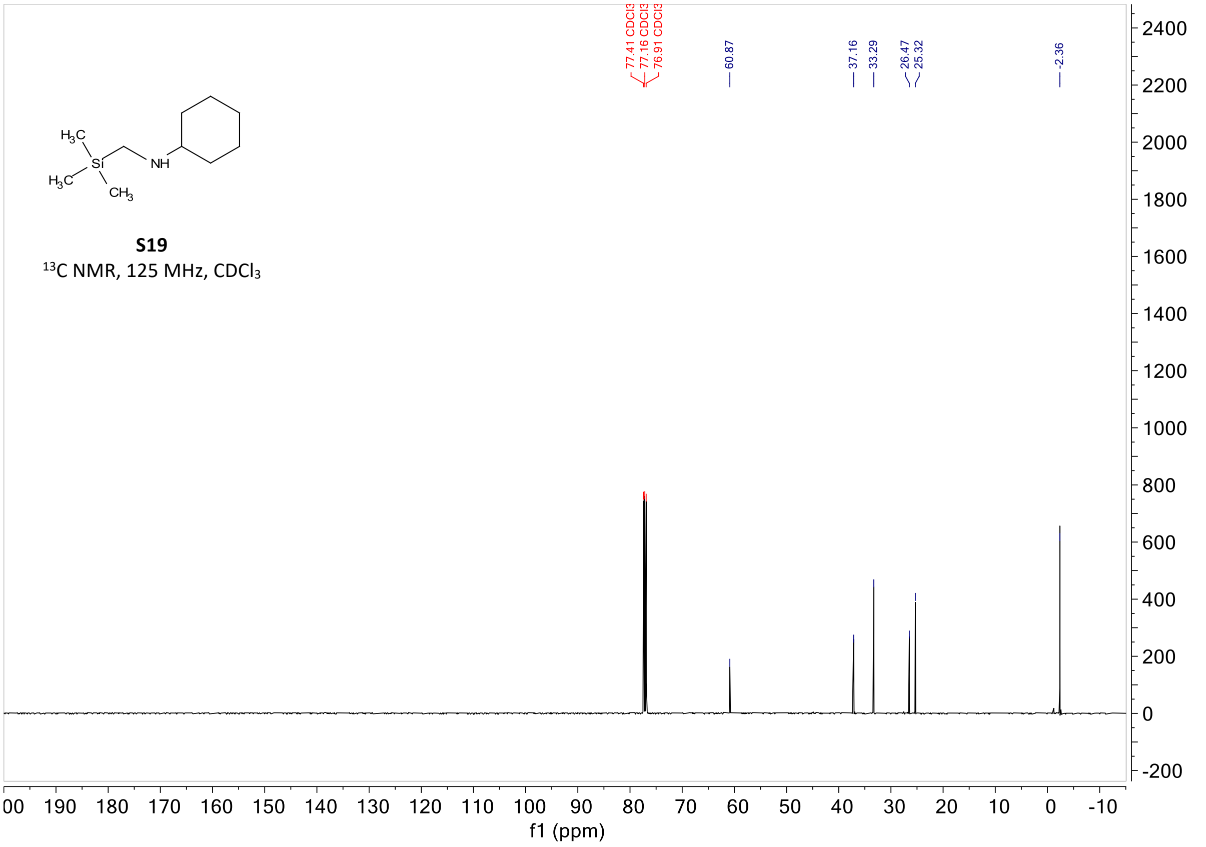

Supplementary Figure 53. ^{13}C NMR spectra of compound **S19**

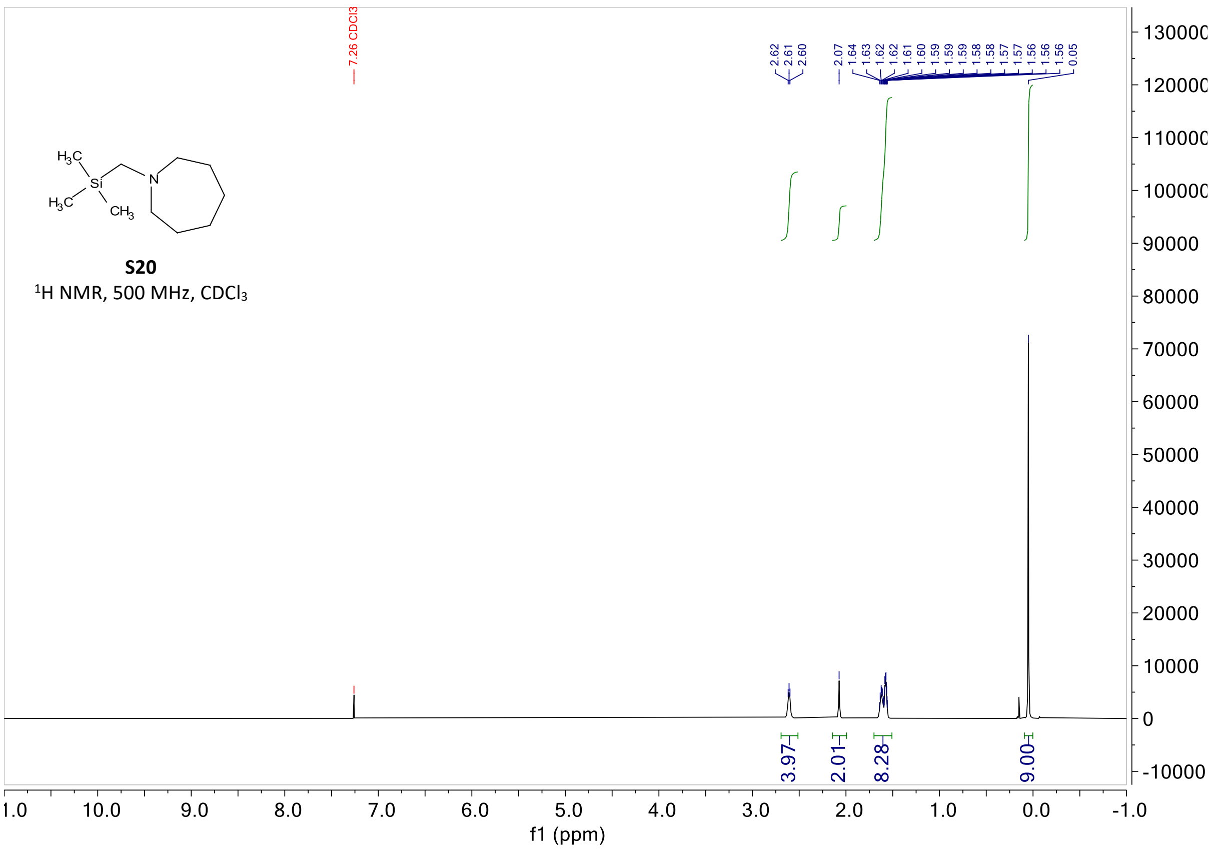

Supplementary Figure 54. ^{1}H NMR spectra of compound **S20**

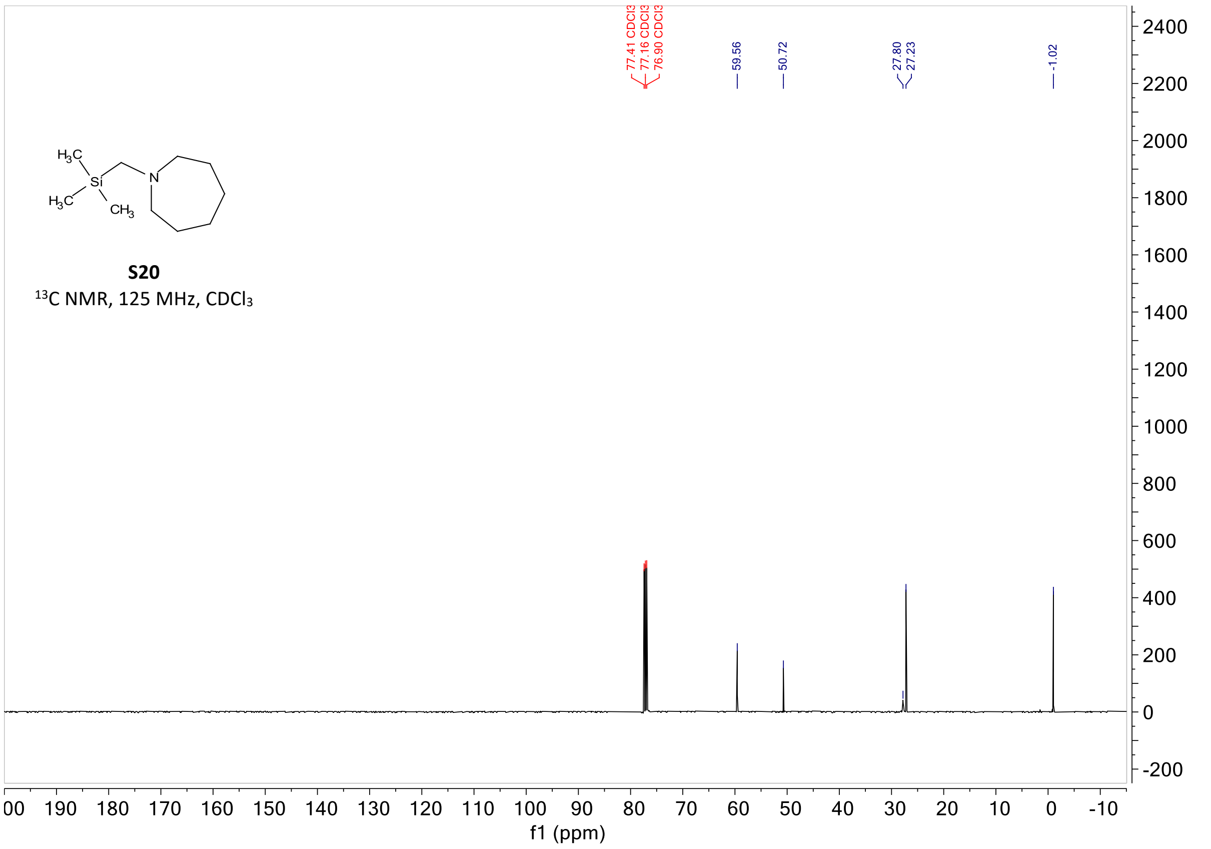

Supplementary Figure 55. ^{13}C NMR spectra of compound **S20**

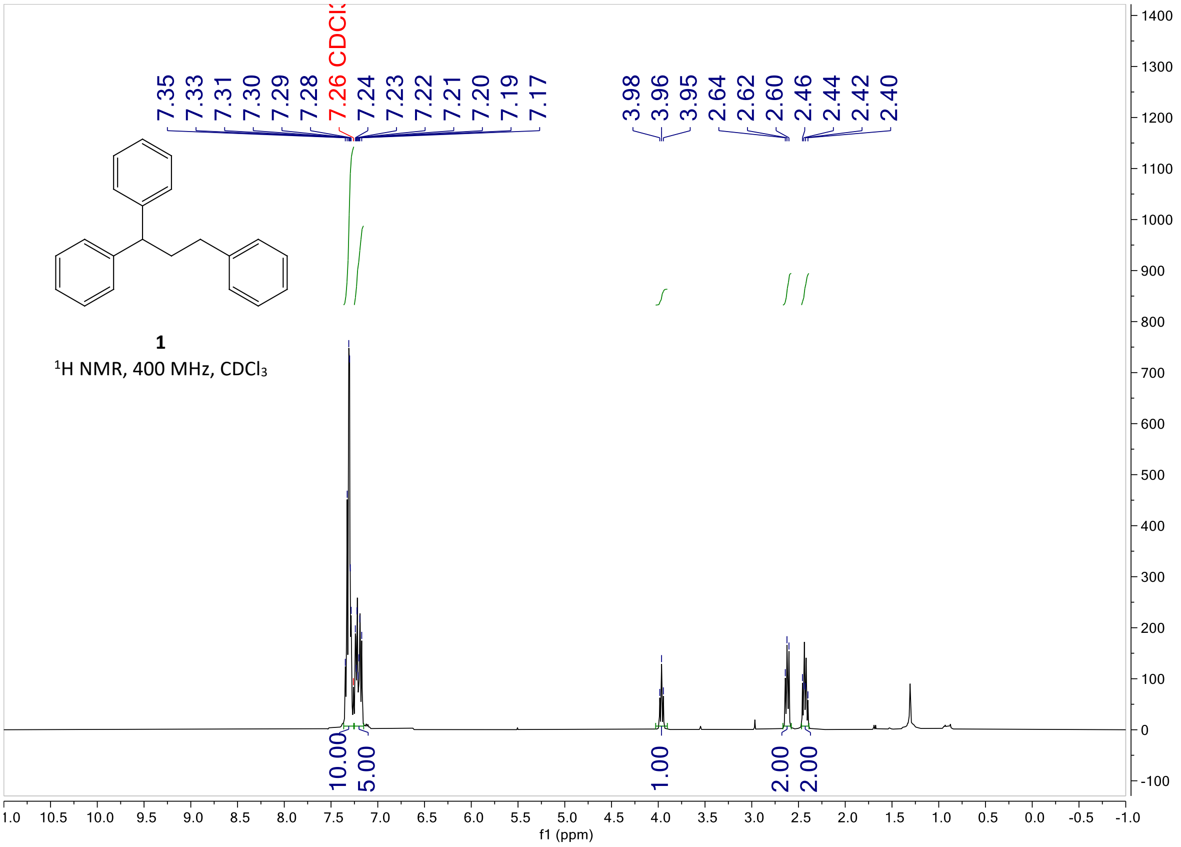

Supplementary Figure 56. ^{1}H NMR spectra of compound **1**

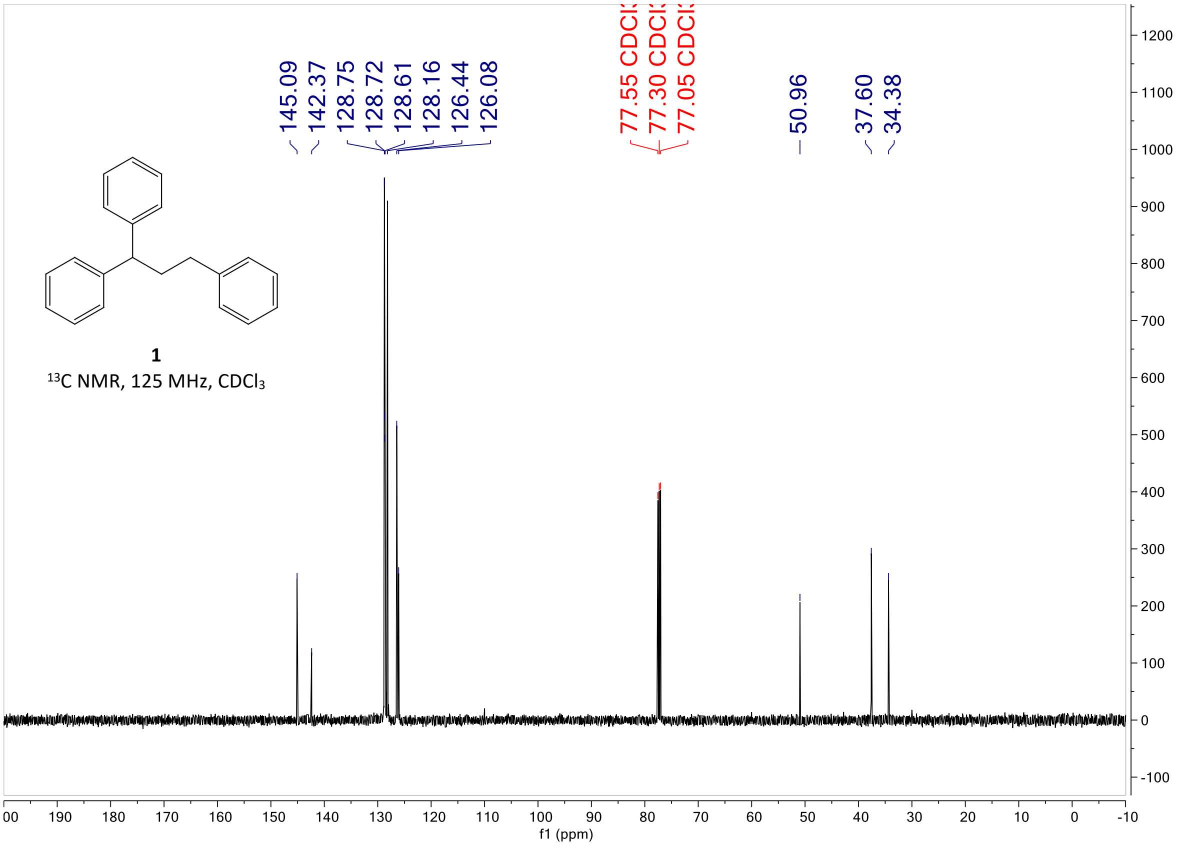

Supplementary Figure 57. ^{13}C NMR spectra of compound **1**

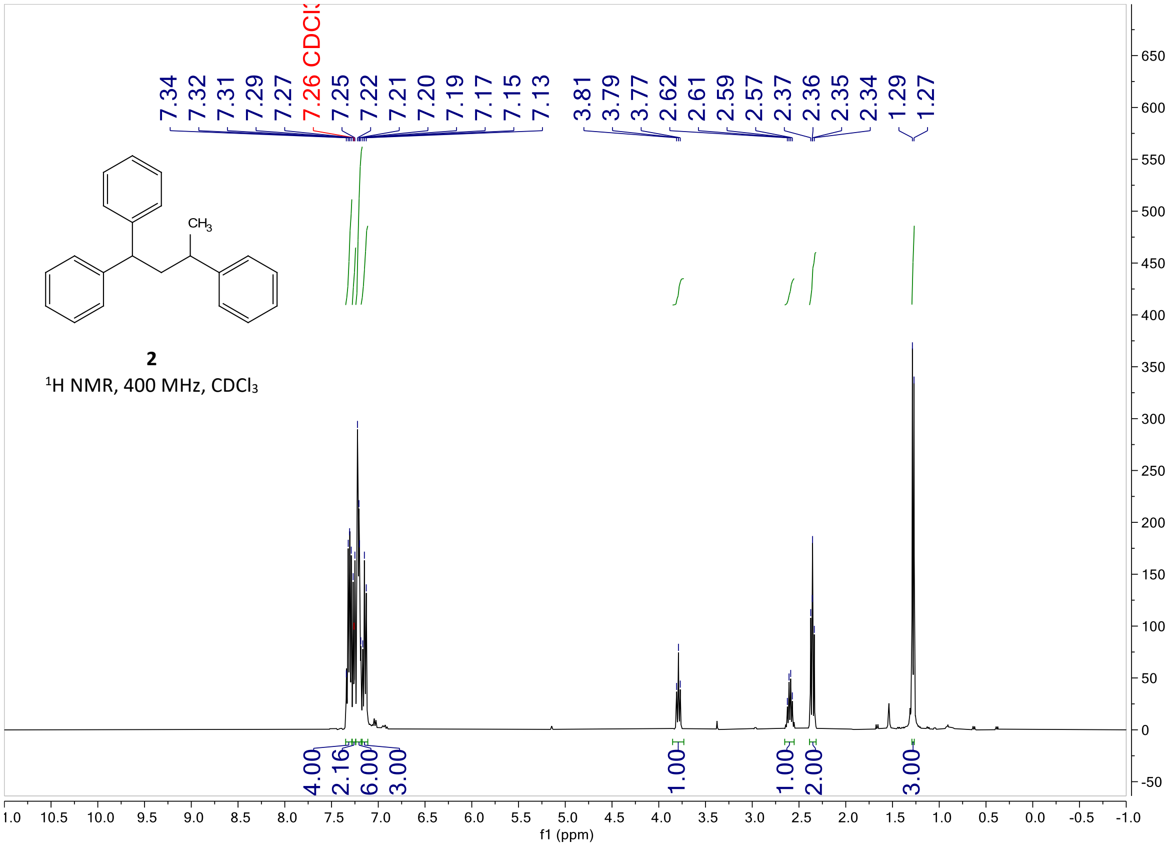

Supplementary Figure 58. ^{1}H NMR spectra of compound **2**

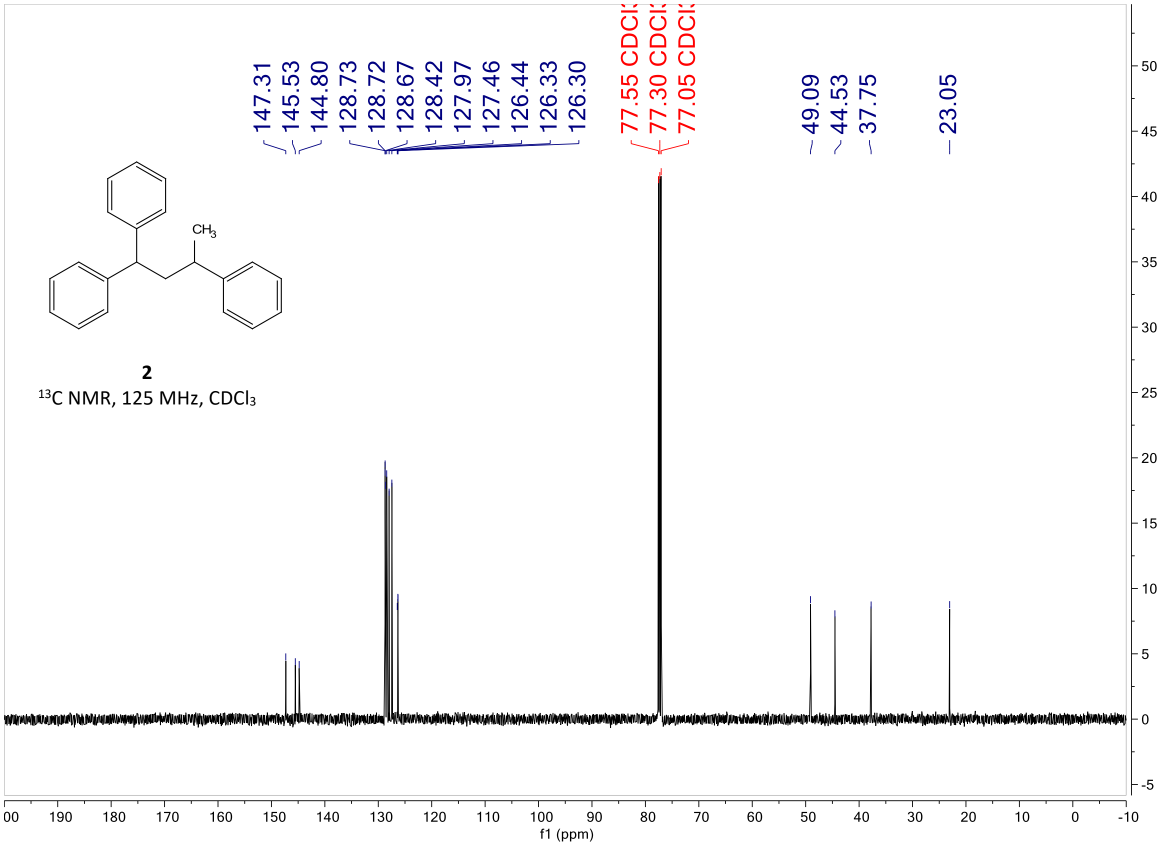

Supplementary Figure 59. ^{13}C NMR spectra of compound **2**

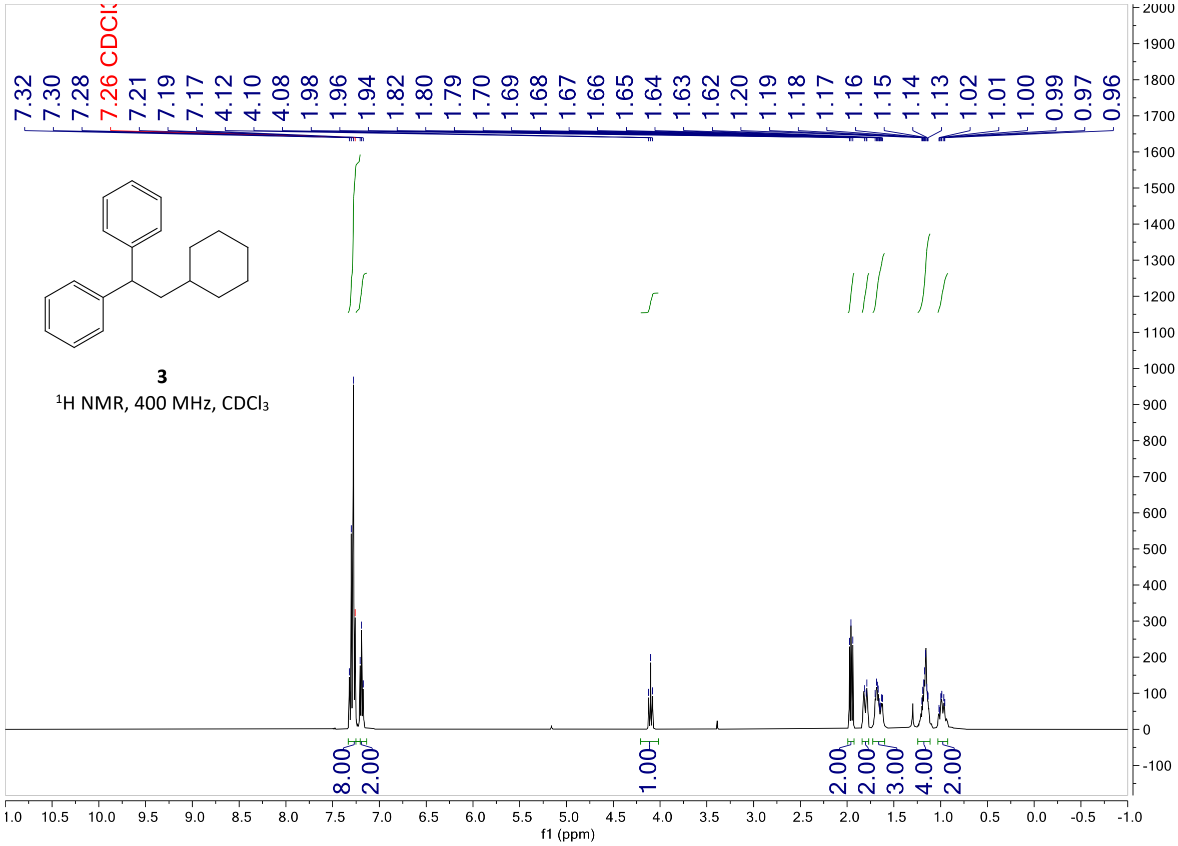

Supplementary Figure 60. ^{1}H NMR spectra of compound **3**

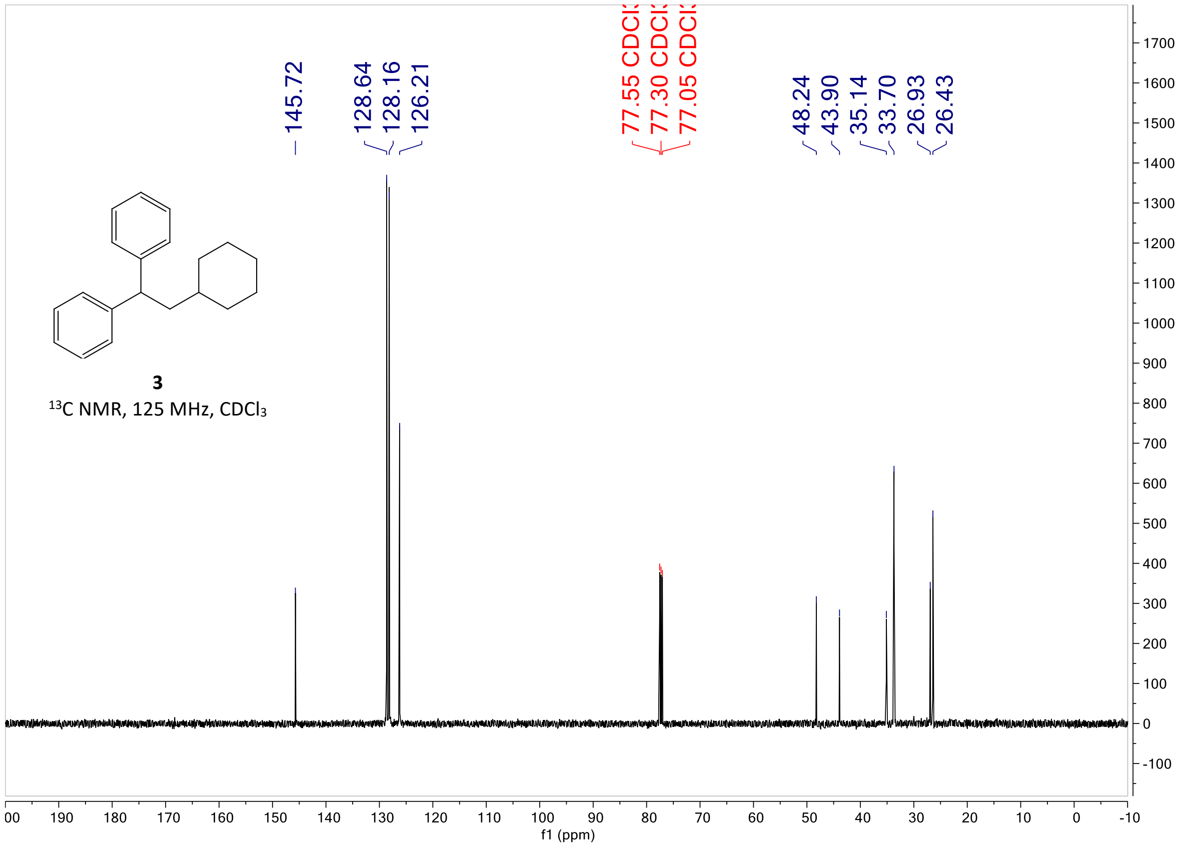

Supplementary Figure 61. ^{13}C NMR spectra of compound **3**

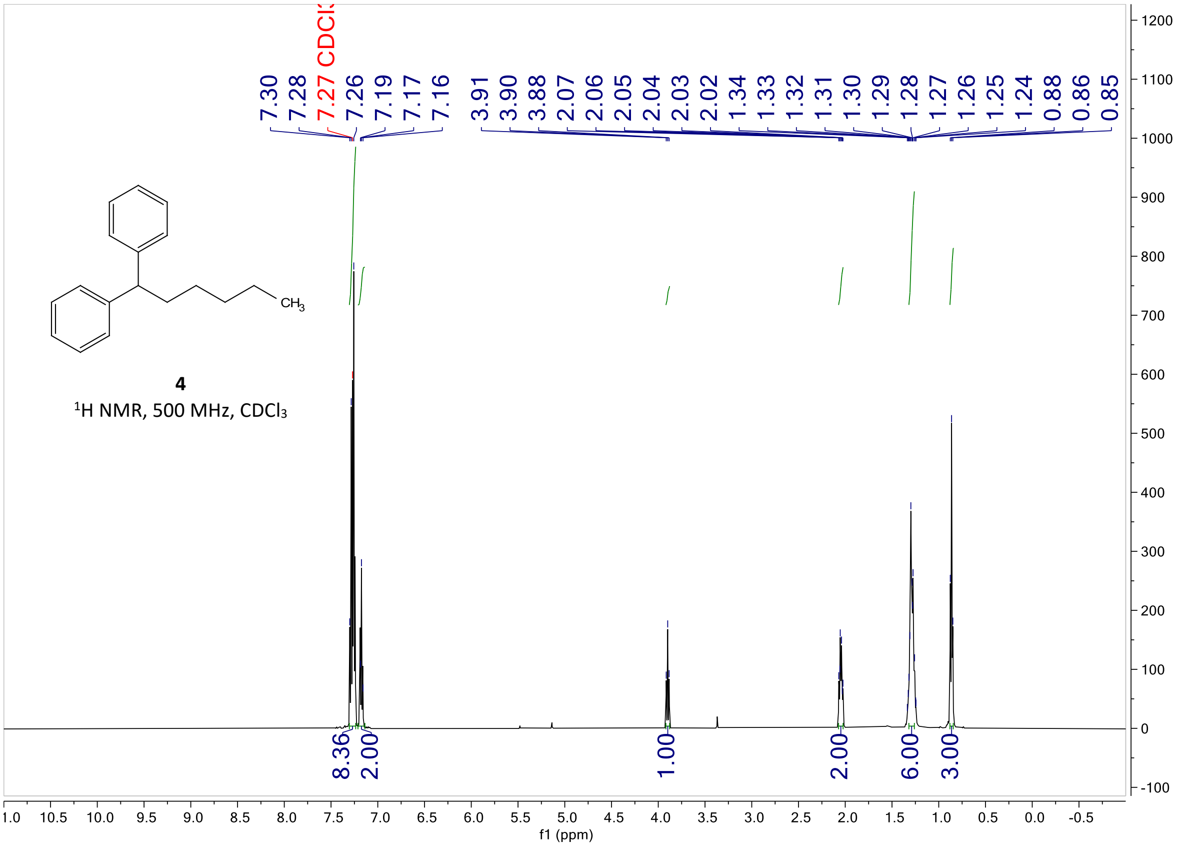

Supplementary Figure 62. ^{1}H NMR spectra of compound **4**

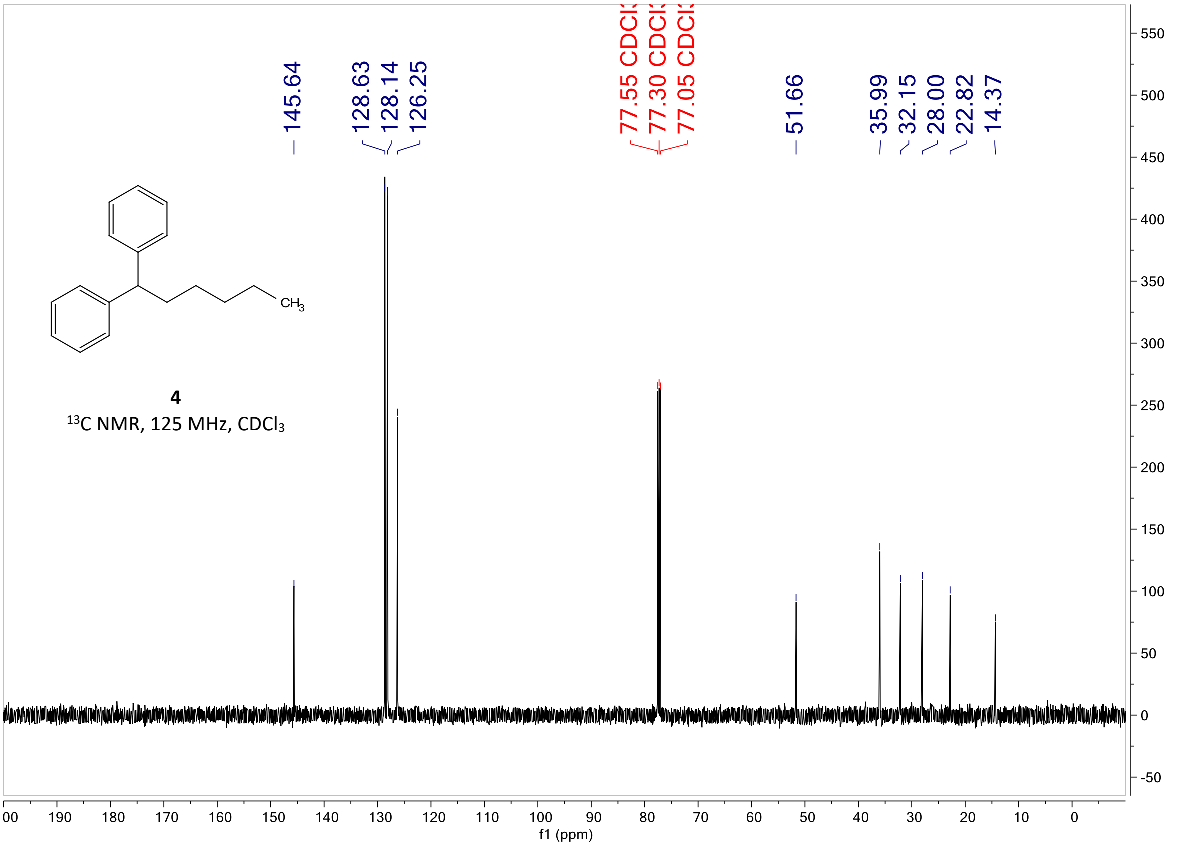

Supplementary Figure 63. ^{13}C NMR spectra of compound **4**

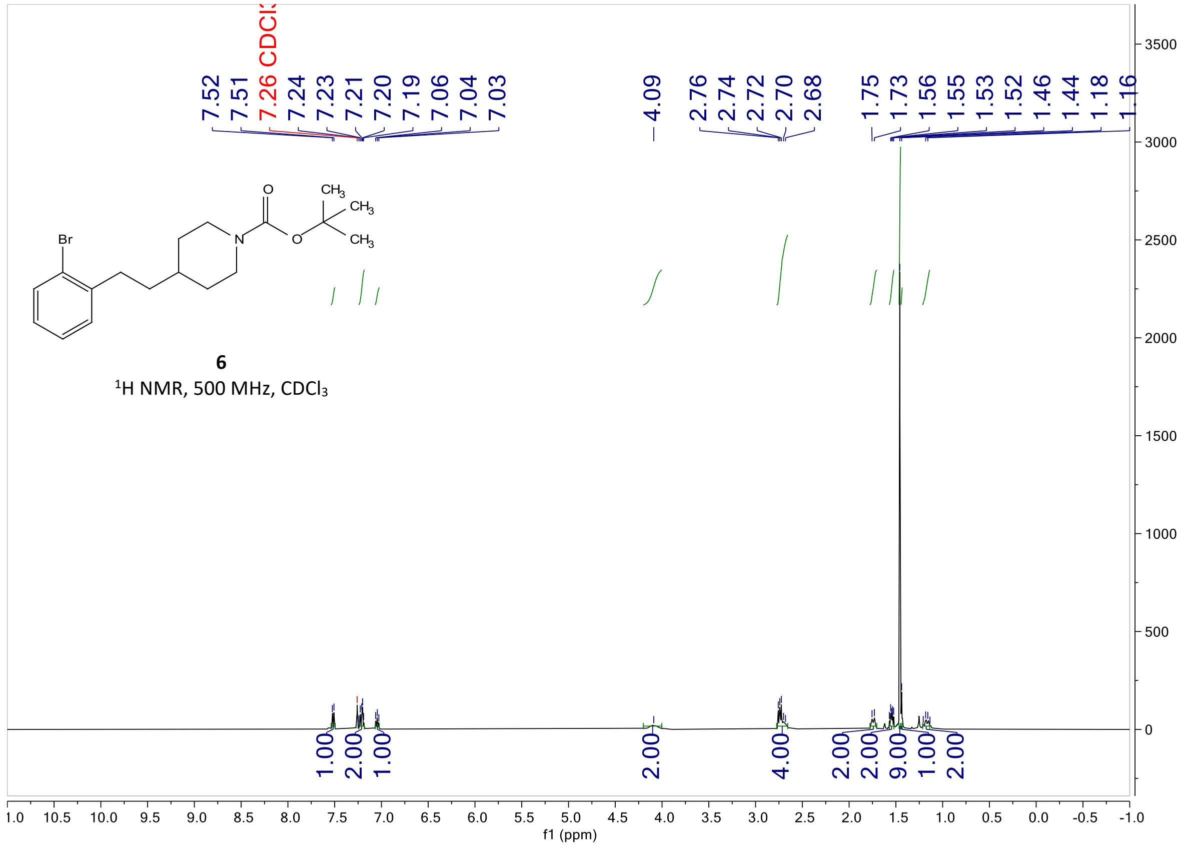

Supplementary Figure 64. ^{1}H NMR spectra of compound **6**

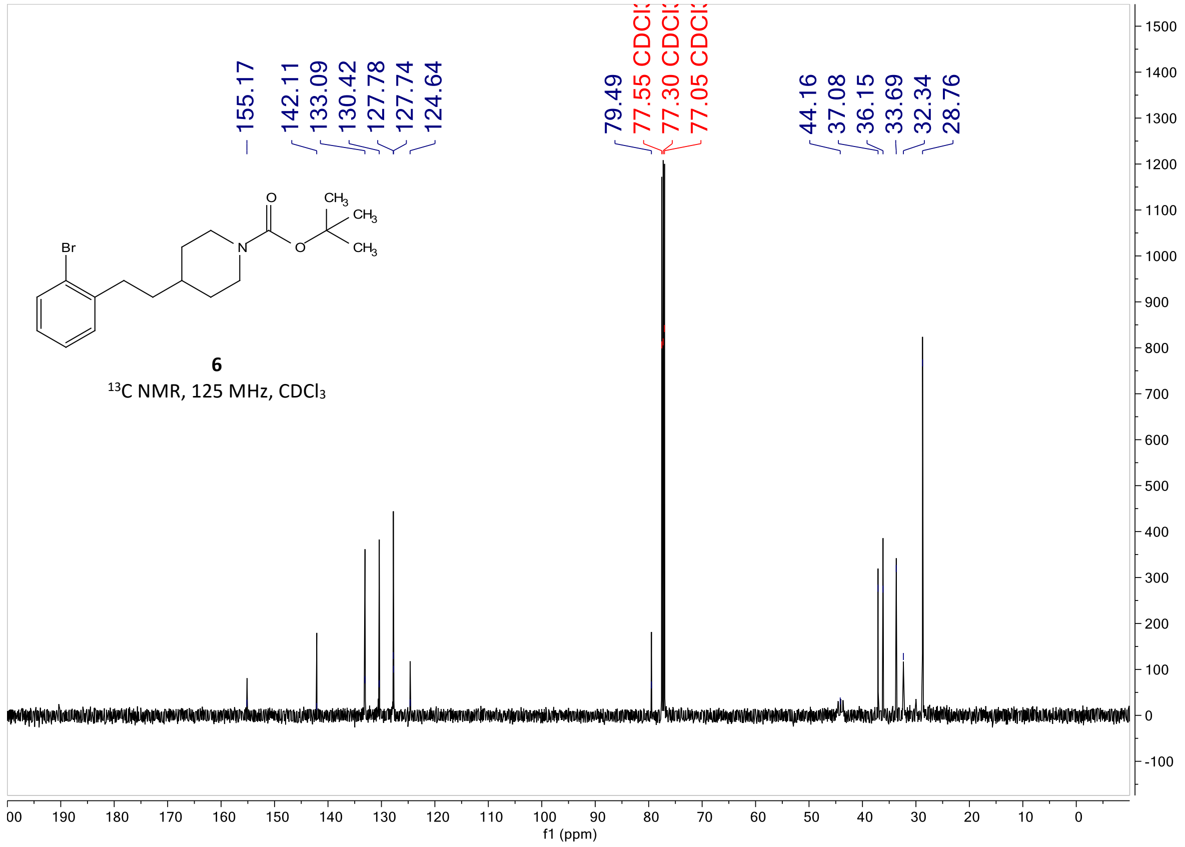

Supplementary Figure 65. ^{13}C NMR spectra of compound **6**

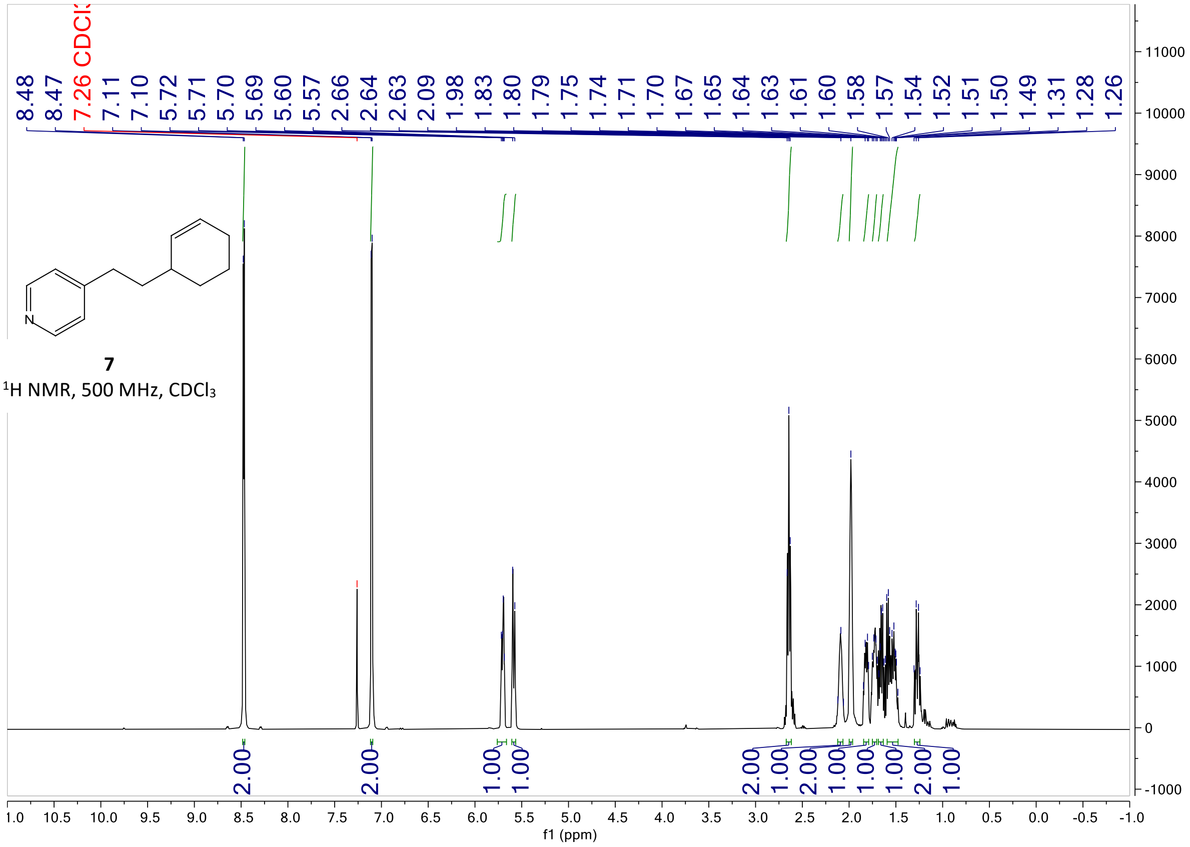

Supplementary Figure 66. ^{1}H NMR spectra of compound **7**

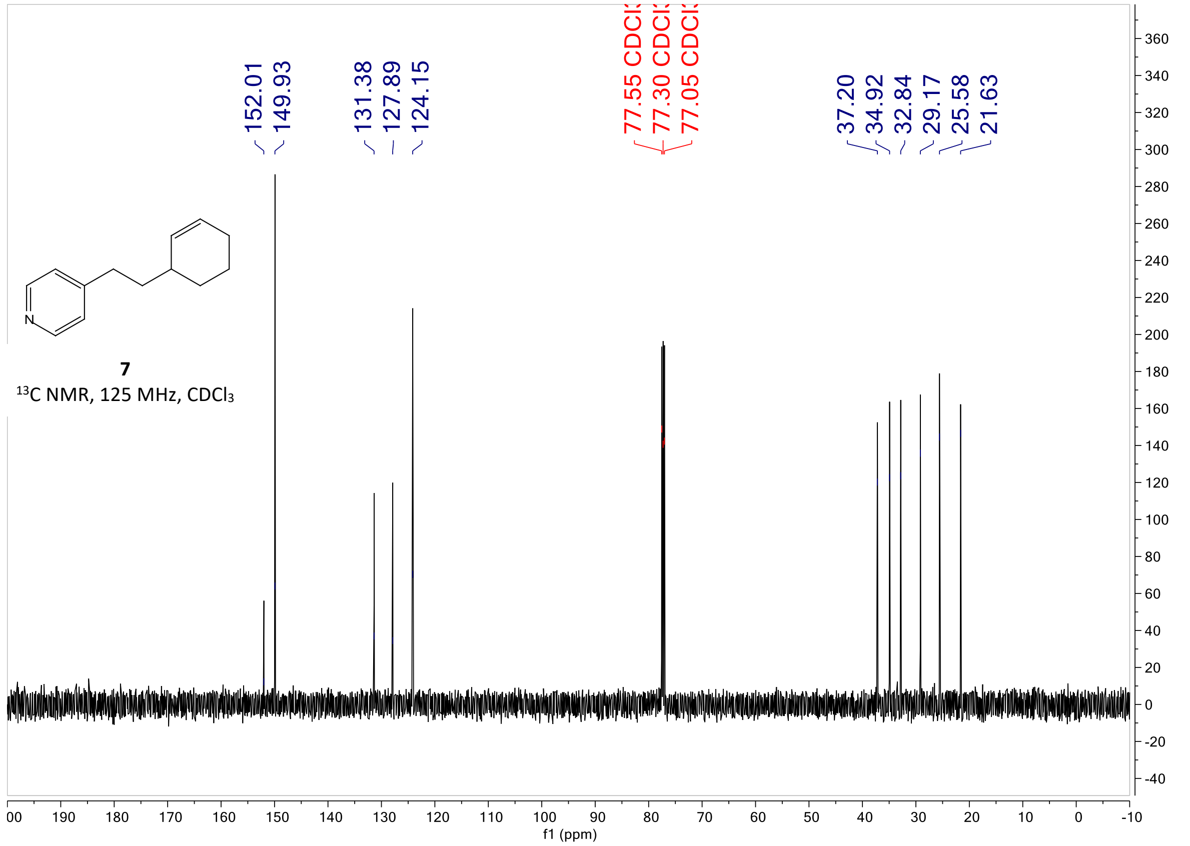

Supplementary Figure 67. ^{13}C NMR spectra of compound **7**

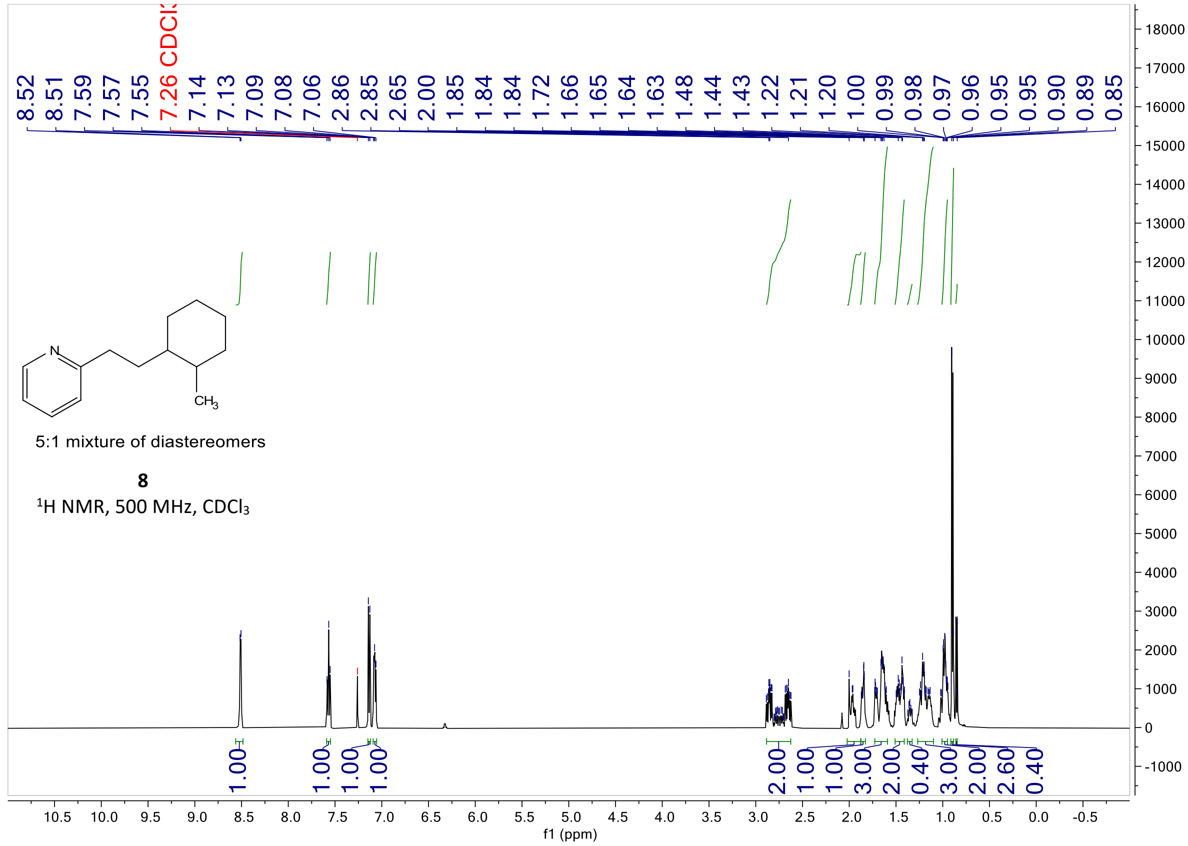

Supplementary Figure 68. ^{1}H NMR spectra of compound **8**

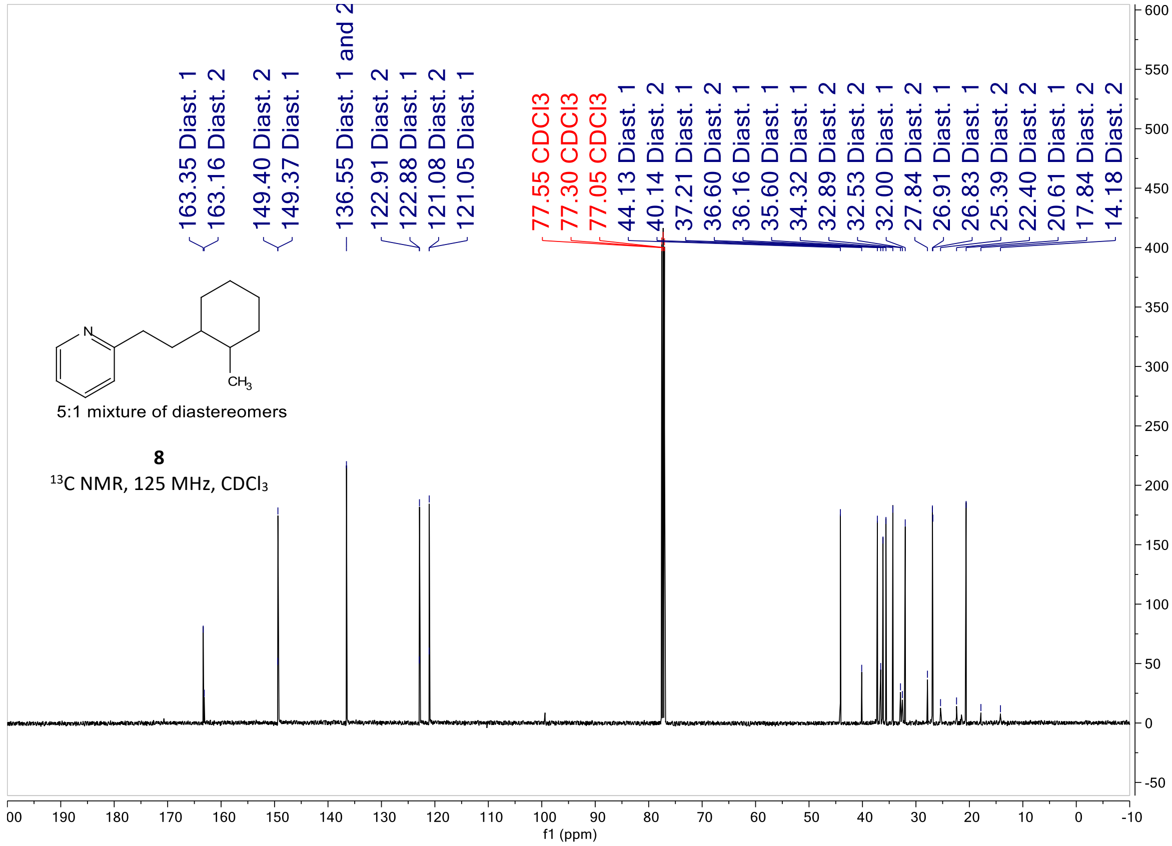

Supplementary Figure 69. ^{13}C NMR spectra of compound **8**

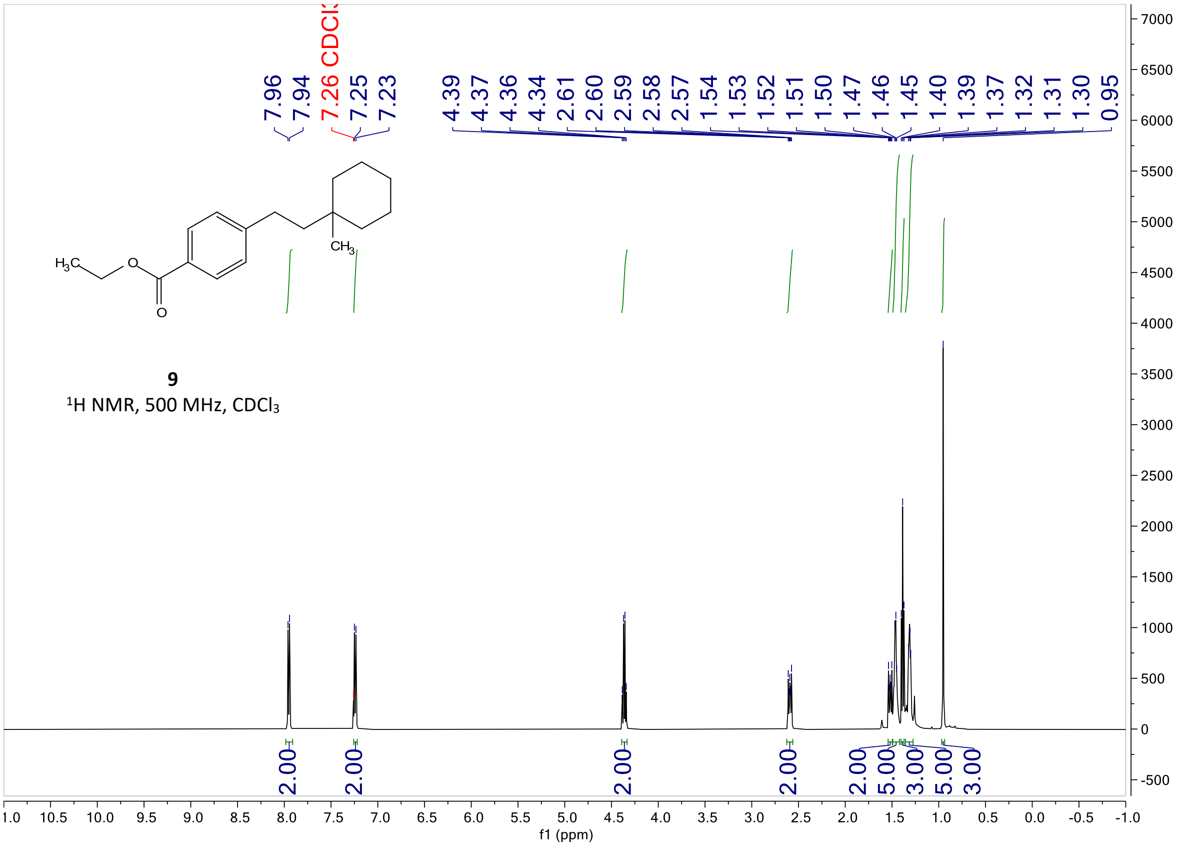

Supplementary Figure 70. ^{1}H NMR spectra of compound **9**

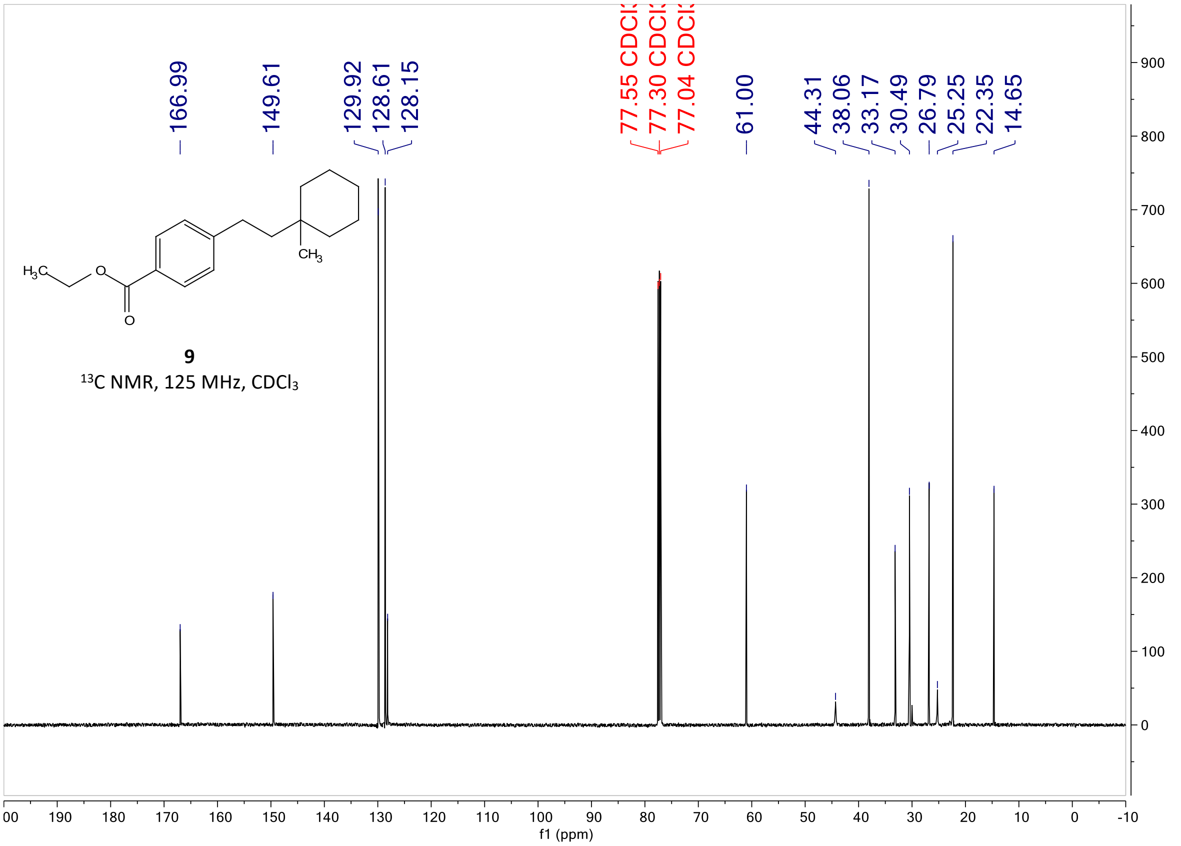

Supplementary Figure 71. ^{13}C NMR spectra of compound **9**

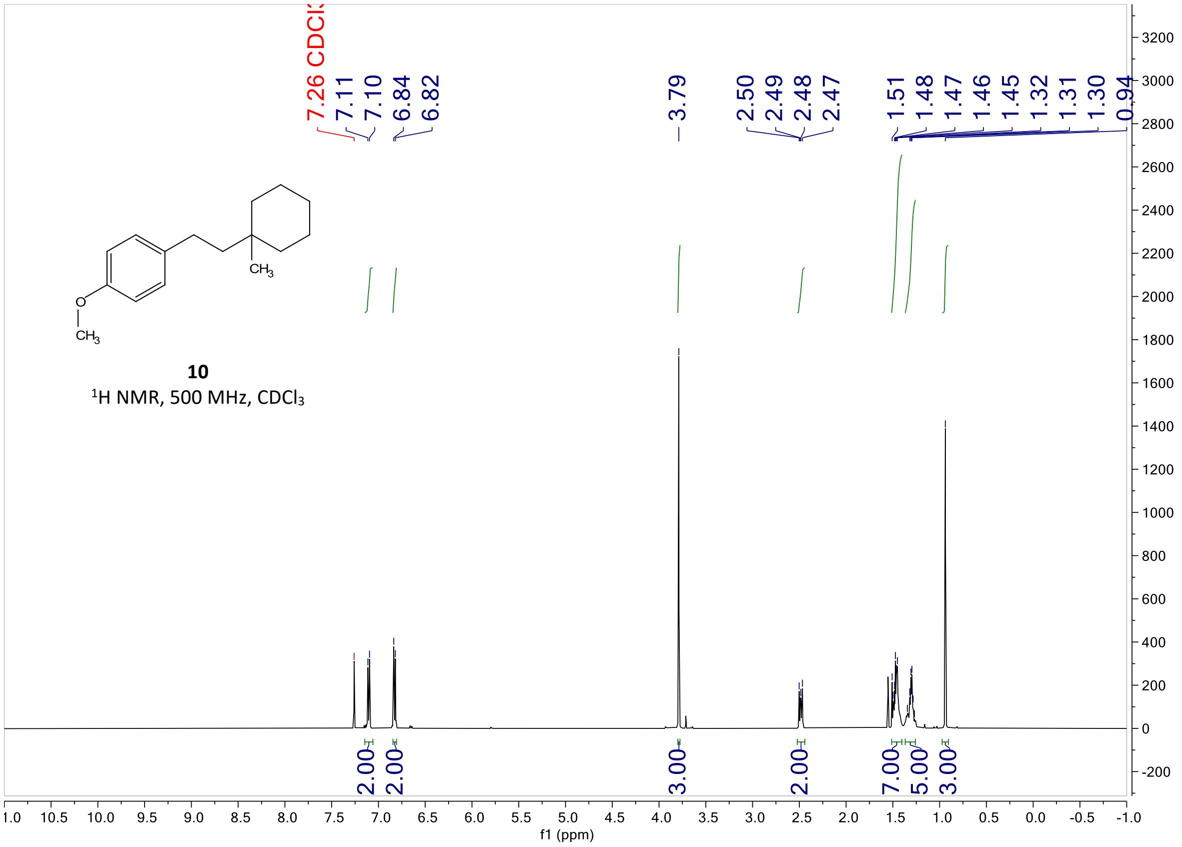

Supplementary Figure 72. ^{1}H NMR spectra of compound **10**

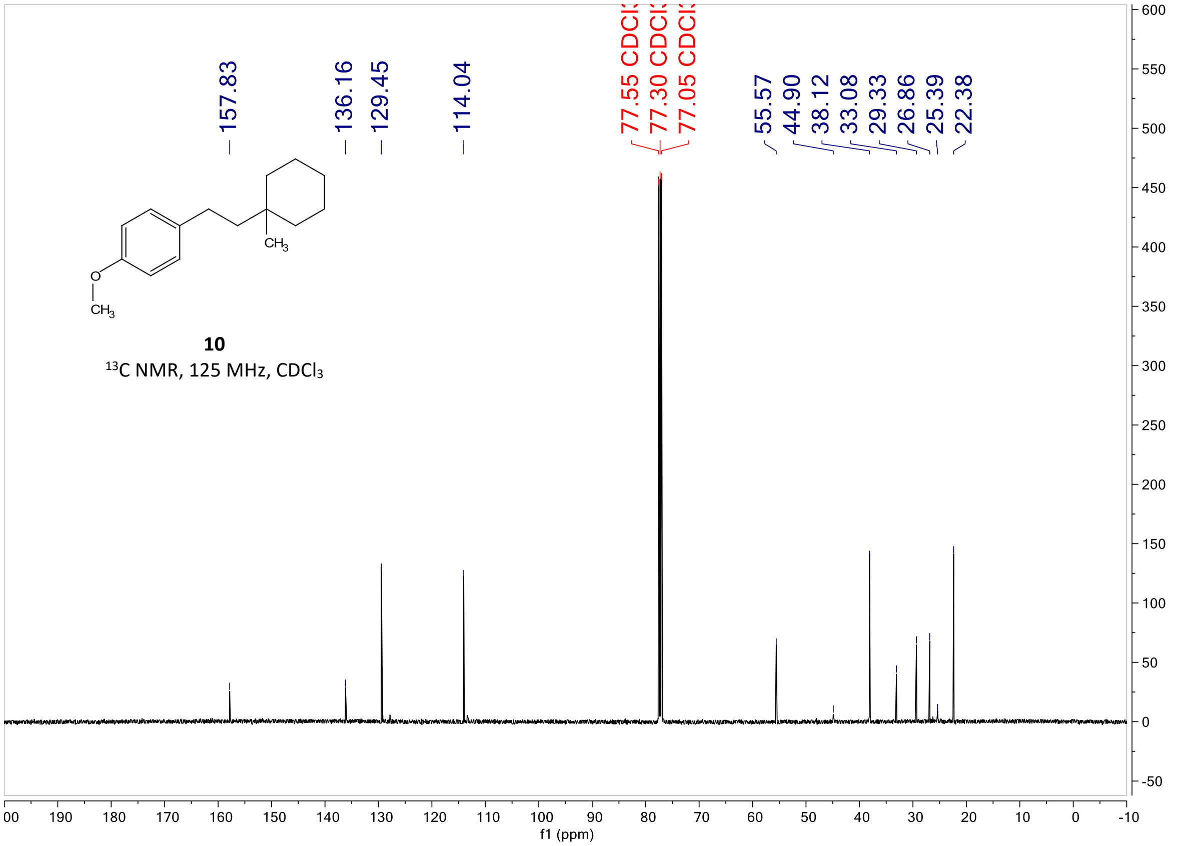

Supplementary Figure 73. ^{13}C NMR spectra of compound **10**

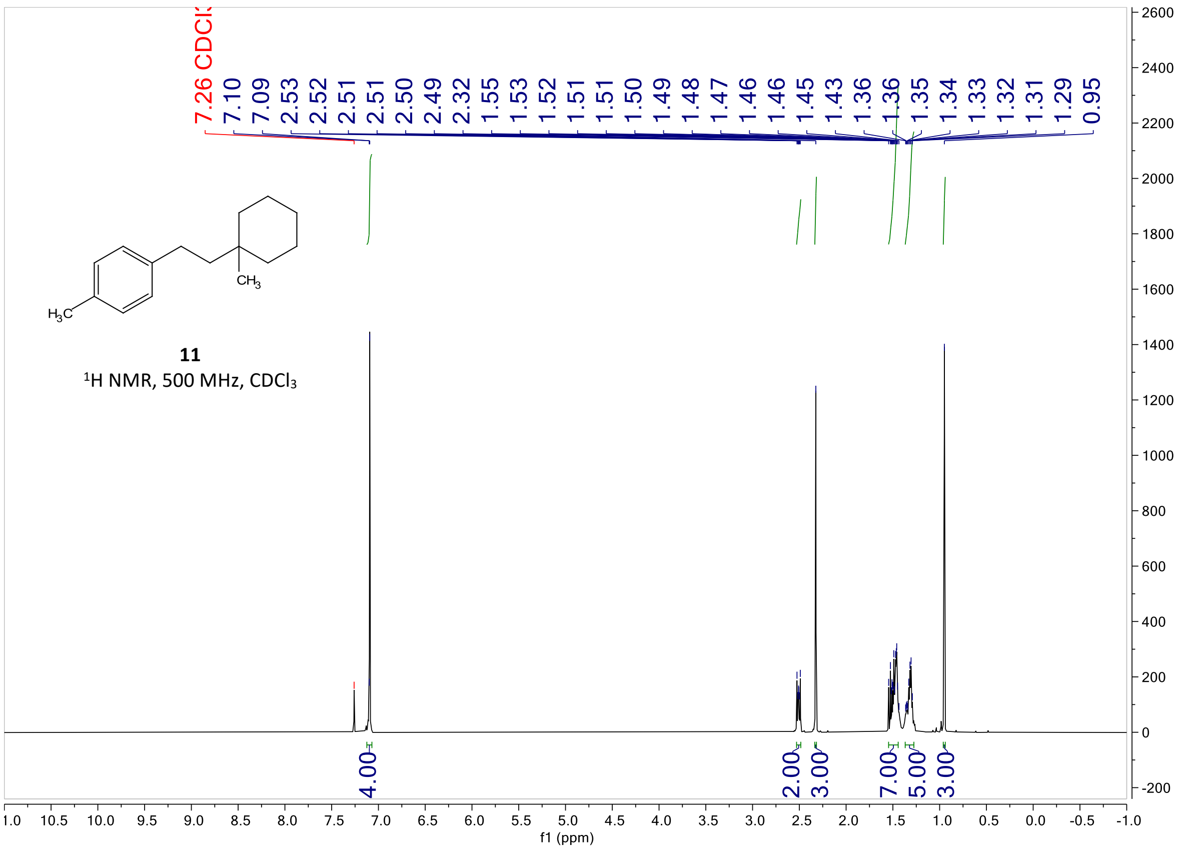

Supplementary Figure 74. ^{1}H NMR spectra of compound **11**

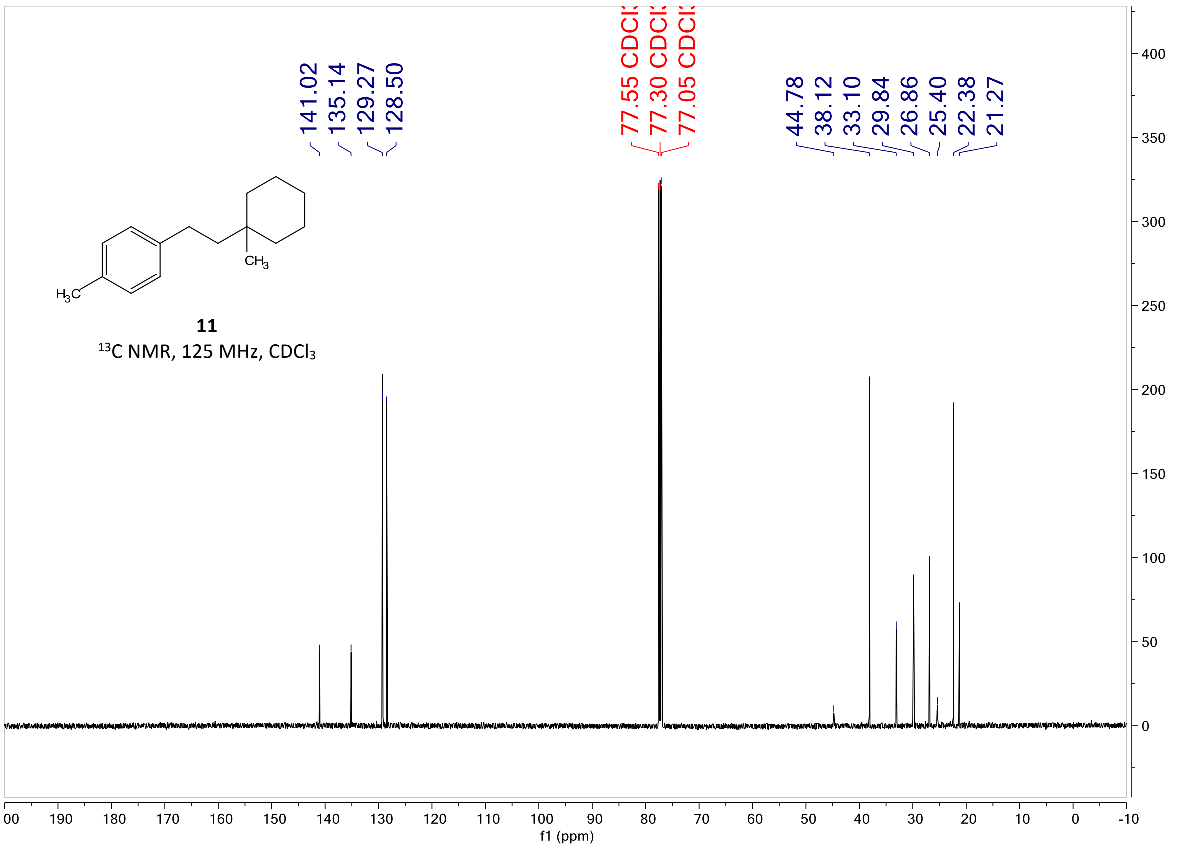

Supplementary Figure 75. ^{13}C NMR spectra of compound **11**

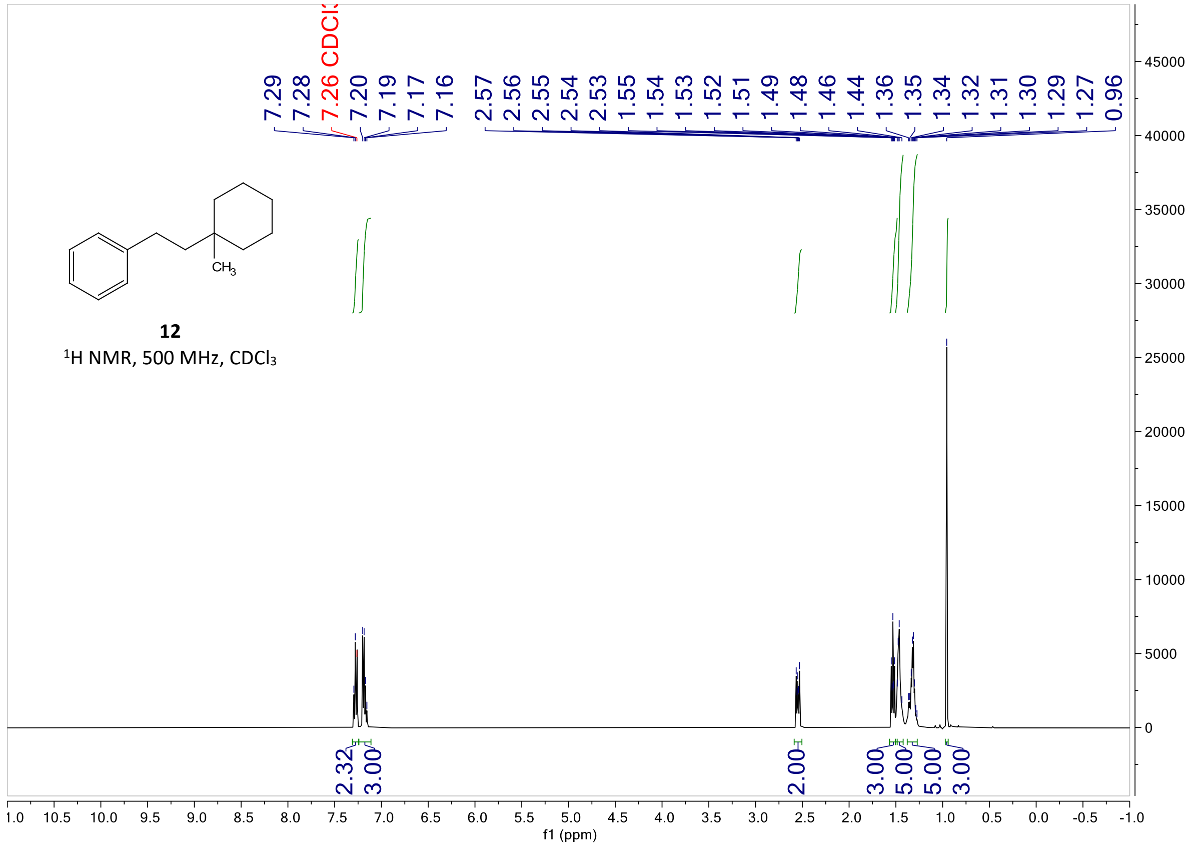

Supplementary Figure 76. ^{1}H NMR spectra of compound **12**

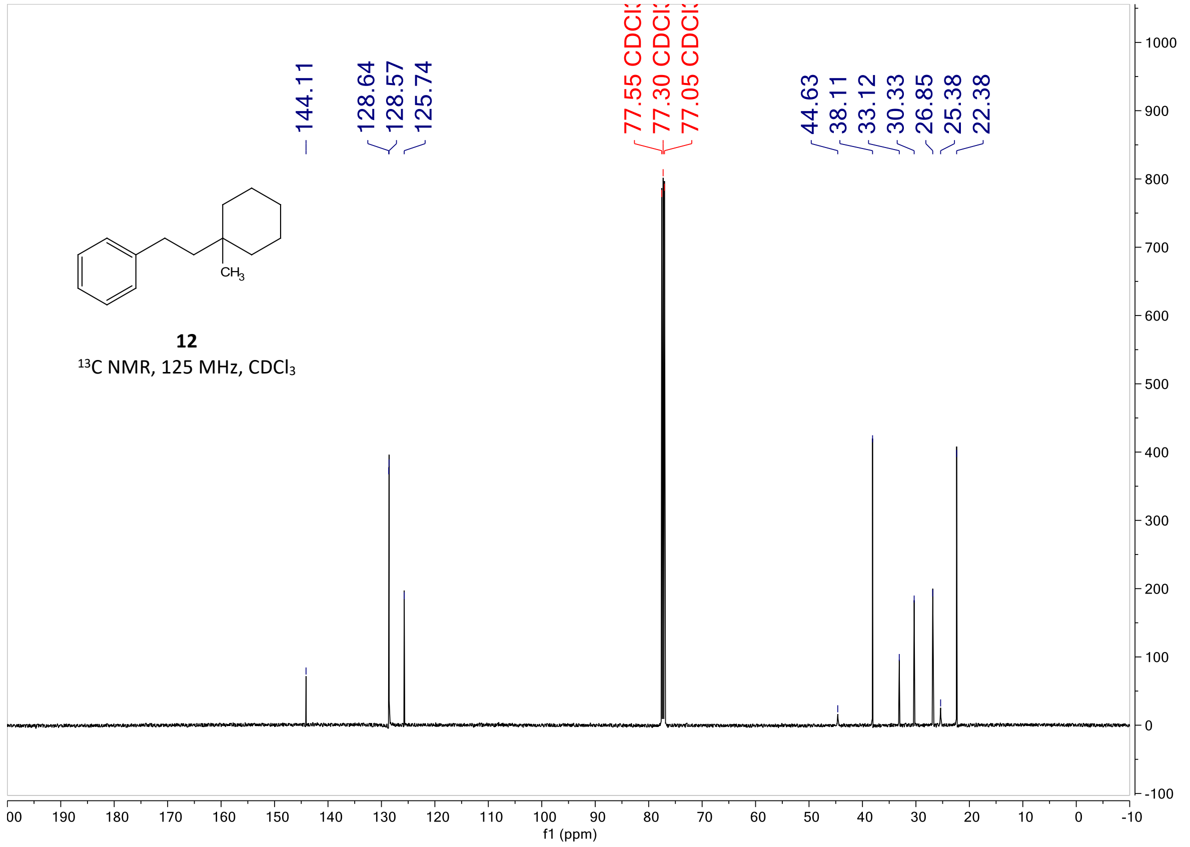

Supplementary Figure 77. ^{13}C NMR spectra of compound **12**

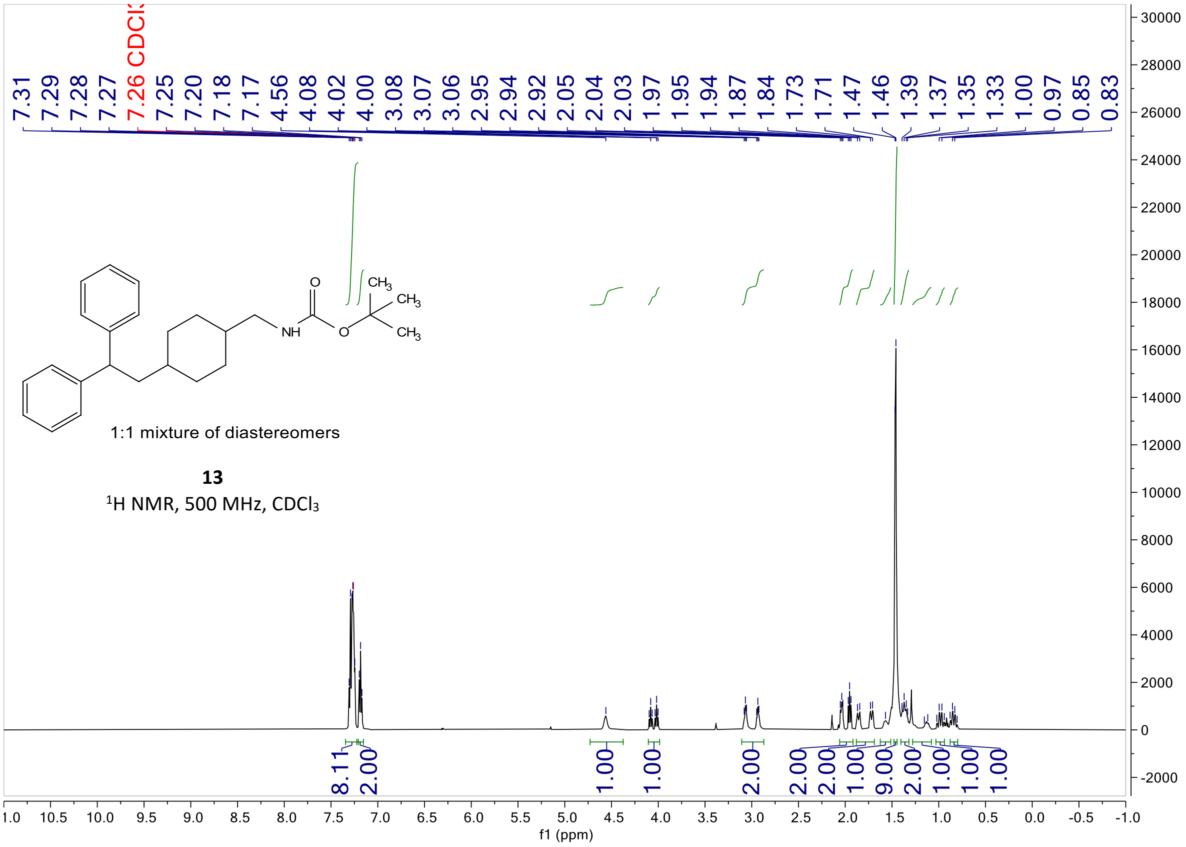

Supplementary Figure 78. ^{1}H NMR spectra of compound **13**

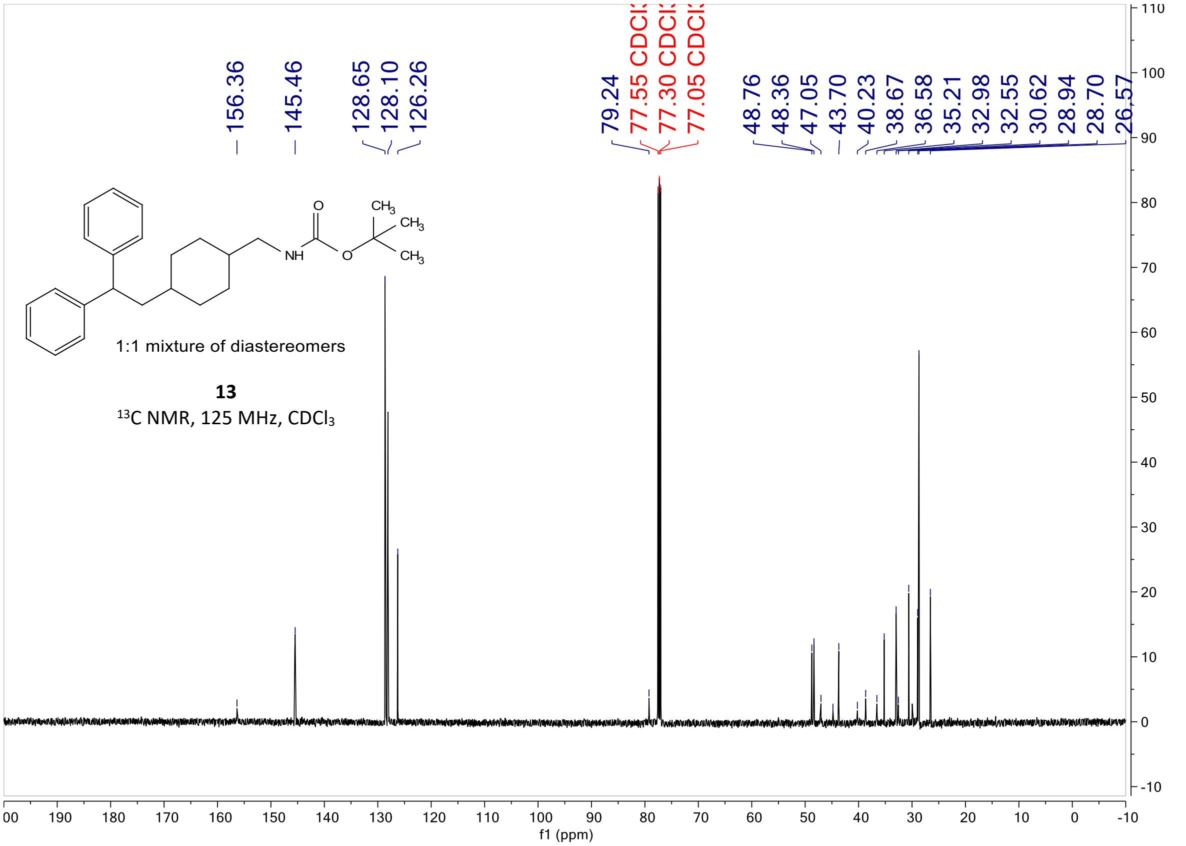

Supplementary Figure 79. ^{13}C NMR spectra of compound **13**

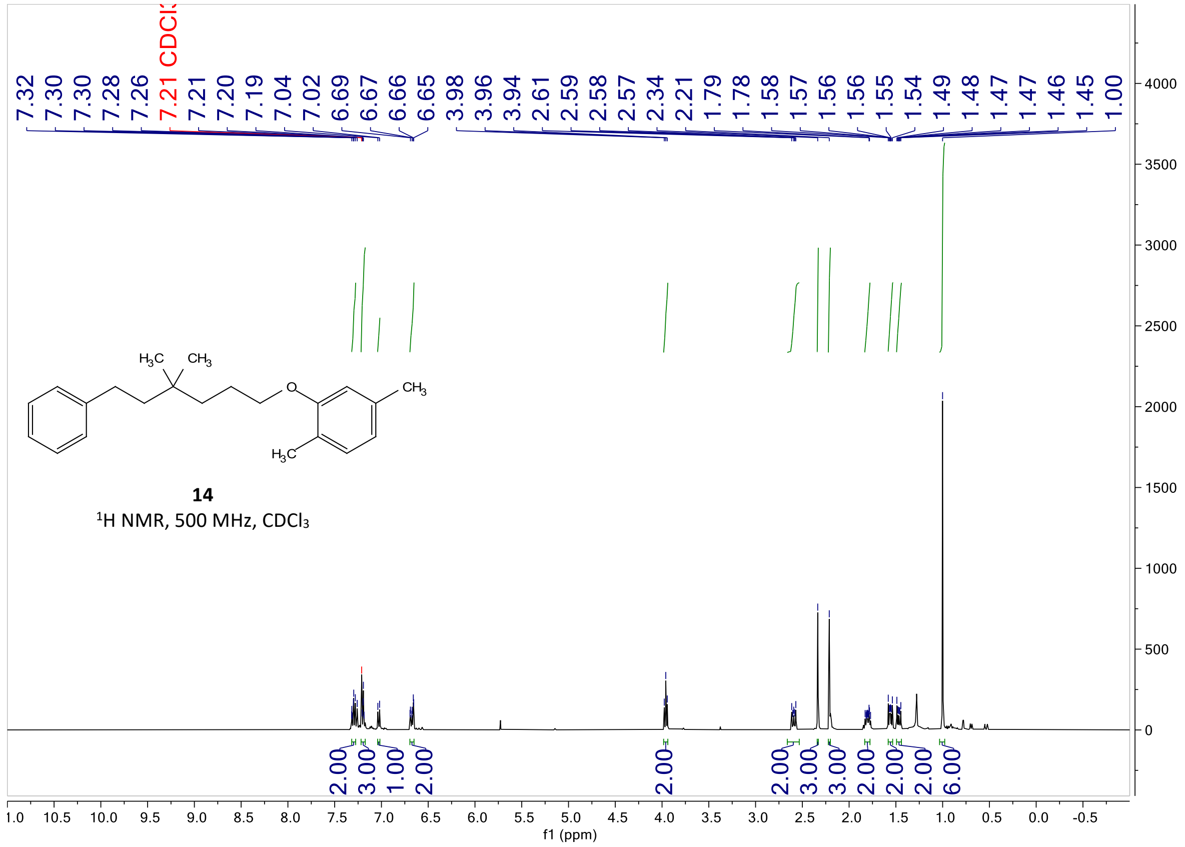

Supplementary Figure 80. ^{1}H NMR spectra of compound **14**

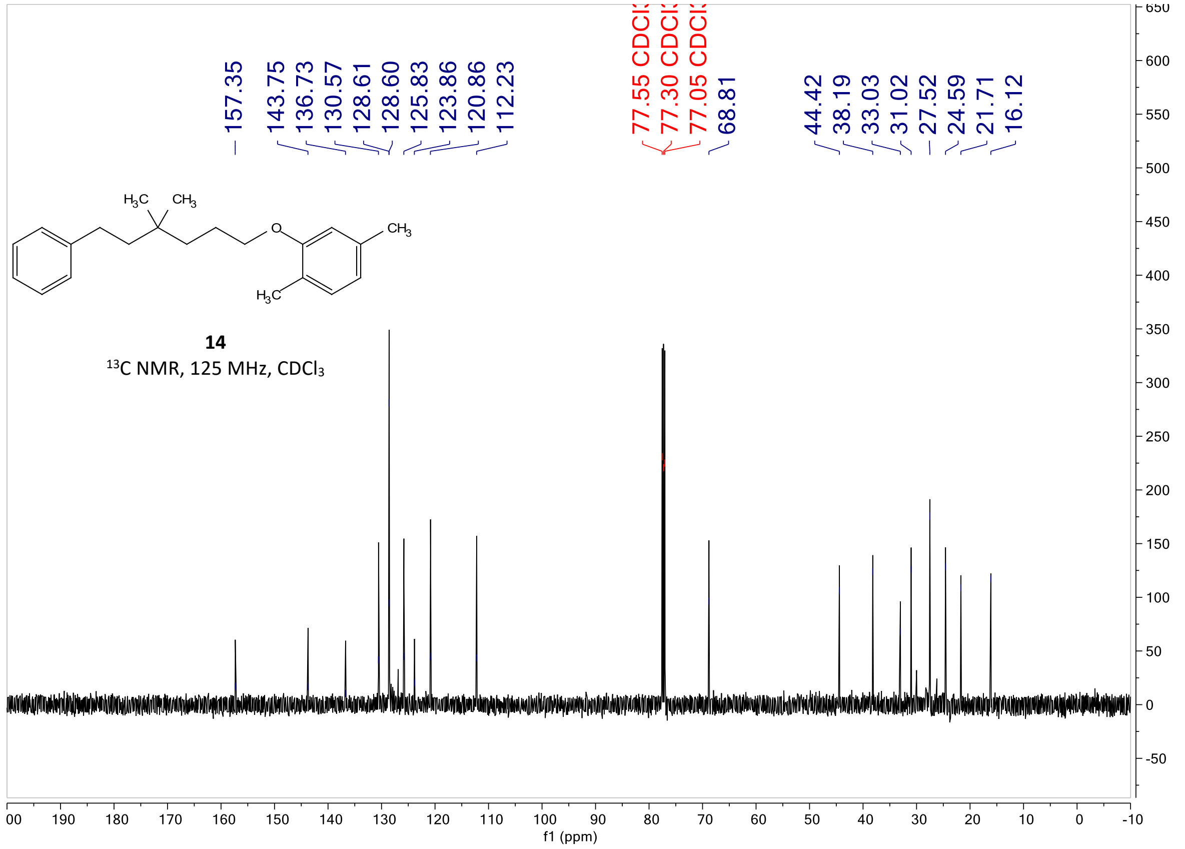

Supplementary Figure 81. ^{13}C NMR spectra of compound **14**

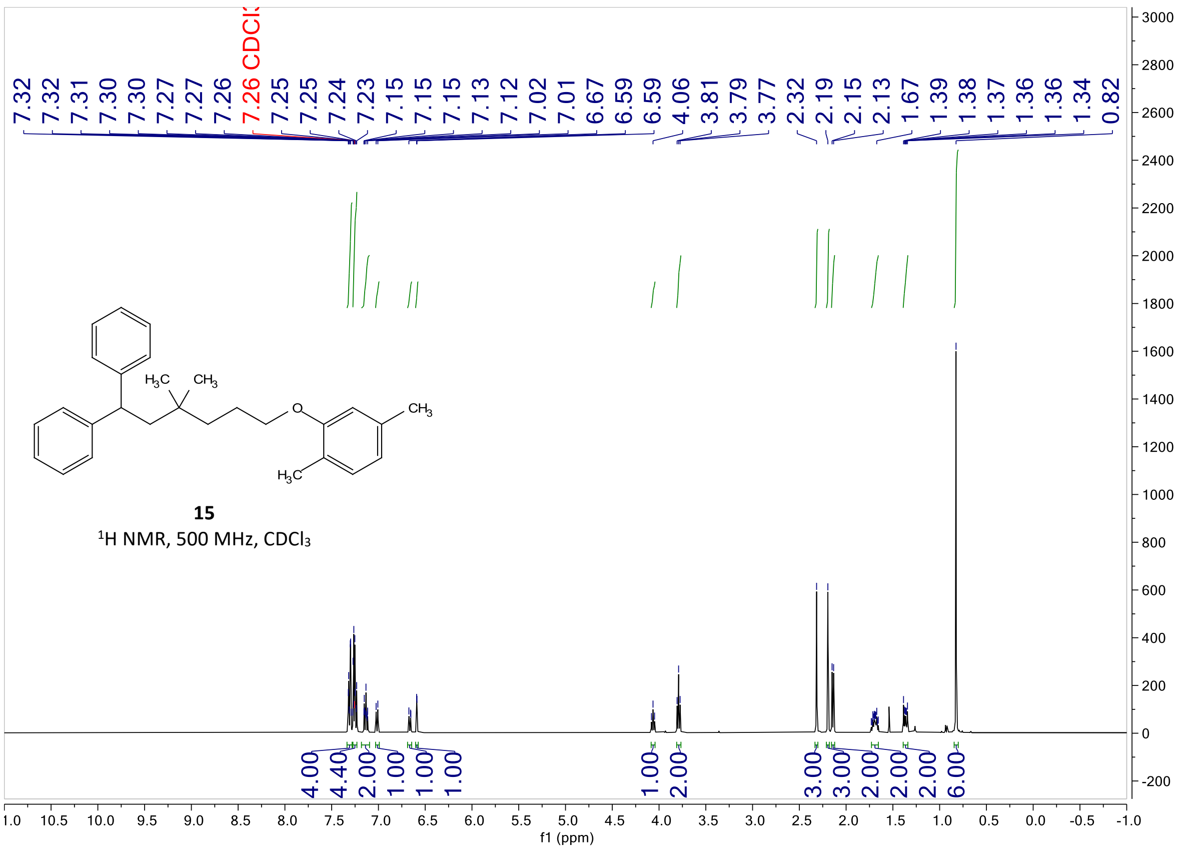

Supplementary Figure 82. ^{1}H NMR spectra of compound **15**

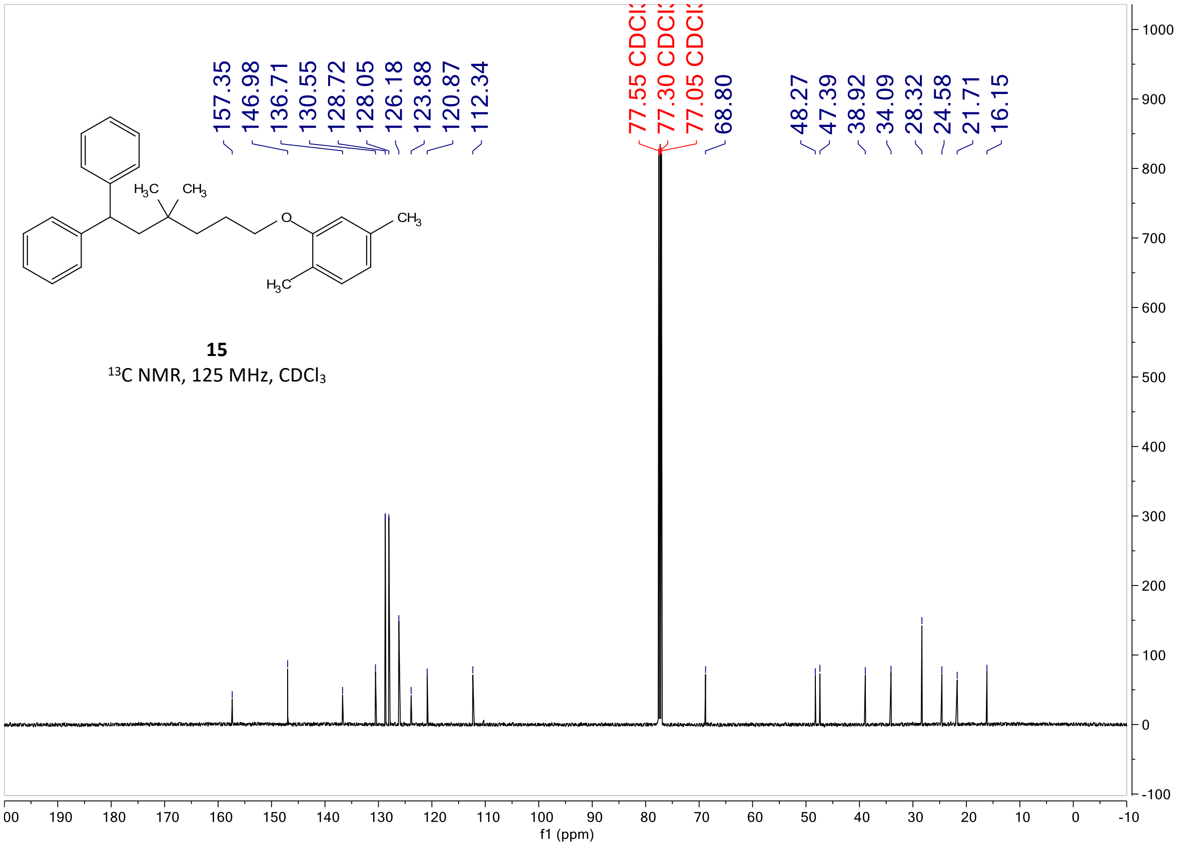

Supplementary Figure 83. ^{13}C NMR spectra of compound **15**

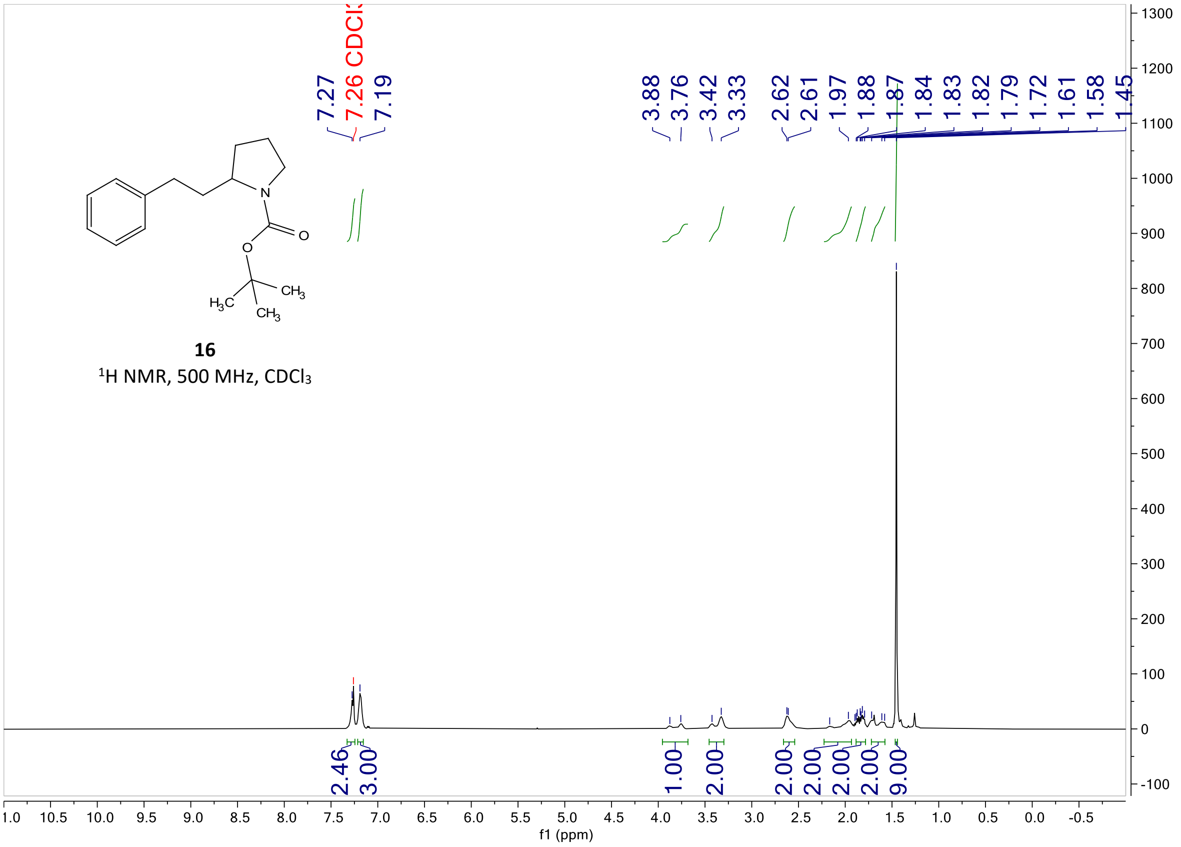

Supplementary Figure 84. ^{1}H NMR spectra of compound **16**

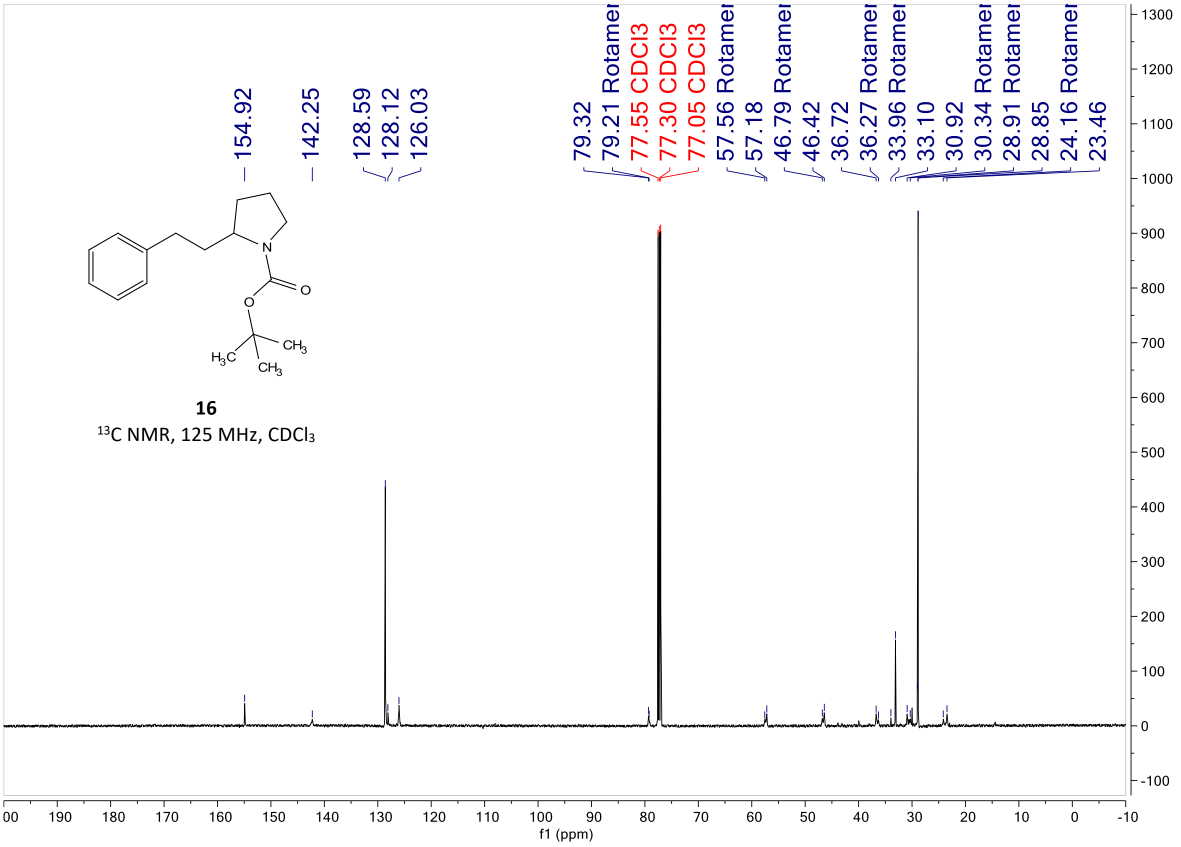

Supplementary Figure 85. ^{13}C NMR spectra of compound **16**

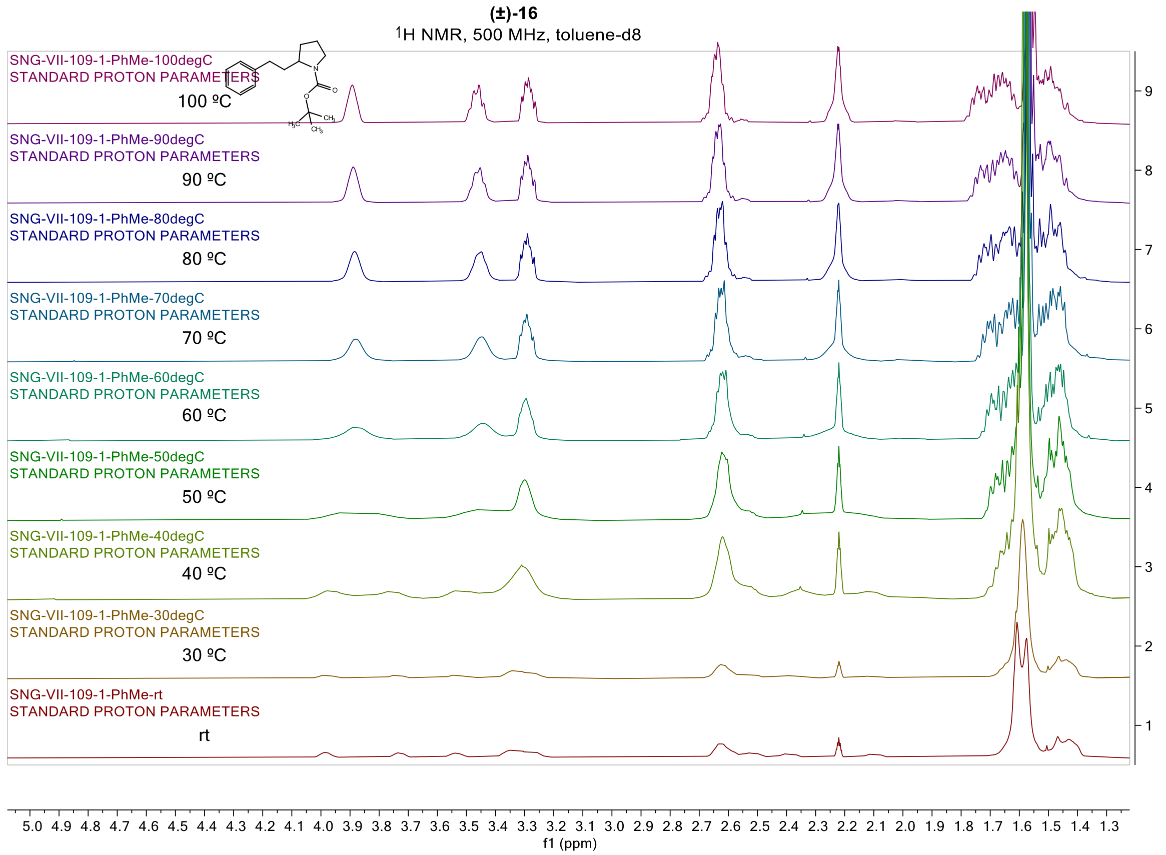

Supplementary Figure 86. ^{1}H NMR spectra of compound **16** in toluene-d8 at various temperatures

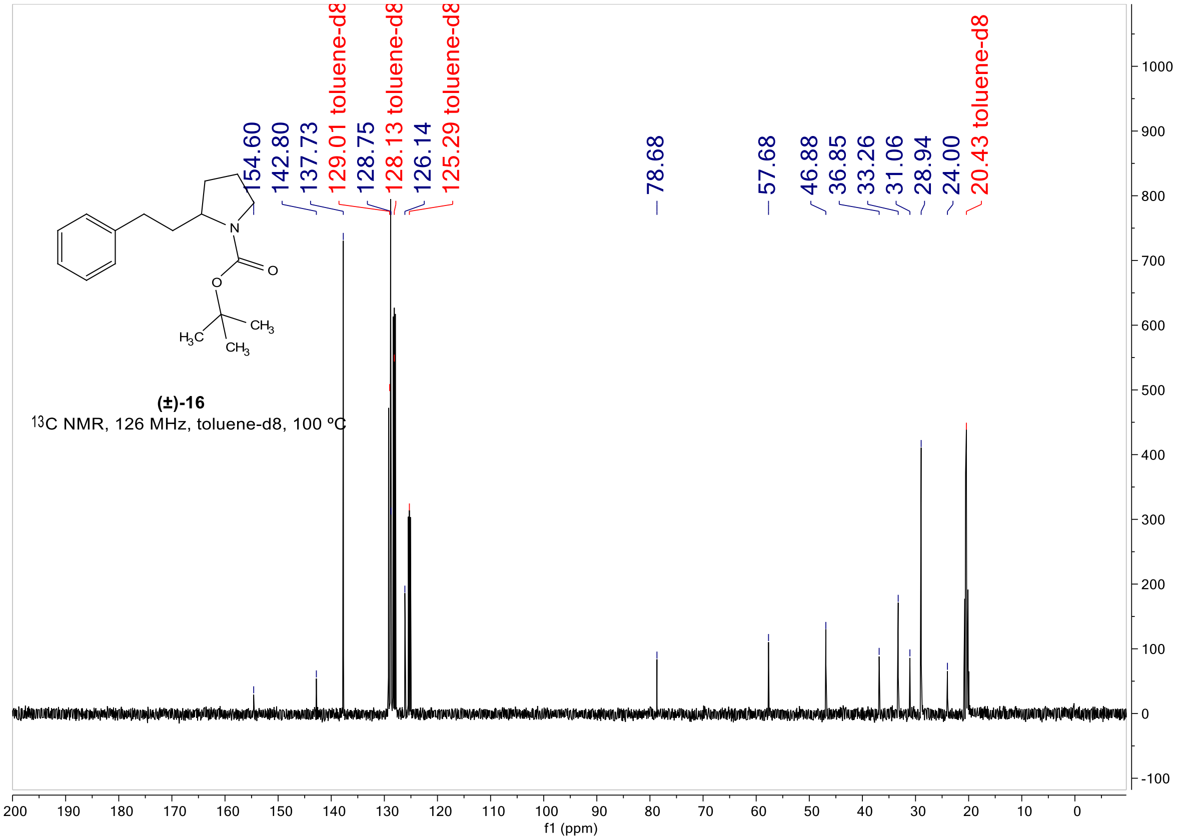

Supplementary Figure 87. ^{13}C NMR spectra of compound **16** in toluene-d8 at 100 °C

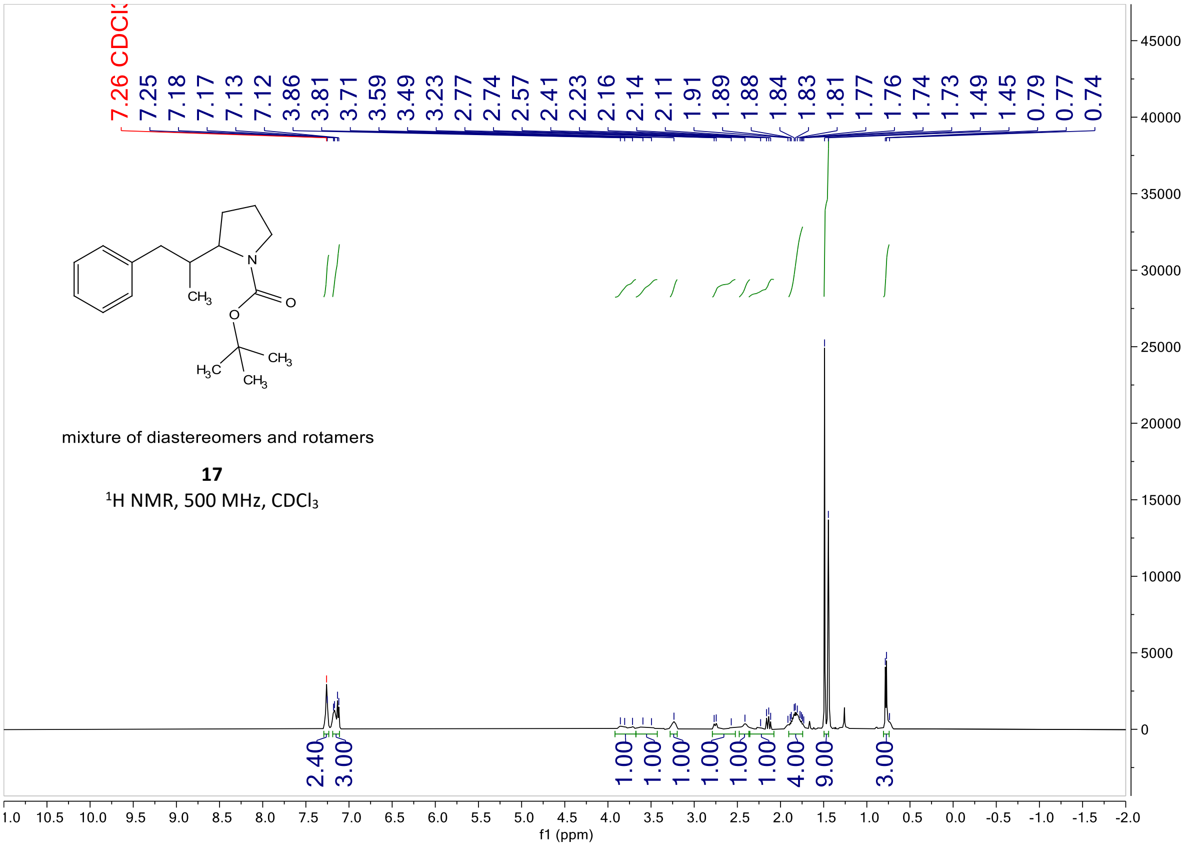

Supplementary Figure 88. ^{1}H NMR spectra of compound **17**

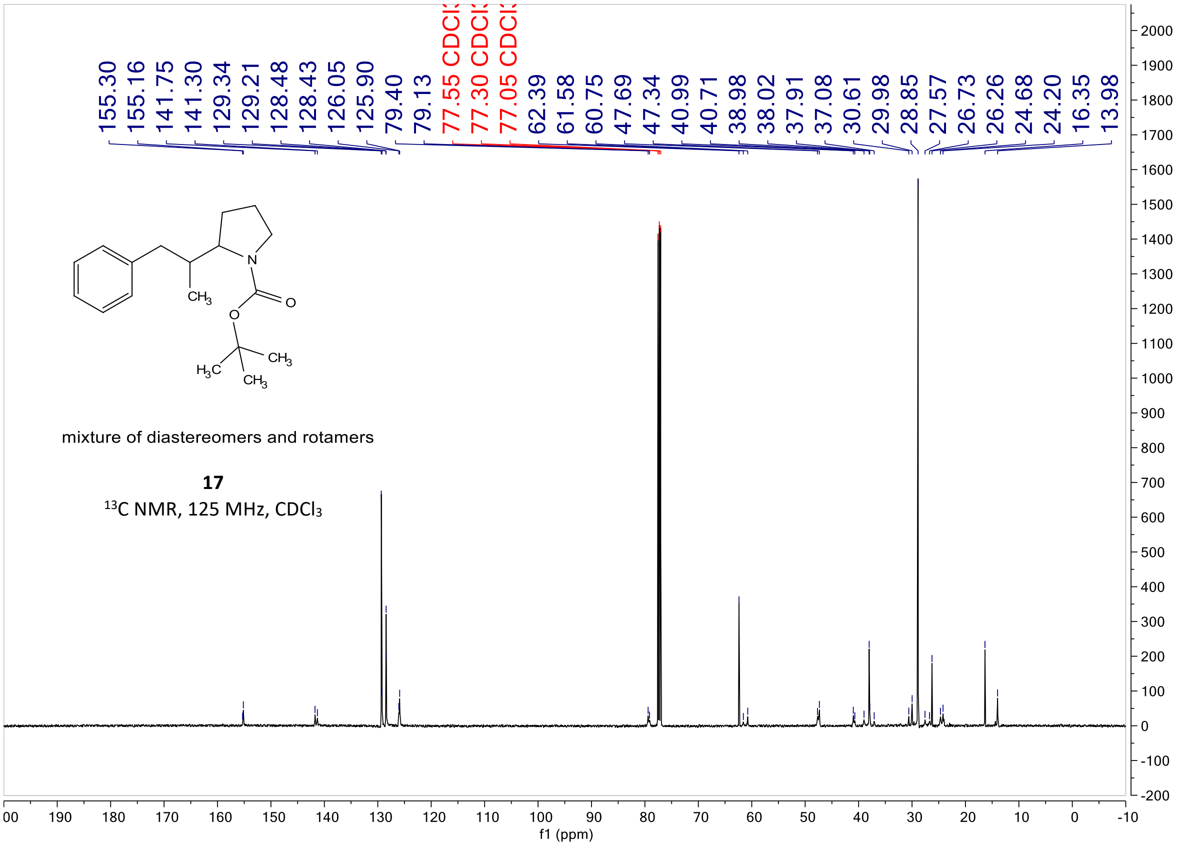

Supplementary Figure 89. ^{13}C NMR spectra of compound **17**

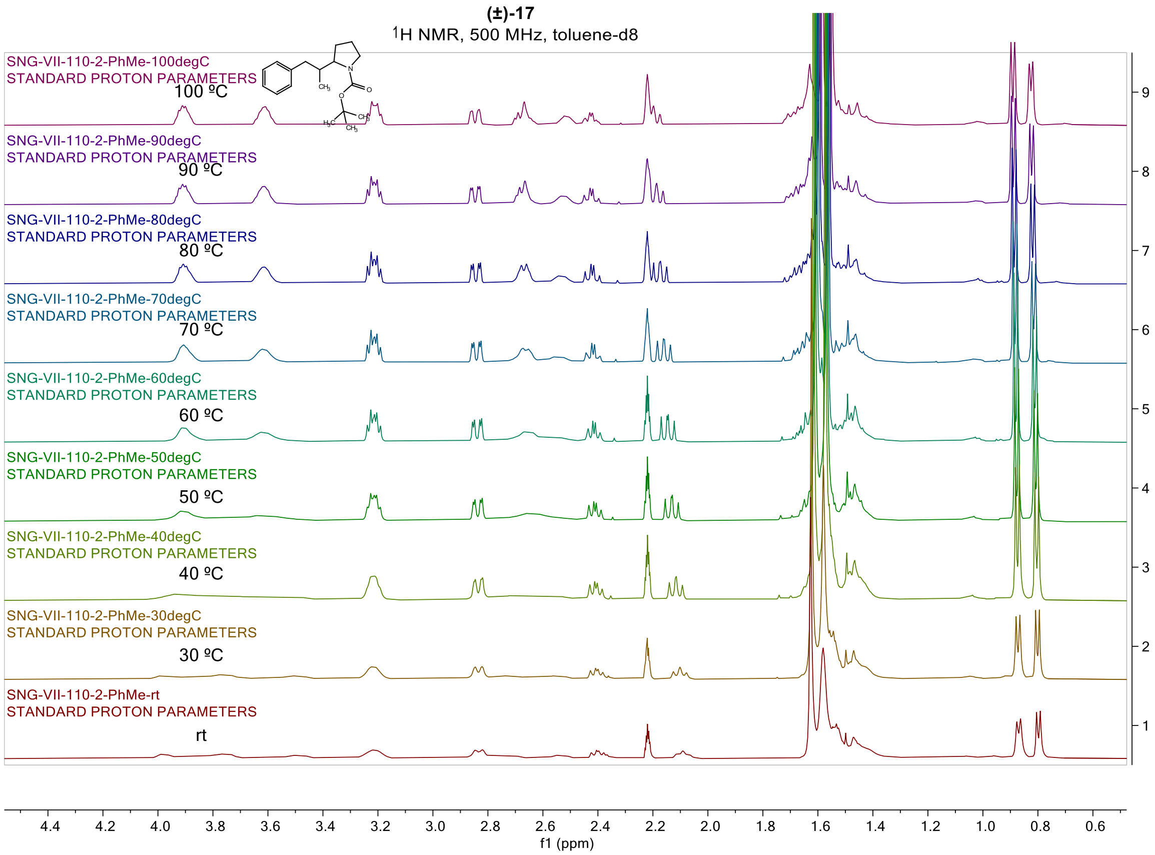

Supplementary Figure 90. ^{1}H NMR spectra of compound **17** in toluene-d8 at various temperatures

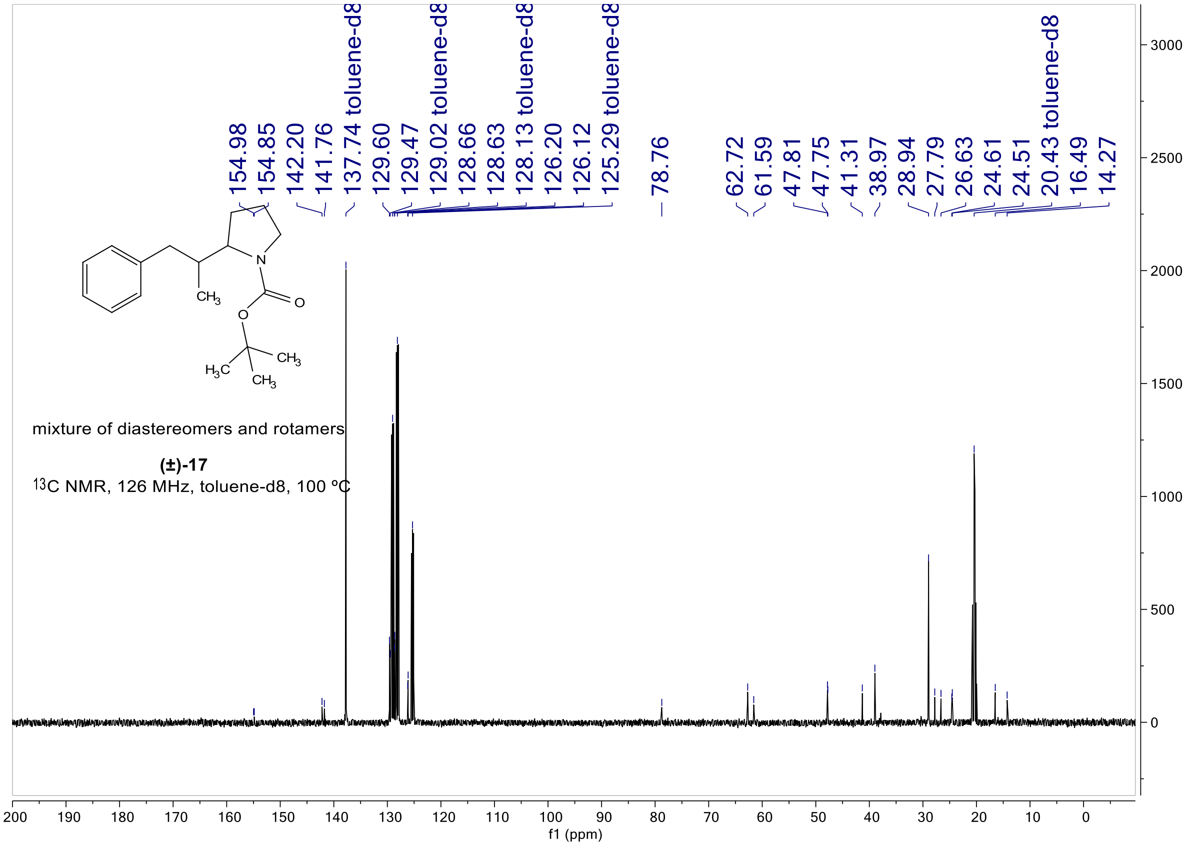

Supplementary Figure 91. ^{13}C NMR spectra of compound **17** in toluene-d8 at 100 °C

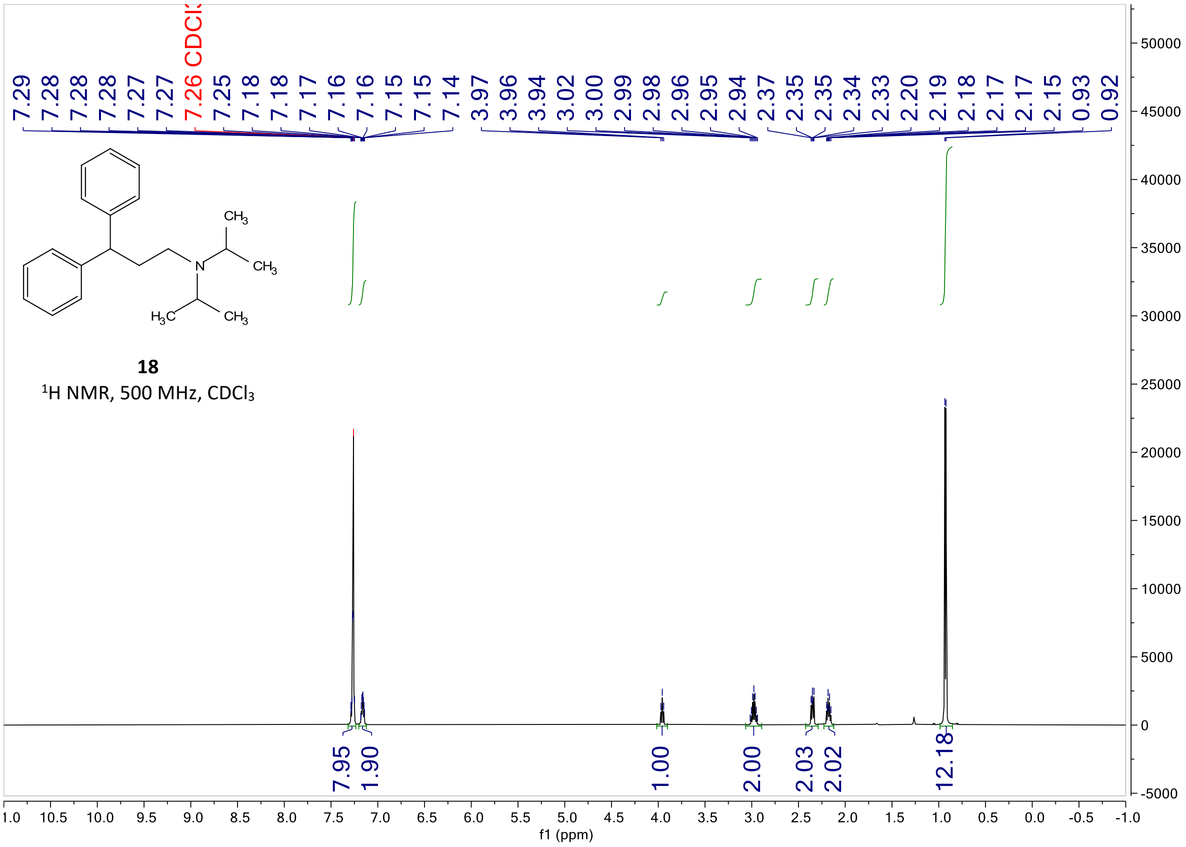

Supplementary Figure 92. ^{1}H NMR spectra of compound **18**

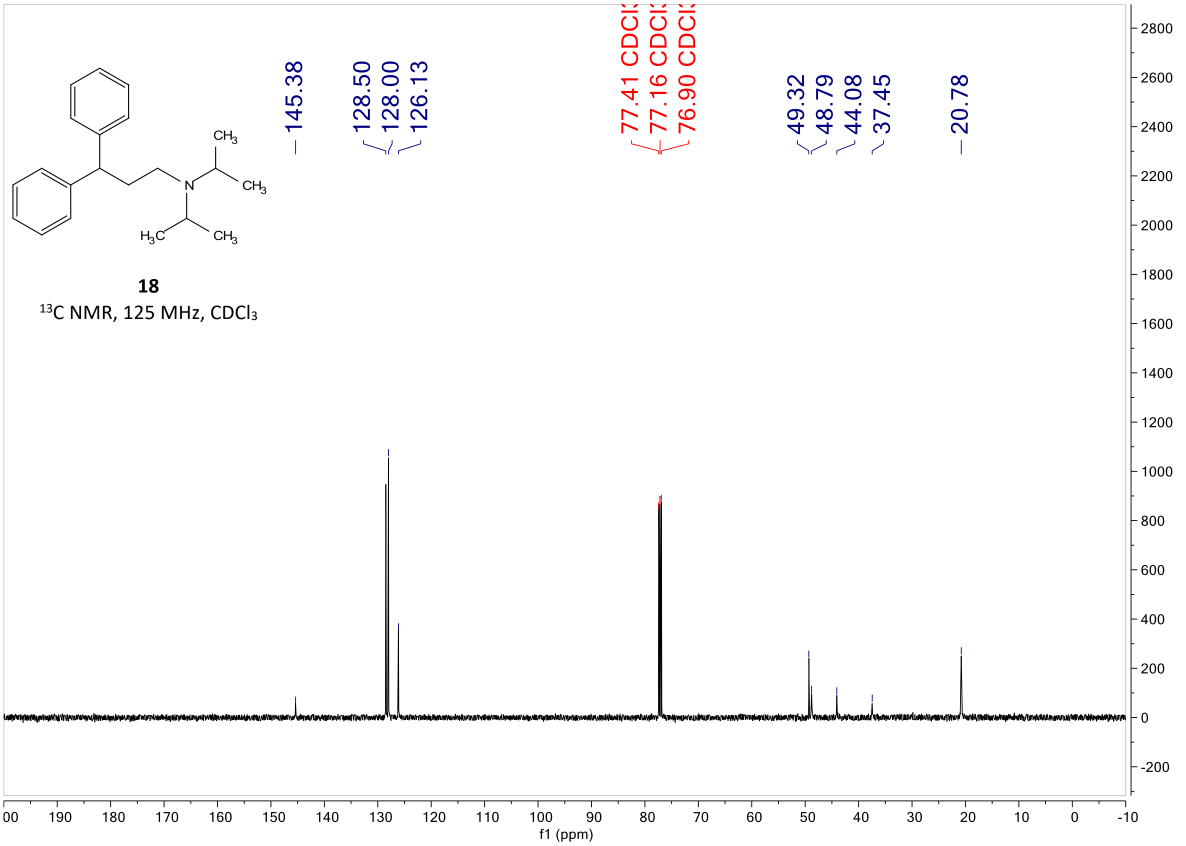

Supplementary Figure 93. ^{13}C NMR spectra of compound **18**

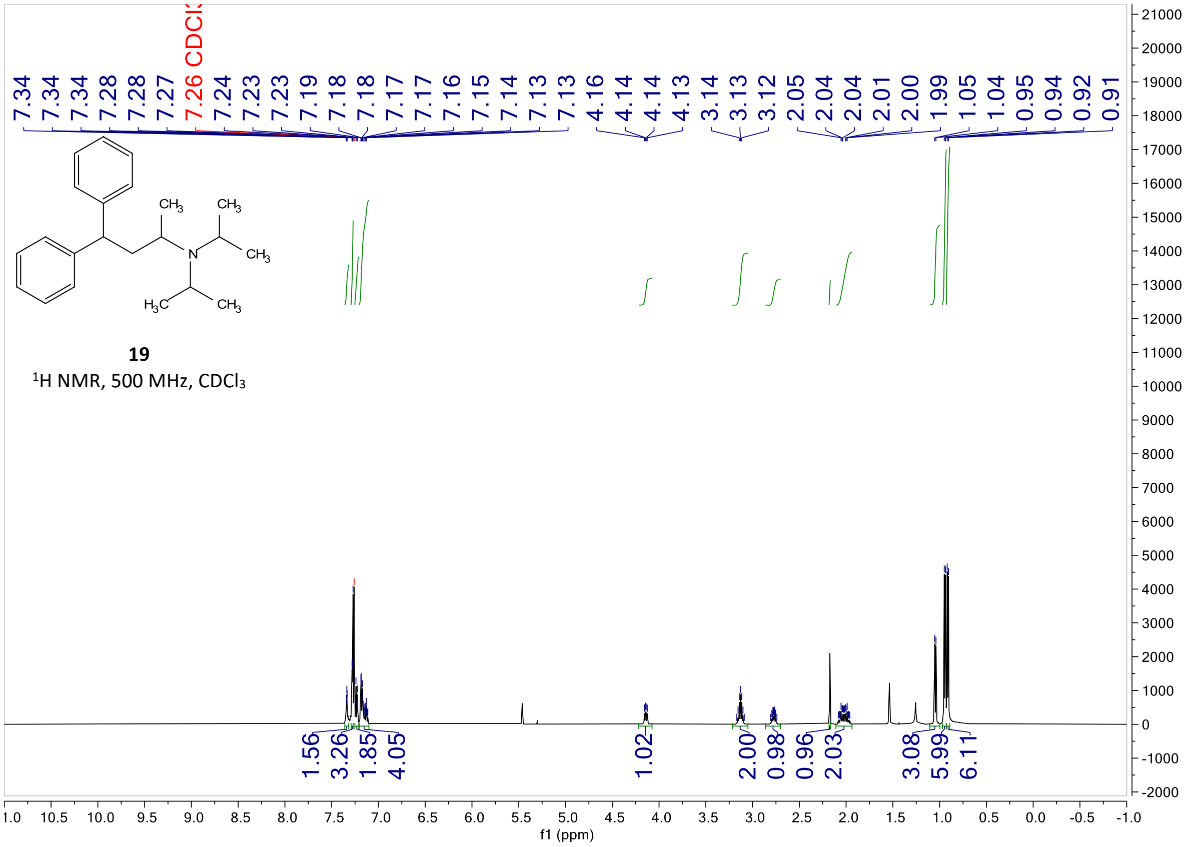

Supplementary Figure 94. ^{1}H NMR spectra of compound **19**

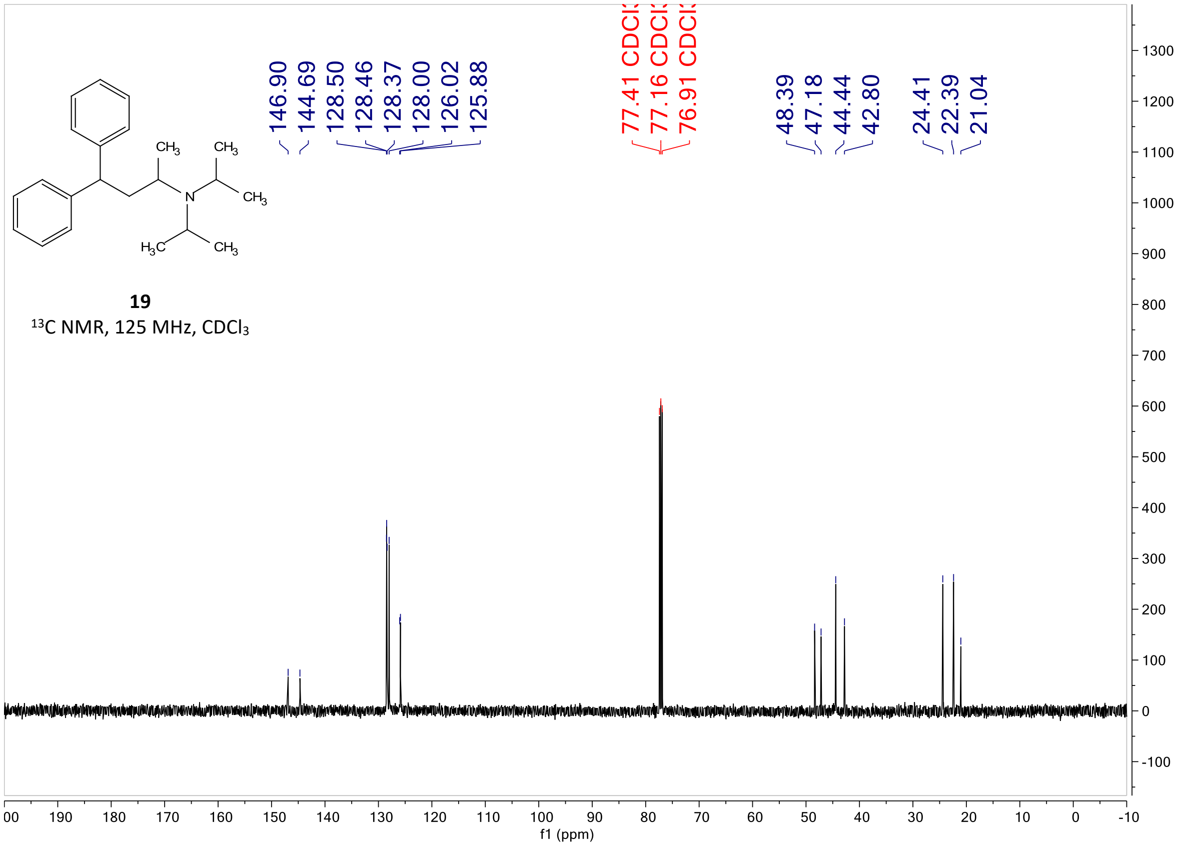

Supplementary Figure 95. ^{13}C NMR spectra of compound **19**

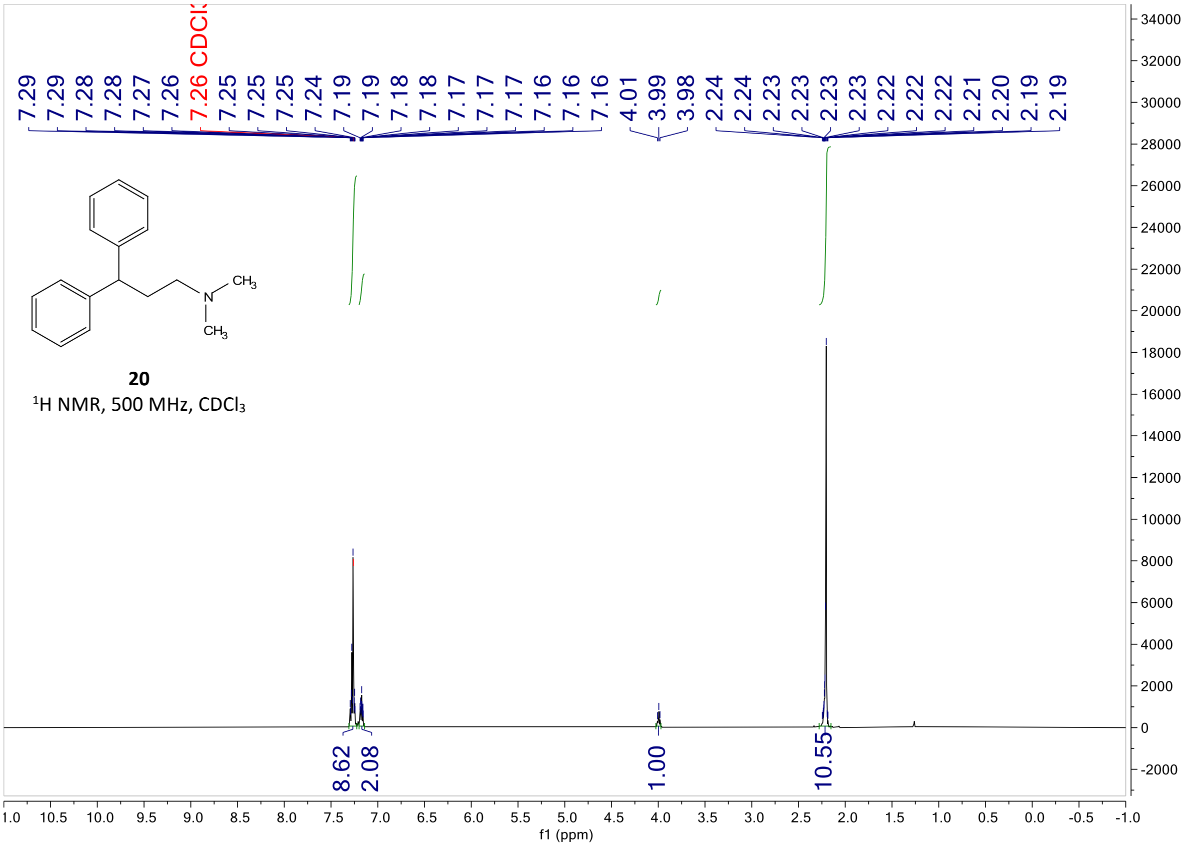

Supplementary Figure 96. ^{1}H NMR spectra of compound **20**

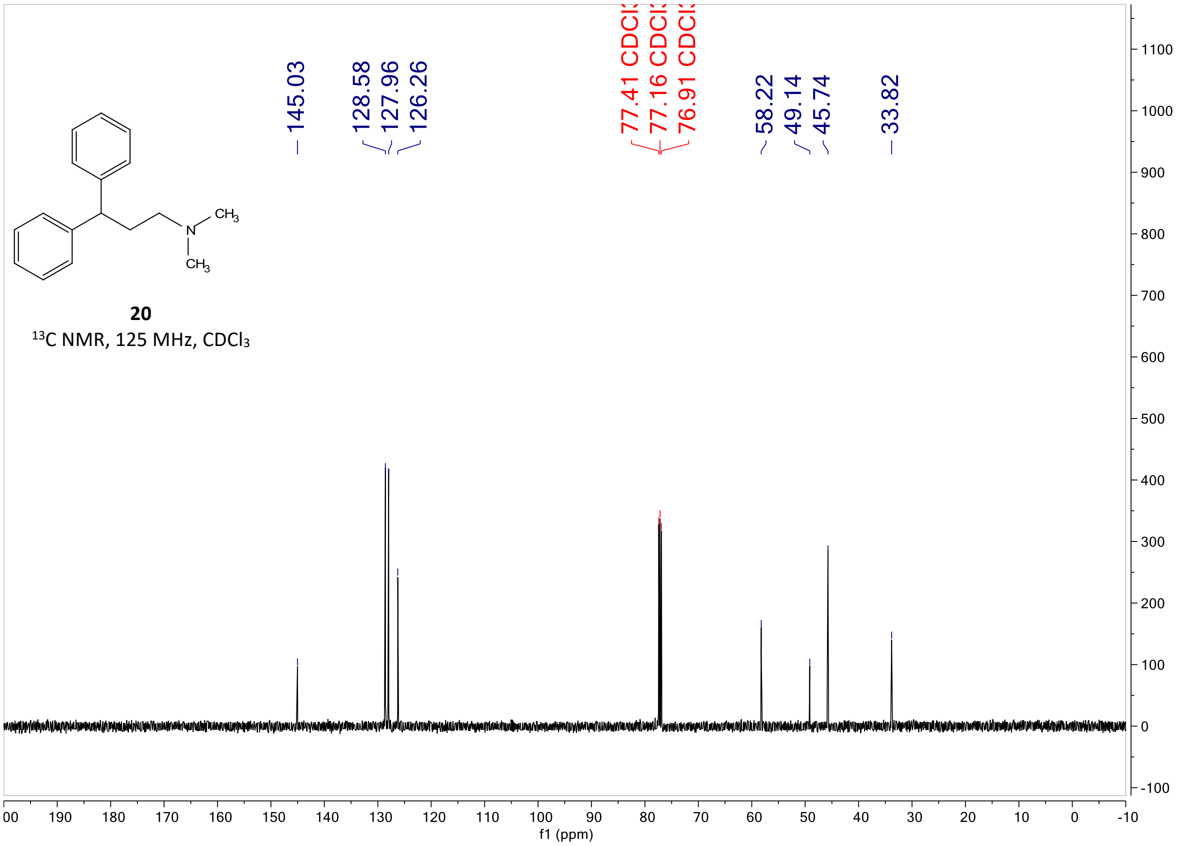

Supplementary Figure 97. ^{13}C NMR spectra of compound **20**

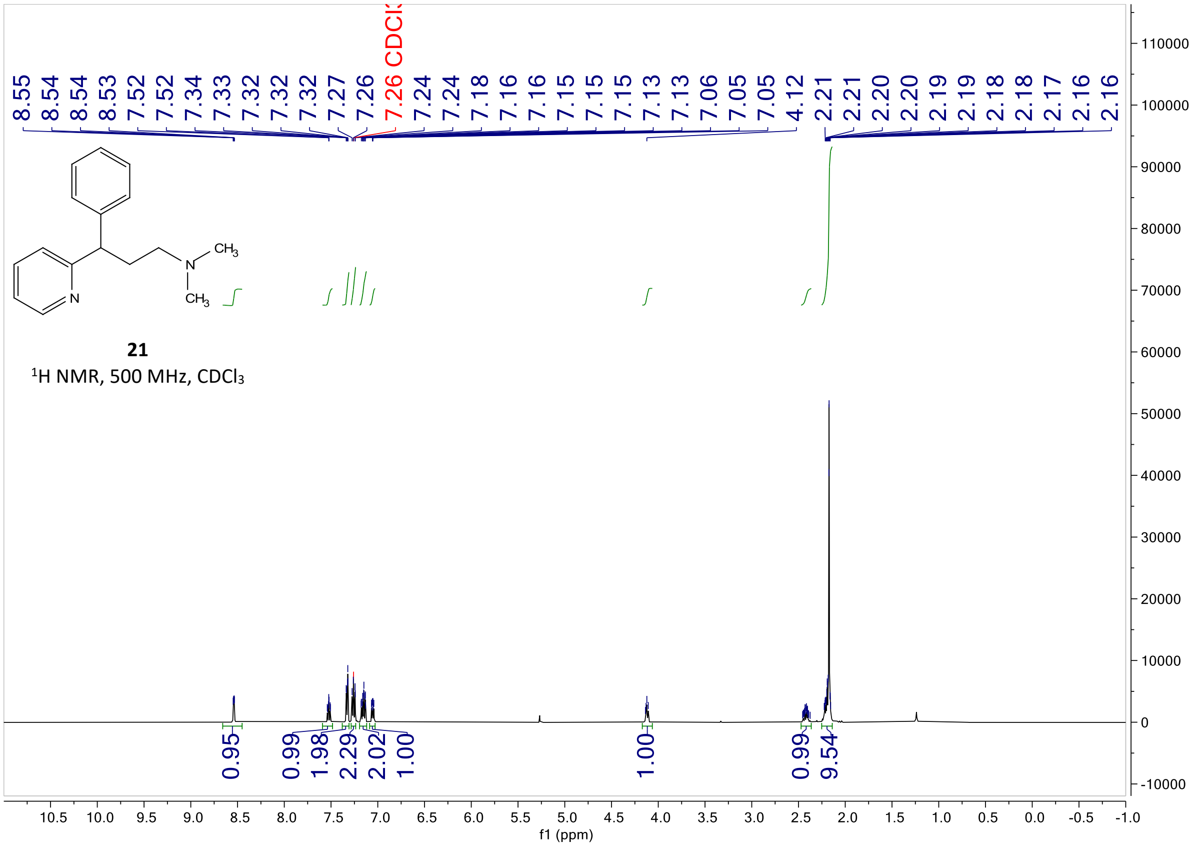

Supplementary Figure 98. ^{1}H NMR spectra of compound **21**

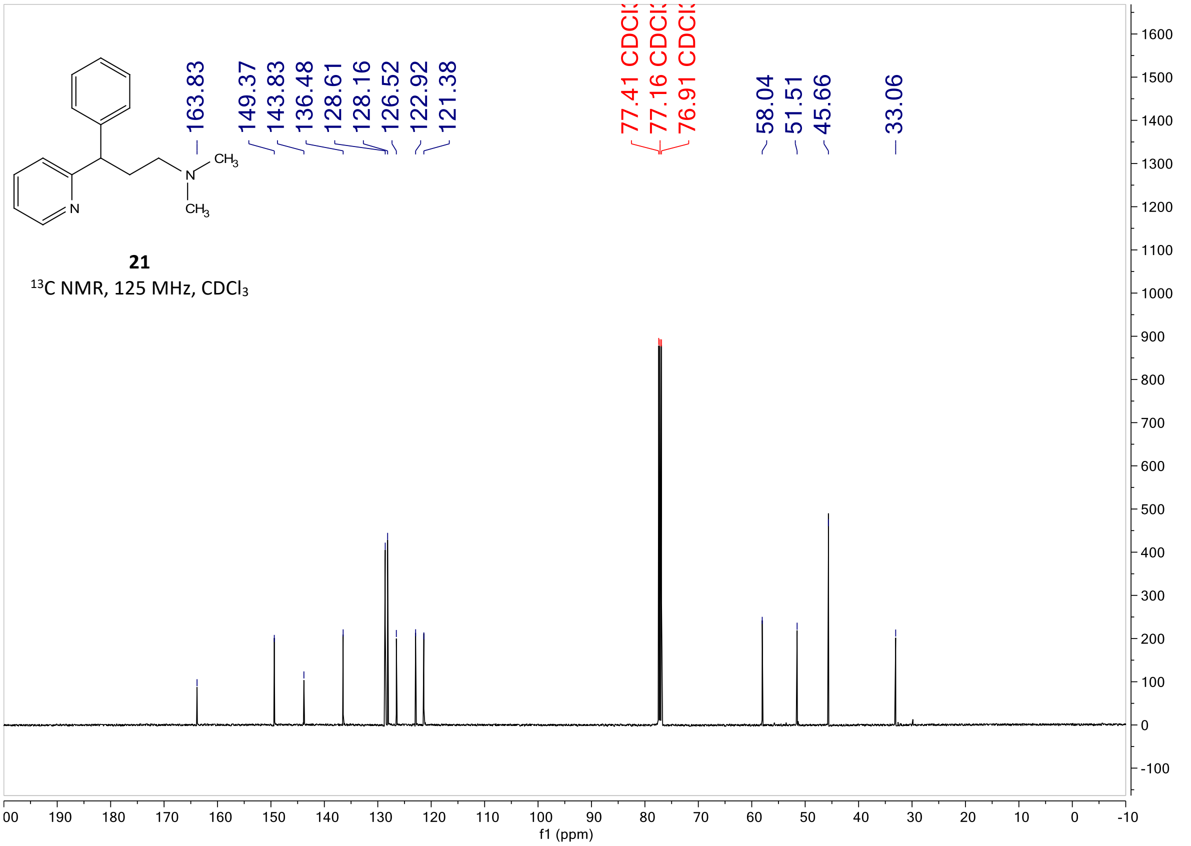

Supplementary Figure 99. ^{13}C NMR spectra of compound **21**

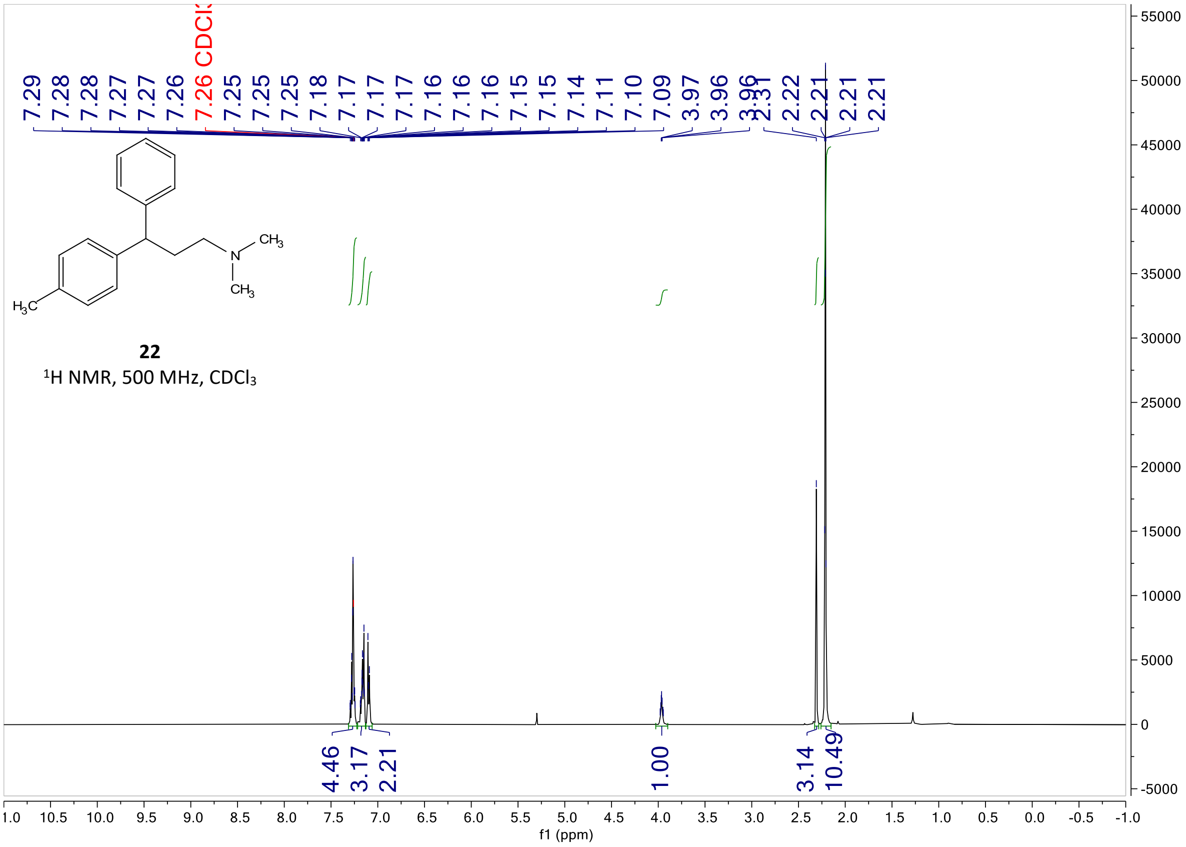

Supplementary Figure 100. ^{1}H NMR spectra of compound **22**

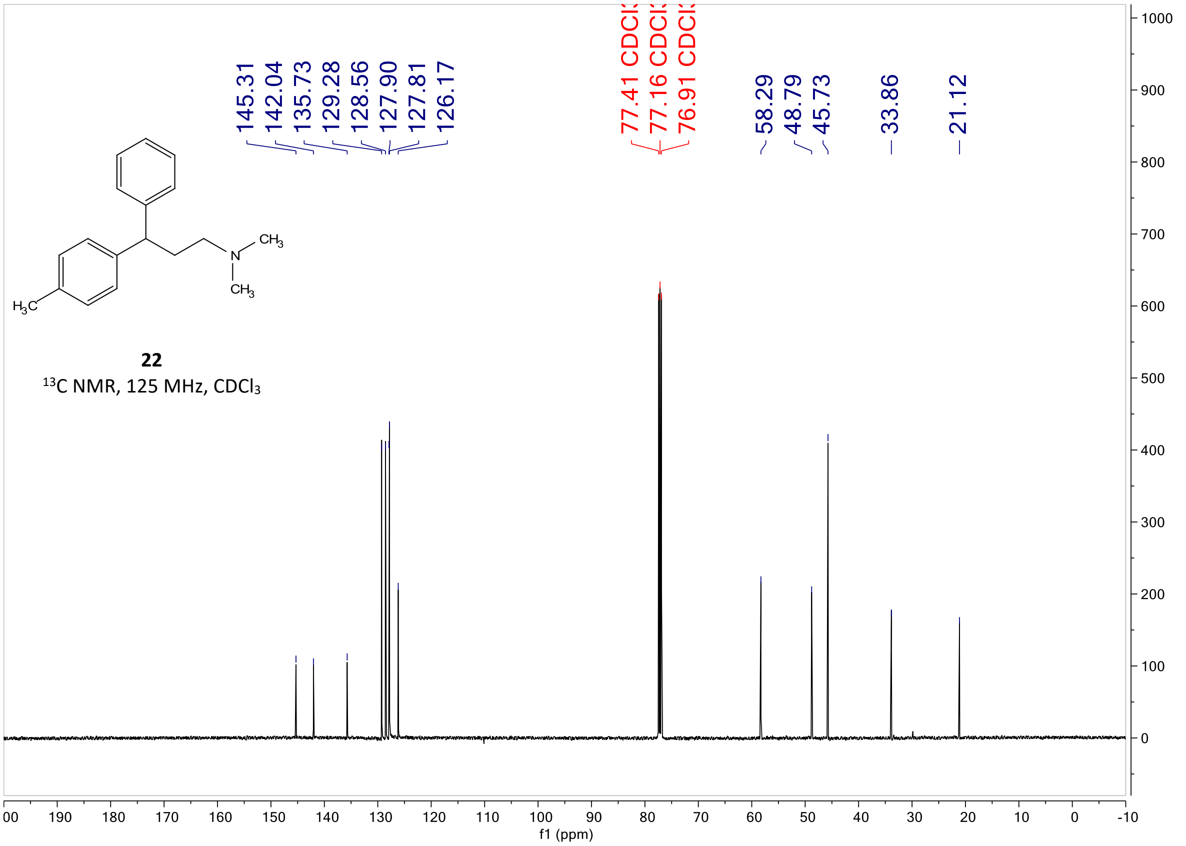

Supplementary Figure 101. ^{13}C NMR spectra of compound **22**

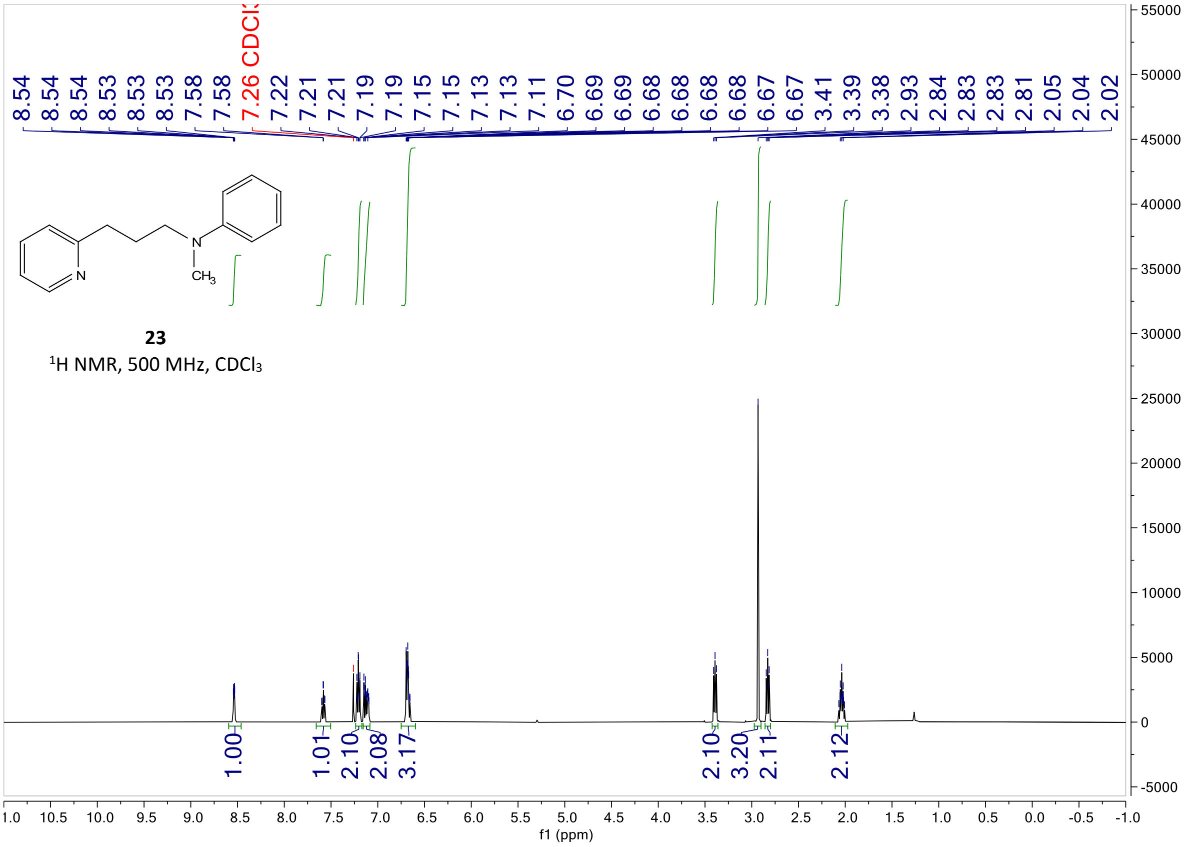

Supplementary Figure 102. ^{1}H NMR spectra of compound **23**

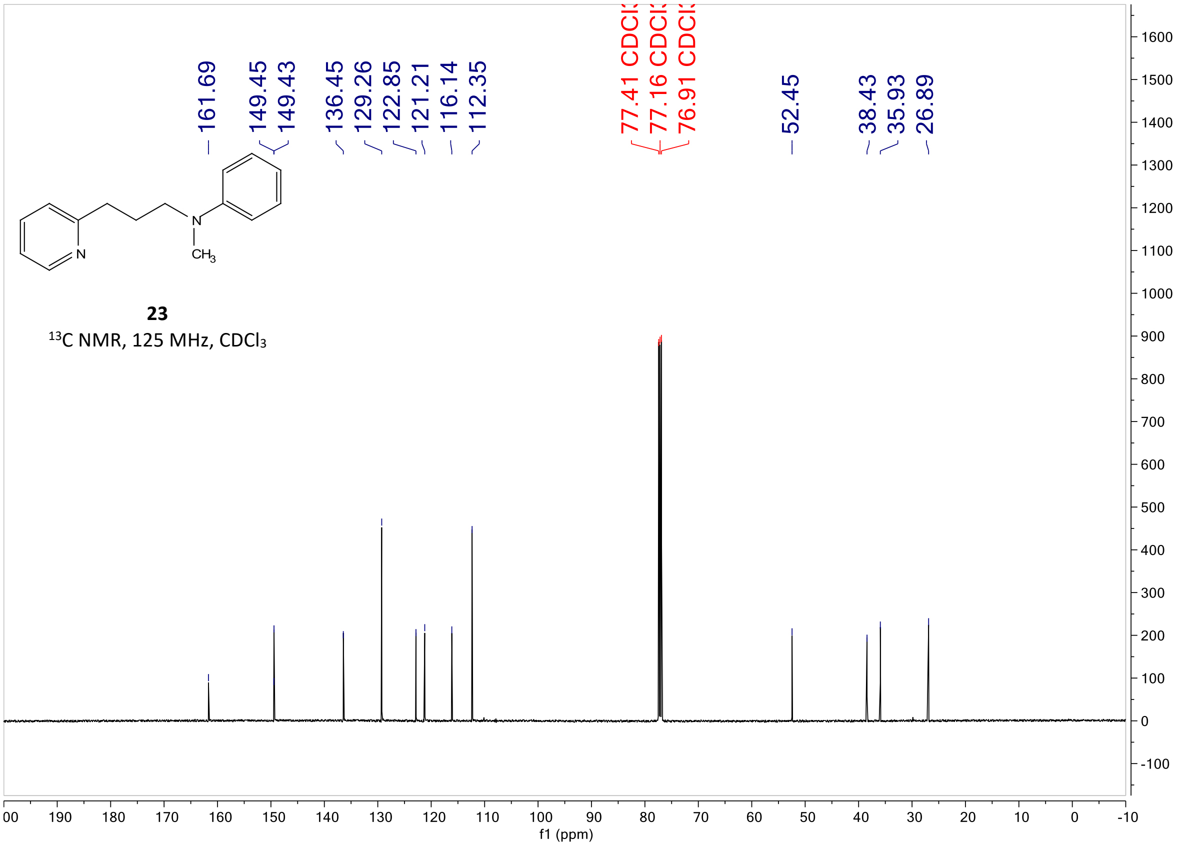

Supplementary Figure 103. ^{13}C NMR spectra of compound **23**

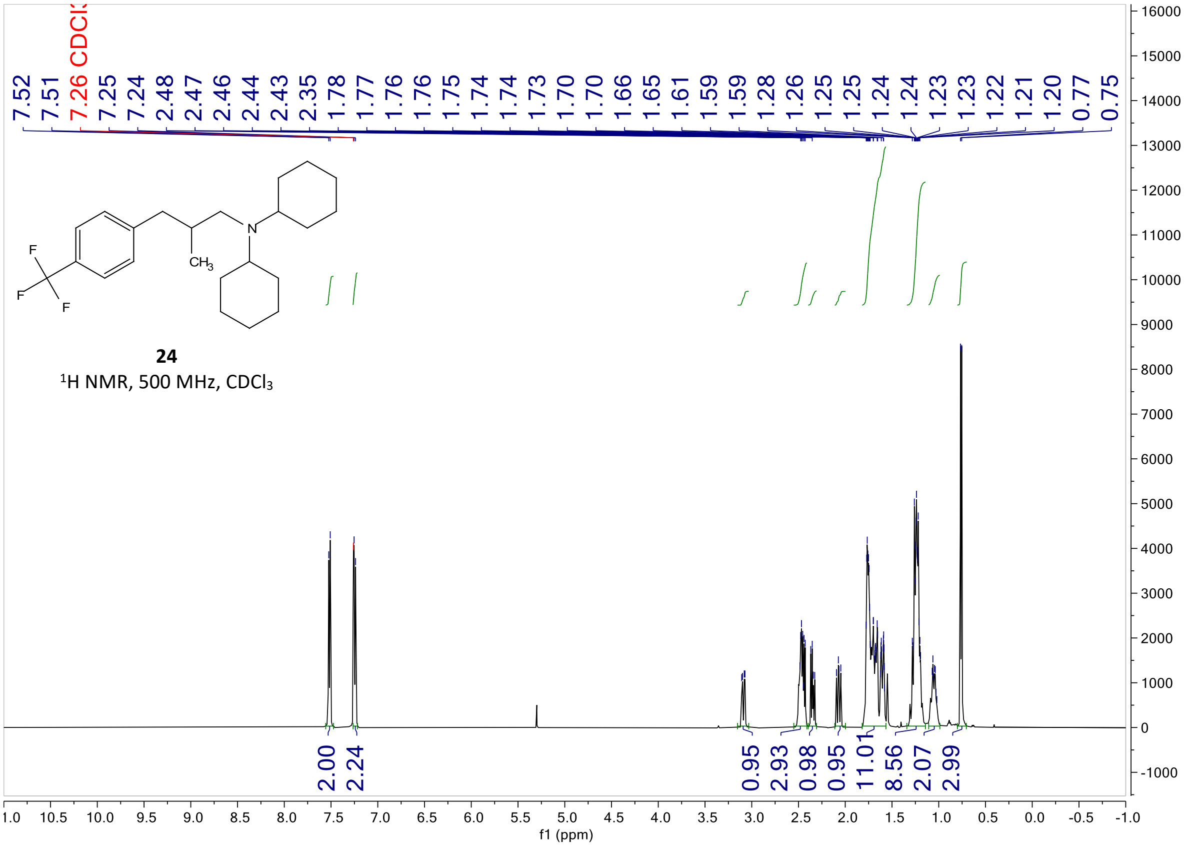

Supplementary Figure 104. ^{1}H NMR spectra of compound **24**

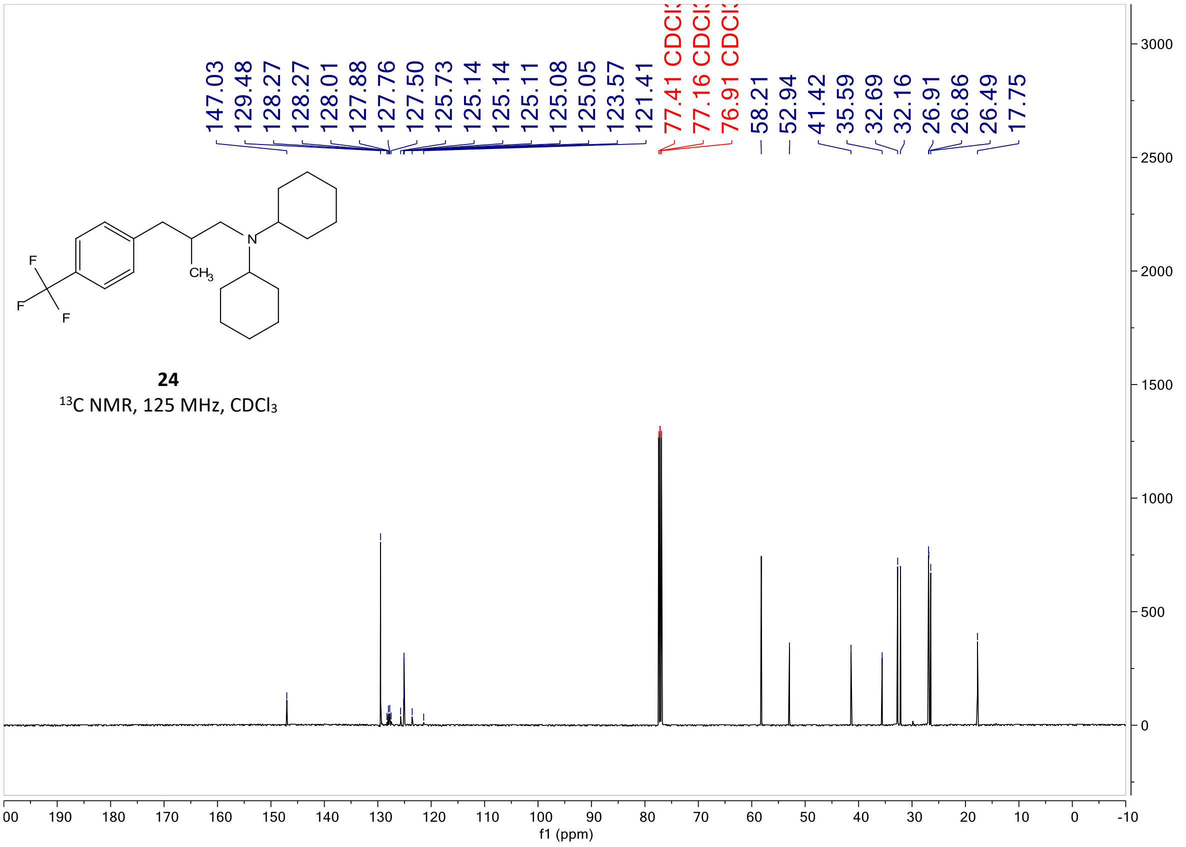

Supplementary Figure 105. ^{13}C NMR spectra of compound **24**

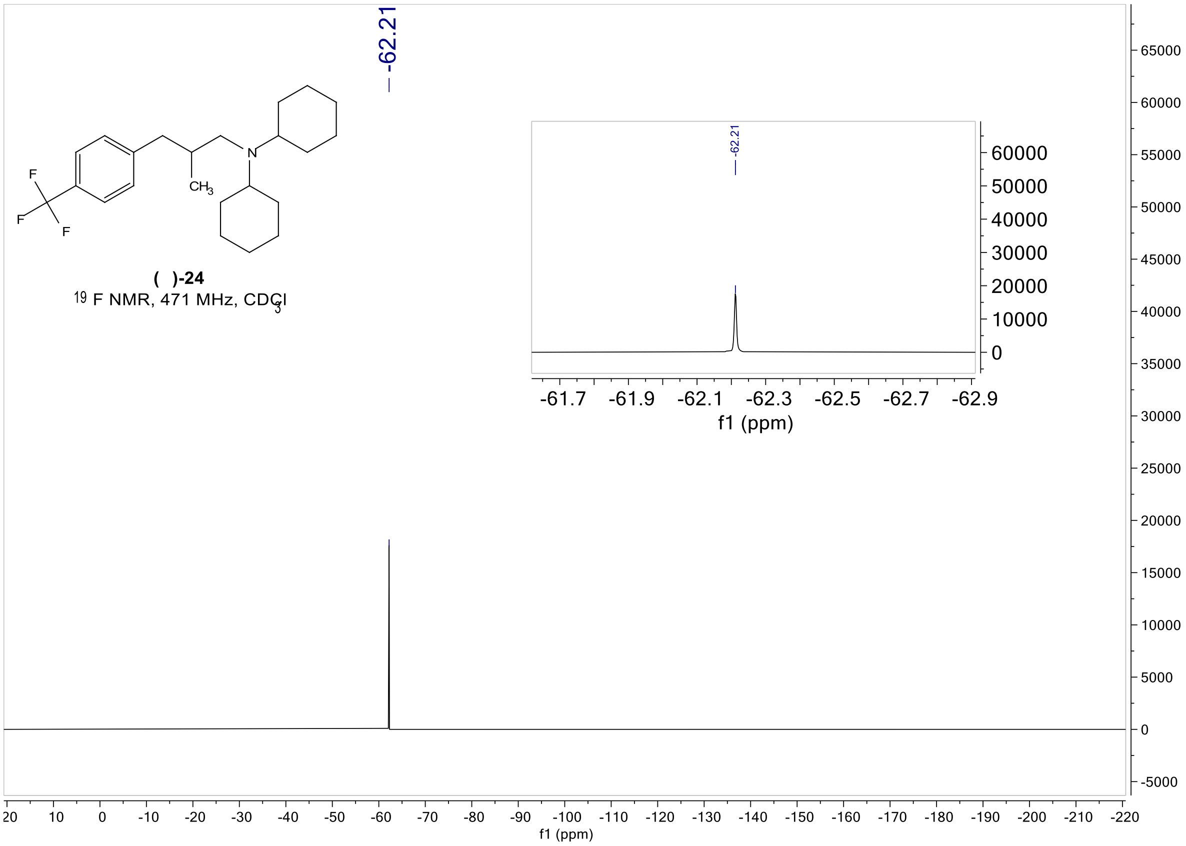

Supplementary Figure 106. ^{19}F NMR spectra of compound **24**

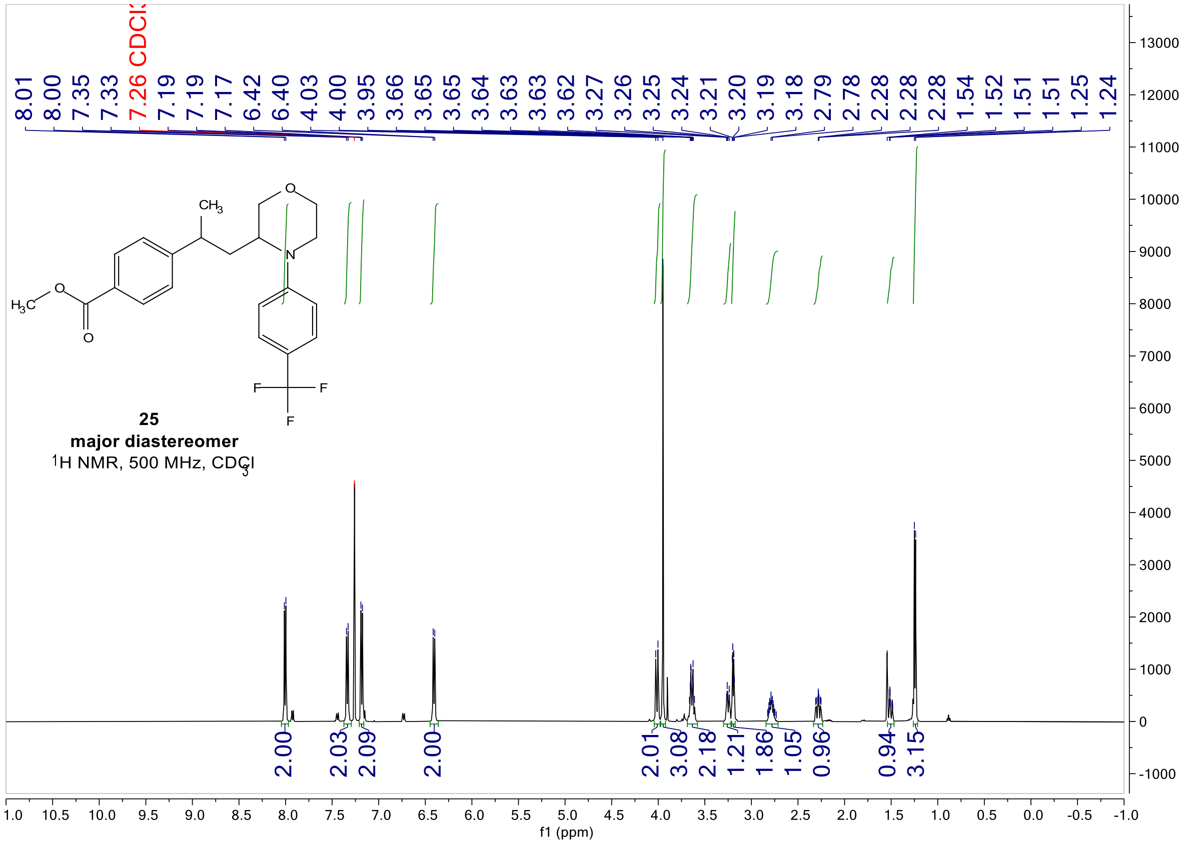

Supplementary Figure 107. ^{1}H NMR spectra of the major diastereomer of compound **25**

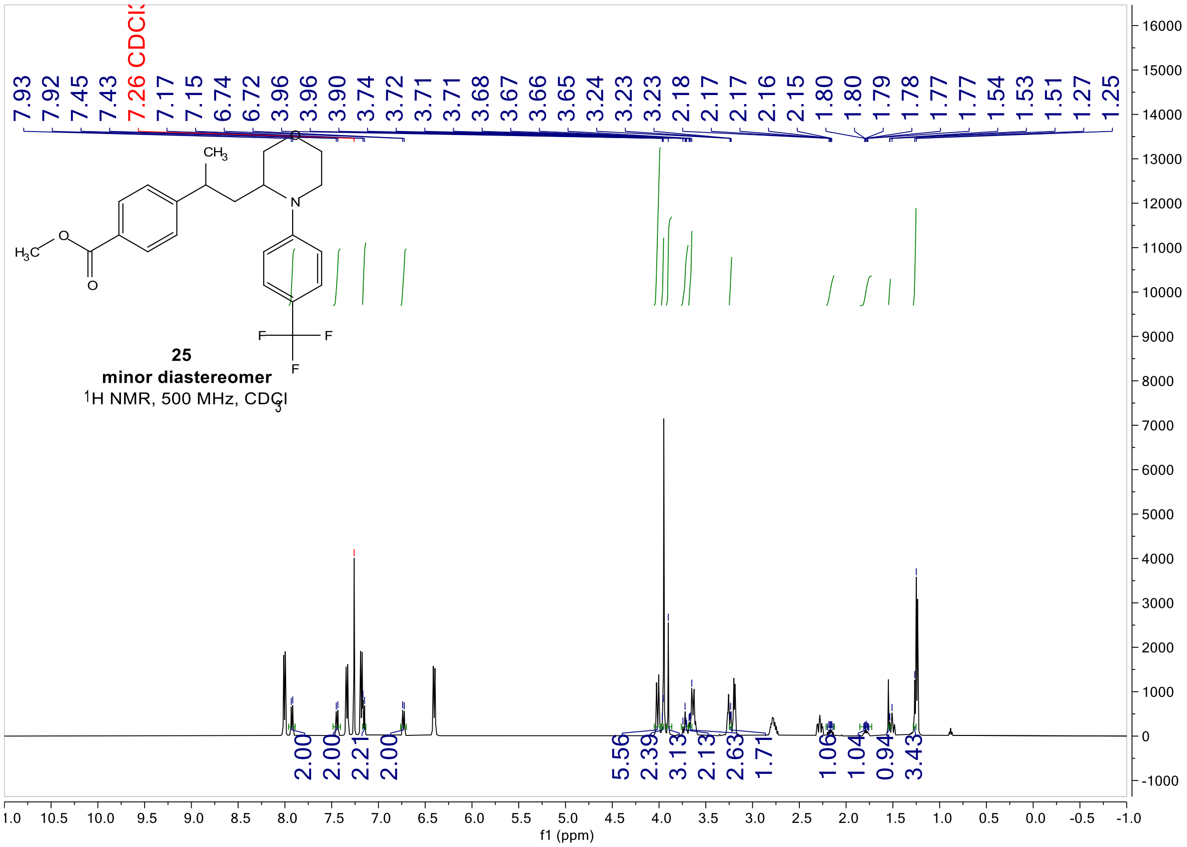

Supplementary Figure 108. ^{1}H NMR spectra of the minor diastereomer of compound **25**

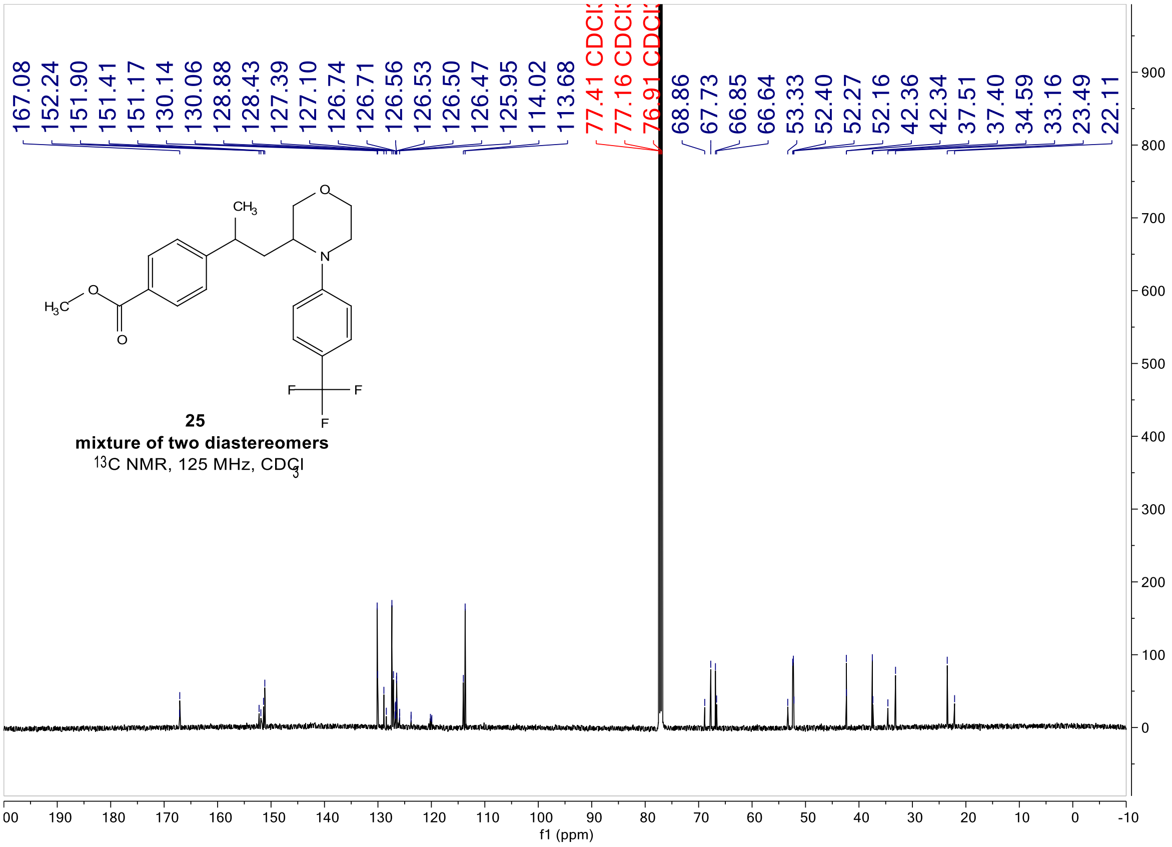

Supplementary Figure 109. ^{13}C NMR spectra of compound **25**

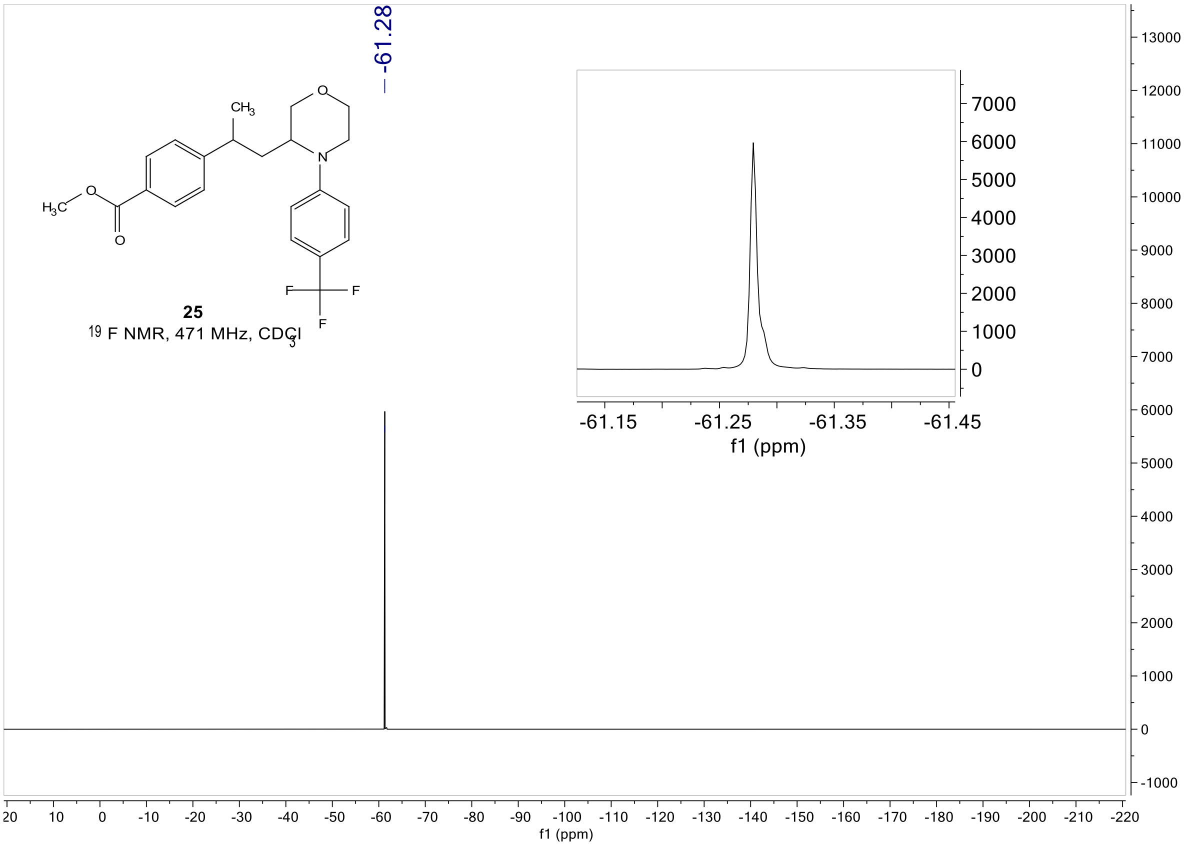

Supplementary Figure 110. ^{19}F NMR spectra of compound **25**

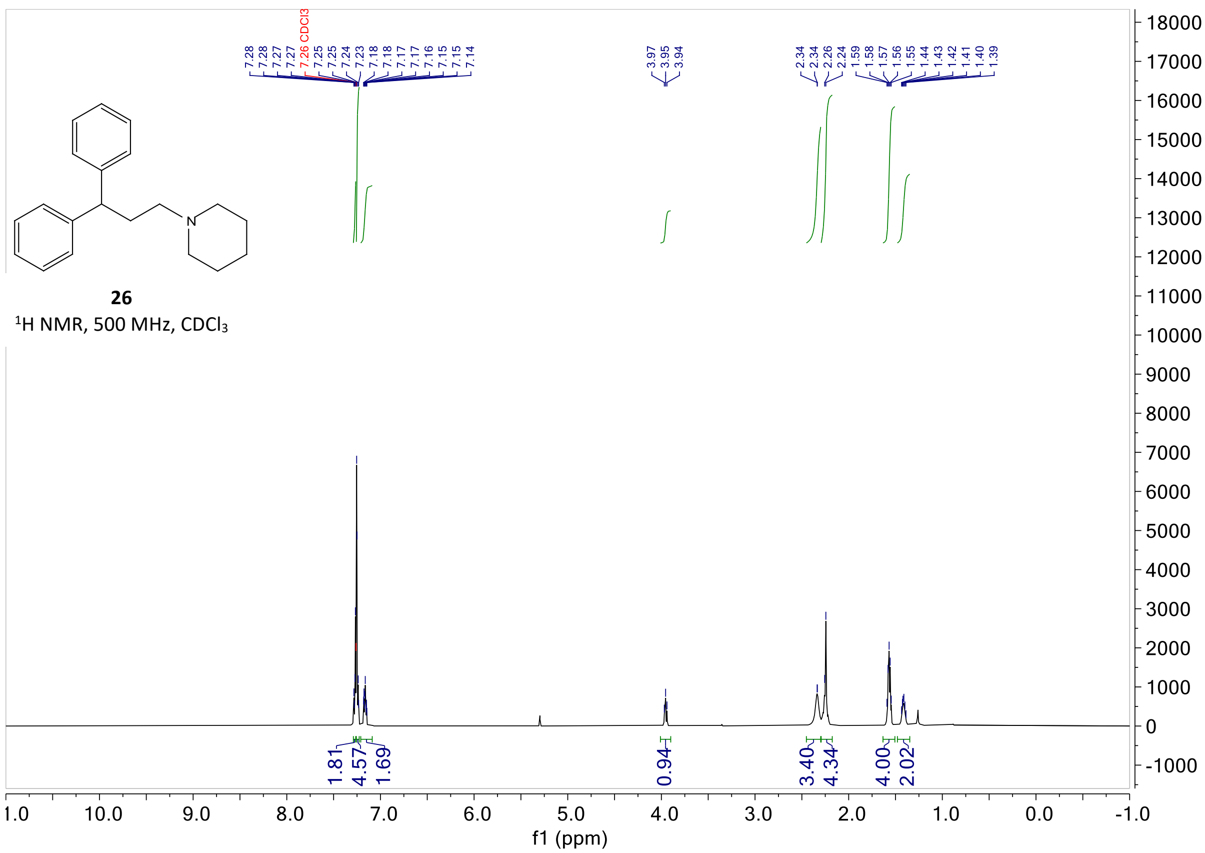

Supplementary Figure 111. ^{1}H NMR spectra of compound **26**

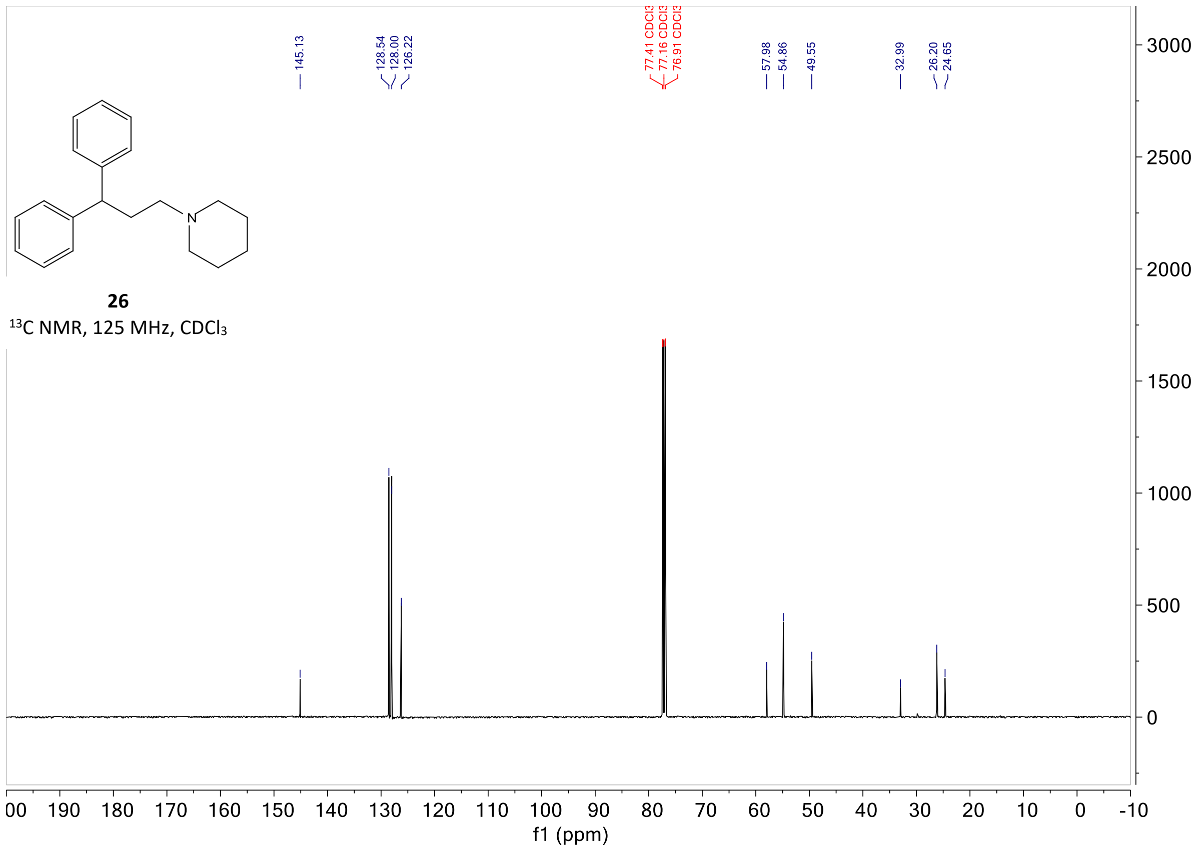

Supplementary Figure 112. ^{13}C NMR spectra of compound **26**

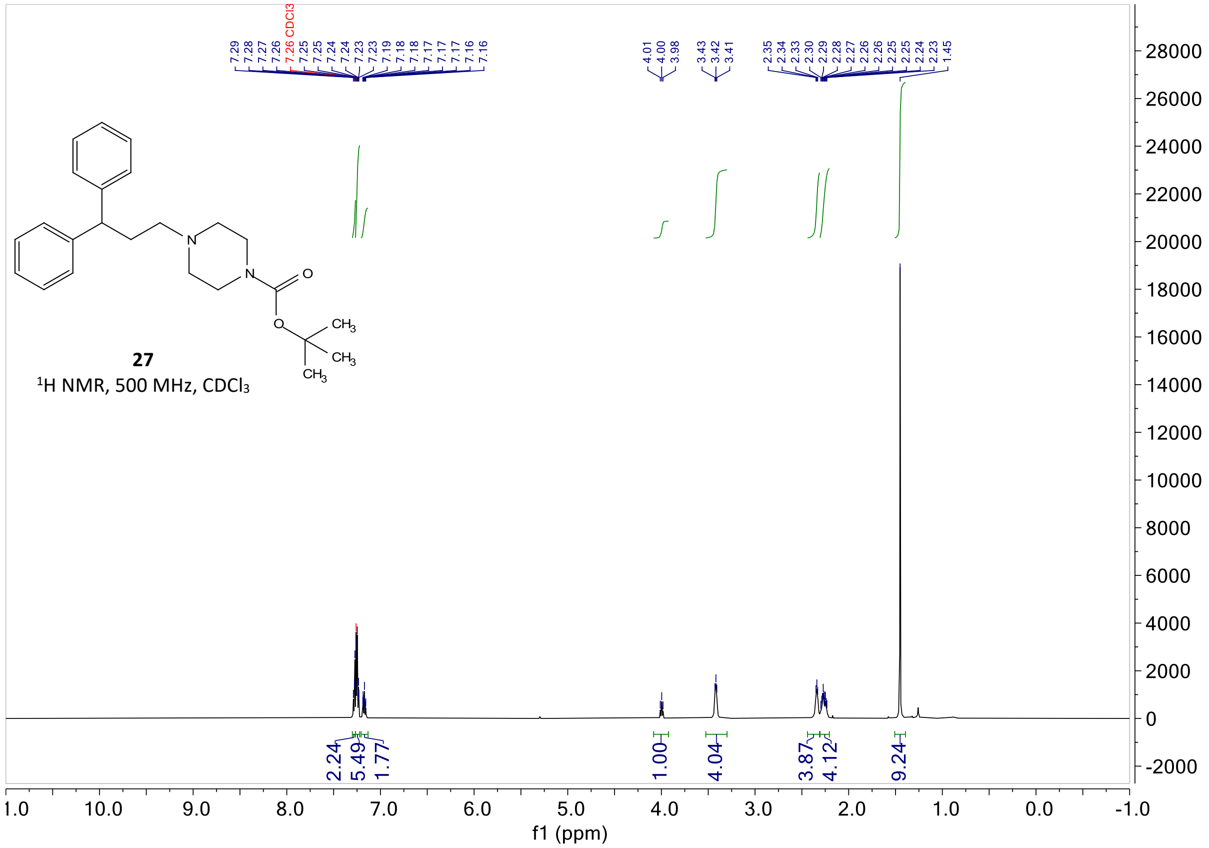

Supplementary Figure 113. ^{1}H NMR spectra of compound **27**

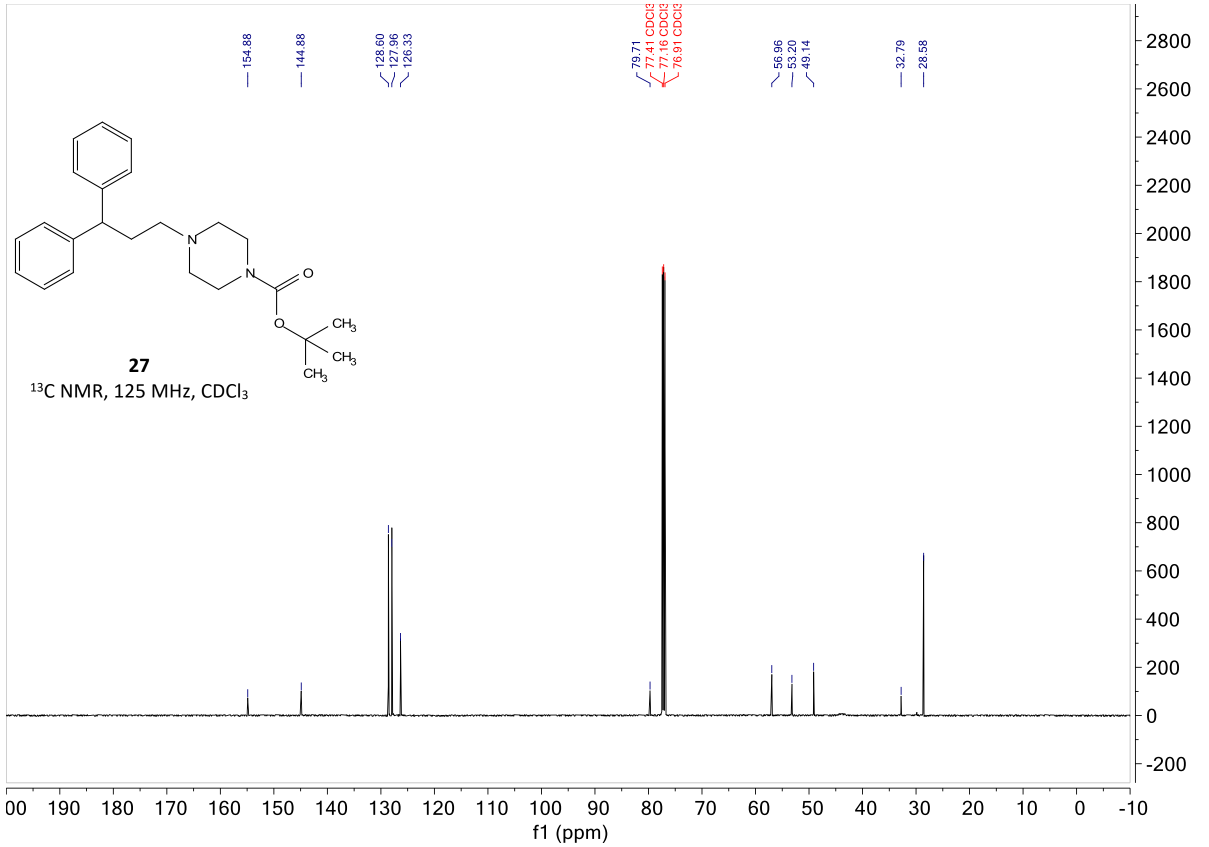

Supplementary Figure 114. ^{13}C NMR spectra of compound **27**

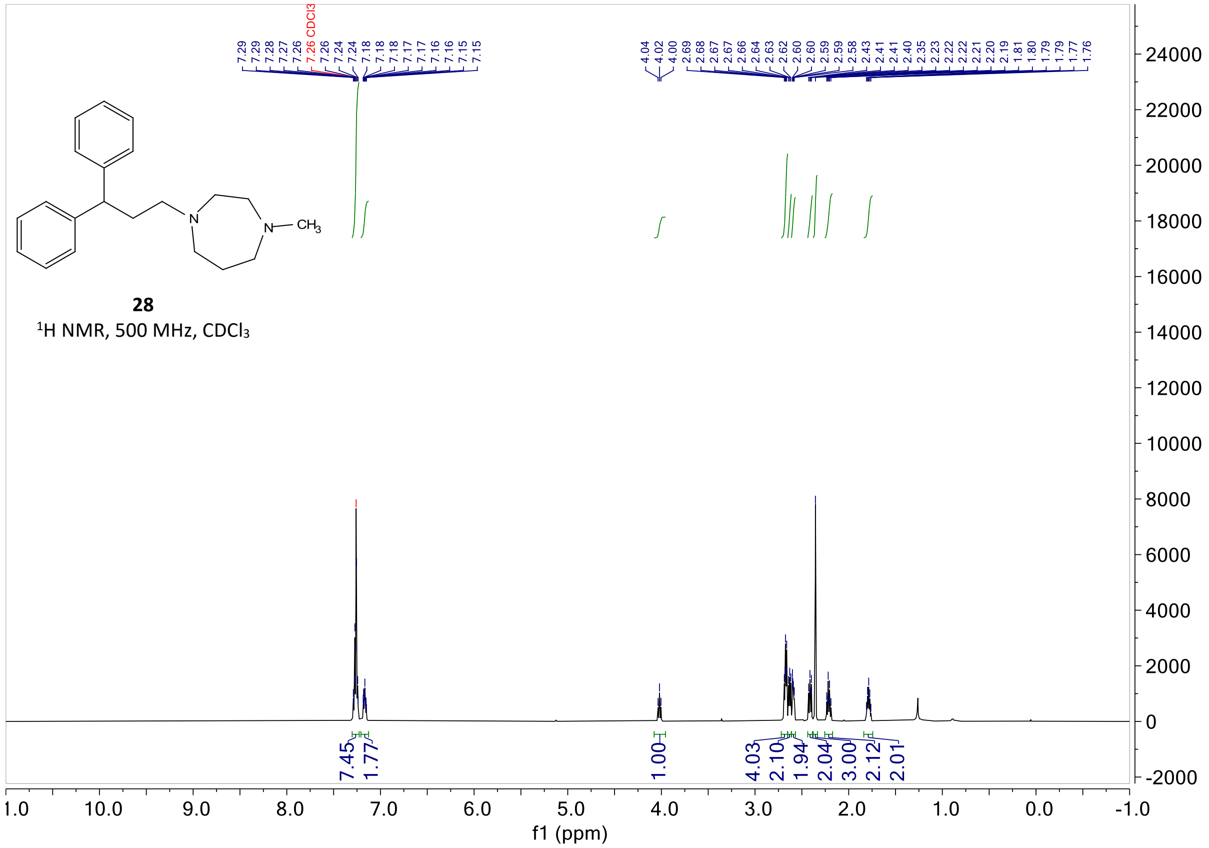

Supplementary Figure 115. ^{1}H NMR spectra of compound **28**

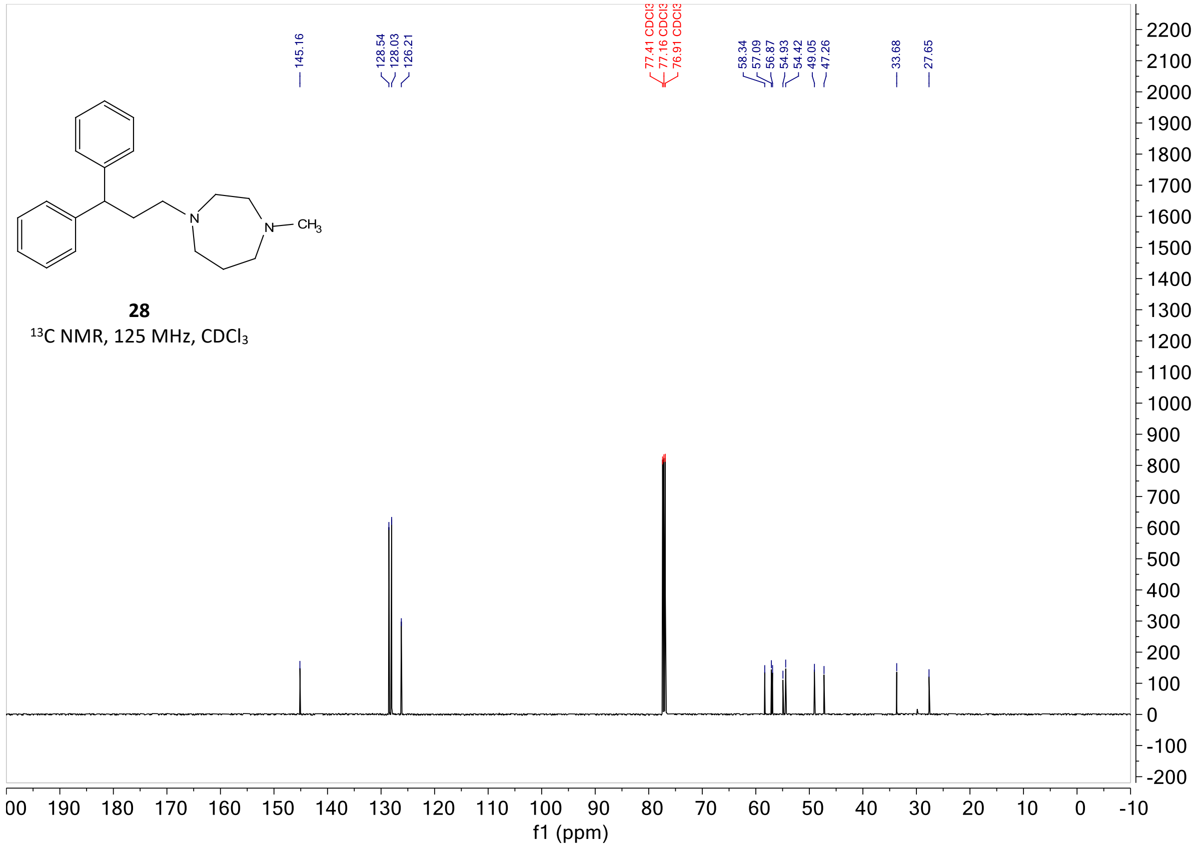

Supplementary Figure 116. ^{13}C NMR spectra of compound **28**

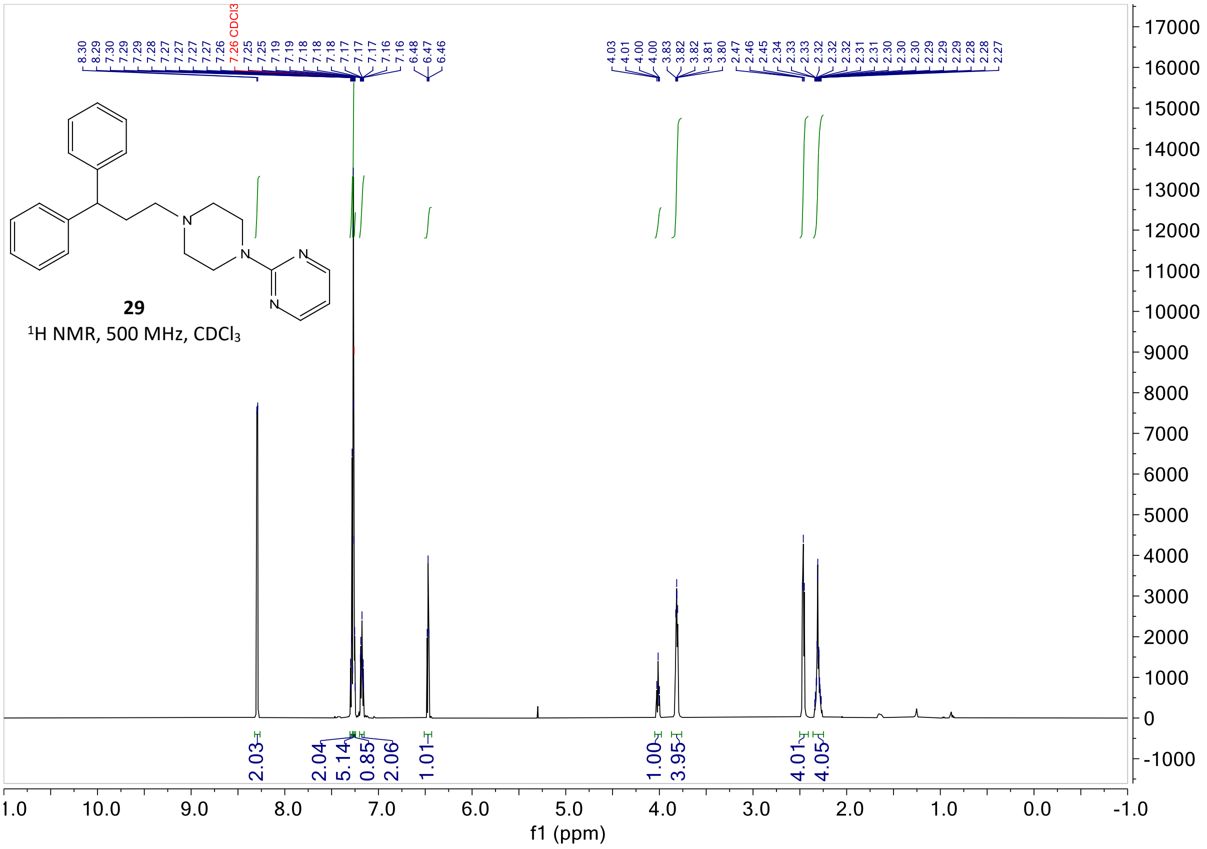

Supplementary Figure 117. ^{1}H NMR spectra of compound **29**

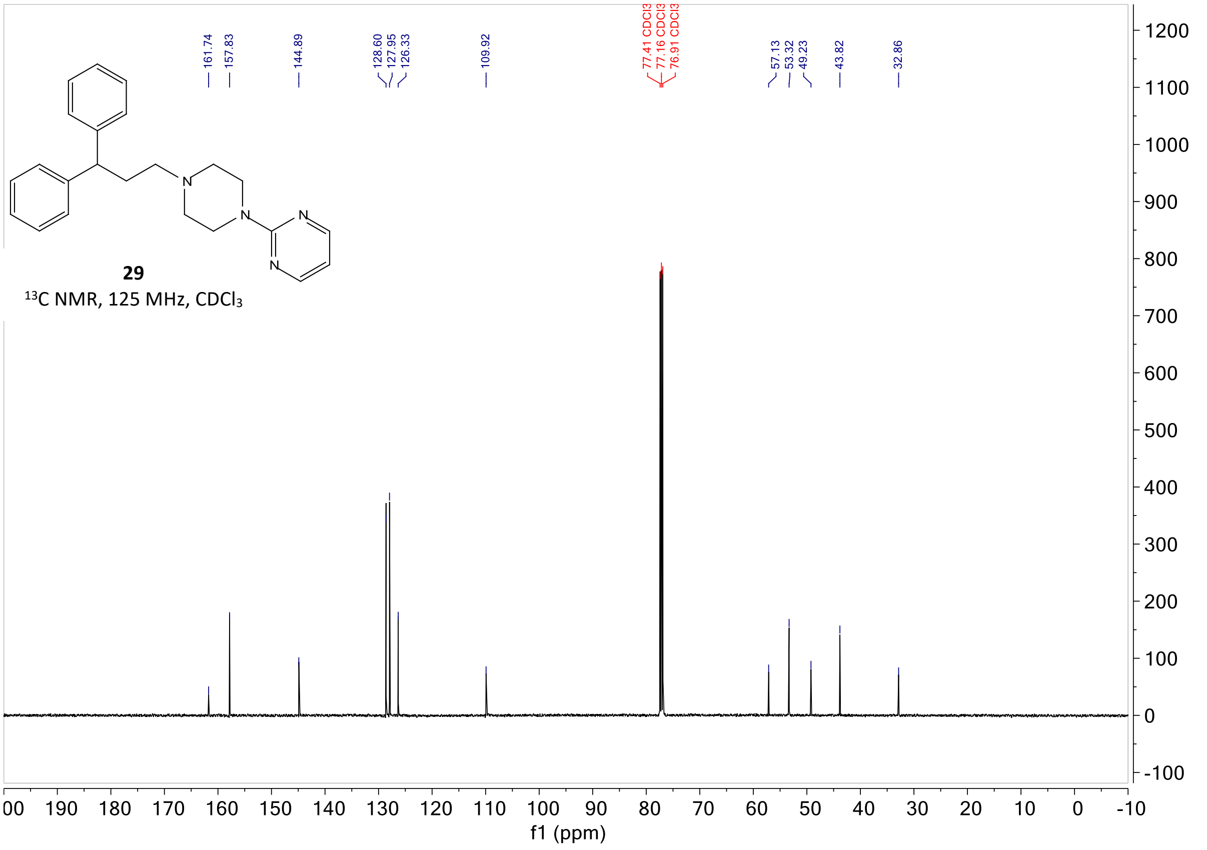

Supplementary Figure 118. ^{13}C NMR spectra of compound **29**

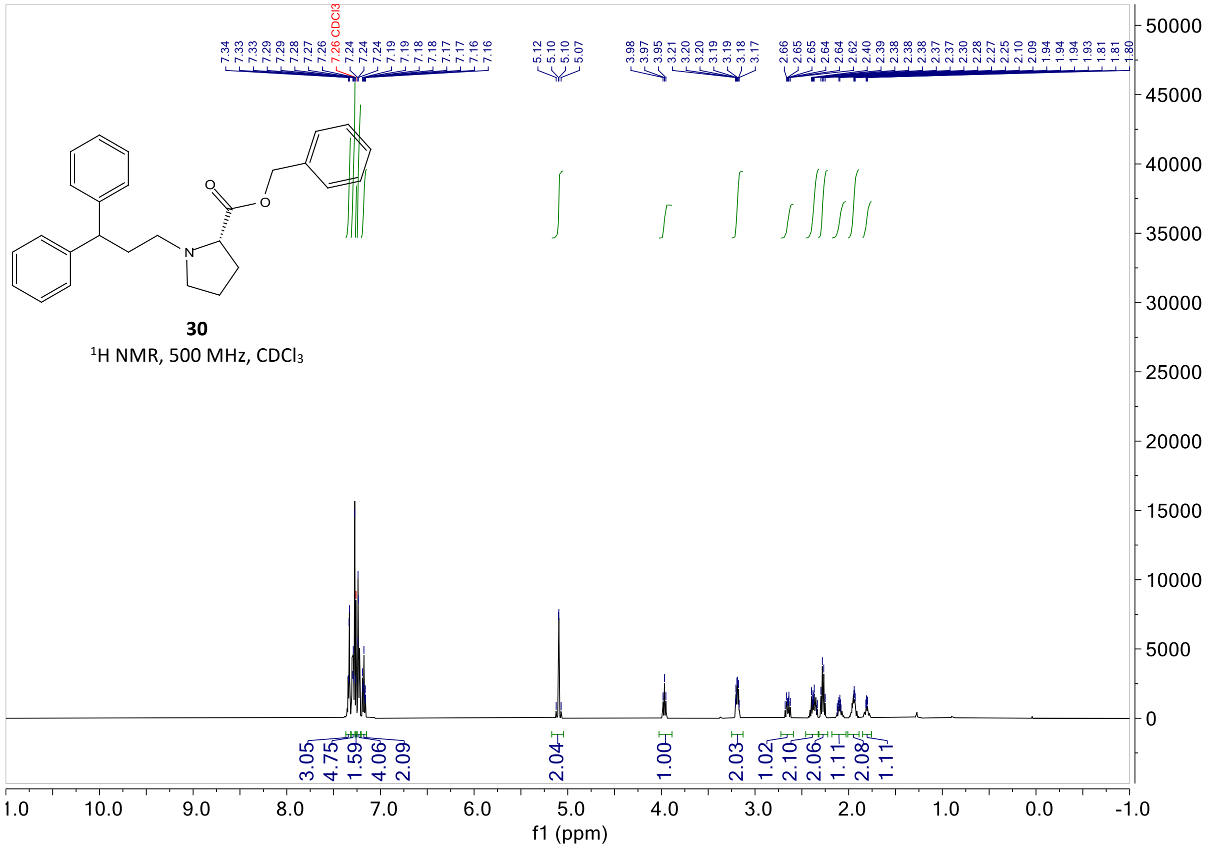

Supplementary Figure 119. ^{1}H NMR spectra of compound **30**

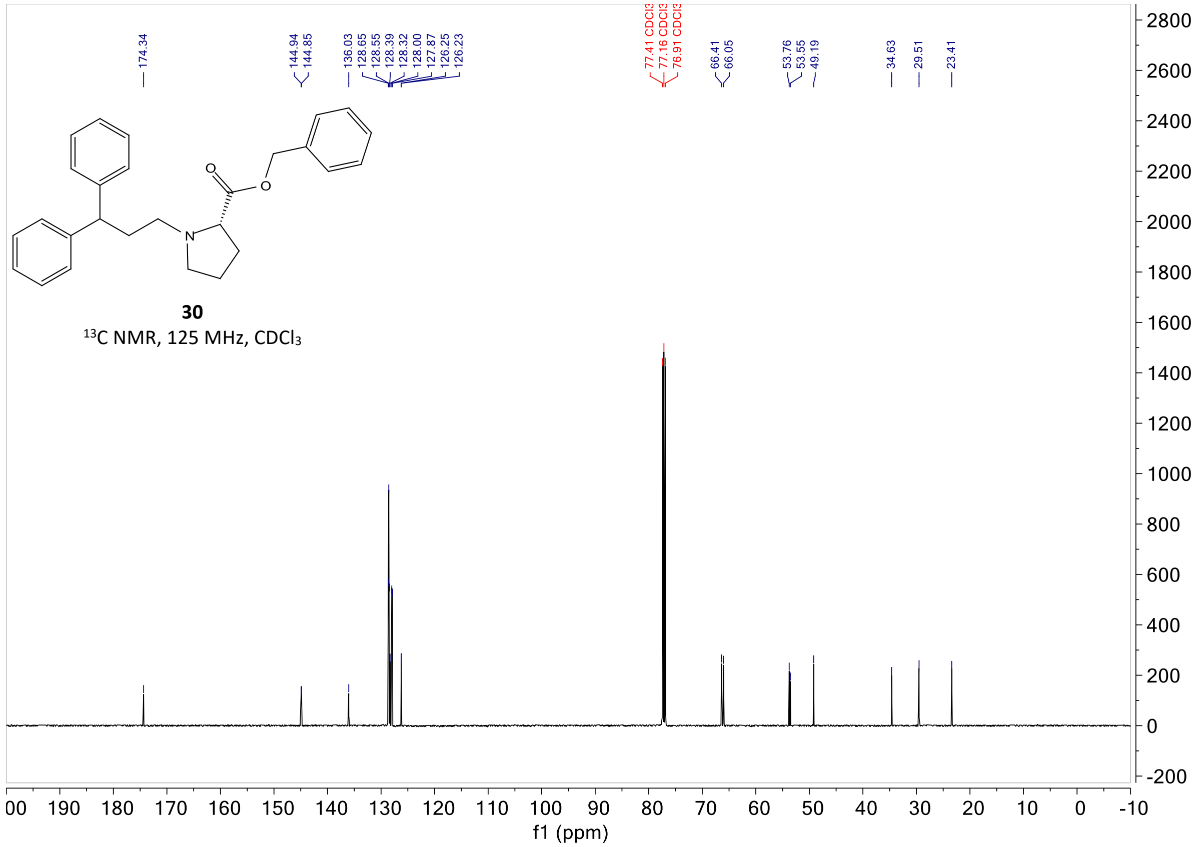

Supplementary Figure 120. ^{13}C NMR spectra of compound **30**

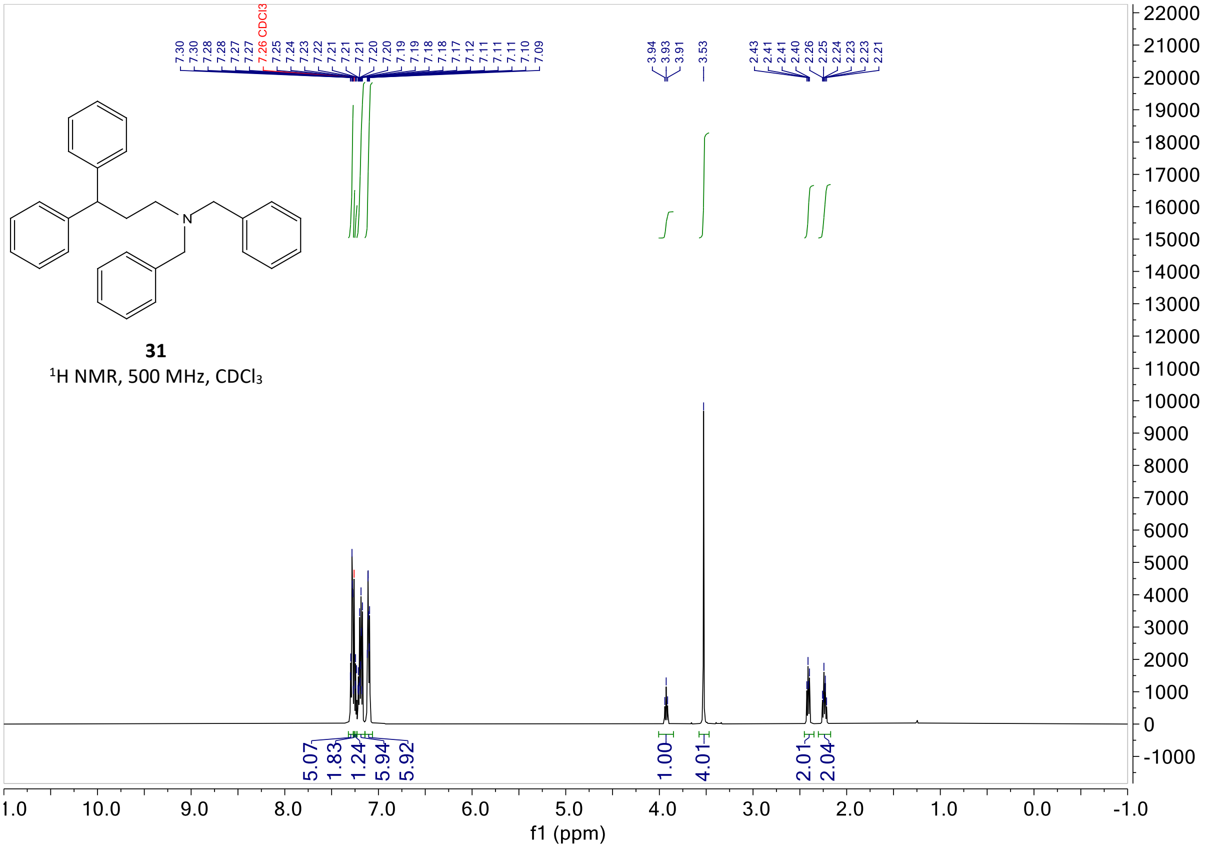

Supplementary Figure 121. ^{1}H NMR spectra of compound **31**

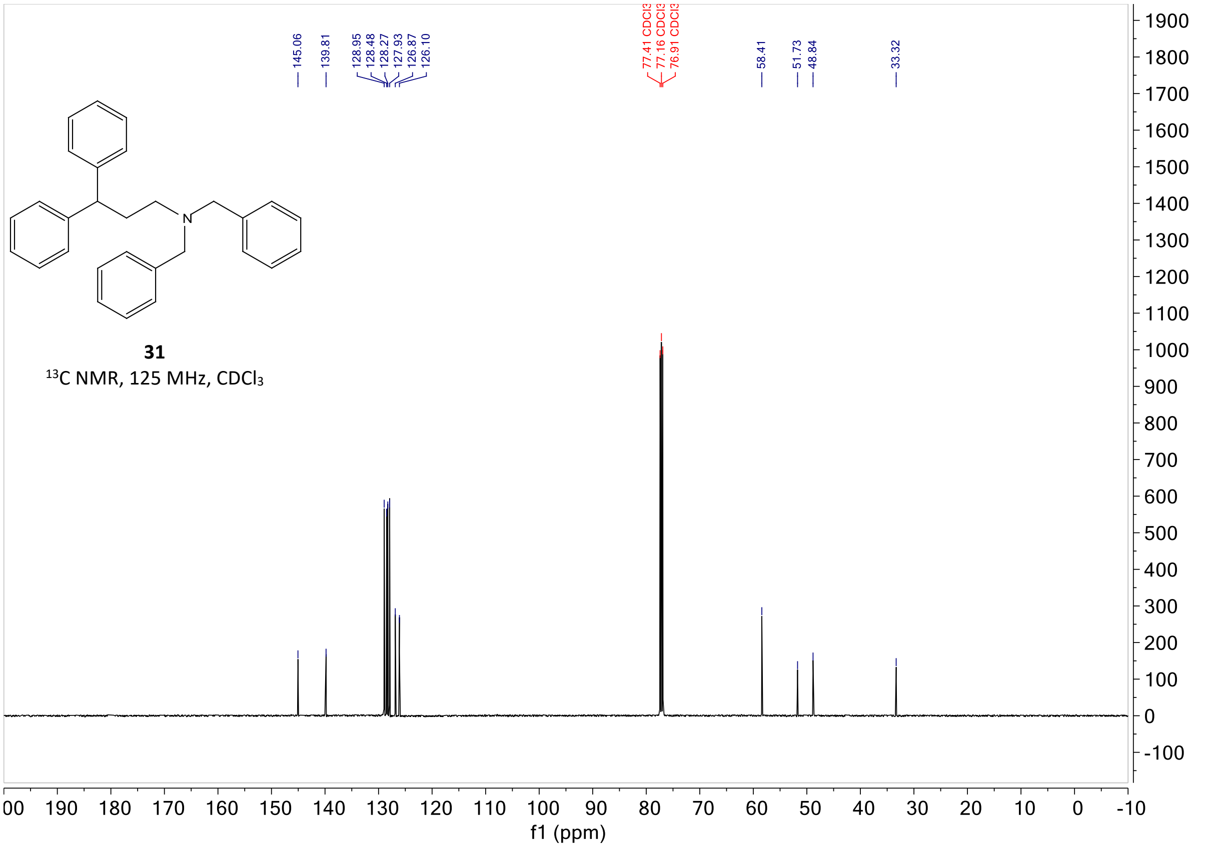

Supplementary Figure 122. ^{13}C NMR spectra of compound **31**

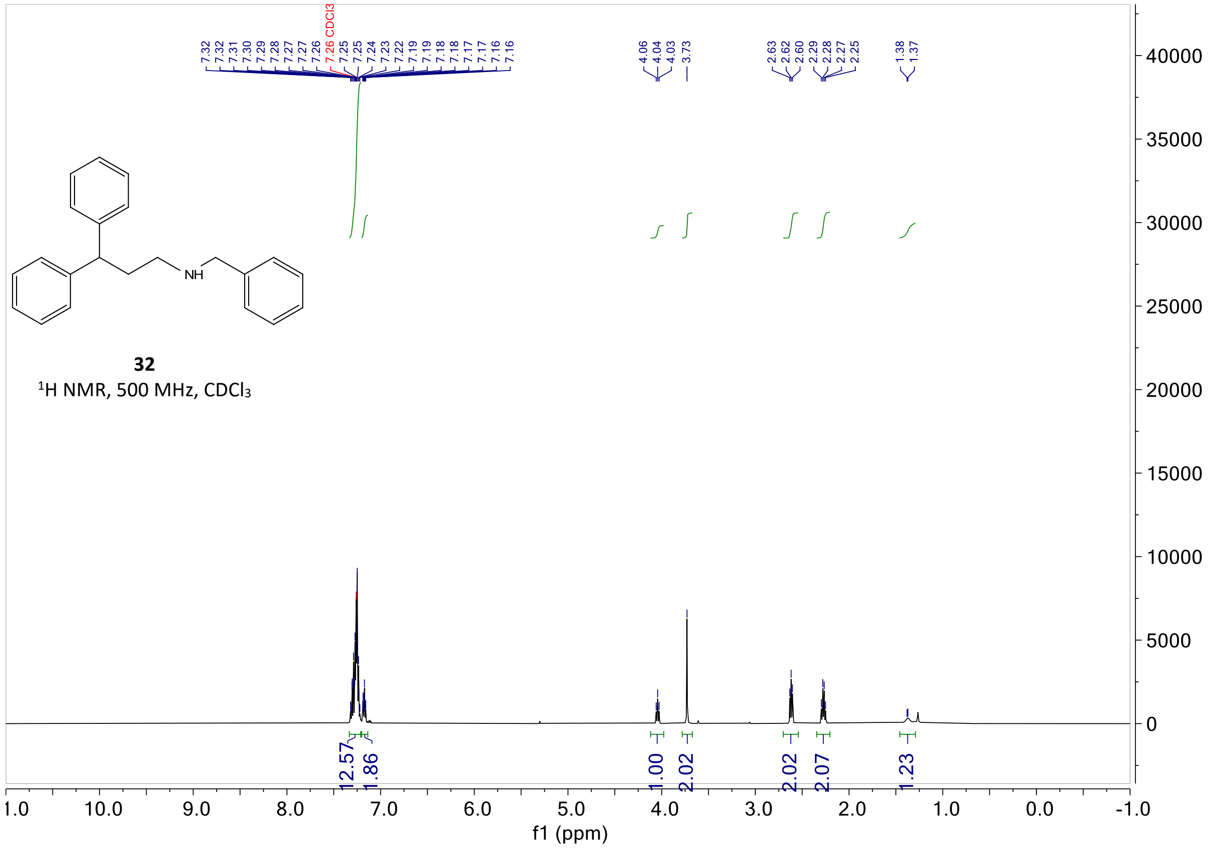

Supplementary Figure 123. ^{1}H NMR spectra of compound **32**

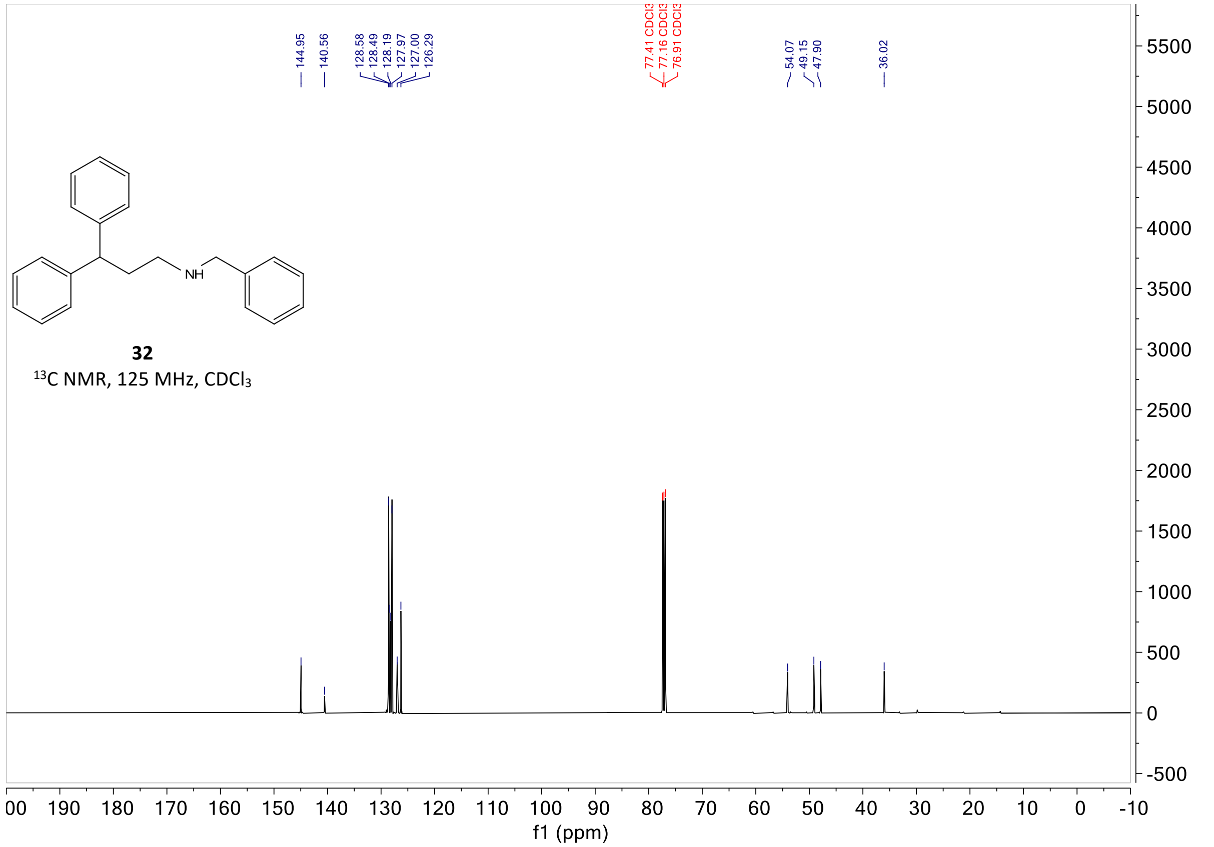

Supplementary Figure 124. ^{13}C NMR spectra of compound **32**

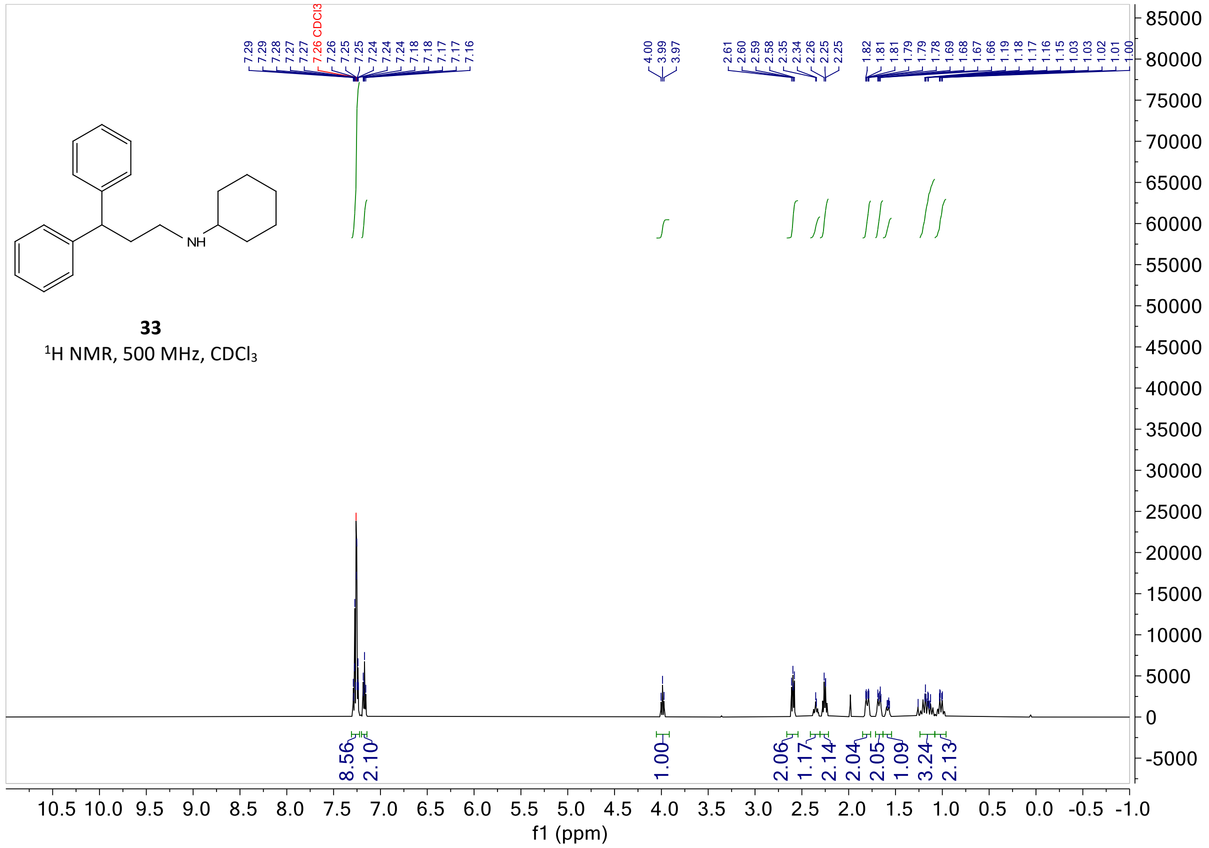

Supplementary Figure 125. ^{1}H NMR spectra of compound **33**

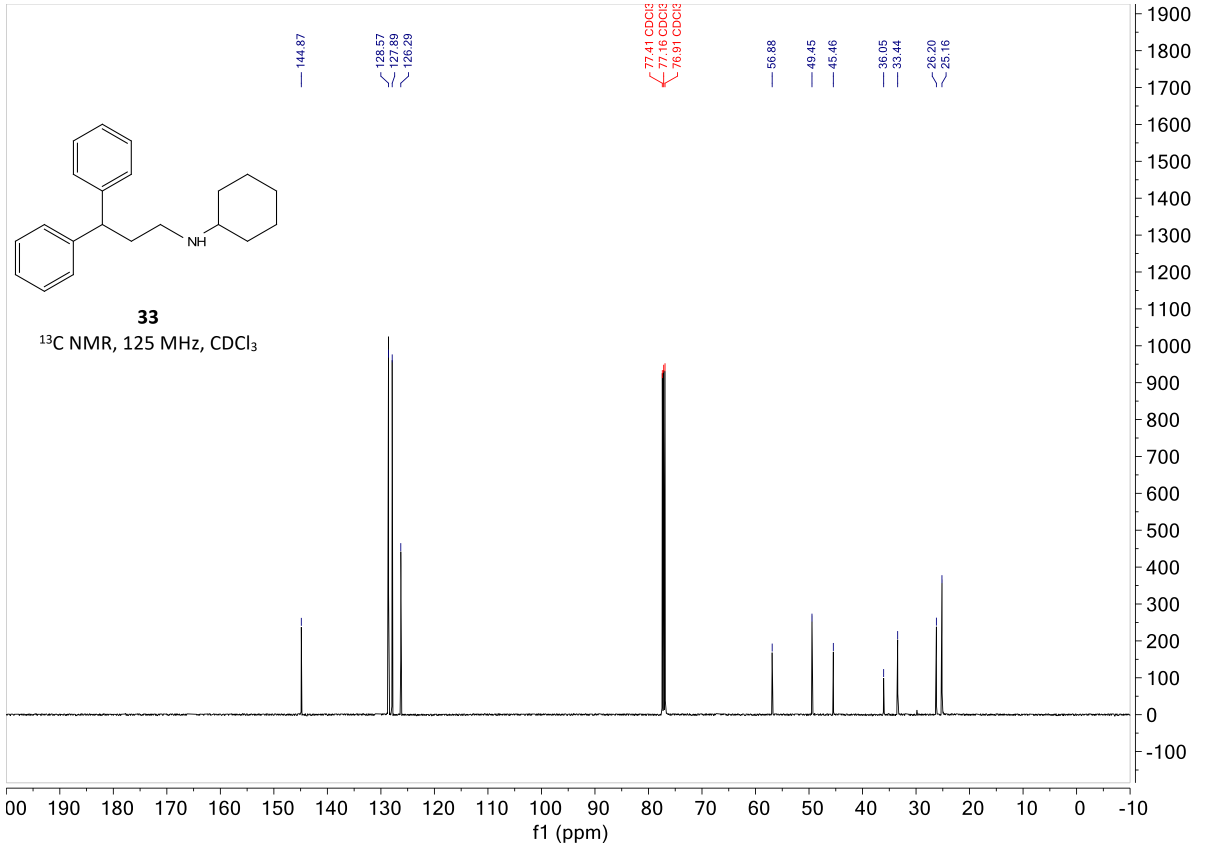

Supplementary Figure 126. ^{13}C NMR spectra of compound **33**

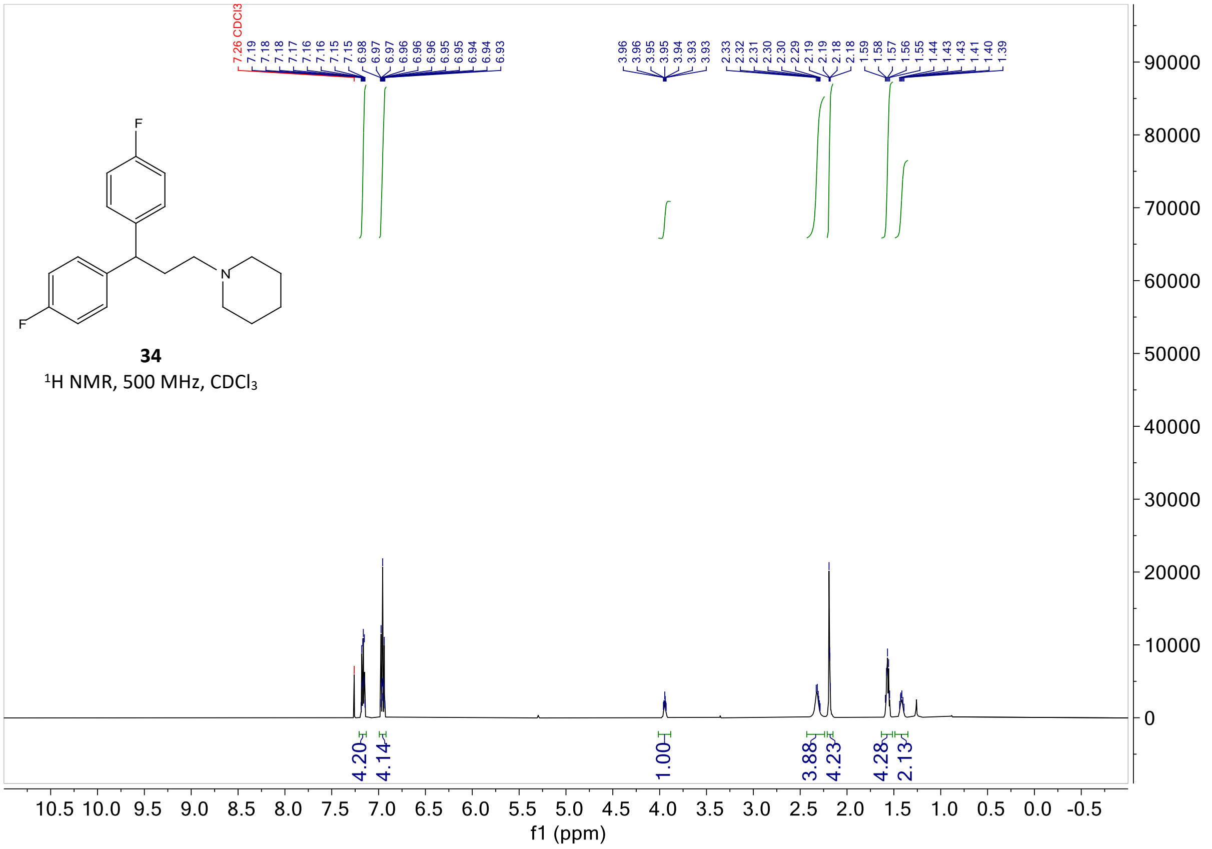

Supplementary Figure 127. ^{1}H NMR spectra of compound **34**

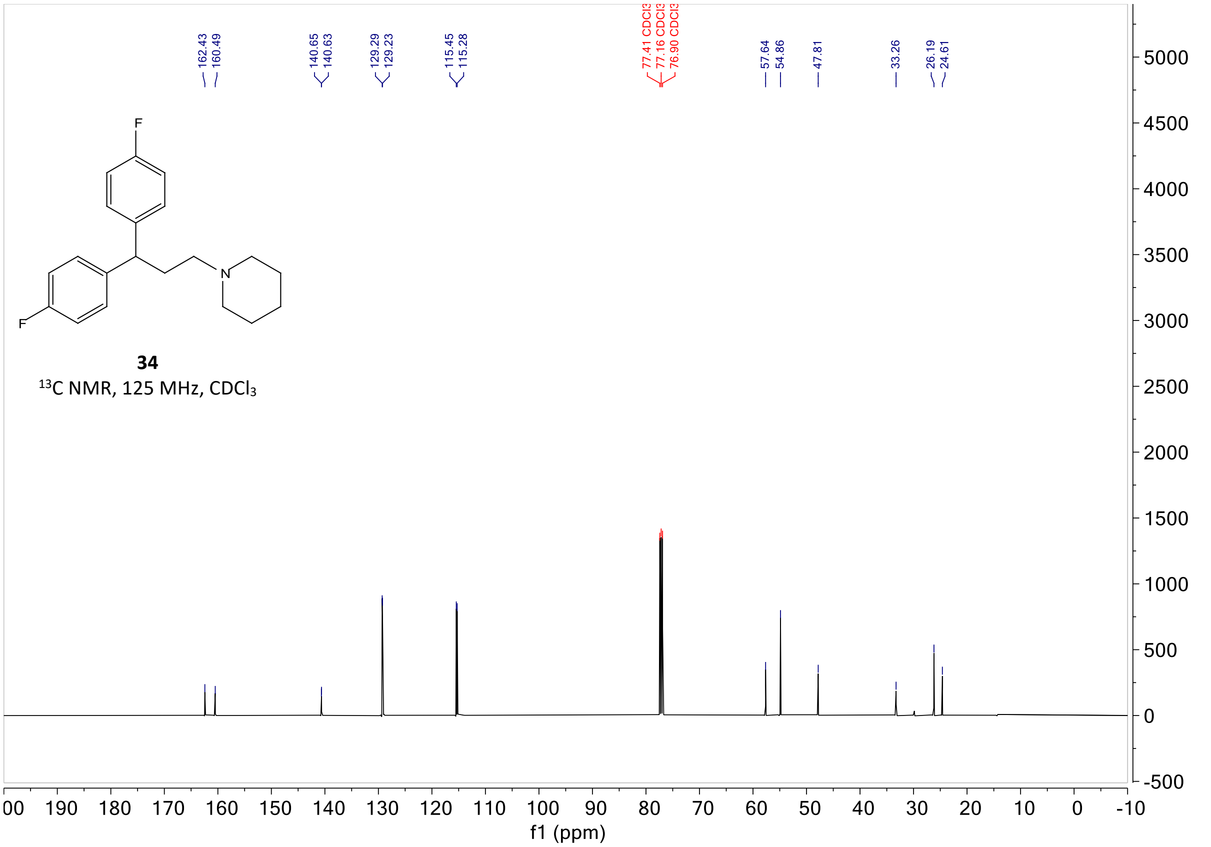

Supplementary Figure 128. ^{13}C NMR spectra of compound **34**

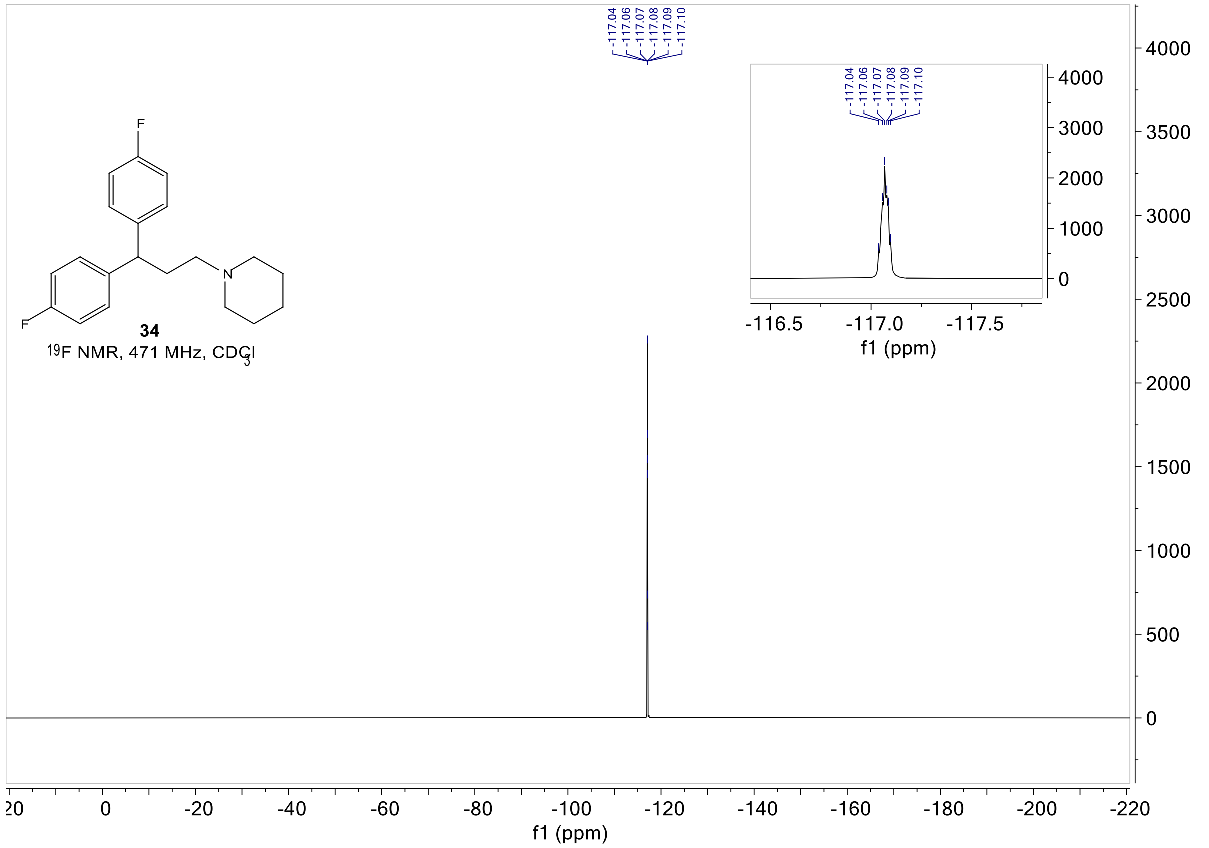

Supplementary Figure 129. ^{19}F NMR spectra of compound **34**

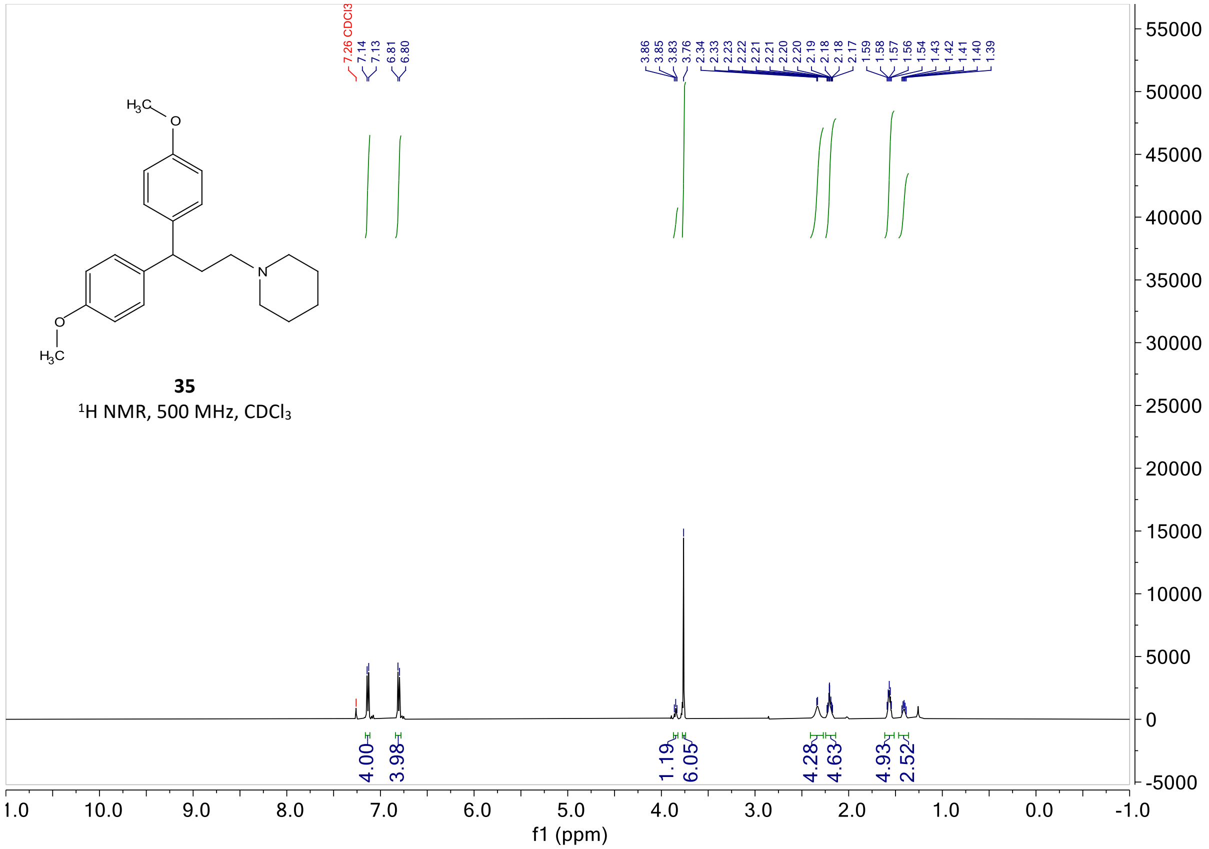

Supplementary Figure 130. ^{1}H NMR spectra of compound **35**

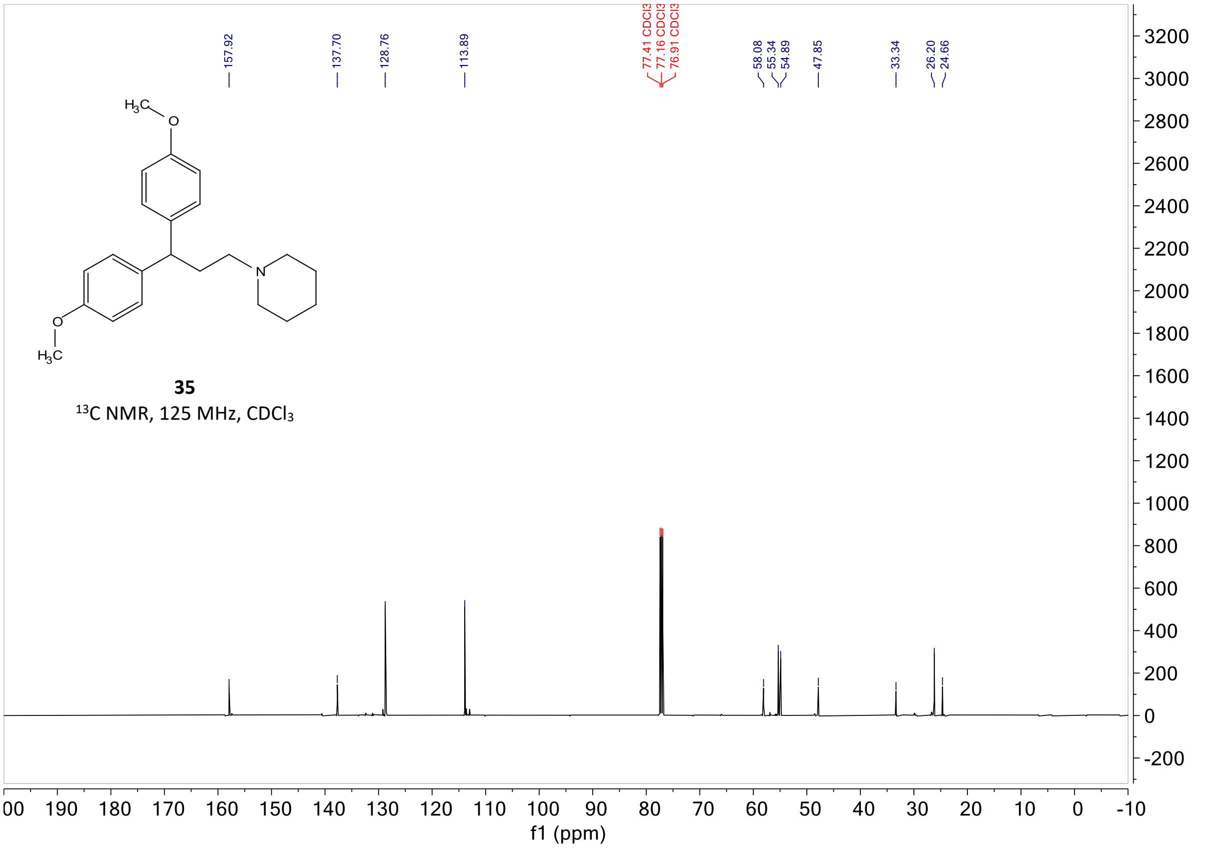

Supplementary Figure 131. ^{13}C NMR spectra of compound **35**

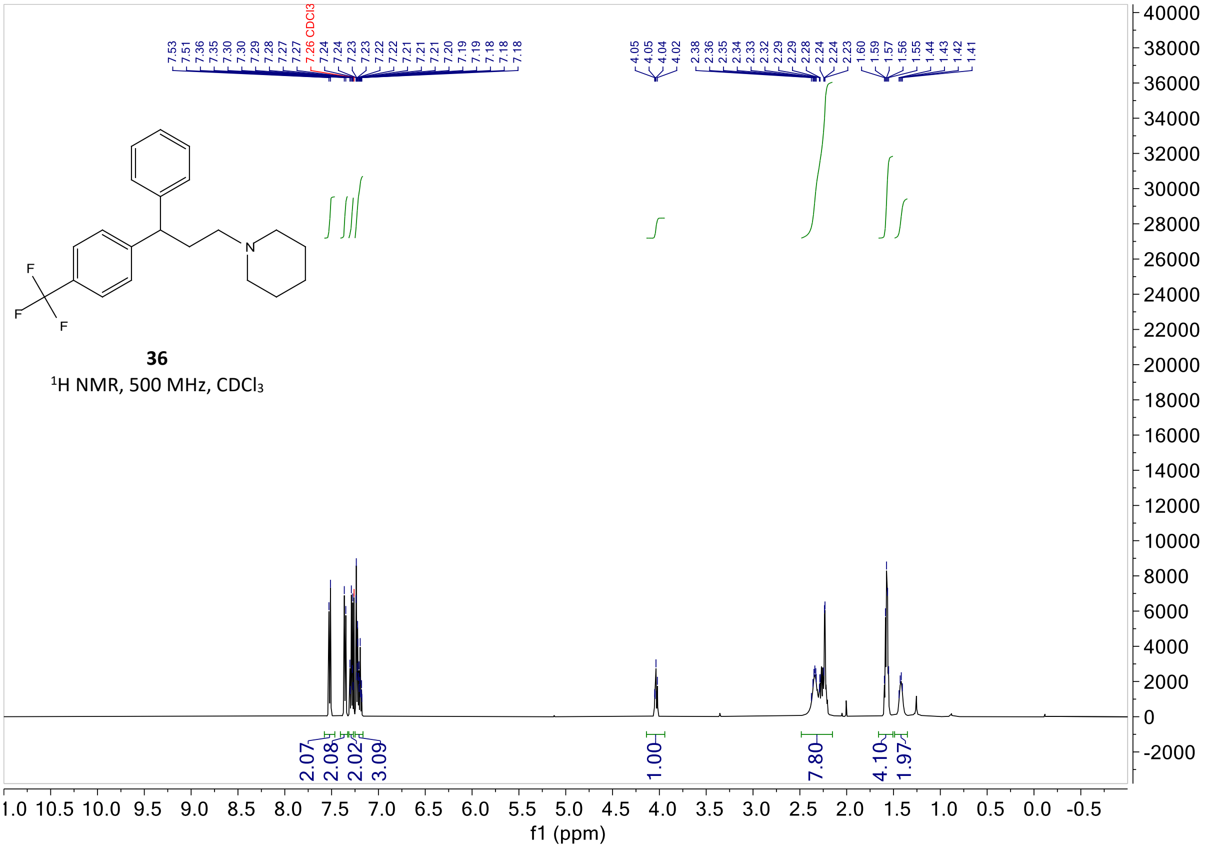

Supplementary Figure 132. ^{1}H NMR spectra of compound **36**

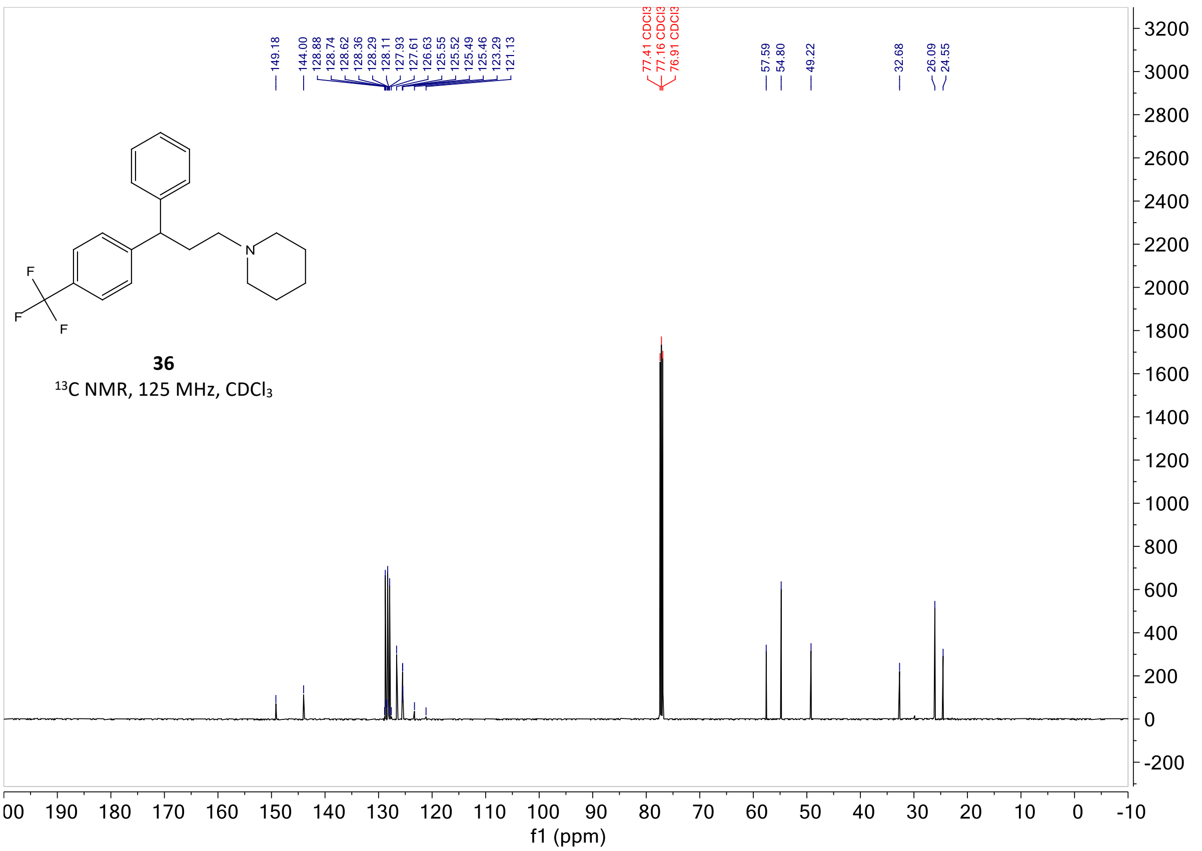

Supplementary Figure 133. ^{13}C NMR spectra of compound **36**

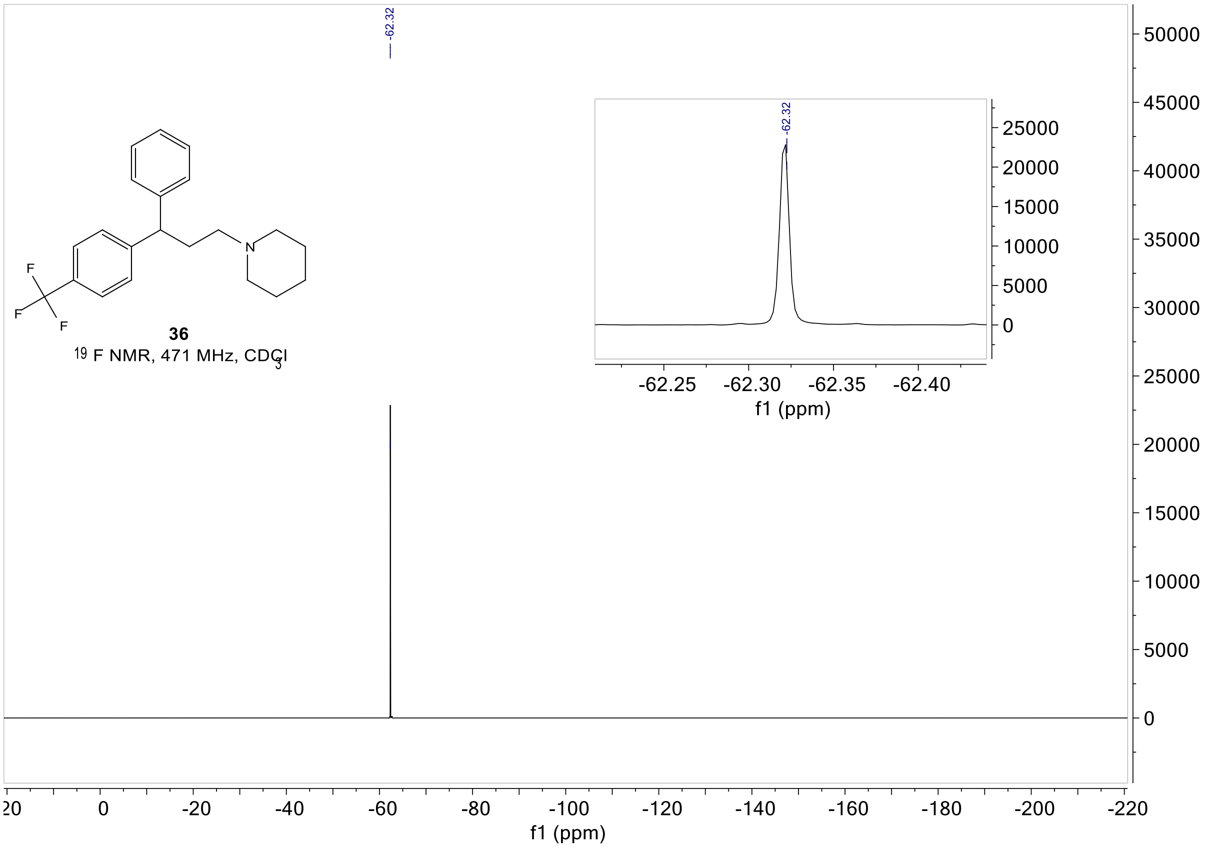

Supplementary Figure 134. ^{19}F NMR spectra of compound **36**

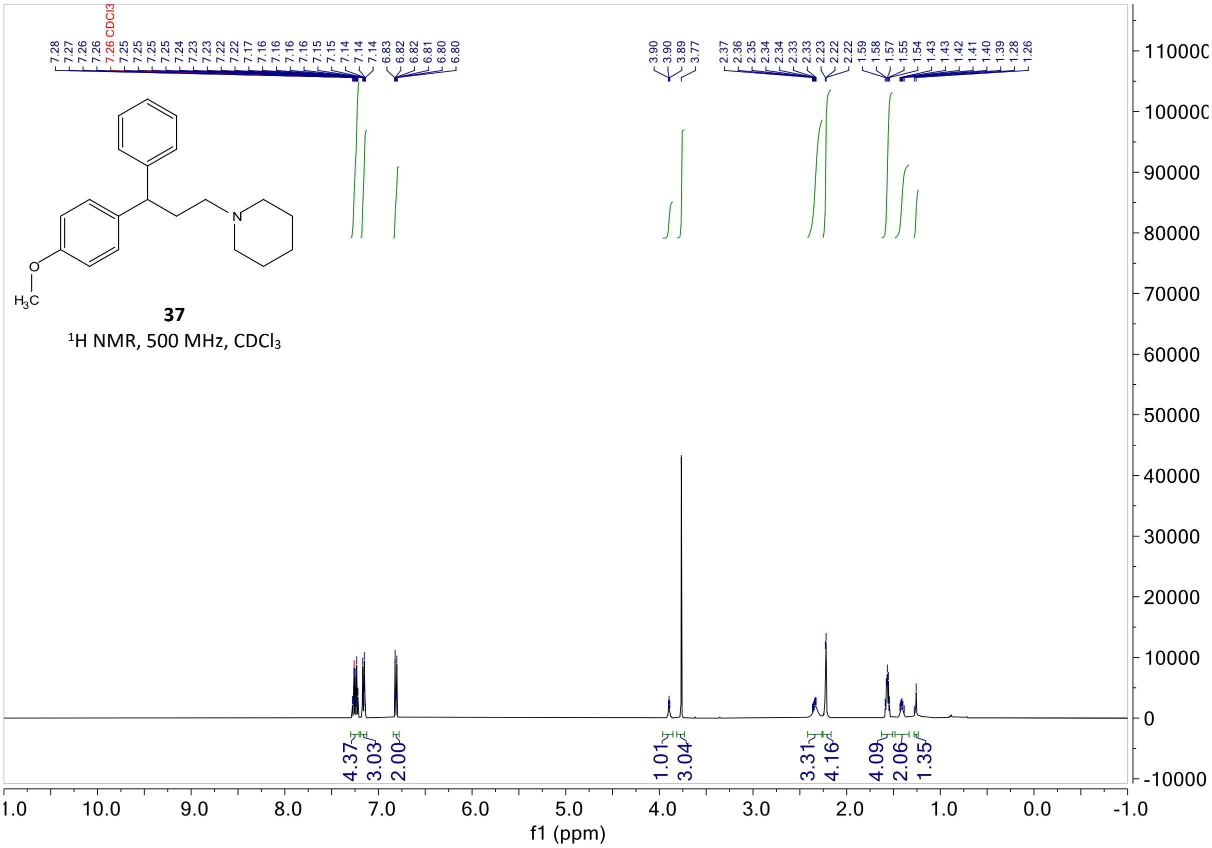

Supplementary Figure 135. ^{1}H NMR spectra of compound **37**

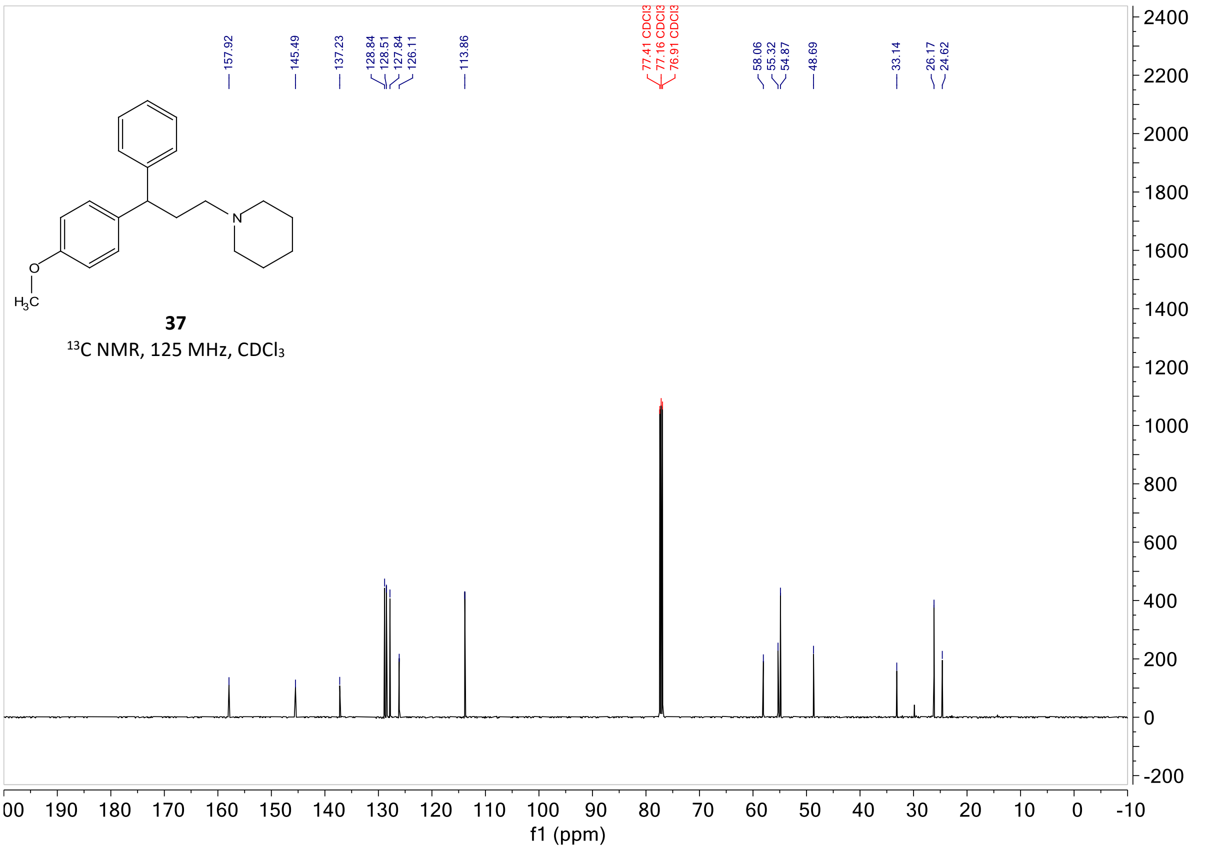

Supplementary Figure 136. ^{13}C NMR spectra of compound **37**

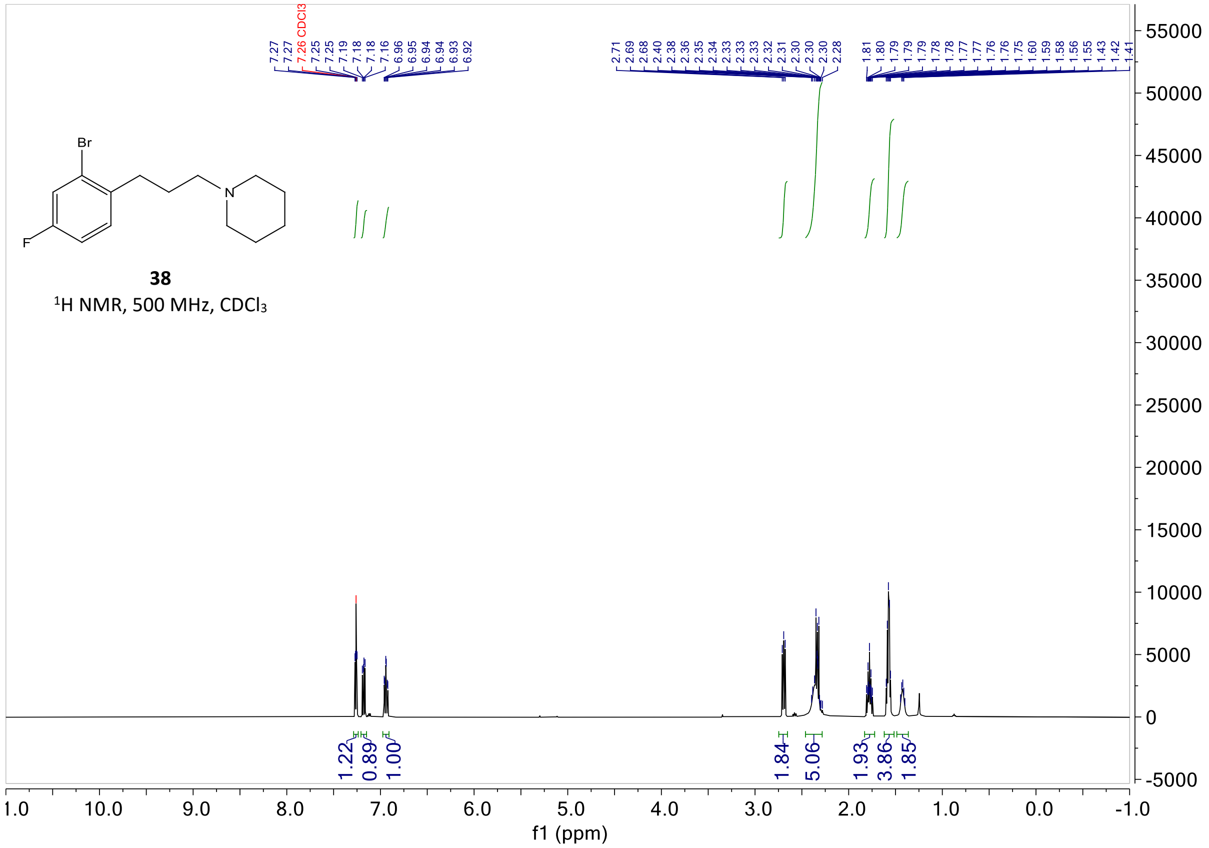

Supplementary Figure 137. ^{1}H NMR spectra of compound **38**

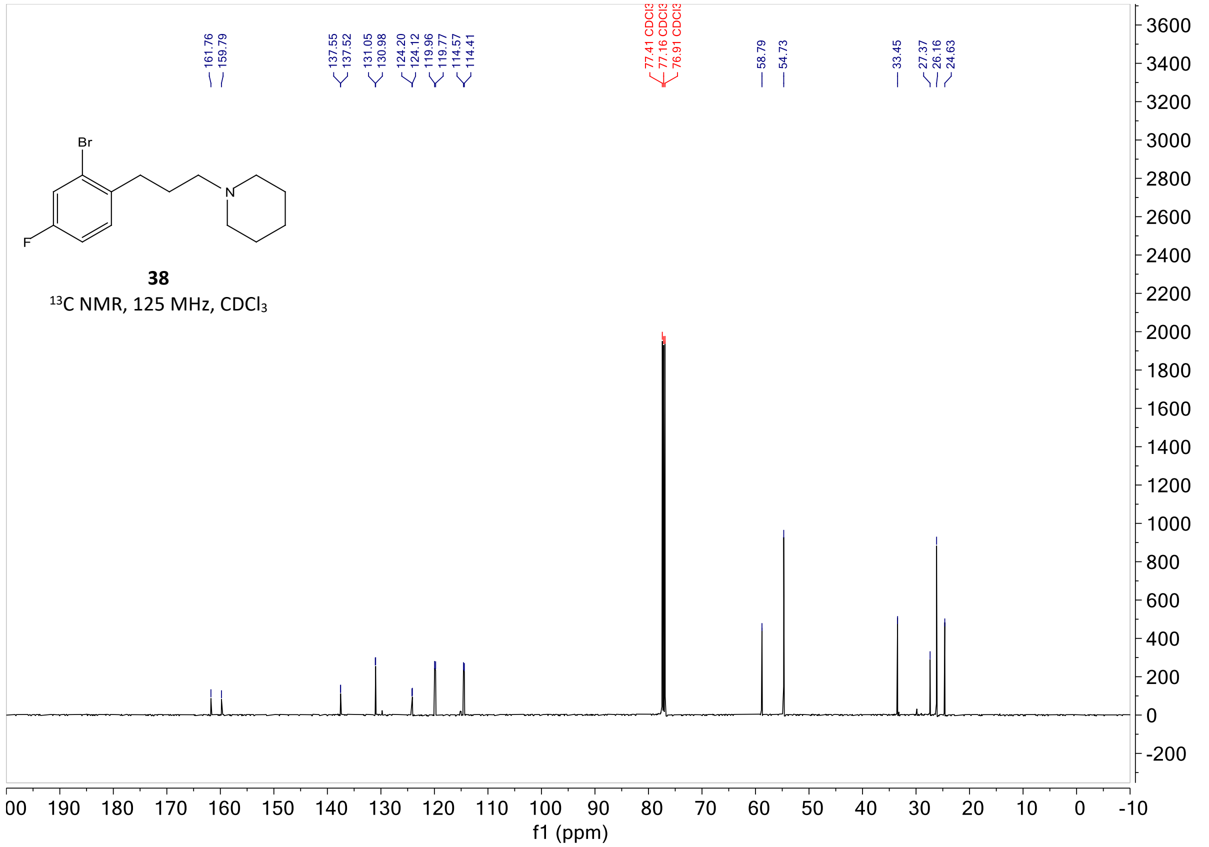

Supplementary Figure 138. ^{13}C NMR spectra of compound **38**

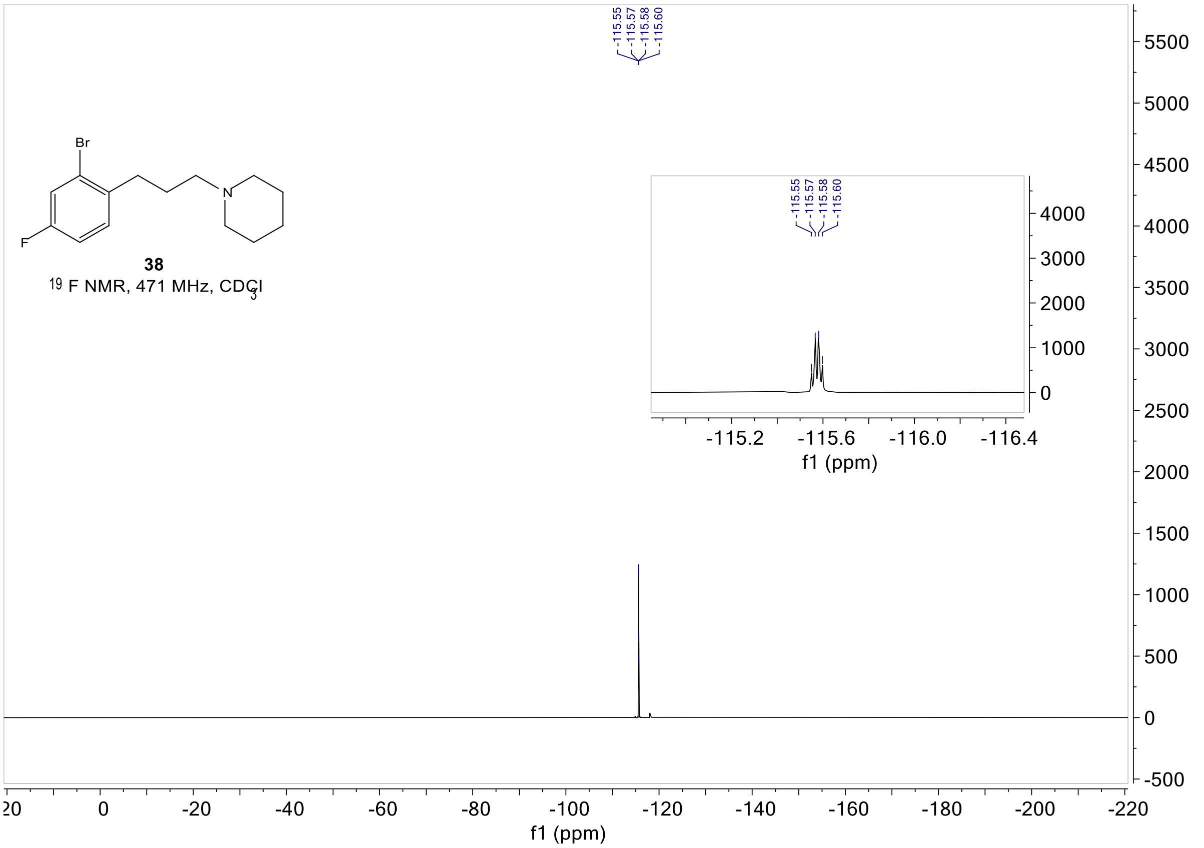

Supplementary Figure 139. ^{19}F NMR spectra of compound **38**

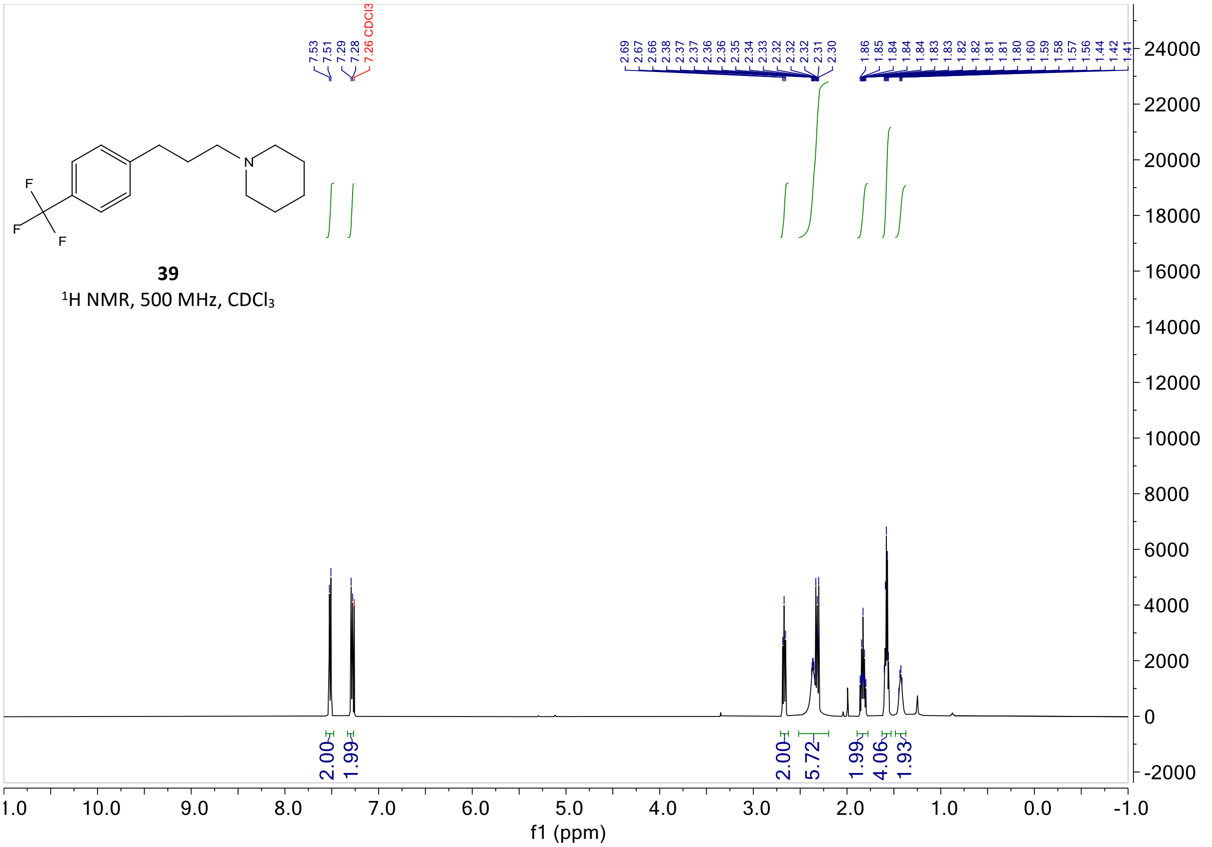

Supplementary Figure 140. ^{1}H NMR spectra of compound **39**

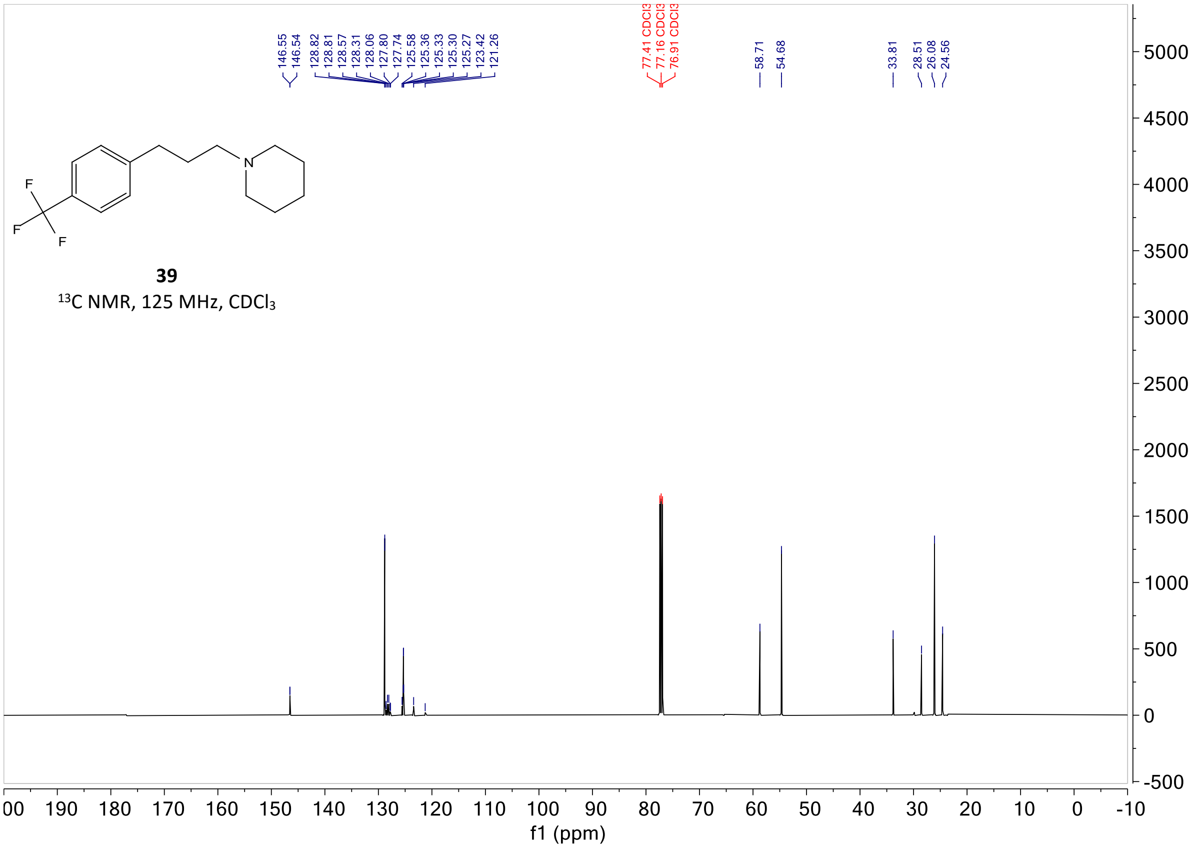

Supplementary Figure 141. ^{13}C NMR spectra of compound **39**

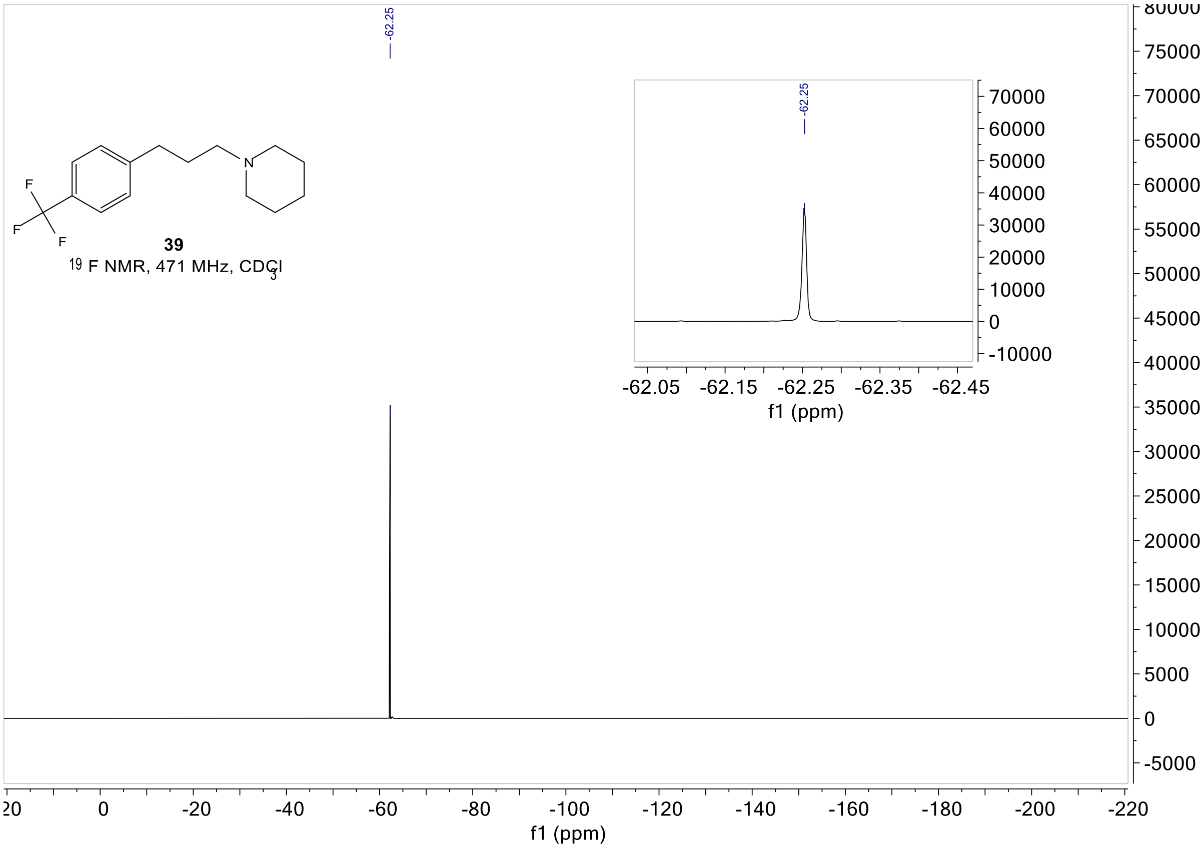

Supplementary Figure 142. ^{19}F NMR spectra of compound **39**

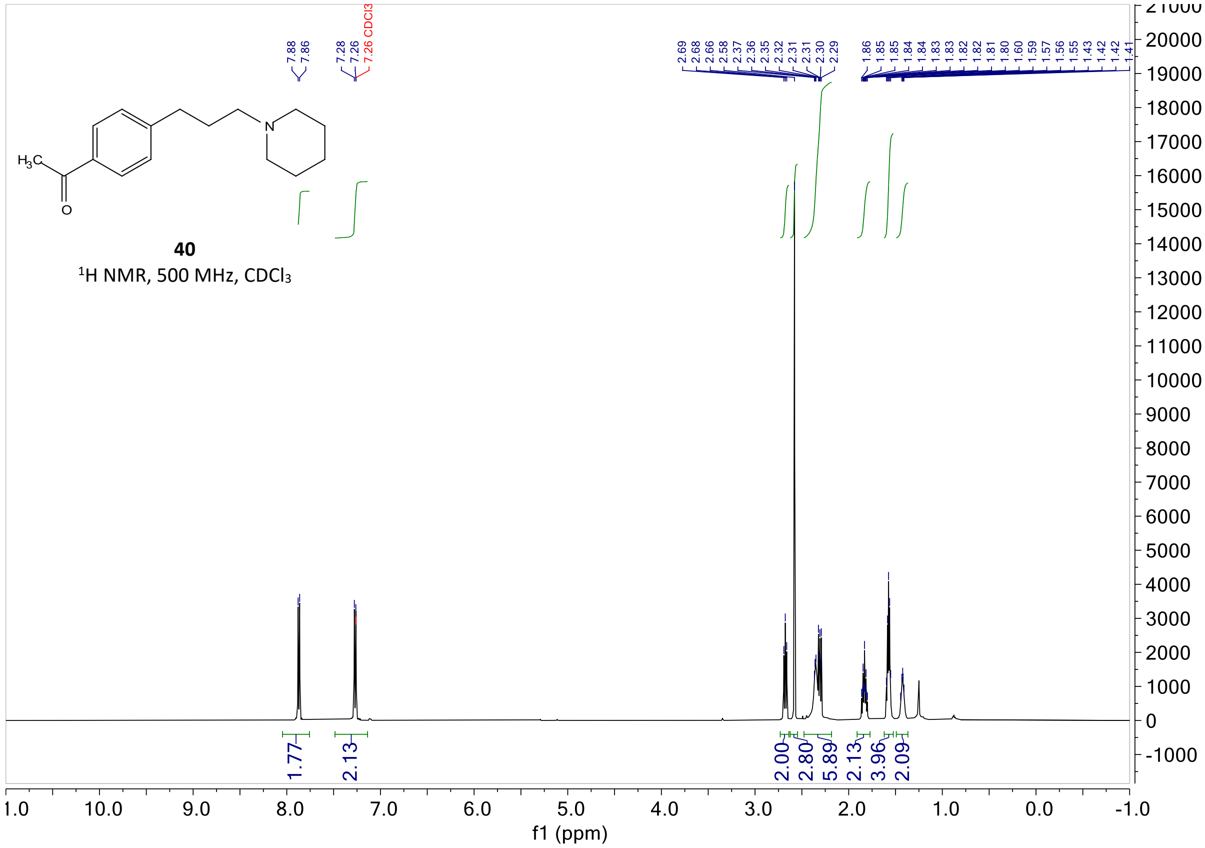

Supplementary Figure 143. ^{1}H NMR spectra of compound **40**

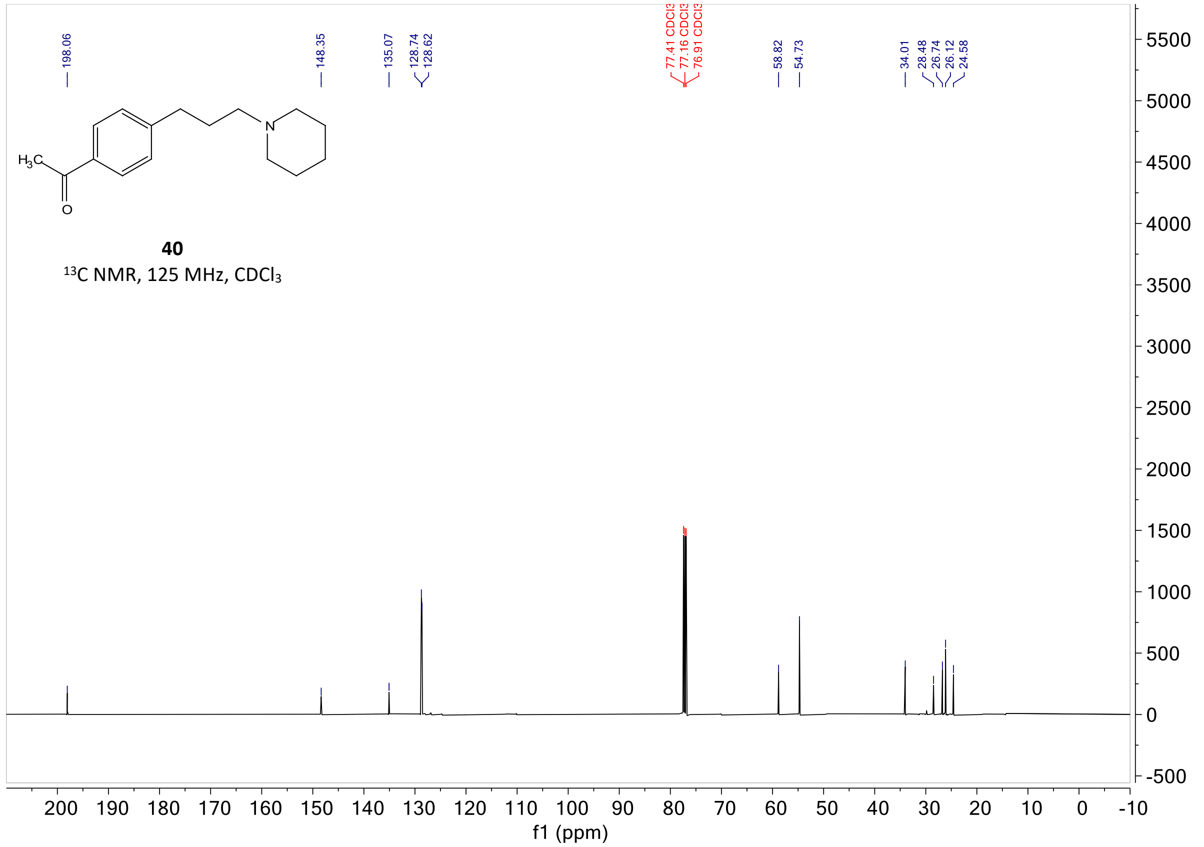

Supplementary Figure 144. ^{13}C NMR spectra of compound **40**

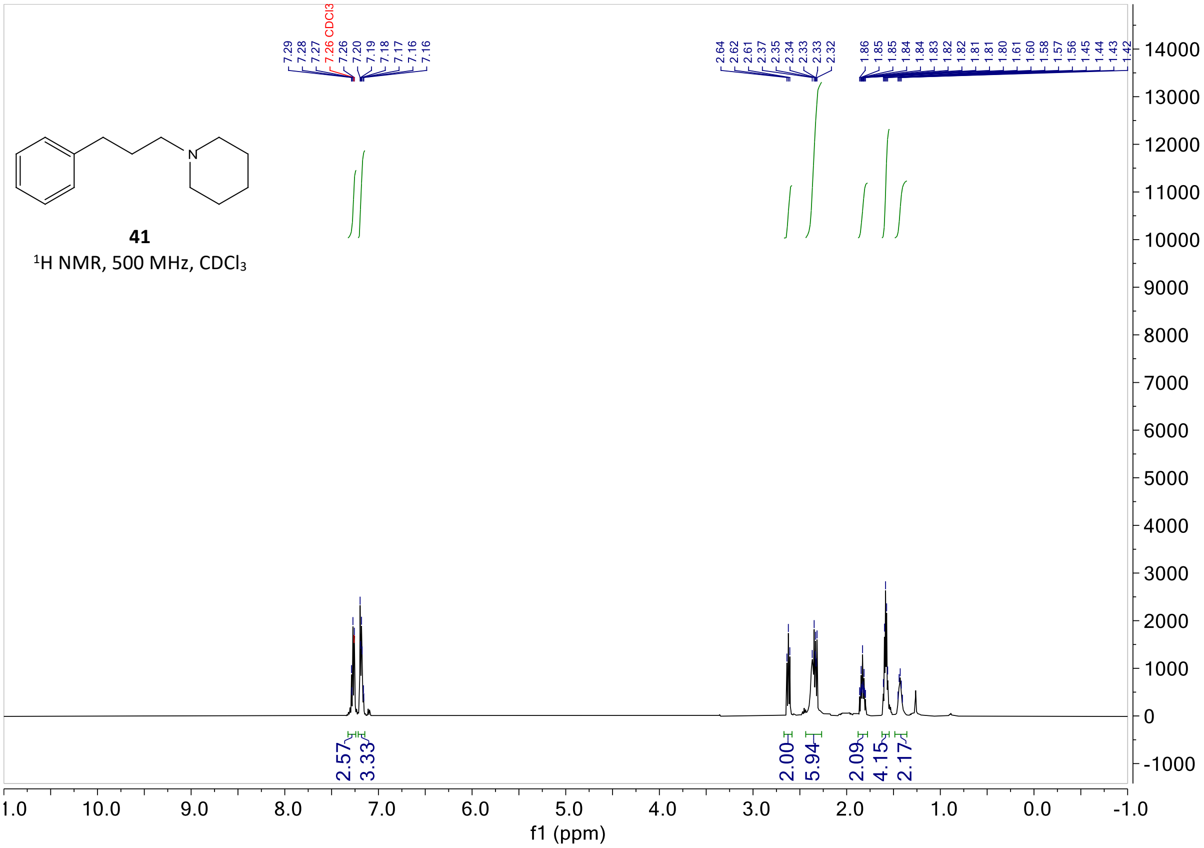

Supplementary Figure 145. ^{1}H NMR spectra of compound **41**

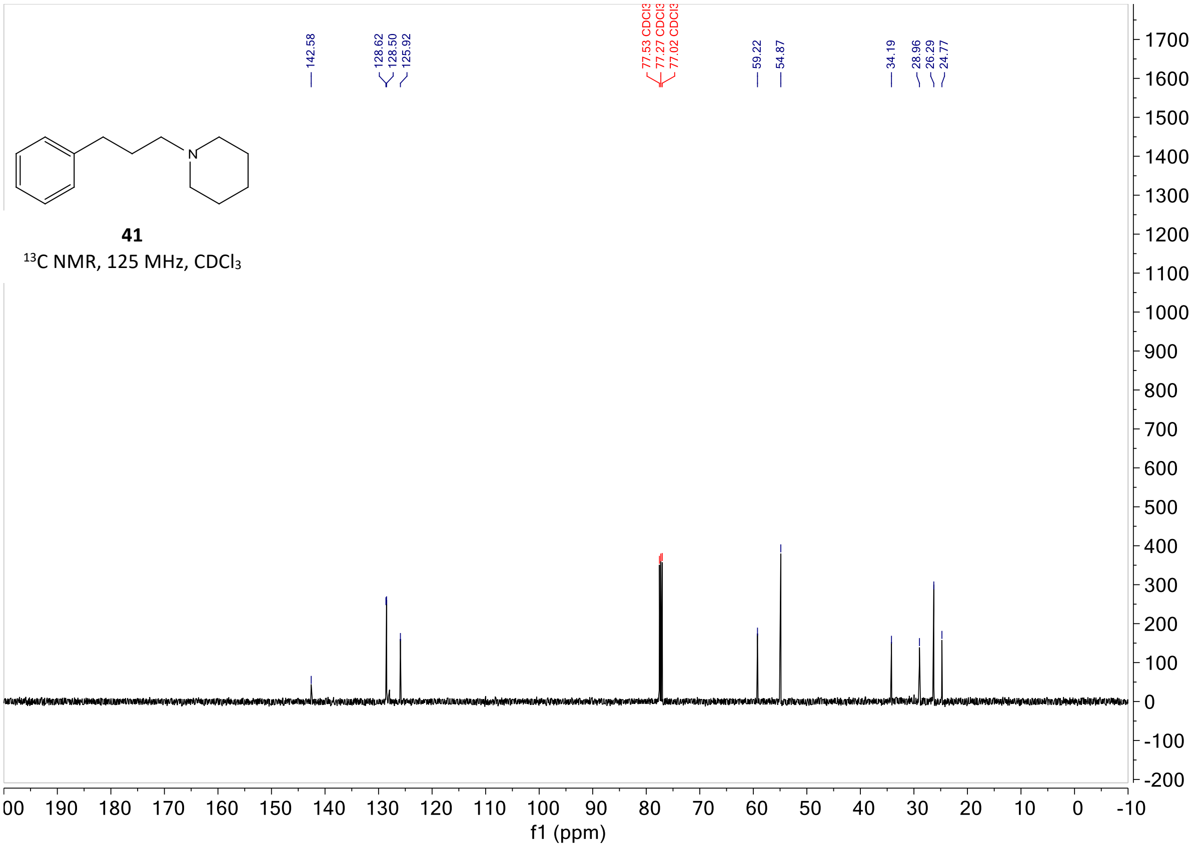

Supplementary Figure 146. ^{13}C NMR spectra of compound **41**

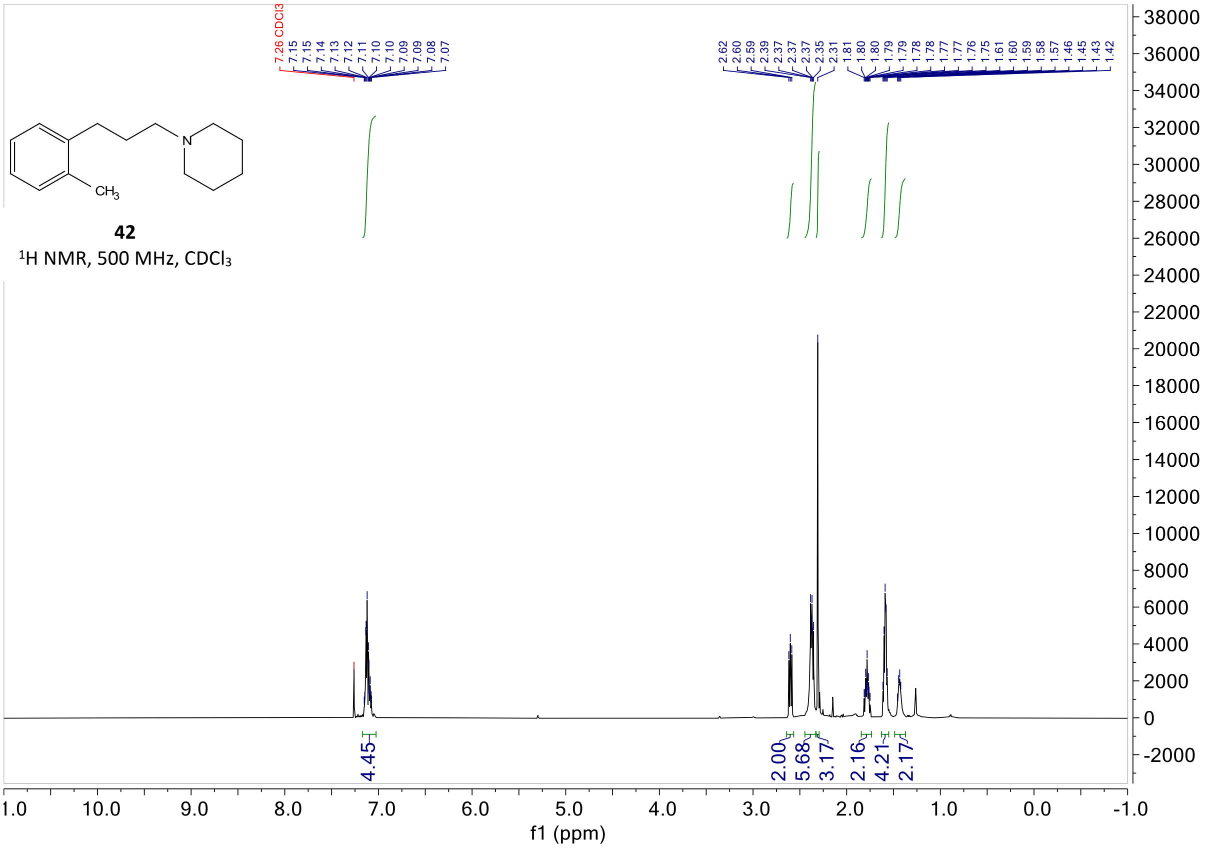

Supplementary Figure 147. ^{1}H NMR spectra of compound **42**

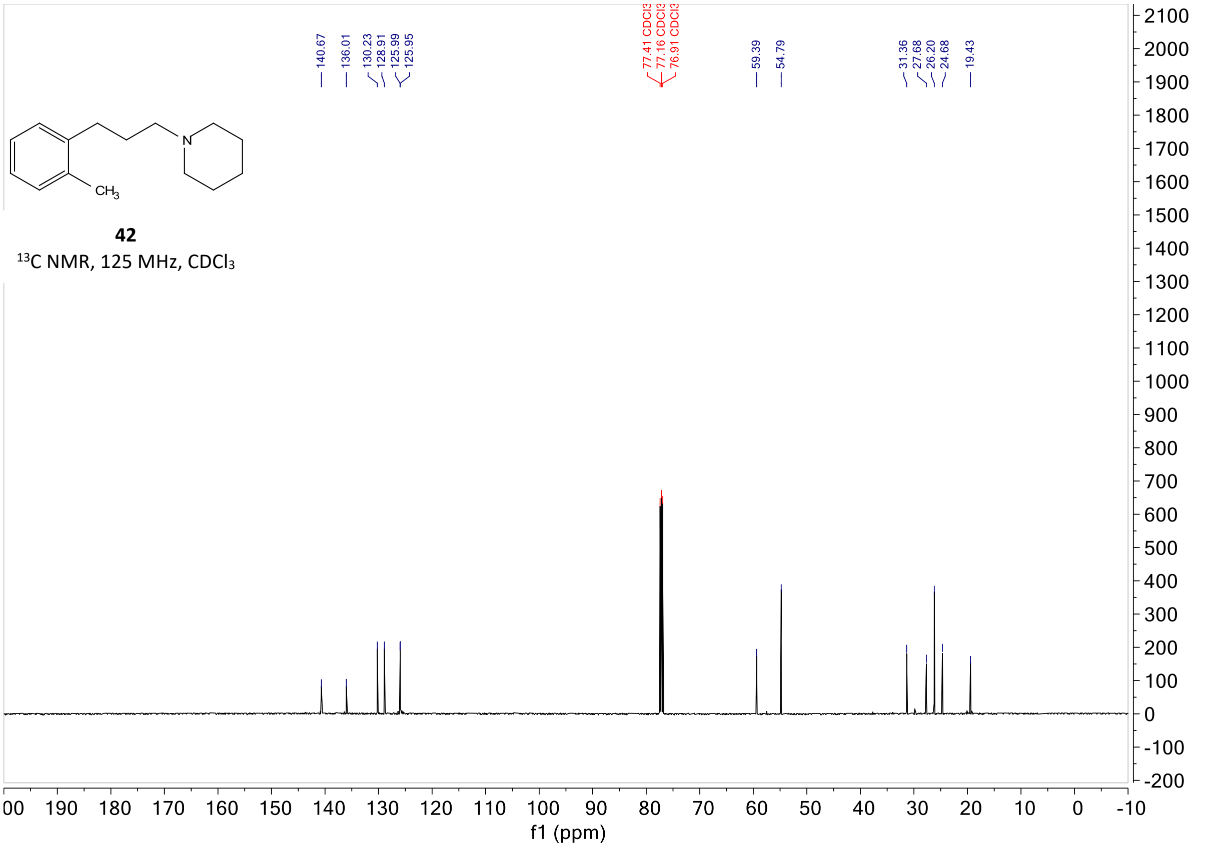

Supplementary Figure 148. ^{13}C NMR spectra of compound **42**

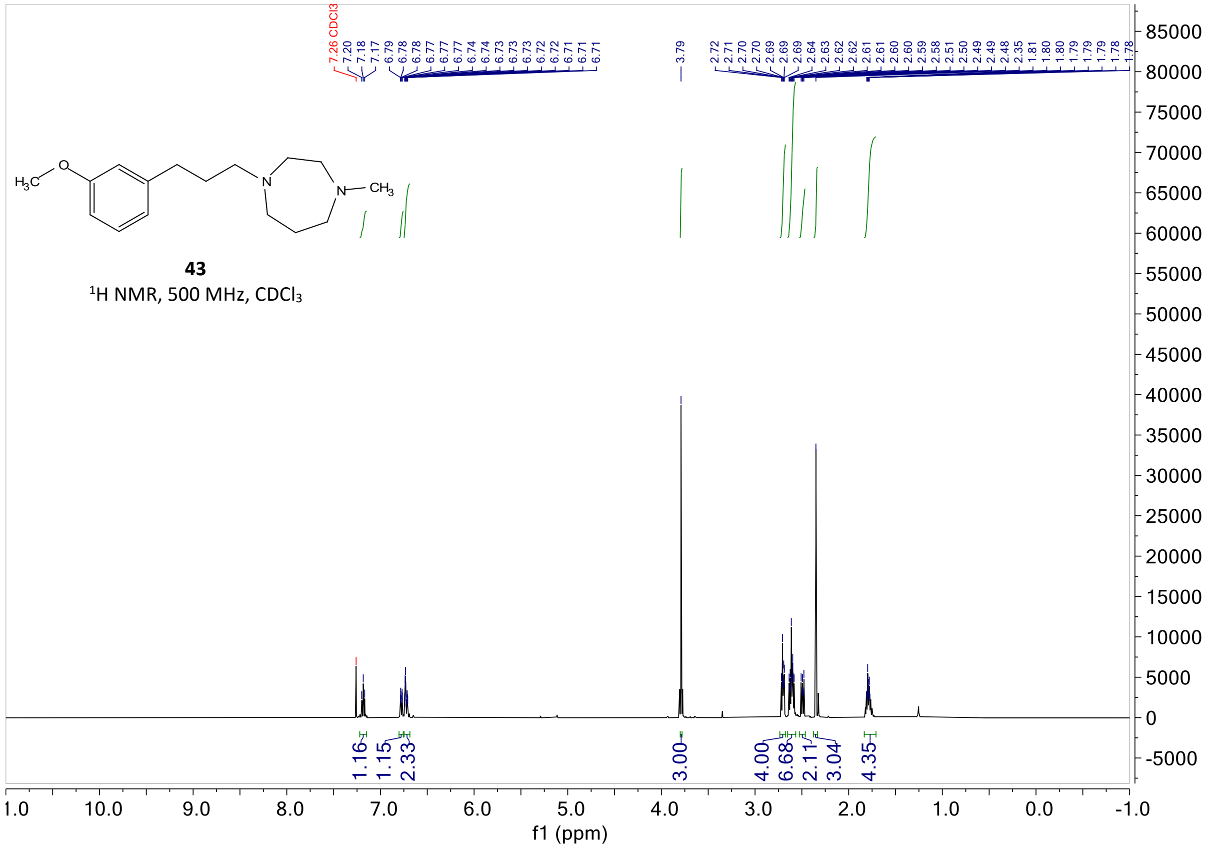

Supplementary Figure 149. ^{1}H NMR spectra of compound **43**

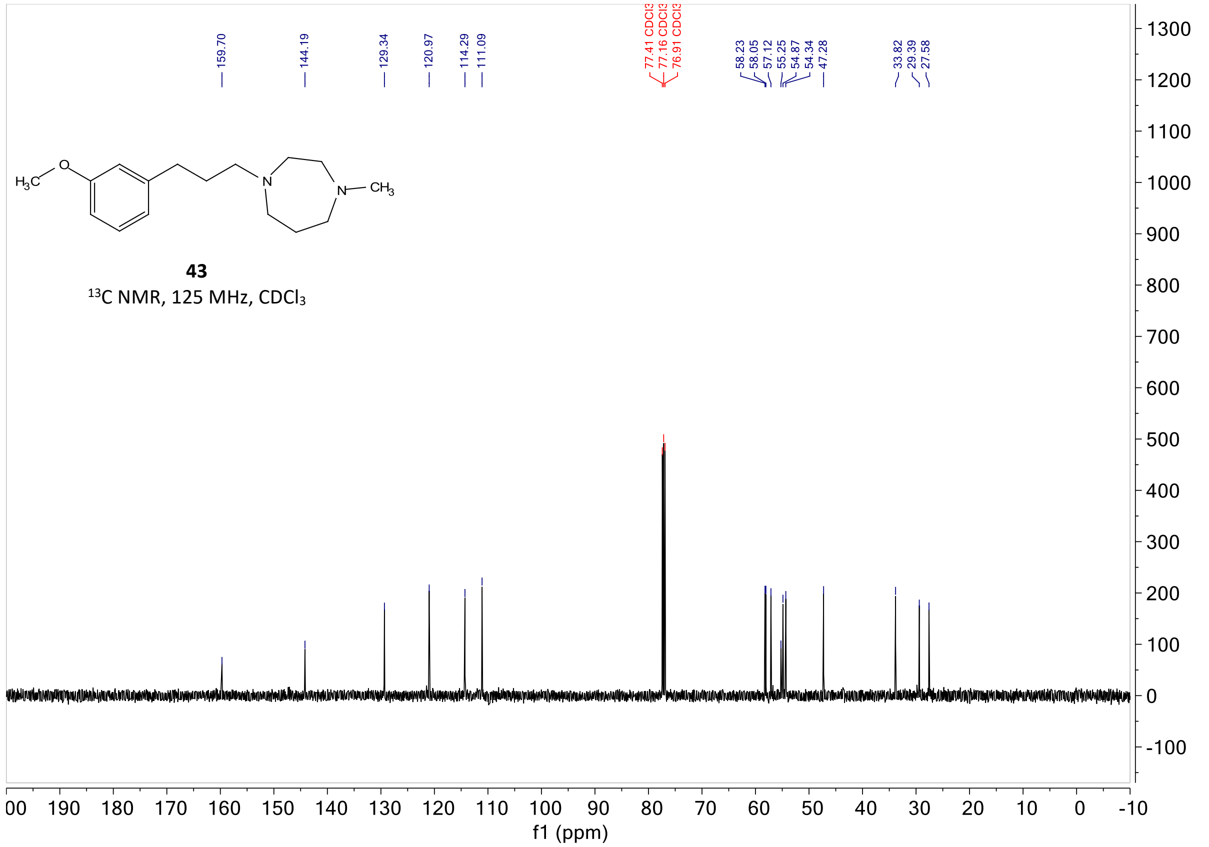

Supplementary Figure 150. ^{13}C NMR spectra of compound **43**

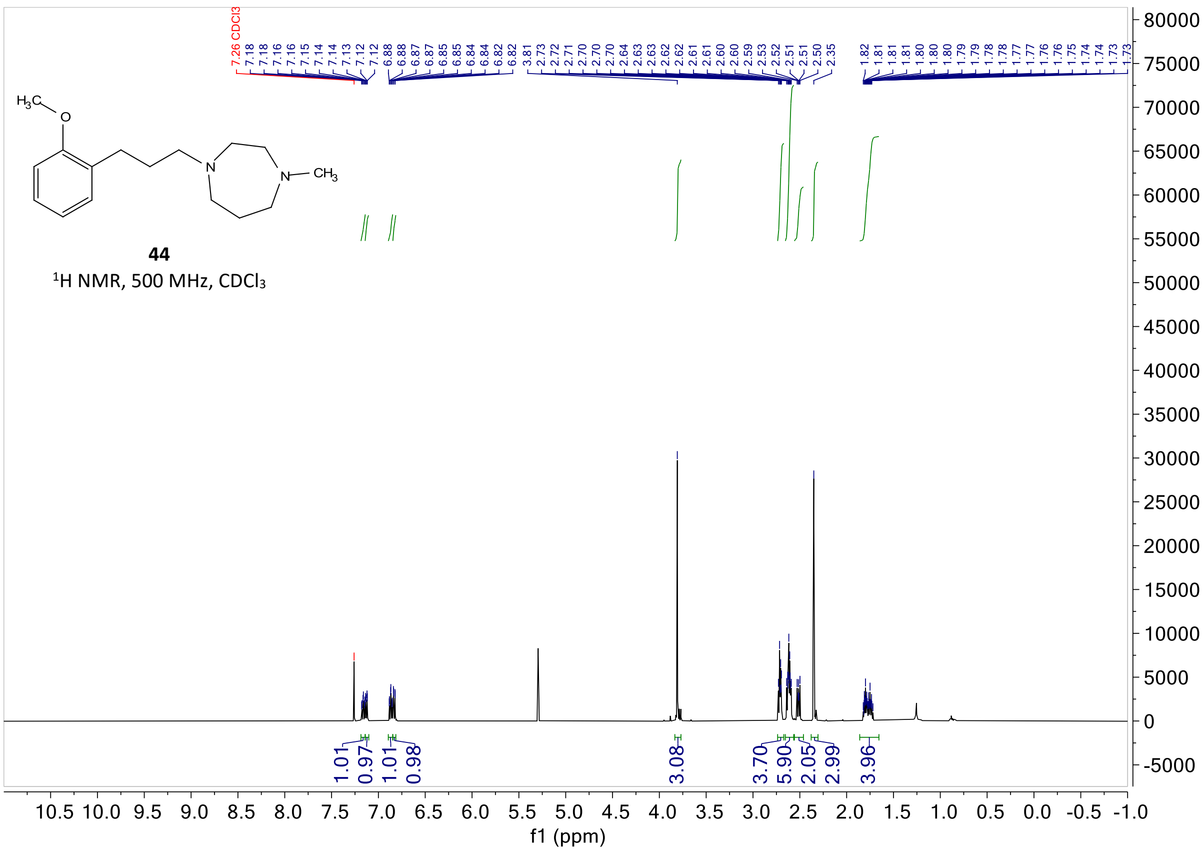

Supplementary Figure 151. ^{1}H NMR spectra of compound **44**

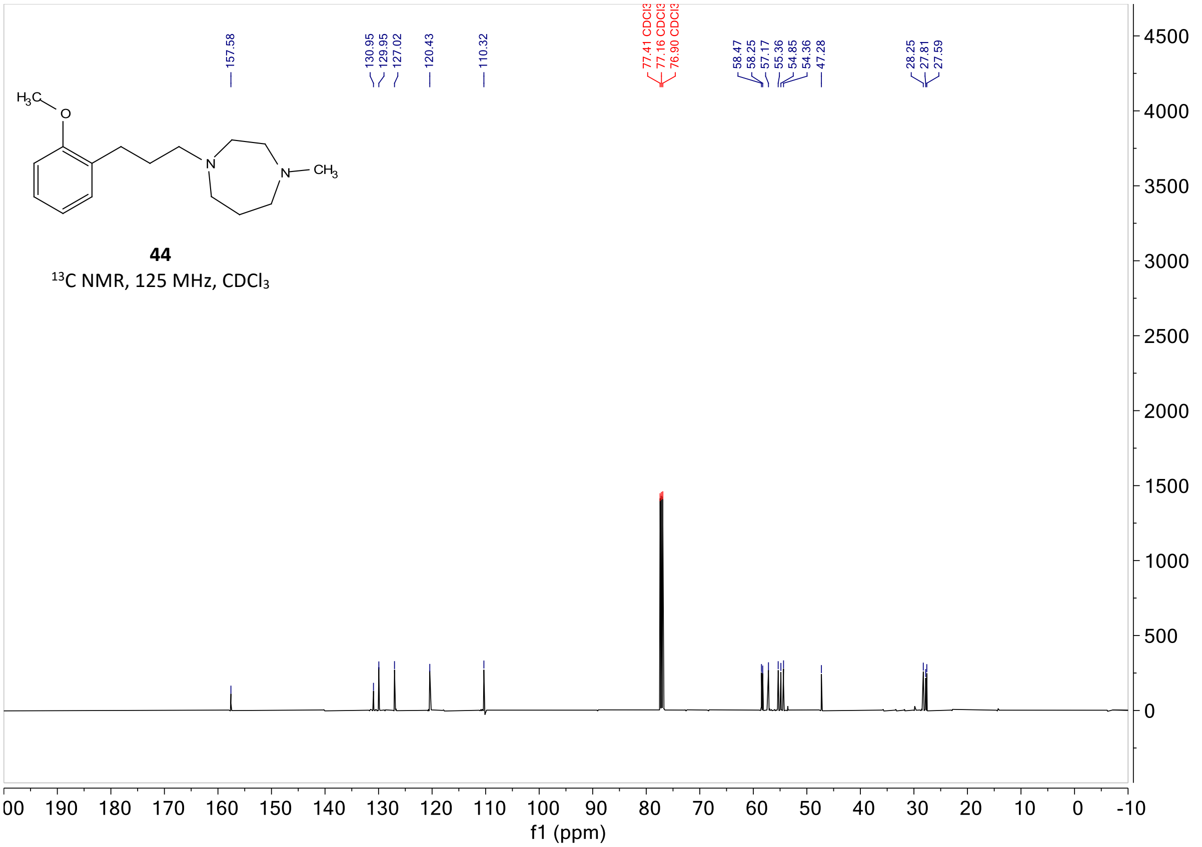

Supplementary Figure 152. ^{13}C NMR spectra of compound **44**

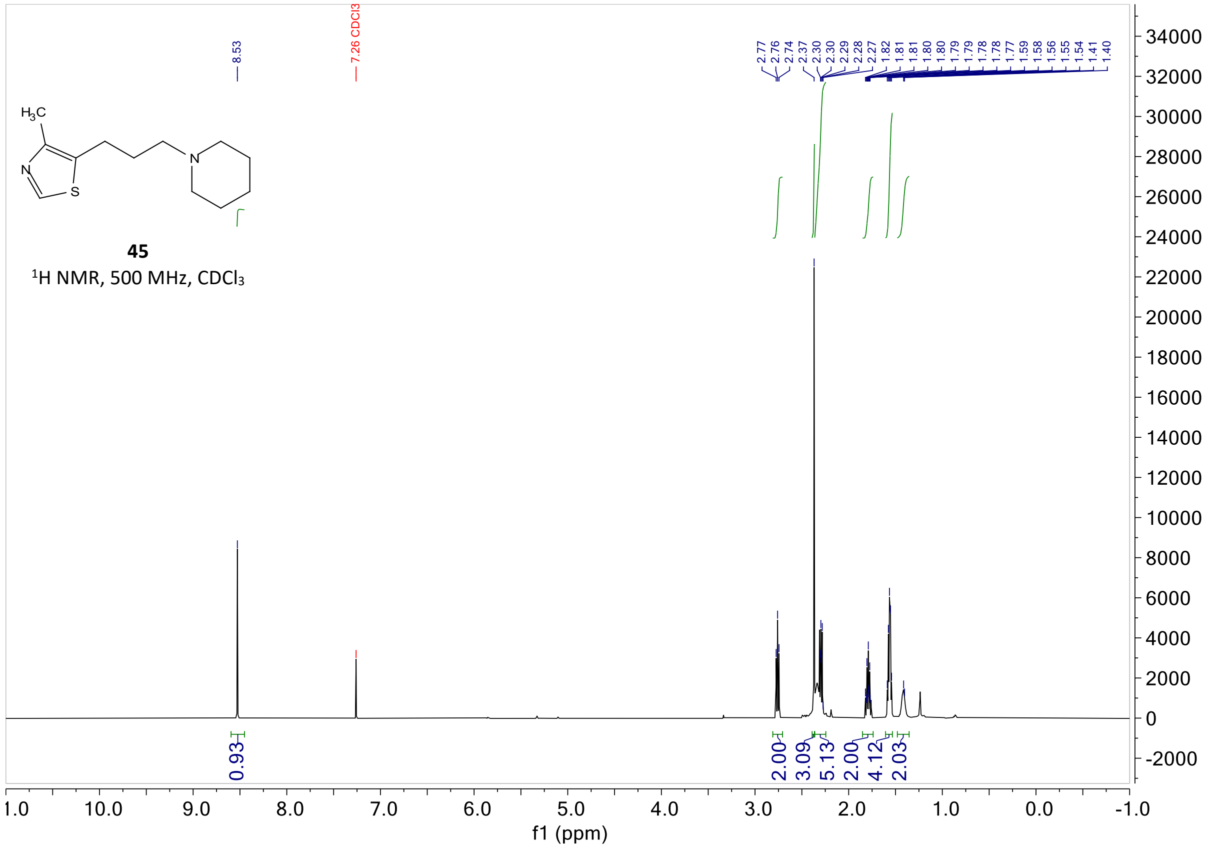

Supplementary Figure 153. ^{1}H NMR spectra of compound **45**

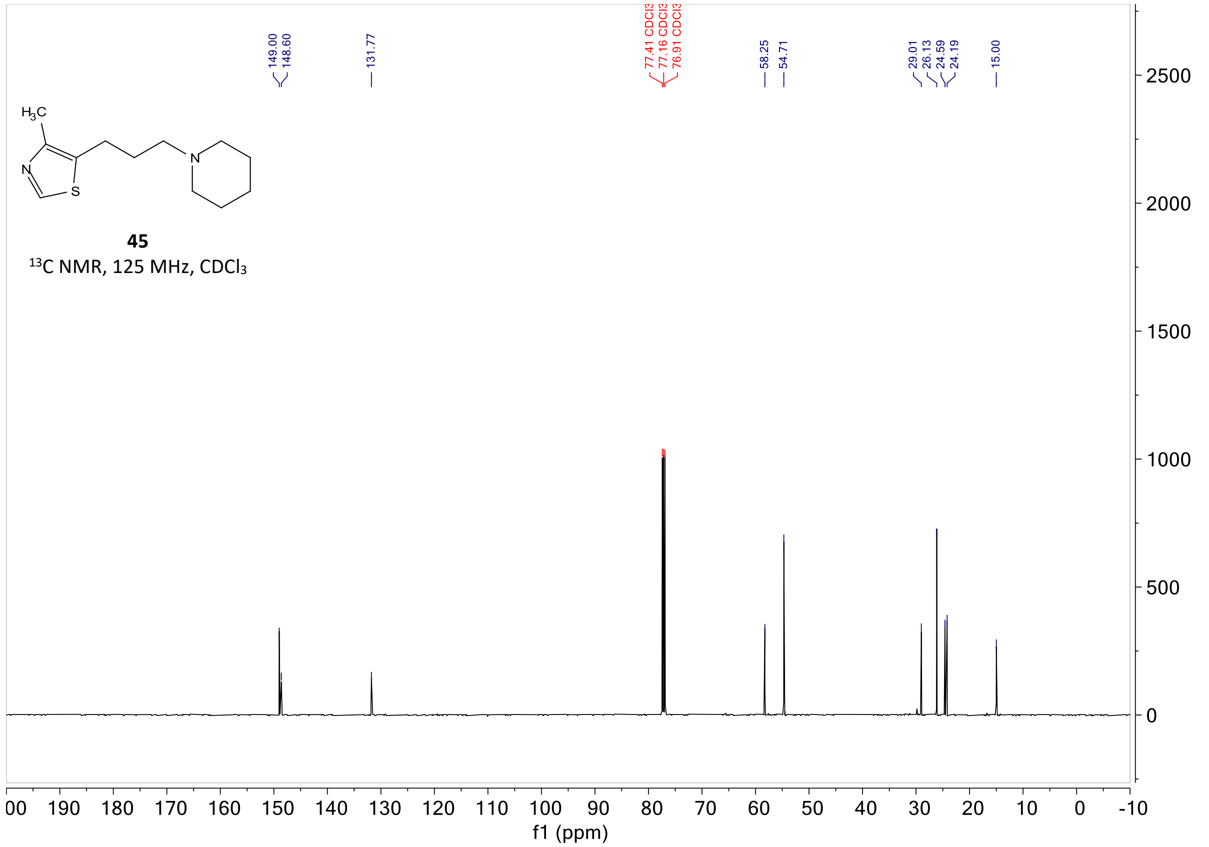

Supplementary Figure 154. ^{13}C NMR spectra of compound **45**

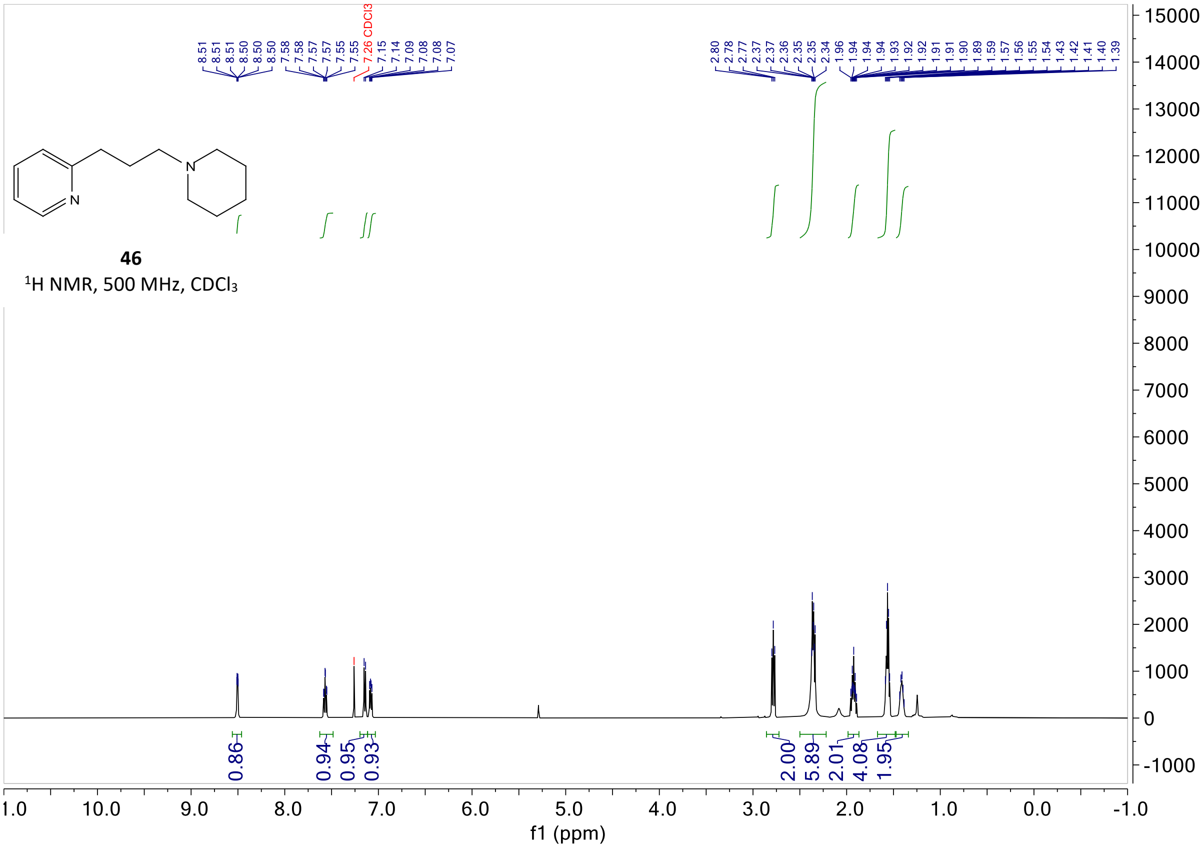

Supplementary Figure 155. ^{1}H NMR spectra of compound **46**

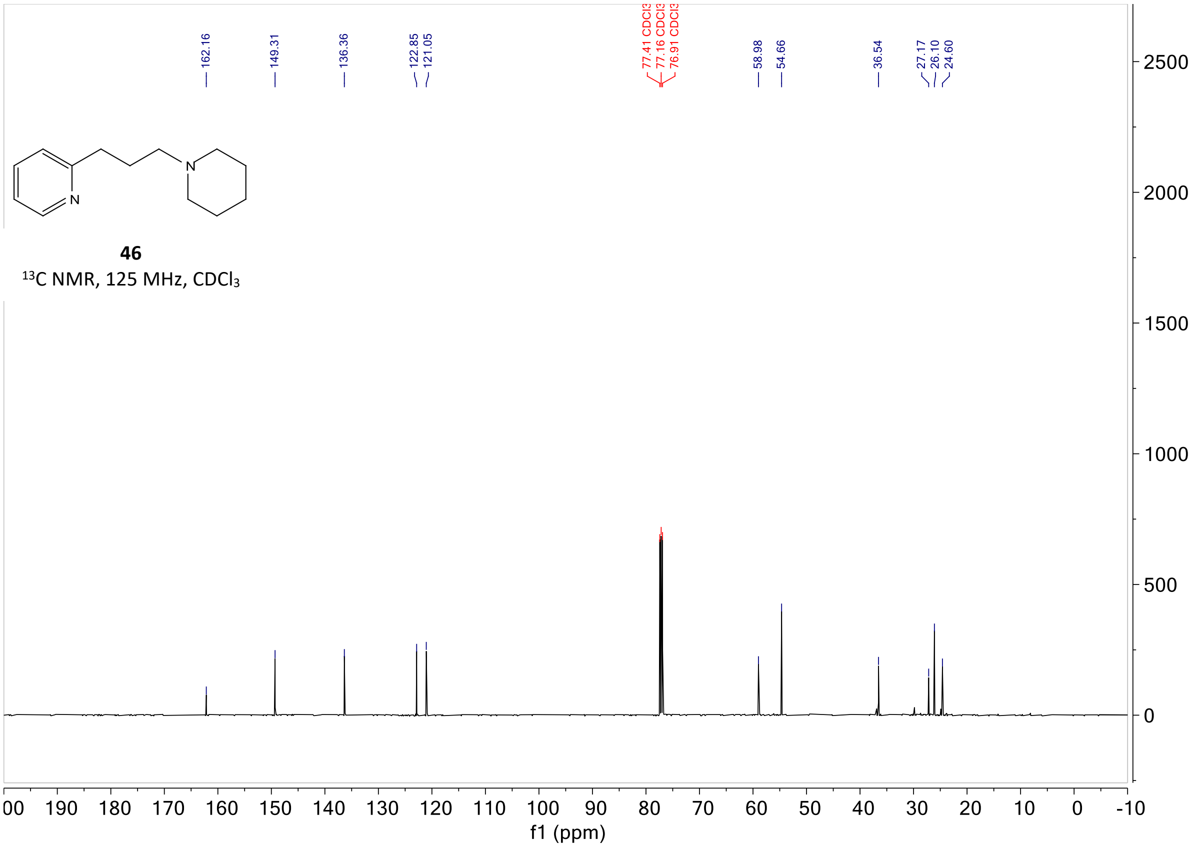

Supplementary Figure 156. ^{13}C NMR spectra of compound **46**

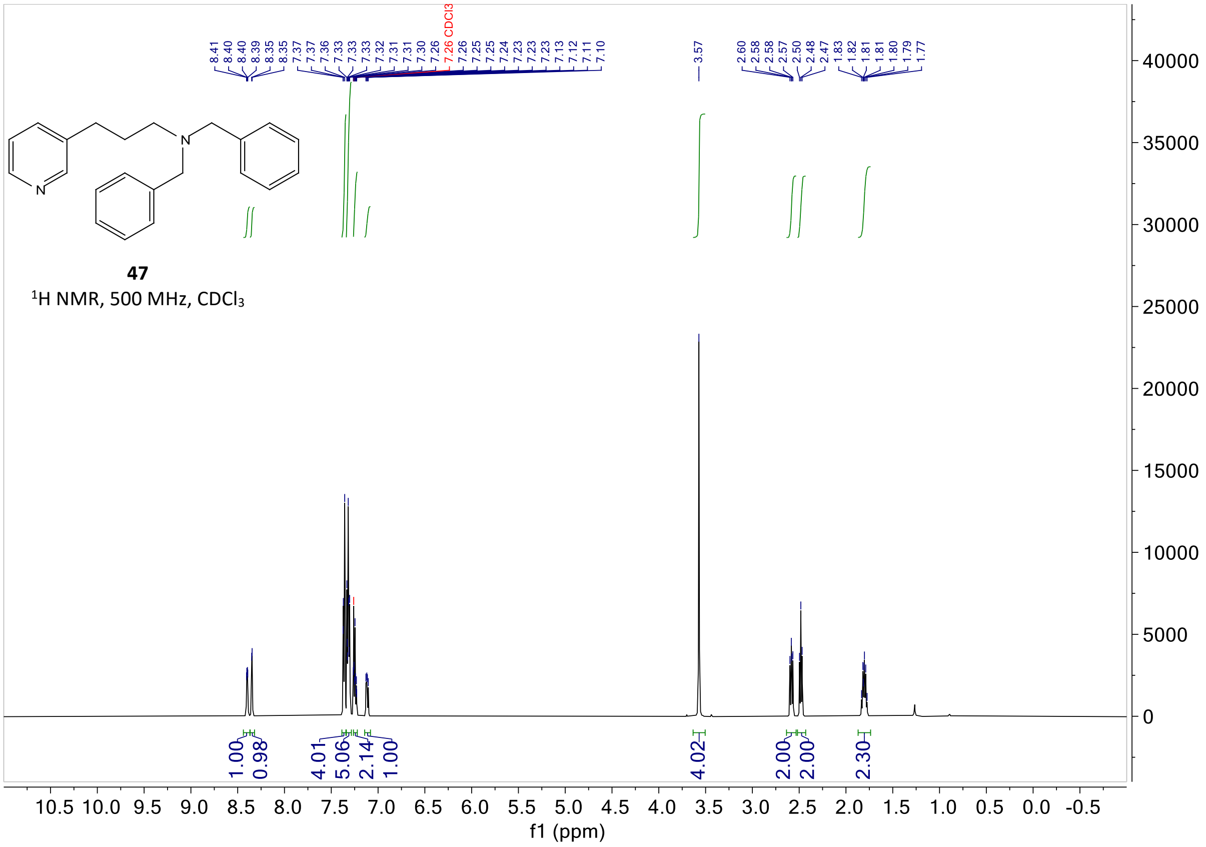

Supplementary Figure 157. ^{1}H NMR spectra of compound **47**

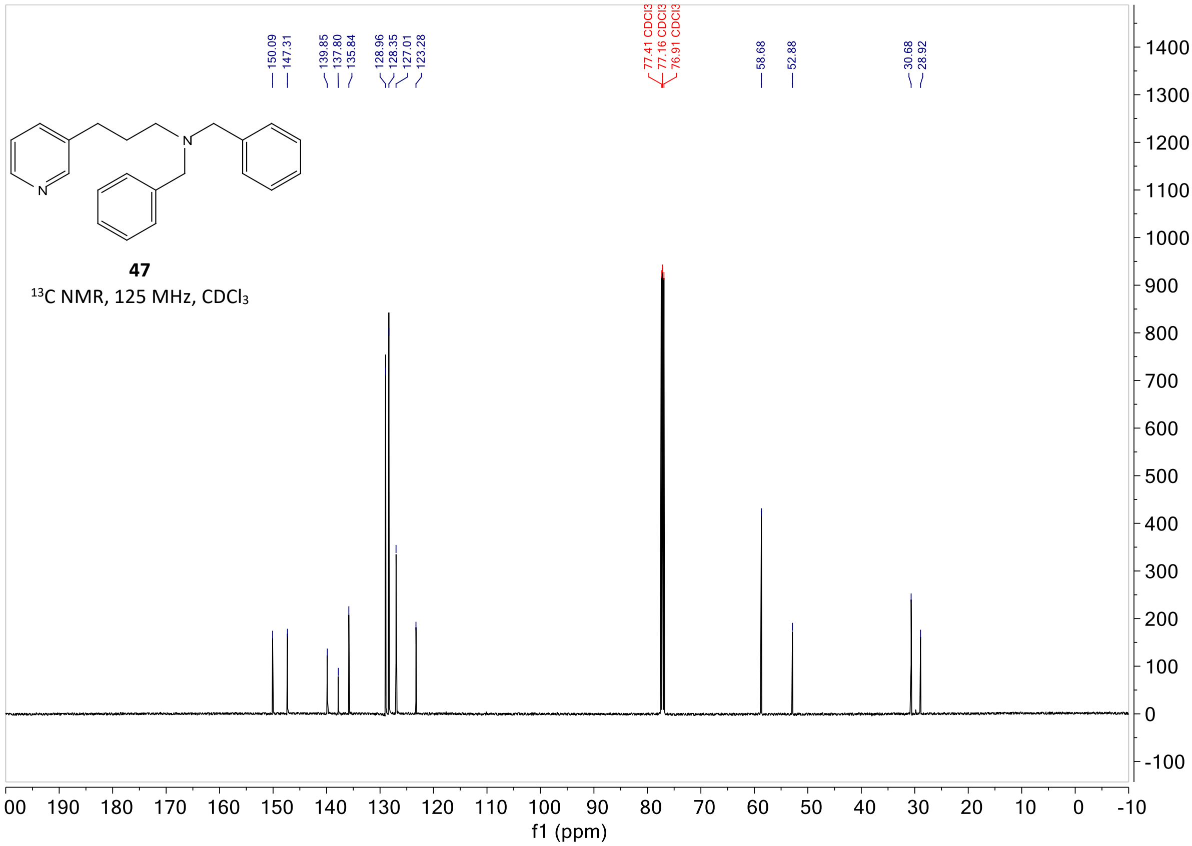

Supplementary Figure 158. ^{13}C NMR spectra of compound **47**

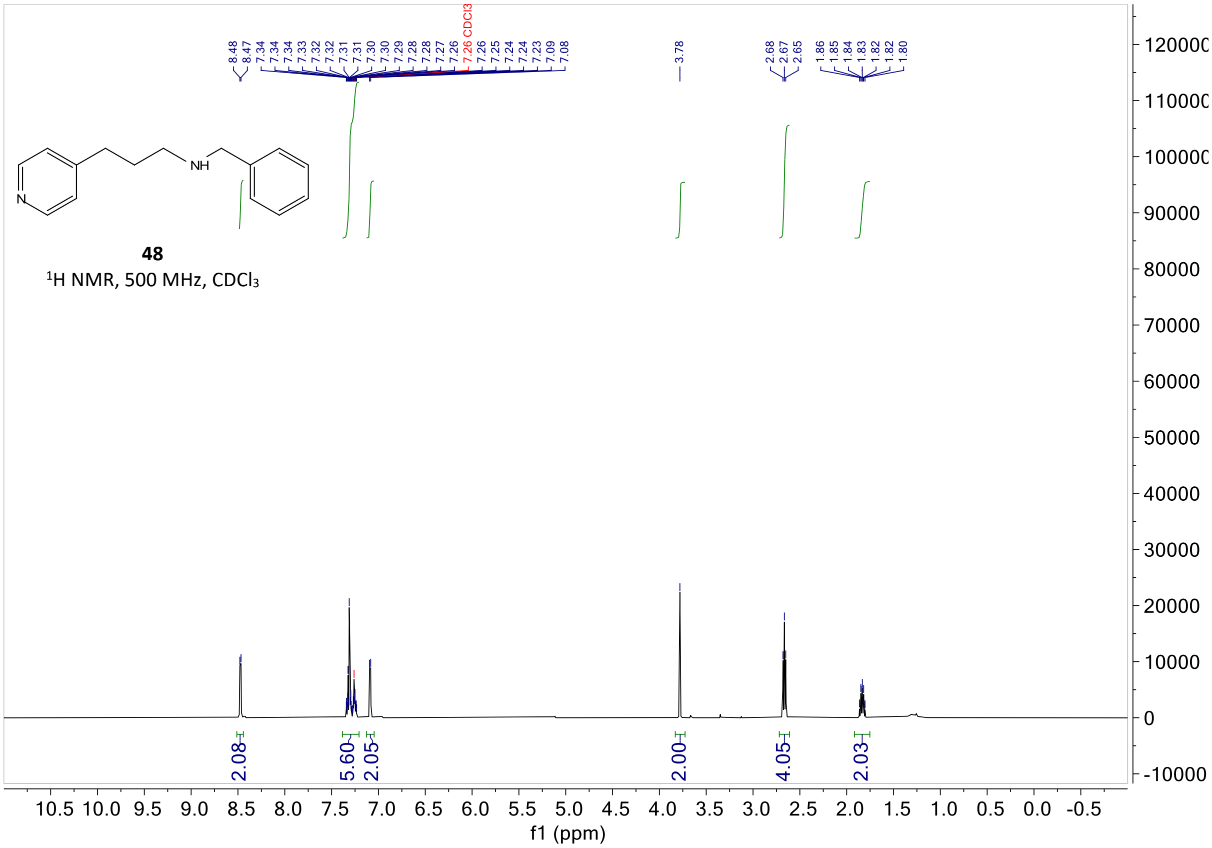

Supplementary Figure 159. ^{1}H NMR spectra of compound **48**

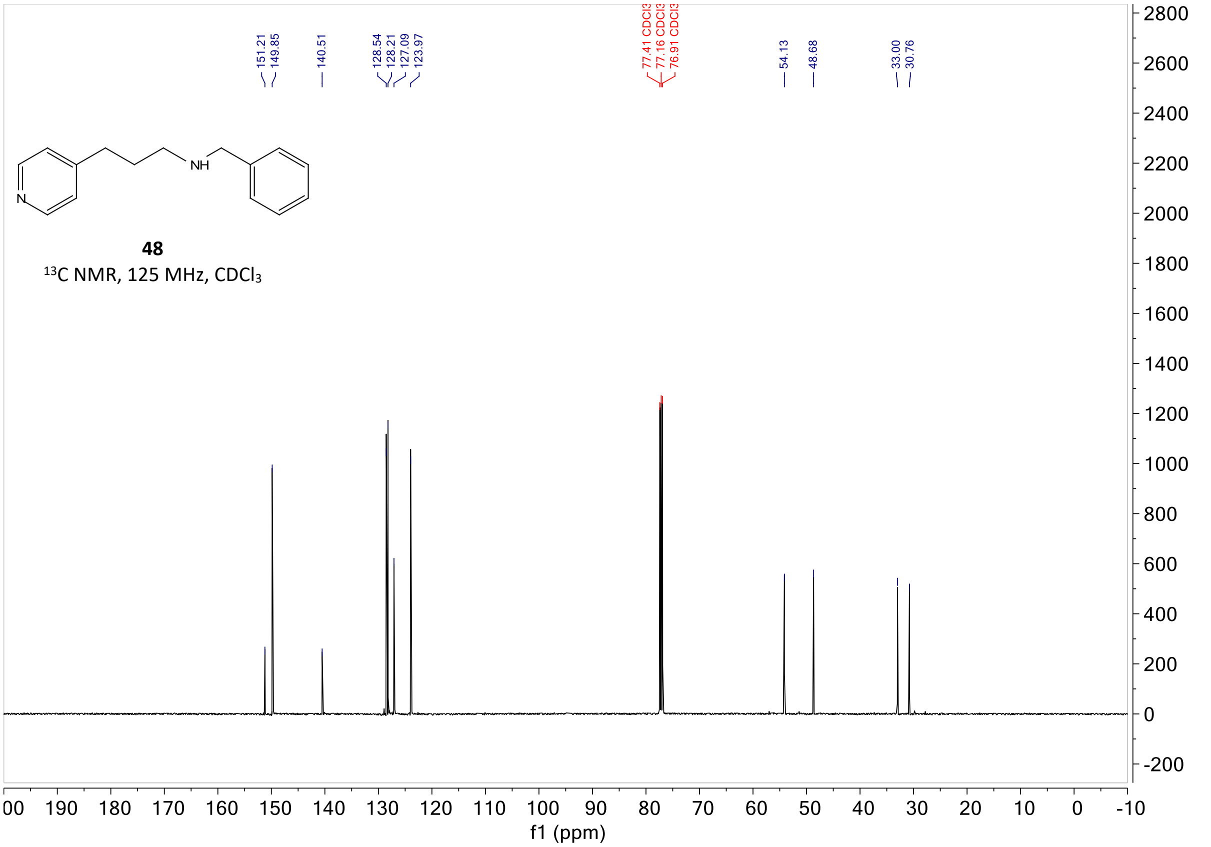

Supplementary Figure 160. ^{13}C NMR spectra of compound **48**

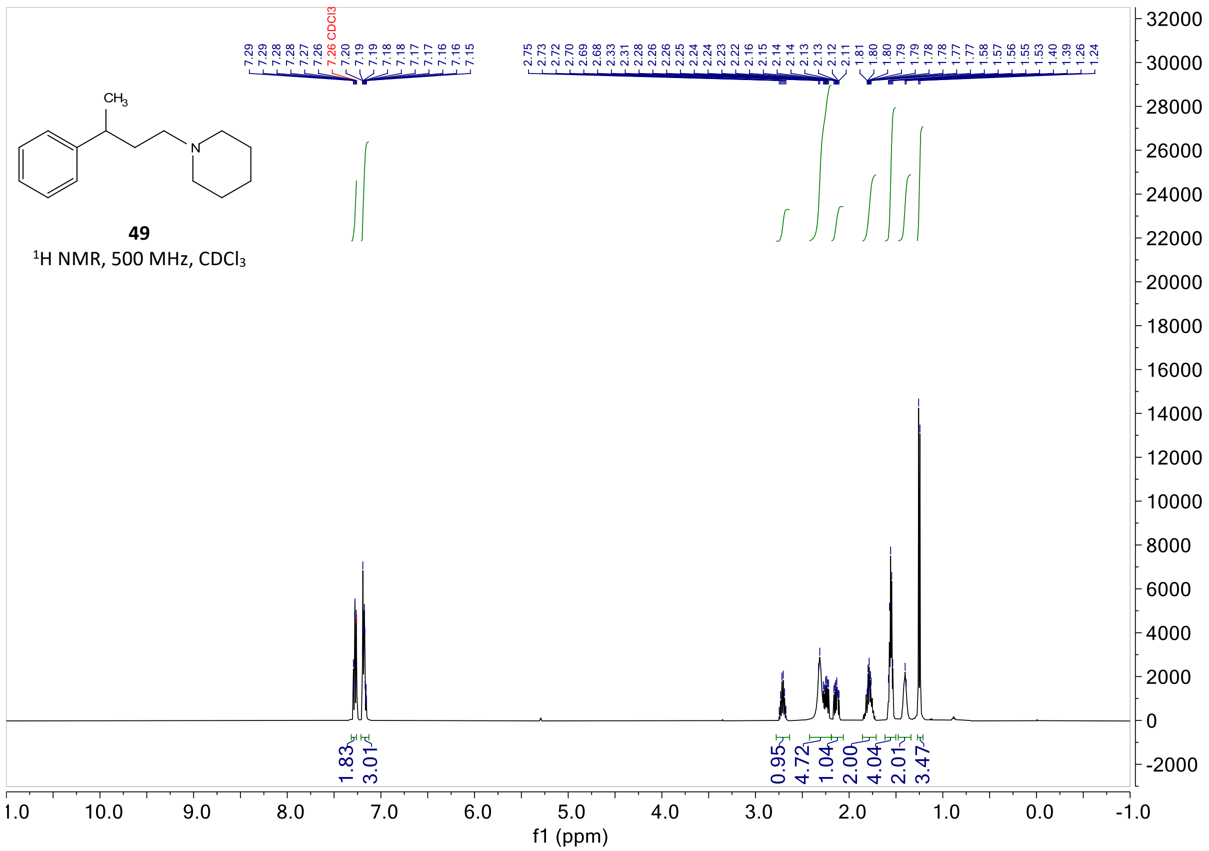

Supplementary Figure 161. ^{1}H NMR spectra of compound **49**

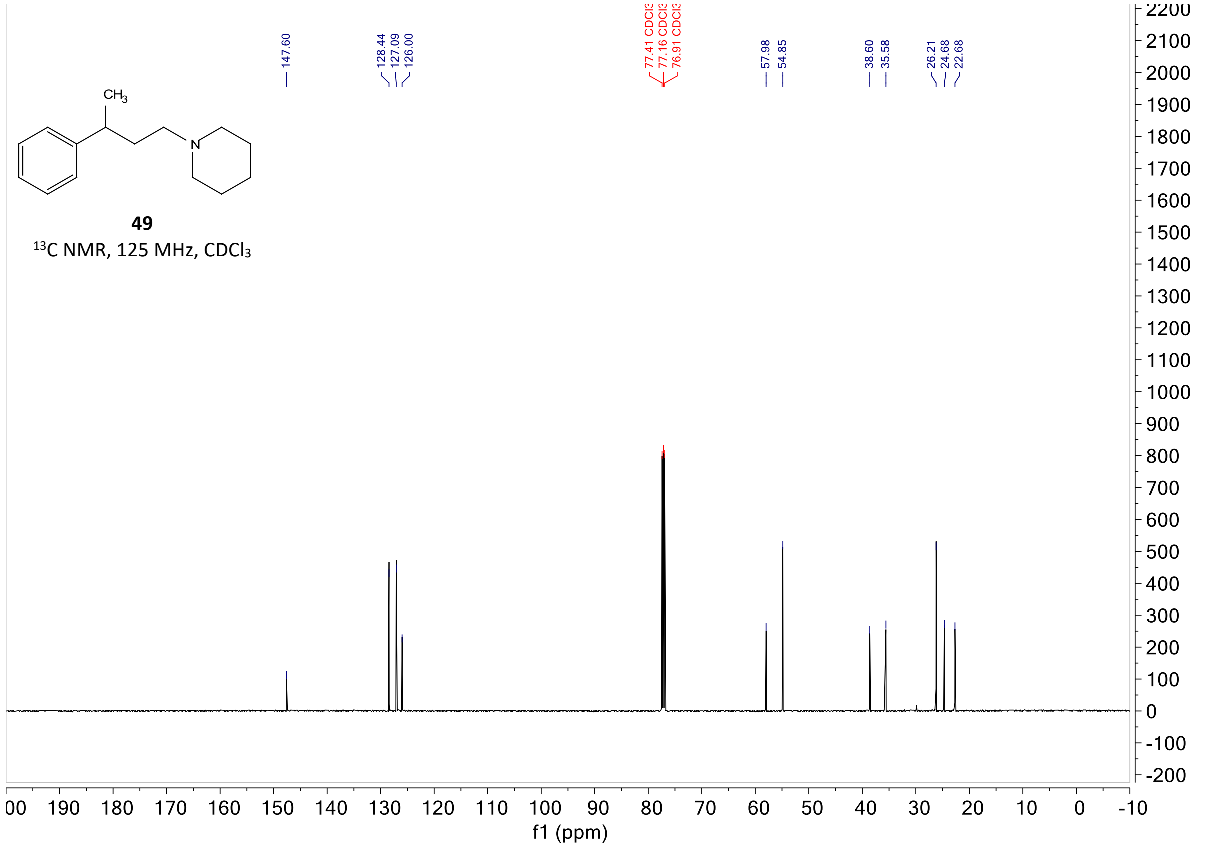

Supplementary Figure 162. ^{13}C NMR spectra of compound **49**

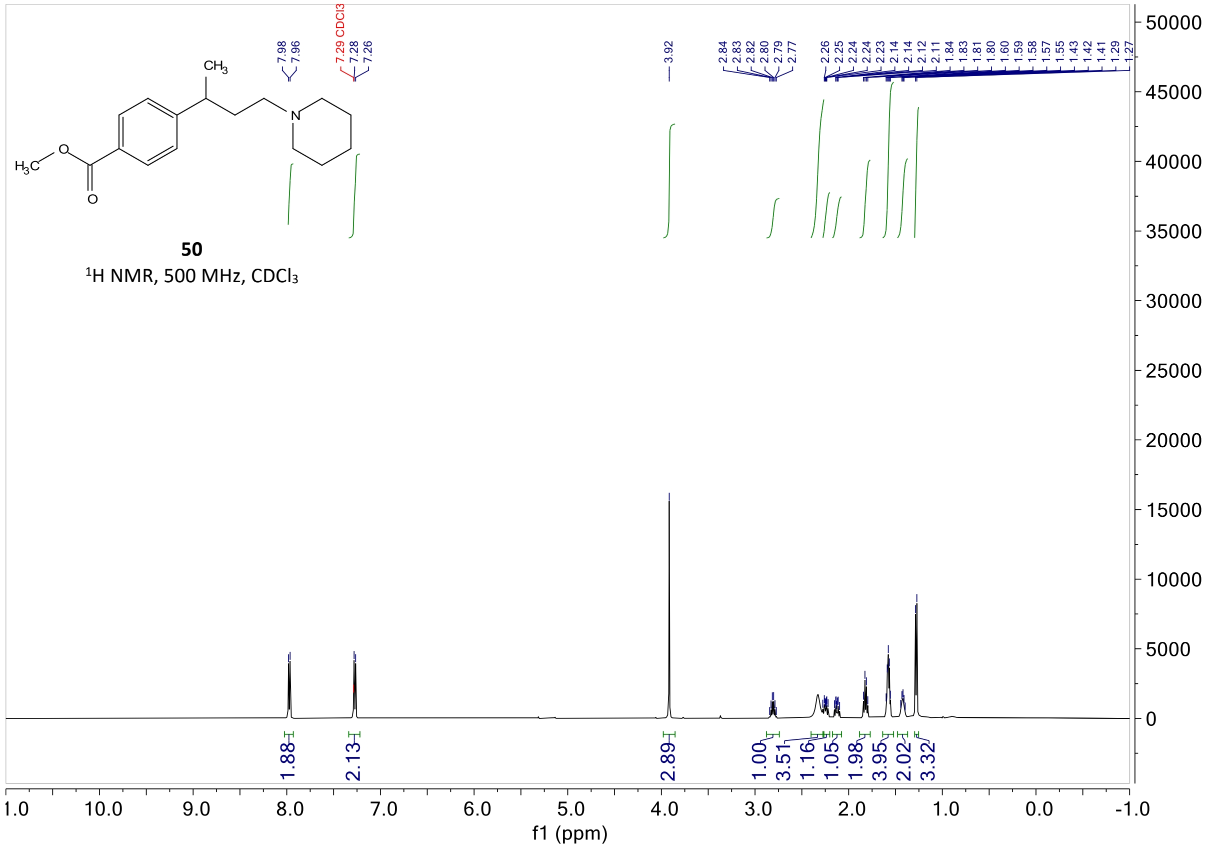

Supplementary Figure 163. ^{1}H NMR spectra of compound **50**

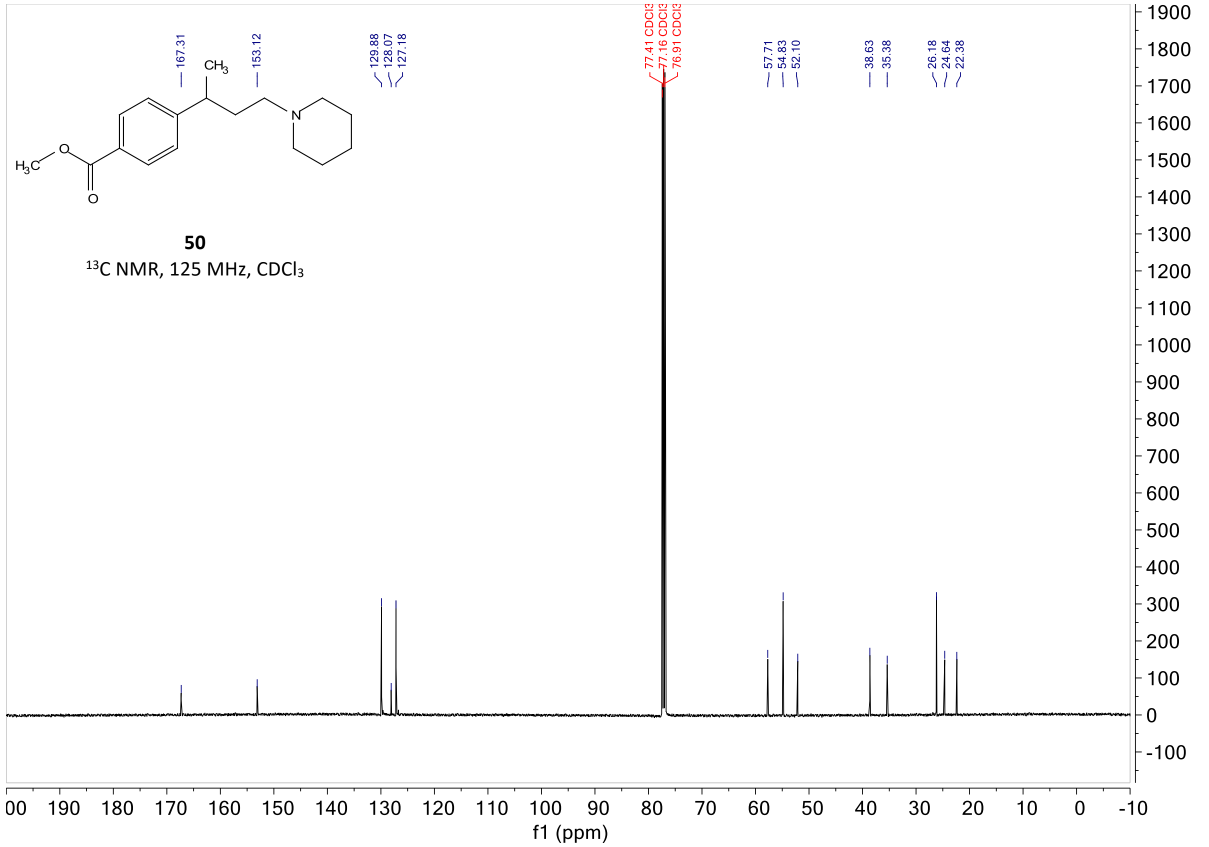

Supplementary Figure 164. ^{13}C NMR spectra of compound **50**

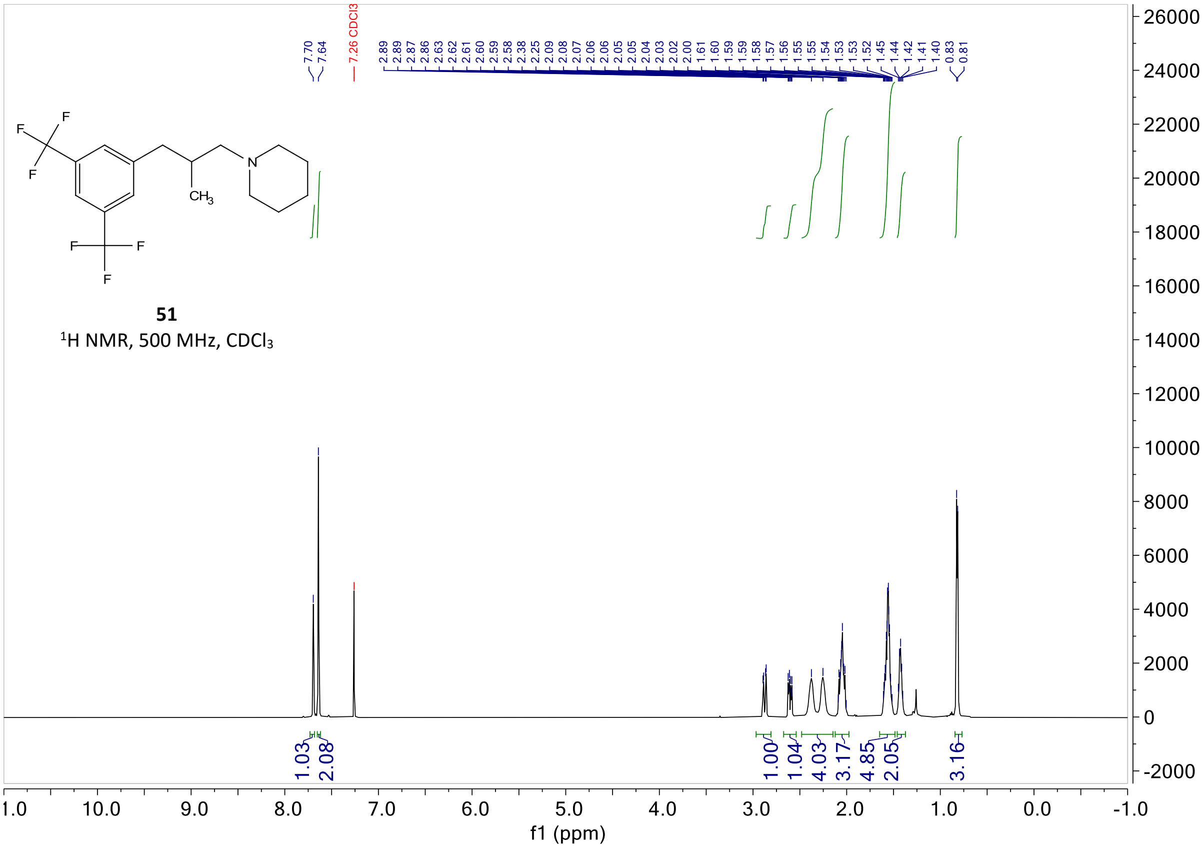

Supplementary Figure 165. ^{1}H NMR spectra of compound **51**

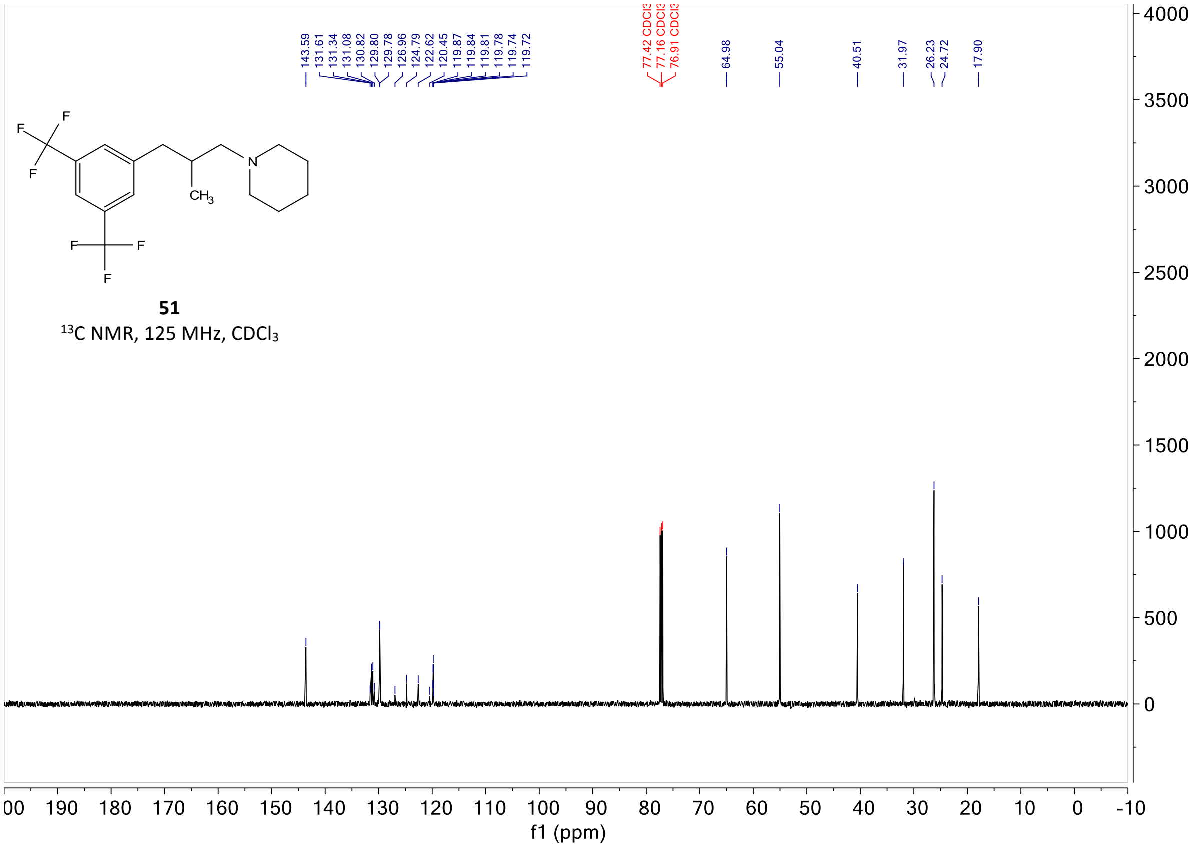

Supplementary Figure 166. ^{13}C NMR spectra of compound **51**

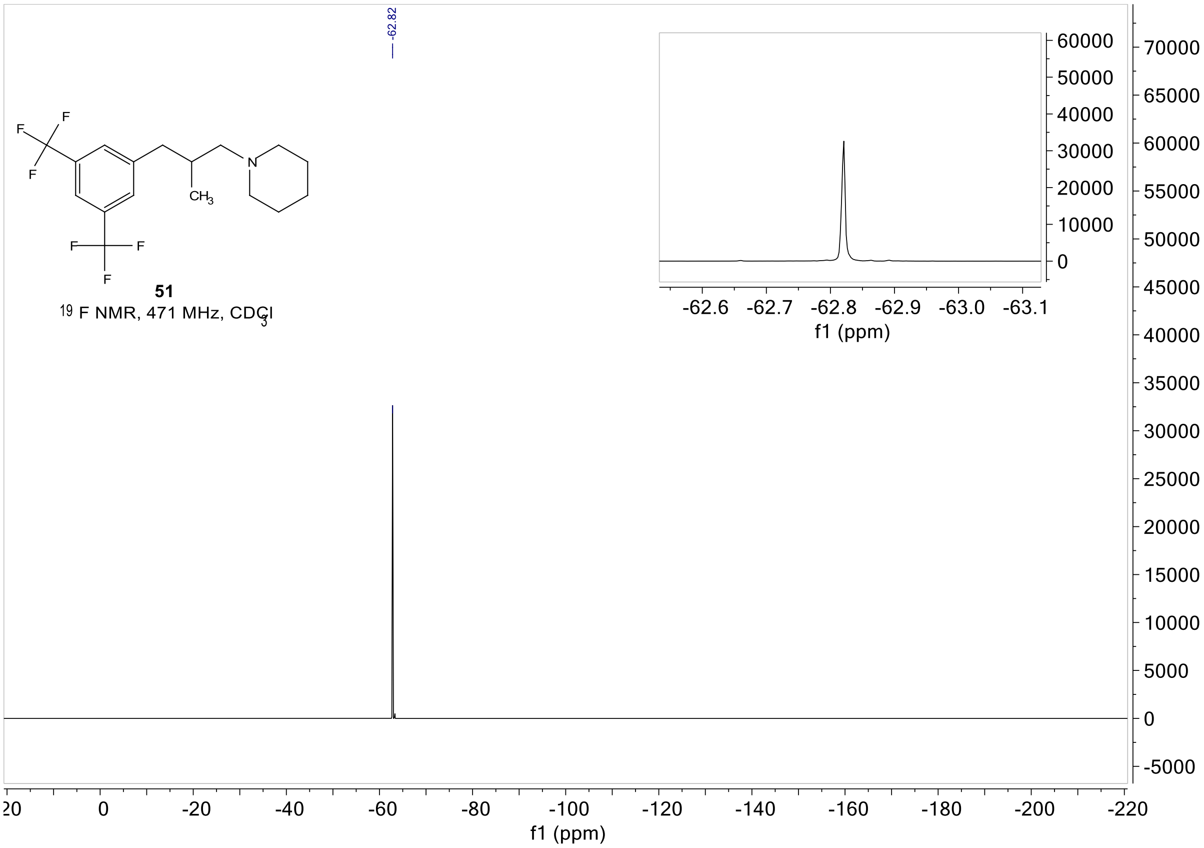

Supplementary Figure 167. ^{19}F NMR spectra of compound **51**

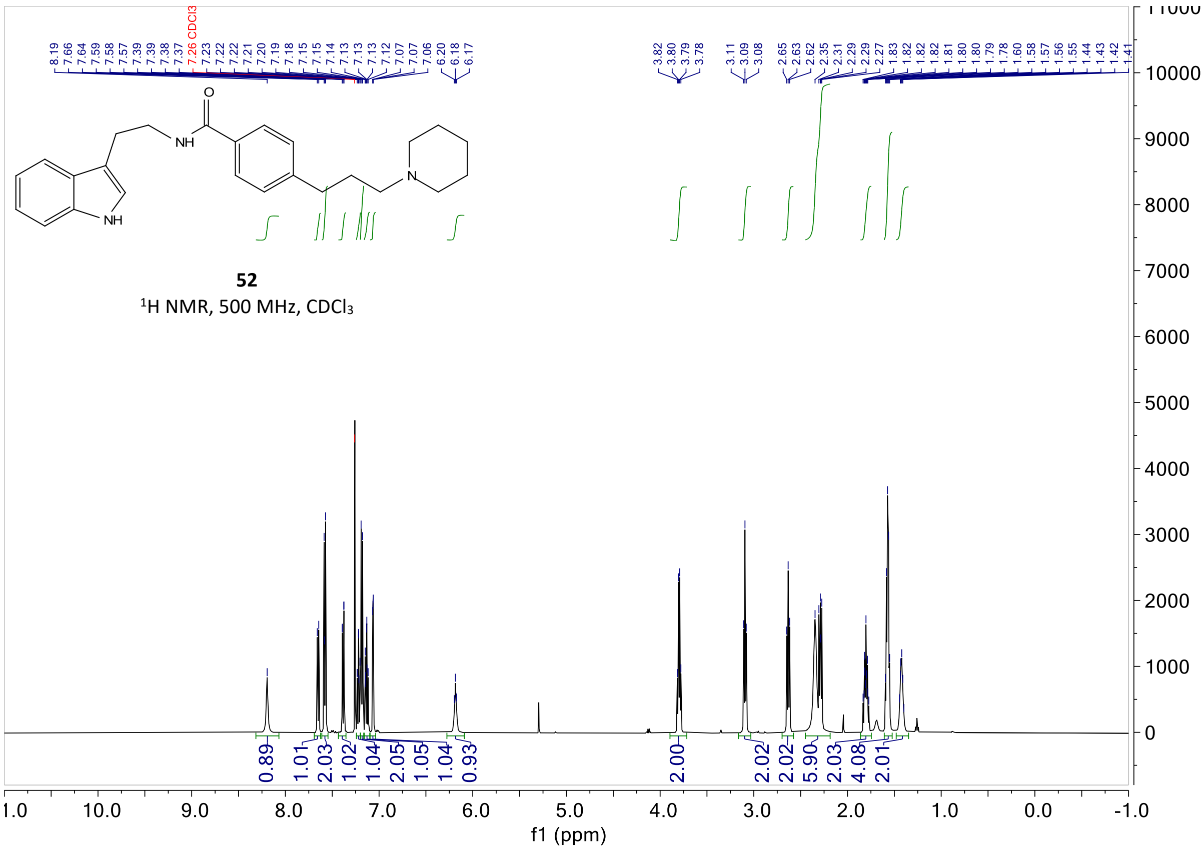

Supplementary Figure 168. ^{1}H NMR spectra of compound **52**

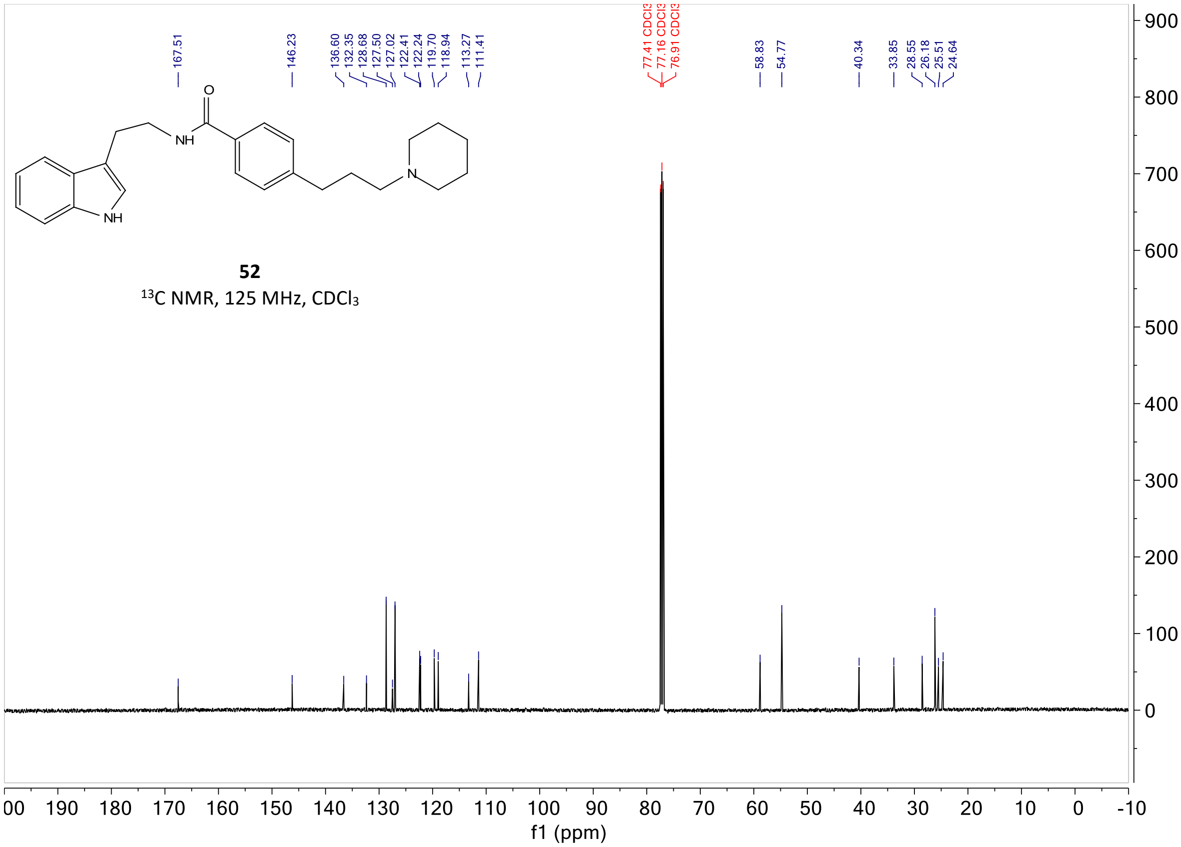

Supplementary Figure 169. ^{13}C NMR spectra of compound **52**

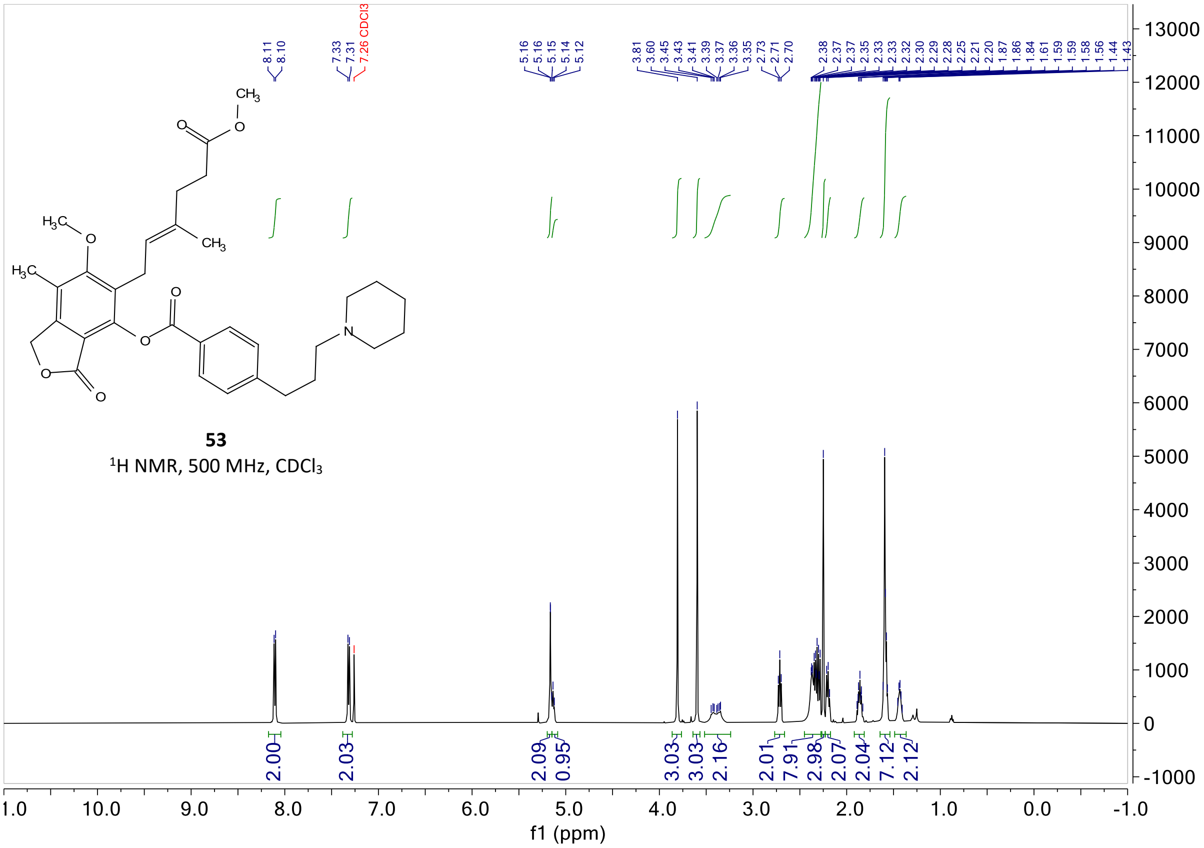

Supplementary Figure 170. ^{1}H NMR spectra of compound **53**

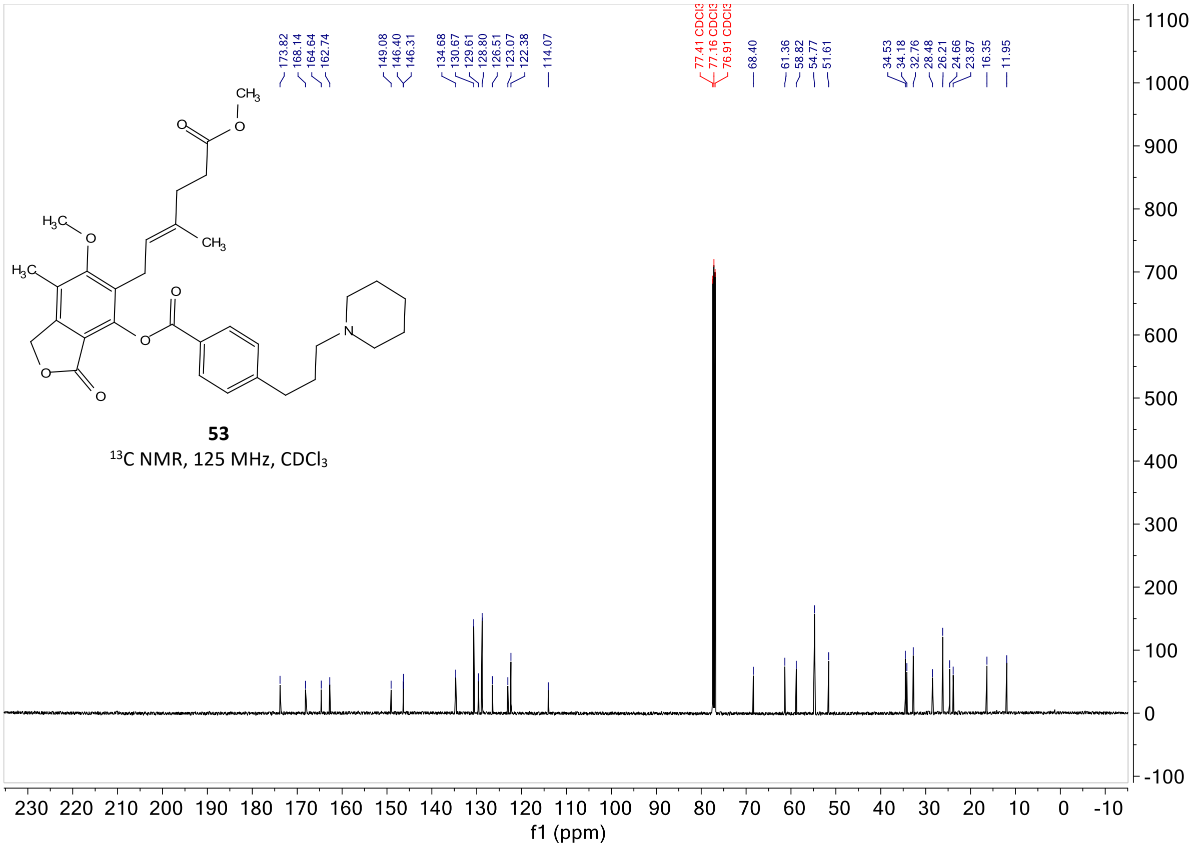

Supplementary Figure 171. ^{13}C NMR spectra of compound **53**

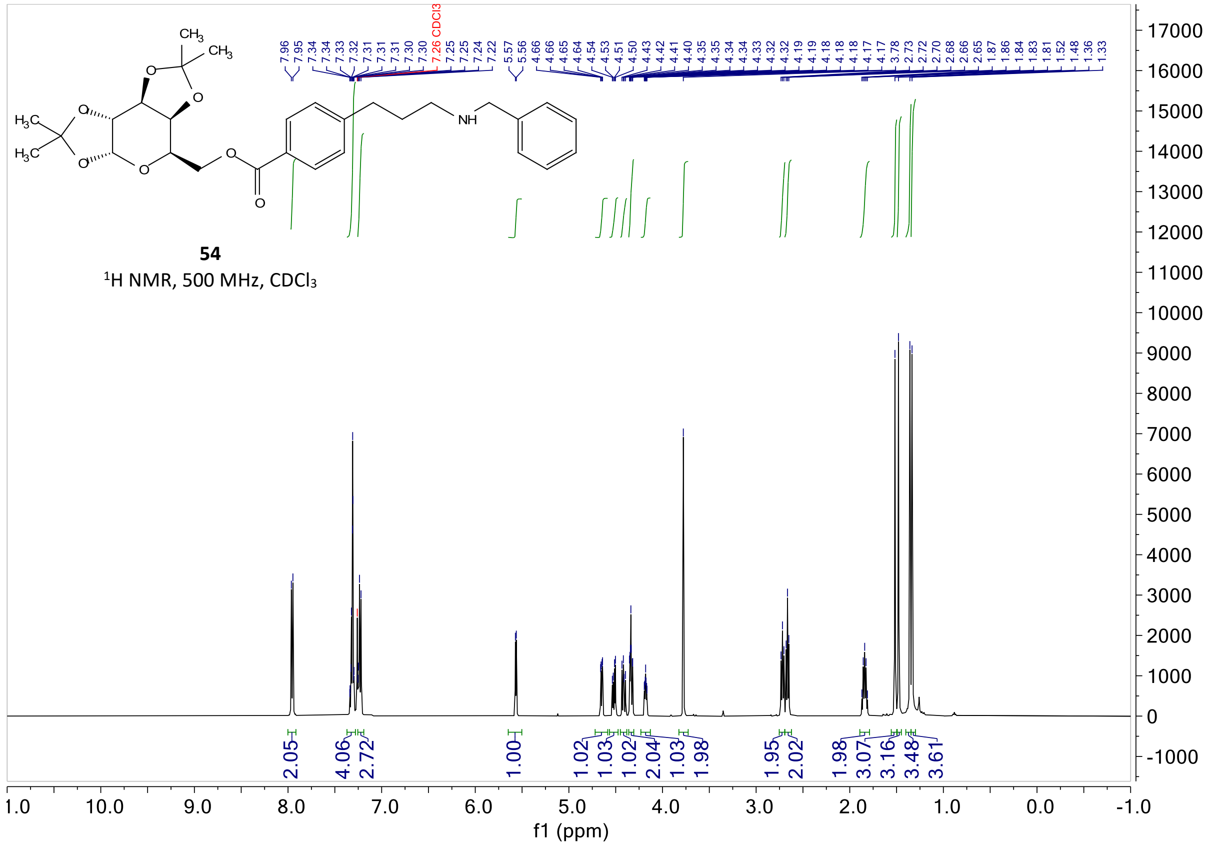

Supplementary Figure 172. ^{1}H NMR spectra of compound **54**

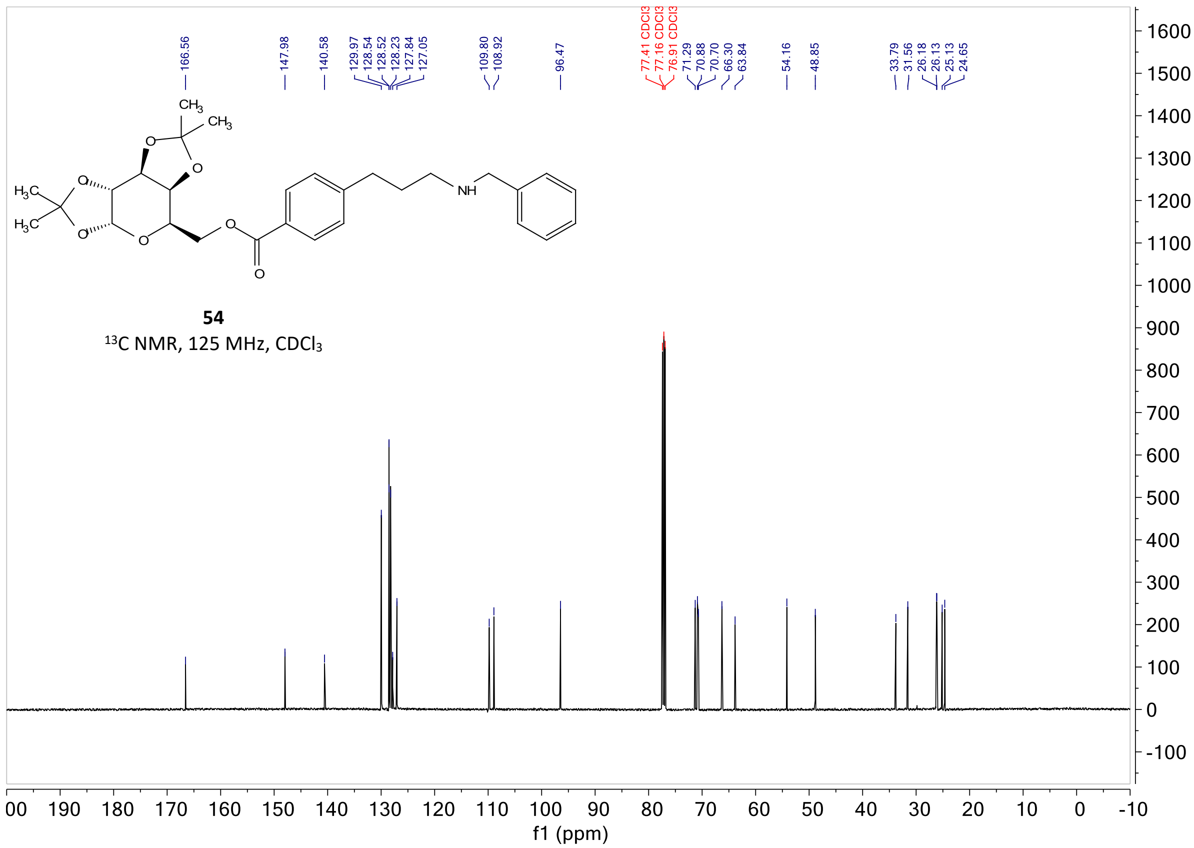

Supplementary Figure 173. ^{13}C NMR spectra of compound **54**

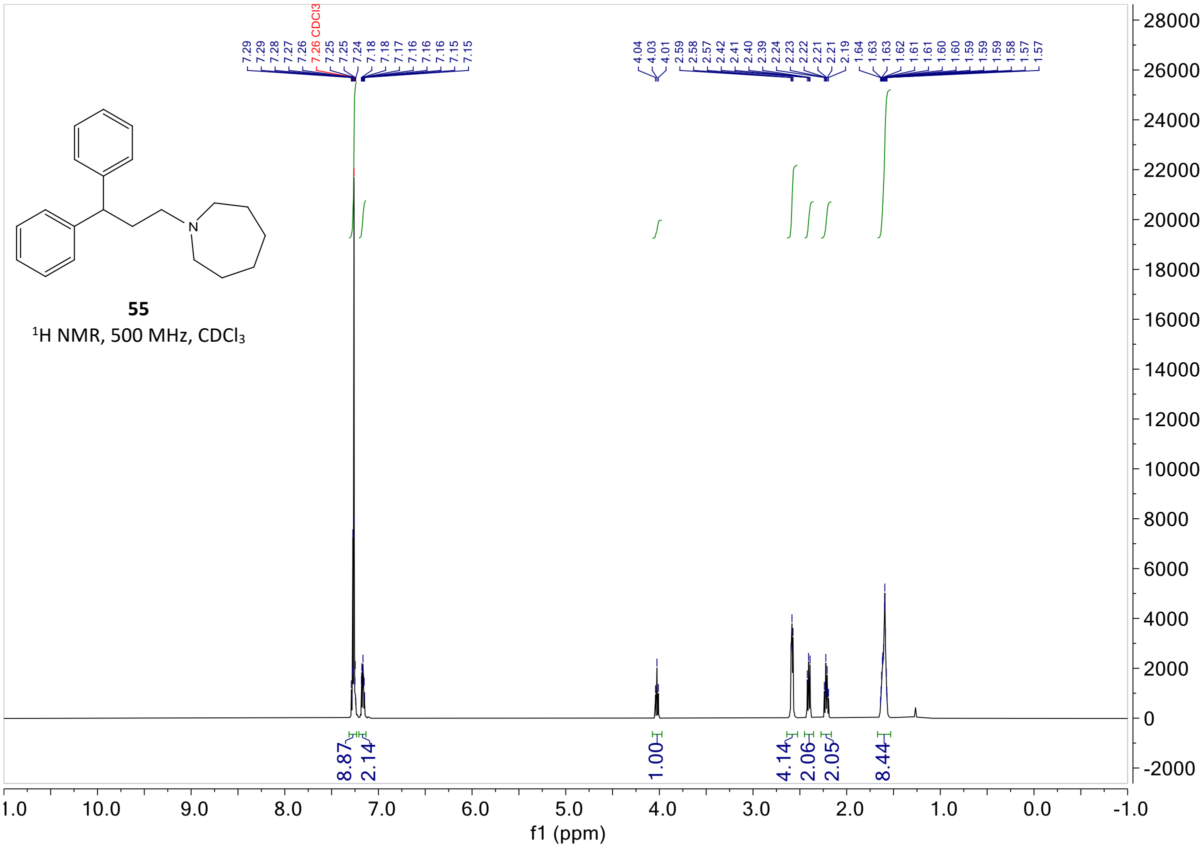

Supplementary Figure 174. ^{1}H NMR spectra of compound **55**

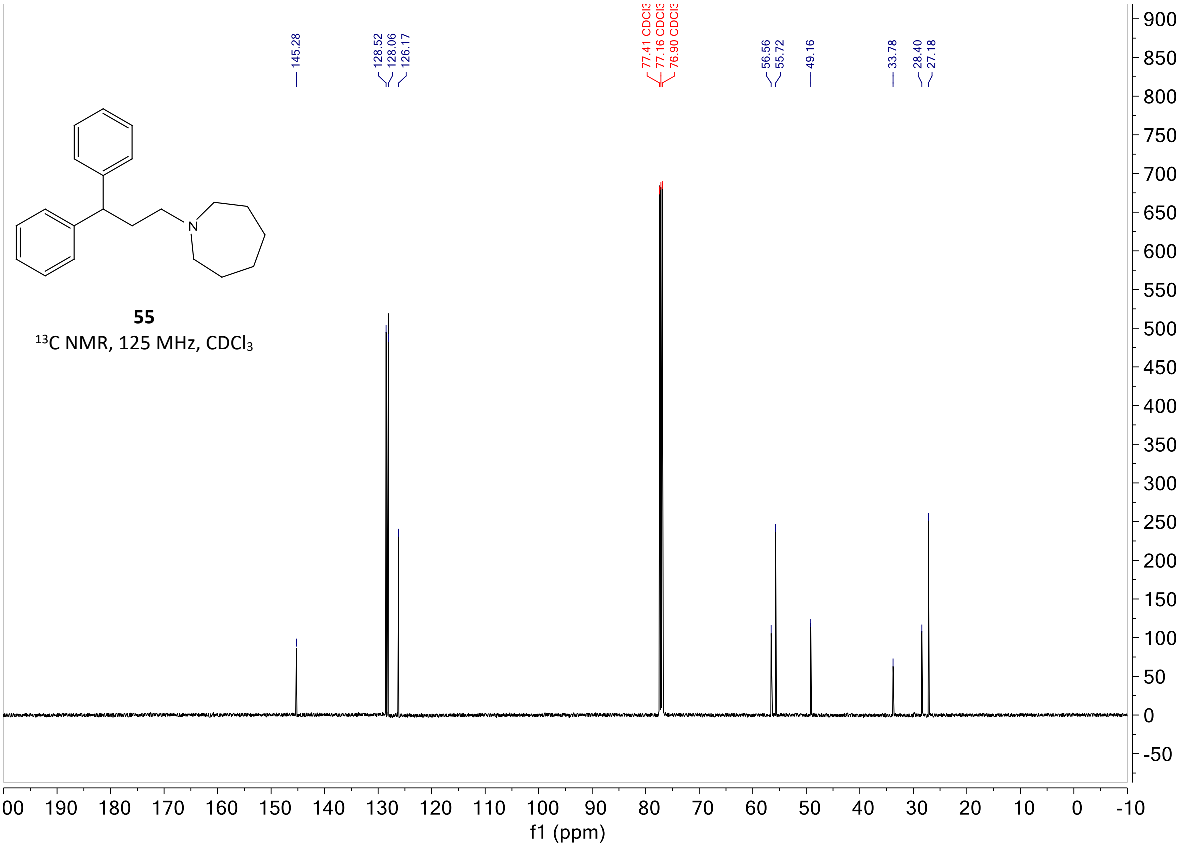

Supplementary Figure 175. ^{13}C NMR spectra of compound **55**

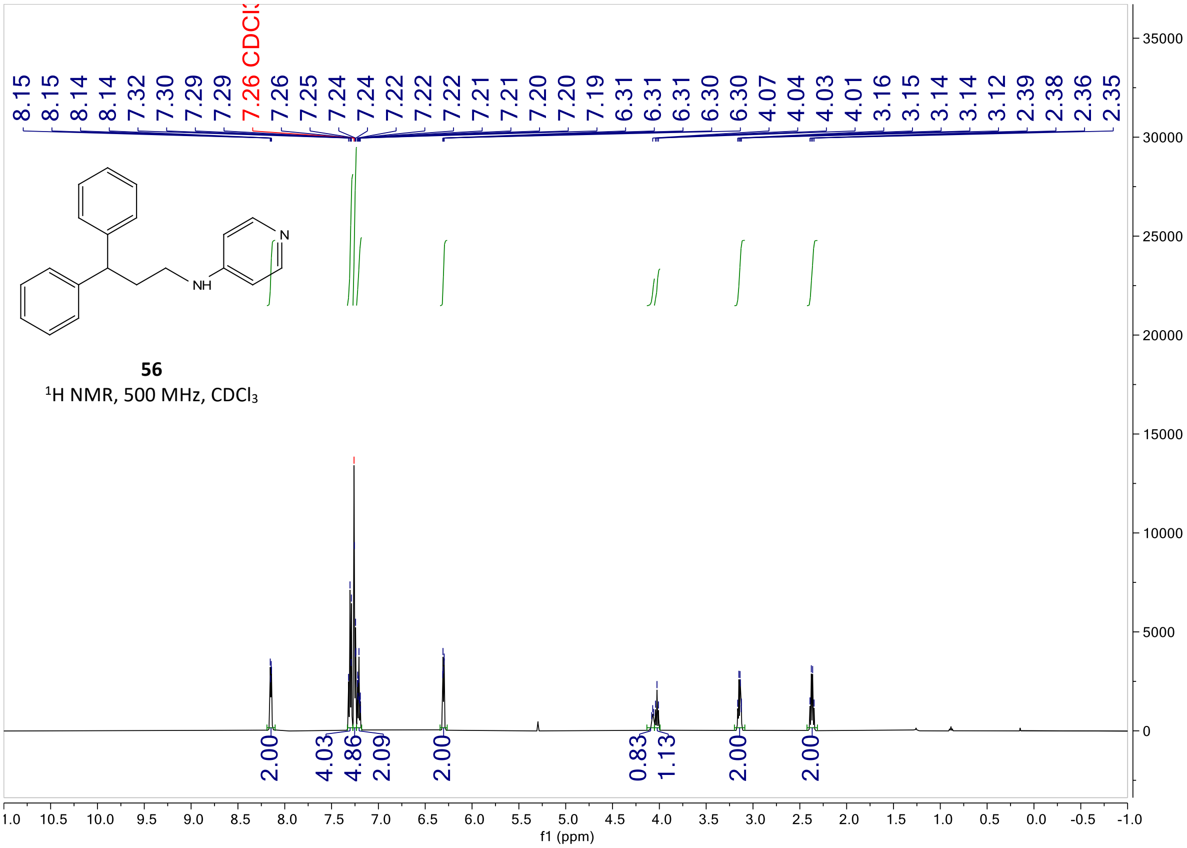

Supplementary Figure 176. ^{1}H NMR spectra of compound **56**

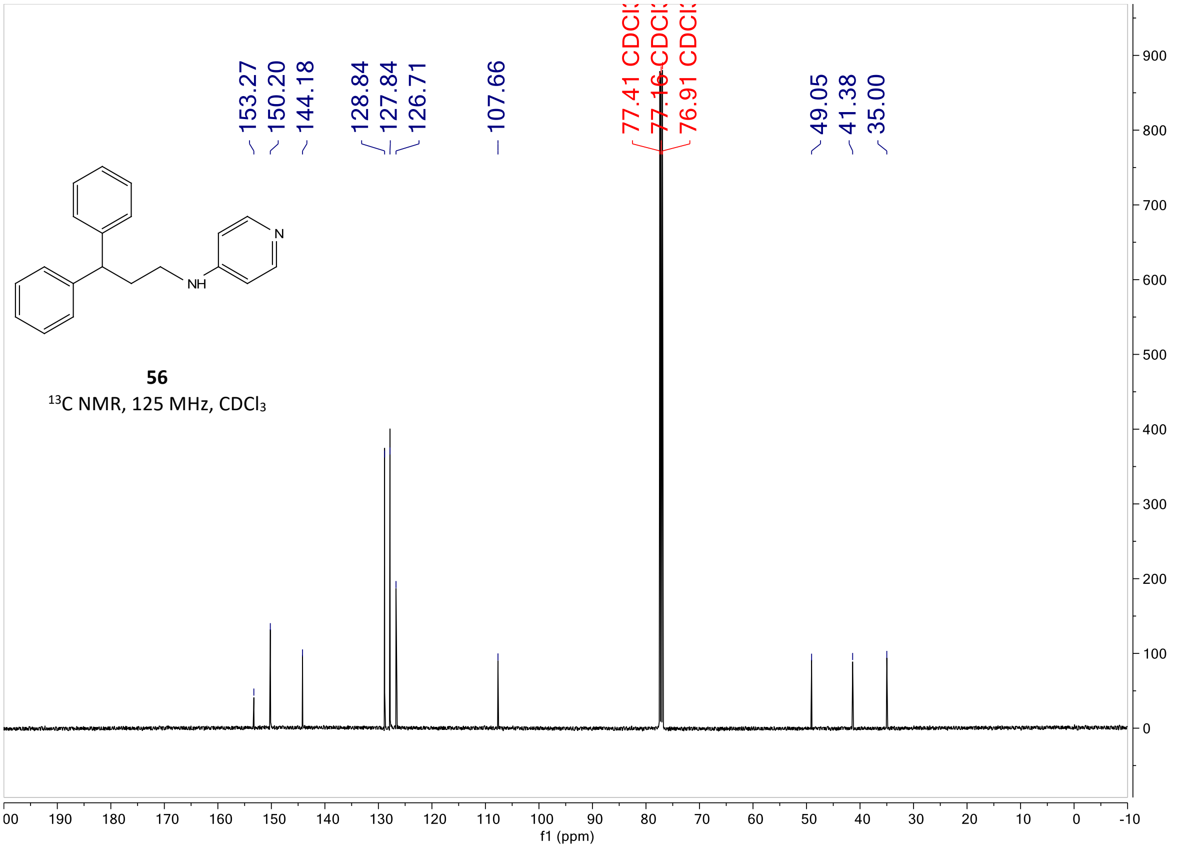

Supplementary Figure 177. ^{13}C NMR spectra of compound **56**

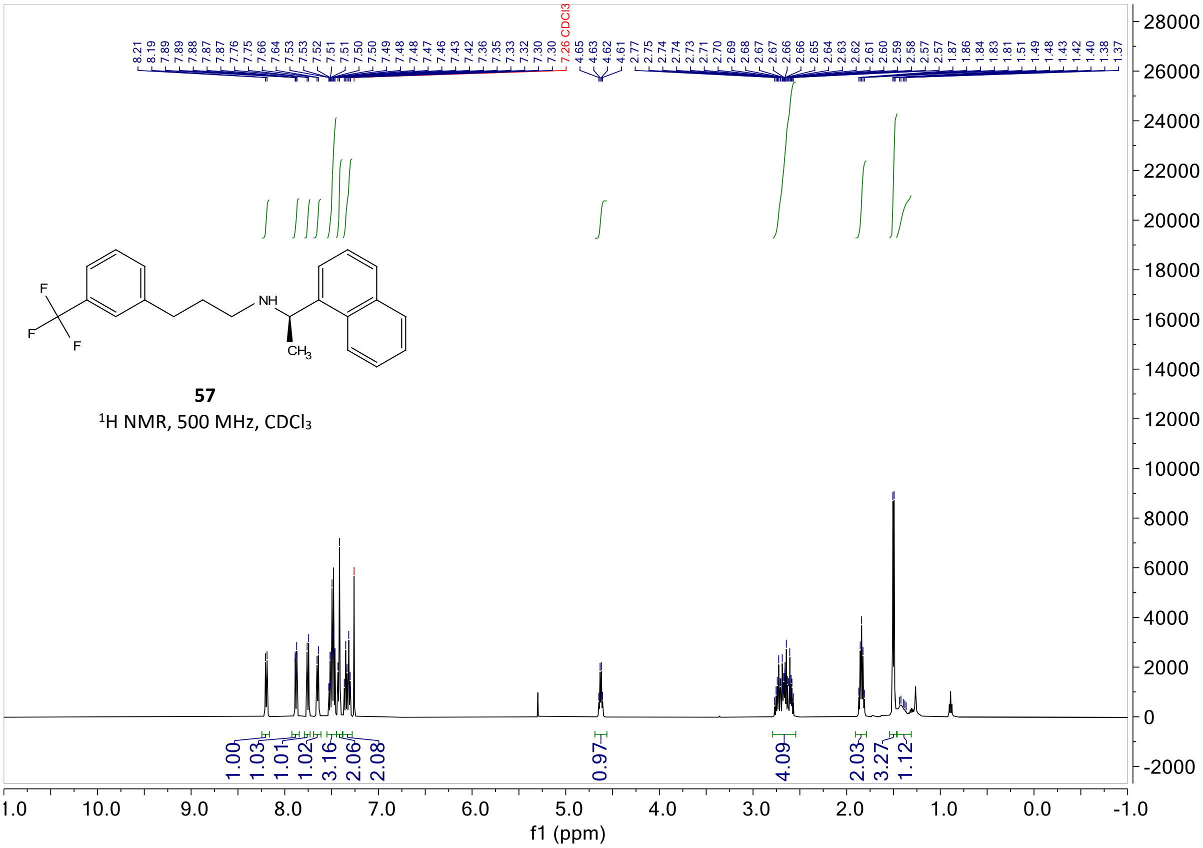

Supplementary Figure 178. ^{1}H NMR spectra of compound **57**

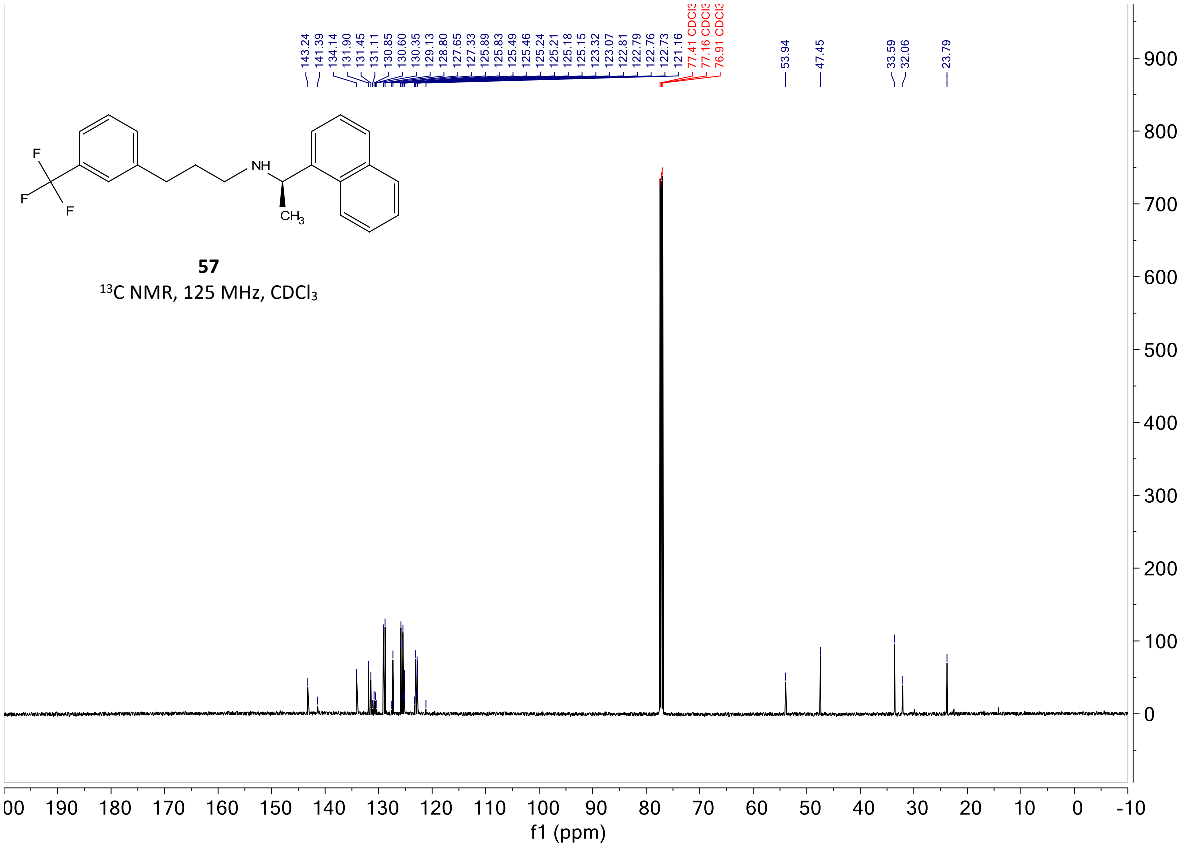

Supplementary Figure 179. ^{13}C NMR spectra of compound **57**

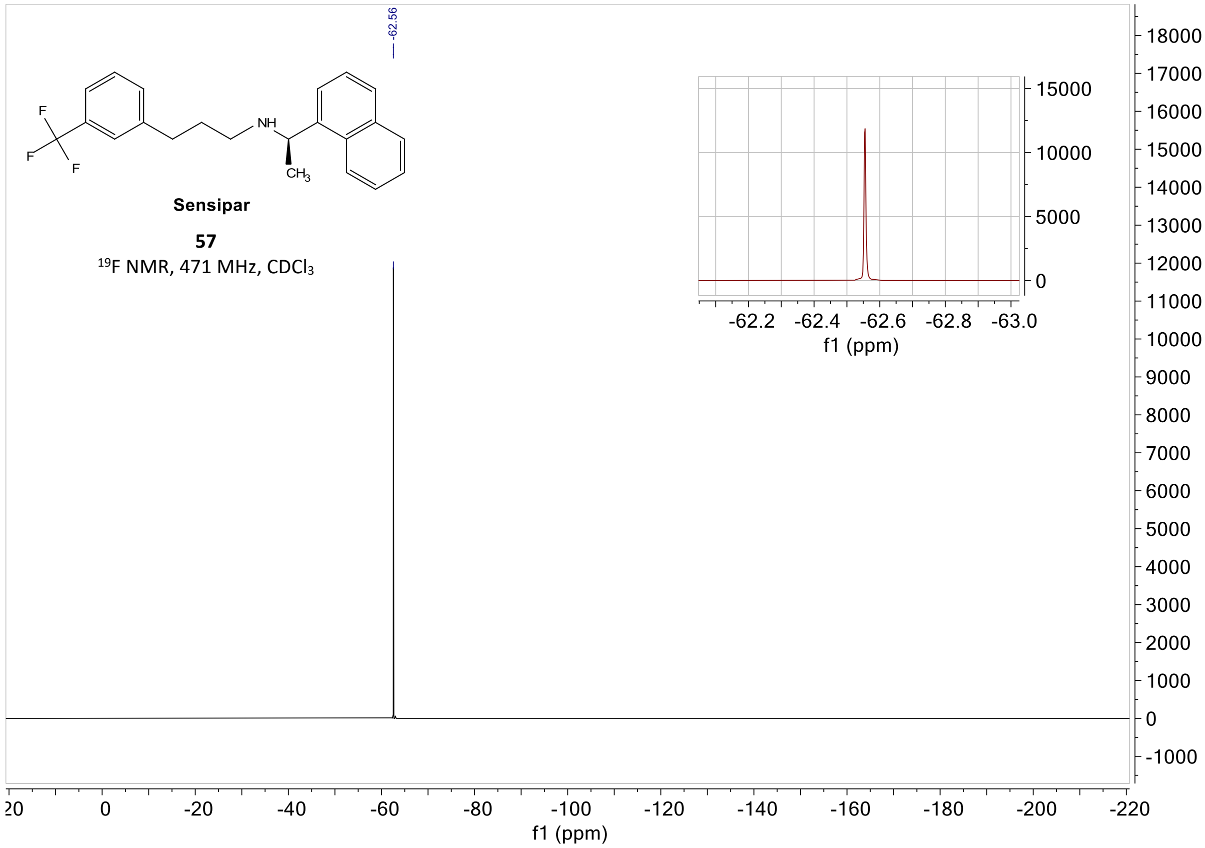

Supplementary Figure 180. ^{19}F NMR spectra of compound **57**

II. Supplementary References

[1] Kawashima, S.; Aikawa, K.; Mikami, K. *Eur. J. Org. Chem.* **2016**, 3166-3170.

[2] Grigg, R. D.; Hoveln, R. V.; Schomaker, J. M. *J. Am. Chem. Soc.* **2012**, *134*, 16131-16134.

[3] Lennox, A. J. J.; Lloyd-Jones, G. C. *Angew. Chem. Int. Ed.* **2012**, *51*, 9385-9388.

[4] Dai, J.-J.; Zhang, W.-M.; Shu, Y.-J.; Sun, Y.-Y.; Xu, J.; Feng, Y.-S.; Xu, H.-J. *Chem. Commun.* **2016**, *52*, 6793-6796.

[5] Smoum, R.; Rubinstein, A.; Srebnik, M. *Org. Biomol. Chem.* **2005**, *3*, 941-944.

[6] Yang, C.-T.; Zhang, Z.-Q.; Tajuddin, H.; Wu, C.-C.; Liang, J.; Liu, J.-H.; Fu, Y.; Czyzewska, M.; Steel, P. G.; Marder, T. B.; Liu, L. *Angew. Chem. Int. Ed.* **2012**, *51*, 528-532.

[7] Endo, K.; Hirokami, M.; Takeuchi, K.; Shibata, T. *Synlett*, **2008**, *20*, 3231-3233.

[8] Unsworth, P. J.; Leonori, D.; Aggarwal, V. K. *Angew. Chem. Int. Ed.* **2014**, *53*, 9846-9850.

[9] Presset, M.; Fleury-Brégeot, N.; Oehlrich, D.; Rombouts, F.; Molander, G. A. *J. Org. Chem.* **2013**, *78*, 4615-4619.

[10] Olsson, V. J.; Sebelius, S.; Selander, N.; Szabó, K. J. *J. Am. Chem. Soc.* **2006**, *128*, 4588-4589.

[11] Cazorla, C.; Métay, E.; Lemaire, M. *Tetrahedron* **2011**, *67*, 8615-8621.

[12] Hasegawa, K.; Kazayama, S.; Mochizuki, N.; Sada, T. Sip3 Receptor Antagonist, JP2005247691A, 2005.

[13] Crockett, M. P.; Tyrol, C. C.; Wong, A. S.; Li, B.; Byers, J. A. *Org. Lett.* **2018**, *20*, 5233-5237.
